# Stringent response governs the oxidative stress resistance and virulence of *Francisella tularensis*

**DOI:** 10.1371/journal.pone.0224094

**Published:** 2019-10-24

**Authors:** Zhuo Ma, Kayla King, Maha Alqahtani, Madeline Worden, Parthasarathy Muthuraman, Christopher L. Cioffi, Chandra Shekhar Bakshi, Meenakshi Malik

**Affiliations:** 1 Department of Basic and Clinical Sciences, Albany College of Pharmacy and Health Sciences, Albany, New York, United States of America; 2 Department of Microbiology and Immunology, New York Medical College, Valhalla, New York, United States of America; Midwestern University, UNITED STATES

## Abstract

*Francisella tularensis* is a Gram-negative bacterium responsible for causing tularemia in the northern hemisphere. *F*. *tularensis* has long been developed as a biological weapon due to its ability to cause severe illness upon inhalation of as few as ten organisms and, based on its potential to be used as a bioterror agent is now classified as a Tier 1 Category A select agent by the CDC. The stringent response facilitates bacterial survival under nutritionally challenging starvation conditions. The hallmark of stringent response is the accumulation of the effector molecules ppGpp and (p)ppGpp known as stress alarmones. The *relA* and *spoT* gene products generate alarmones in several Gram-negative bacterial pathogens. RelA is a ribosome-associated ppGpp synthetase that gets activated under amino acid starvation conditions whereas, SpoT is a bifunctional enzyme with both ppGpp synthetase and ppGpp hydrolase activities. *Francisella* encodes a monofunctional RelA and a bifunctional SpoT enzyme. Previous studies have demonstrated that stringent response under nutritional stresses increases expression of virulence-associated genes encoded on Francisella Pathogenicity Island. This study investigated how stringent response governs the oxidative stress response of *F*. *tularensis*. We demonstrate that RelA/SpoT-mediated ppGpp production alters global gene transcriptional profile of *F*. *tularensis* in the presence of oxidative stress. The lack of stringent response in *relA/spoT* gene deletion mutants of *F*. *tularensis* makes bacteria more susceptible to oxidants, attenuates survival in macrophages, and virulence in mice. This work is an important step forward towards understanding the complex regulatory network underlying the oxidative stress response of *F*. *tularensis*.

## Introduction

*Francisella tularensis* is a Gram-negative bacterium responsible for causing tularemia in the northern hemisphere. *F*. *tularensis* has long been developed as a biological weapon due to its ability to cause severe illness upon inhalation of as few as ten organisms and based on its potential to be used as a bioterror agent is now classified as a Tier 1 Category A select agent by the CDC [1–3]. The virulent strains are classified under *F*. *tularensis* subsp. *tularensis* (type A), and *F*. *tularensis* subsp. *holarctica* (type B), whereas avirulent strains belong to *F*. *novicida* or *F*. *philomiragia* [4]. The virulent SchuS4 strain belongs to *F*. *tularensis* subsp. *tularensis*, while the live vaccine strain (LVS) is derived from *F*. *tularensis* subsp. *holarctica*. *Francisella* is a facultative intracellular pathogen and can replicate in a variety of cell types; however, macrophages are the primary sites of replication [5,6]. The clinical presentation of tularemia depends on the route, dose, and infecting strain of *F*. *tularensis*. The ulceroglandular, oculoglandular, or the typhoidal forms of tularemia are not fatal. However, pneumonic tularemia is a highly acute and fatal form of the disease.

The unique intracellular life cycle of *Francisella* exposes the bacteria to oxidative stress conditions upon its entry, brief residence in the phagosomes, and escape from phagosomes into the cytosol where replication takes place [5]. *F*. *tularensis* genome encodes conventional antioxidant enzymes such as Fe- and CuZn-containing superoxide dismutases (SodB and SodC, respectively), catalase (KatG), and alkyl hydroperoxide reductase (AhpC) homologs to counter the oxidative stress generated at these distinct intracellular locations [7–10]. Unlike other bacterial pathogens, SoxR, an oxidative stress response regulator that regulates the expression of SodB, SodC, and SodA (manganese-containing Sod) is absent in *F*. *tularensis*. Instead, another oxidative stress regulator OxyR is present, which regulates the expression of KatG and AhpC in *F*. *tularensis* [11]. In *F*. *tularensis*, SodB, KatG, and AhpC are induced in response to oxidative stress and are secreted in abundance in the extracellular milieu and into the cytosol of *F*. *tularensis* infected macrophages [12]. Both the SodB and KatG are secreted by the Type I Secretion System of *F*. *tularensis* [13]. Expression of these primary antioxidant genes starts immediately upon phagocytosis of *F*. *tularensis* SchuS4 and remains significantly upregulated during phagosomal and cytosolic phases suggesting that *F*. *tularensis* experiences oxidative stress at both of these intracellular locations [14].

The stringent response facilitates bacterial survival under nutritionally challenging starvation conditions. The hallmark of stringent response is the accumulation of the effector molecules ppGpp and (p)ppGpp (guanosine-5'-diphosphate-3'-diphosphate and guanosine-5'-triphosphate-3'-diphosphate) known as stress alarmones [15]. The *relA* and *spoT* gene products generate alarmones in several Gram-negative bacterial pathogens. RelA is a ribosome-associated ppGpp synthetase that gets activated under amino acid starvation conditions whereas, SpoT is a bifunctional enzyme with both ppGpp synthetase and ppGpp hydrolase activities. During amino acid starvation, the presence of uncharged tRNA in the acceptor site of the ribosomes sends a signal to the RelA protein associated with ribosomes to catalyze the phosphorylation of GTP in conjunction with ATP as a donor to generate (p)ppGpp. The cytosolic SpoT is required for the basal synthesis of ppGpp during bacterial growth, (p)ppGpp degradation, and elevated synthesis of ppGpp under several stress conditions, including fatty acid and carbon starvation [16]. The ppGpp cause global reprogramming of cellular and metabolic function by binding to the β’-subunit of the RNA polymerase to activate or repress several genes or by interacting directly with proteins to promote adaptation, survival, and transmission in adverse growth conditions.

*Francisella* encodes a monofunctional *relA* and a bifunctional *spoT* gene. A single *relA* gene deletion mutant of *F*. *novicida* grows better than the wild type bacteria, but exhibits attenuated virulence in a mouse model of tularemia [17]. *F*. *tularensis* utilizes *relA/spoT* mediated ppGpp production to promote stable physical interactions between the components of the transcription machinery to activate the expression of virulence-associated genes encoded on Francisella Pathogenicity Island (FPI). It has also been shown that SpoT rather than RelA-dependent production of ppGpp is essential for the expression of virulence genes in *F*. *tularensis* [18]. In addition to *relA/spoT*; *migR*, *trmE*, and *chpA* genes of *F*. *tularensis* through unknown mechanisms are also involved in ppGpp production, and regulation of FPI encoded virulence-associated genes [19]. A global gene expression profile of *F*. *tularensis* SchuS4 under nutrient limitation conditions induced by serine hydroxamate showed ppGpp-dependent upregulation of genes involved in virulence, metabolism, and stress responses associated with downregulation of genes required for transport and cell division [20]. These studies demonstrate that stringent response under environmental and nutritional stresses increase FPI gene expression. However, the contribution of the stringent response in gene regulation and management of the oxidative stress response when *Francisella* is experiencing oxidative stress conditions is not known.

In this study, we investigated how stringent response governs the oxidative stress response of *F*. *tularensis*. Our results provide a link between the stringent and oxidative stress responses. We demonstrate that *relA/spoT*-mediated ppGpp production alters the global gene transcriptional profile of *F*. *tularensis* in the presence of oxidative stress. The lack of stringent response in *relA/spoT* gene deletion mutants of *F*. *tularensis* makes bacteria more susceptible to oxidants, attenuates survival in macrophages, and virulence in mice. This study provides evidence that the stringent response in *Francisella* contributes to oxidative stress resistance by regulating the production of several antioxidant enzymes, and enhances our understanding of the intracellular survival mechanisms of *F*. *tularensis*.

## Materials and methods

### Ethics statement

This study was carried out in strict accordance with the recommendations and guidelines of the National Council for Research (NCR) for care and use of animals. All the animal experiments were conducted in the centralized Animal Resources Facility of New York Medical College licensed by the USDA and the NYS Department of Health, Division of Laboratories and Research, and accredited by the American Association for the Accreditation of Laboratory Care. The use of animals and protocols were approved by the Institutional Animal Care and Use Committee (IACUC) of New York Medical College (Protocol Number 69-2-0914H). Mice were administered an anesthetic cocktail consisting of ketamine (5 mg/kg) and xylazine (4 mg/kg) and underwent experimental manipulation only after they failed to exhibit a toe pinch reflex. Mice exhibiting more than 25% weight loss, anorexia, dehydration and impairment of mobility were removed from the study and euthanized by approved means. Humane endpoints were also necessary for mice which survived at the conclusion of the experiments. Mice were administered an anesthetic cocktail of ketamine and xylazine intraperitoneally and then euthanized via cervical dislocation, a method that is consistent with recommendations of the Panel on Euthanasia of the American Veterinary Medical Association. In all experimental procedures, efforts were made to minimize pain and suffering.

### Bacterial strains and cultures

*F*. *tularensis* subspecies *holarctica* live vaccine strain (LVS; ATCC 29684; American Culture Collection, Rockville, MD) obtained from BEI Resources, Manassas, VA was used in this study. Bacterial cultures were grown on modified MH-chocolate agar plates (MMH) supplemented with 2% IsoVitaleX. For broth cultures, bacteria were grown in Muller-Hinton Broth (MHB). Working stocks of all bacterial cultures were prepared by growing to mid-log phase at 37°C with 5% CO_2_ in MHB, snap-frozen in liquid nitrogen, and stored at -80°C until further use. *Escherichia coli* DH5-α strain (Invitrogen) used for cloning was grown either on Luria-Bertani (LB) broth or LB agar plates (Invitrogen). For the selection of transformants, mutants, or transcomplemented strains, LB-broth or LB-agar media was supplemented with kanamycin (10μg/mL) or hygromycin (200μg/mL). The bacterial strains used in this study are shown in Table 1.

**Table 1:** List of bacterial strains and plasmid vectors used in this study.

*Francisella* Strains	Genotype	Source
*F*. *tularensis* LVS	Wild type strain	ATCC
Δ*relA* mutant	Deletion mutant of *F*. *tularensis* LVS *relA* gene	
Δ*relA/spoT* mutant	Deletion mutant of *F*. *tularensis* LVS *relA and spoT* gene	This study
*relA* transcomplement (Δ*relA* + p*relA*)	*F*. *tularensis* LVS, Δ*relA*, pMM016(pMP822+*relA*), Hygro^r^	This study
*spoT* transcomplement (Δ*relA/spoT* + p*spoT*)	*F*. *tularensis* LVS, Δ*relA/spoT*,pMM017(pMP822+ *spoT*), Hygro^r^	This study
***E*. *coli* Strains**		
DH5α	F– Φ80*lac*ZΔM15 Δ(*lac*ZYA-*arg*F) U169 *rec*A1 *end*A1 *hsd*R17 (rK–, mK+) *pho*A *sup*E44 λ– *thi*-1 *gyr*A96 *rel*A1	Invitrogen
**Plasmids**		
pMP822	*E*. *coli-Francisella* shuttle vector, Hygro^r^	[21]
pJC84	*E*. *coli-Francisella* suicide vector, Kan^r^	[14]
pMM014	pJC84 + fused flanking fragment of *relA* gene, Kan^r^	This study
pMM015	pJC84 + fused flanking fragment of *spoT* gene, Kan^r^	This study
pMM017	pMP822 + *relA*,Hygro^r^	This study
pMM018	pMP822 + *spoT*,Hygro^r^	This study
pMM019	pMP822 + *sodB*,Hygro^r^	This Study
pMM020	pMP822 + *katG*,Hygro^r^	This Study

### Generation of *F*. *tularensis* LVS Δ*relA* and Δ*relA*Δ*spoT* gene deletion mutants and transcomplemented strains

In-frame, single-gene deletion mutants of the *relA* gene (*FTL_0285*) (Δ*relA*), and a double gene deletion mutant of *relA* and *spoT* gene (*FTL_1413*) (Δ*relA*Δ*spoT*) of *F*. *tularensis* were generated using a previously described allelic replacement method [10,22,23]. For confirmation of the mutants, a duplex colony PCR was performed using internal *relA* and/or *spoT* gene-specific primers along with *sodB* gene-specific primers as internal controls. *In-frame* deletions of both the *relA* and *spoT* genes were confirmed by DNA sequencing of the flanking regions. The deletion strain was designed in a way to preserve the downstream open reading frames to avoid any potential polar effects due to gene deletions.

The Δ*relA* gene deletion mutant was transcomplemented with full-length *relA* gene cloned in the *BamHI* and *Xhol* restriction sites of *E*. *coli-Francisella* vector pMP882. The Δ*relA*Δ*spoT* was transcomplemented with a full-length *spoT* gene cloned in the pMP822 vector in a similar fashion. The transcomplementation vectors were electroporated into the *F*. *tularensis relA* and Δ*relA*Δ*spoT* gene deletion mutants and selected on MMH agar supplemented with 200μg/mL hygromycin. The transcomplementation was confirmed by PCR. The primer sequences used for generation and screening of mutants and transcomplementation are shown in Tables 2 and 3.

**Table 2:** List of primers used for the generation of gene deletion mutants.

Primers	Sequence	Purpose
***Ft relA* upstream fragment**		
MP494	5’-CAAggatccTGGTGATAACATTATTACCACCGC-3’	F-primer with a *BamH*I at 5’ end
MP495	5’-TTGCATAATTACCCACTTTTTG-3’	R- primer
***Ft relA* downstream fragment**		
MP496	5’-CAAAAAGTGGGTAATTATGCAAGAGGTCTGCTAATAATTAACAA-3’	**F**-primer
MP497	5’-TGATgtcgacAAATGGTATACTCAACCCTGCC-3’	R-Primer with a *Sal*I site
***Ft spoT* upstream fragment**		
MP512	5’-CAAagatct TTATGCAAAGAGGTCTTCGGTT-3’	F-primer with a *Bgl*II site
MP513	5’-GAAACAAAACATATTTAGCAAAAT-3’	R-primer
***Ft spoT* gene downstream fragment**		
MP514	5’-ATTTTGCTAAATATGTTTTGTTTCTAAATTATTATTAAATATTAGTTCTTG-3’	F-primer
MP515	5’-TGATgtcgacTGAGCAAATGTCAAAAATGGC-3’	R-Primer with a *Sal*I site
***Ft relA* mutant screening**		
MP037	5’-CCGGATCCATGAAATTTGAATTACCAAAAC-3’	F-primer for *sodB* as control
MP038	5’-CGCTGCAGCTAATCAGCGAATTGCTCAGAAAC-3’	R-primer for *sodB* as control
MP506	5’-GGGTTATCACAAATAATGTTGATG-3’	F-primer for *relA*
MP507 MP509	5’-ATCTAAGACTGTCGAACCTTCAGC-3’ 5’-CAAGGCAATGAAATAAAATCAAC-3’	R-primer for *relA* For sequencing
***FtspoT* mutant screening**		
MP518	5’-AATGCAGGTTGGCTGACAAA-3’	F-primer for *spoT*
MP519 MP521	5’-CGCCAGAAAAACTTACAGTTACTA-3’ 5’-CGCTGATAGAAAAATCGATGAGG-3’	R-primer for *spoT* For sequencing

**Table 3:** List of primers used for the generation of complementation constructs.

**Complementation for *Ft*.*relA***		
MP510	5’-CAAggatccATGCAAGTTATTGACTCTAAACT-3’	F-primer for *relA* with a *BamH*I site
MP511	5’-TGATctcgagTTAGCAGACCTCTTCATTATCATC-3’	R-primer for *relA* with an *Xho*I site
**Complementation for *Ft*.*spoT***		
MP522	5’-CAA ggatcc ATGTTTTGTTTCTATGACCTTAA-3’	F-primer for *spoT* with a *BamH*I site
MP523	5’-CGACctgcagTTACTTATTTAATATTCTTTCGAT-3’	R-primer for *spoT* with an *Pst*I site

### Detection of ppGpp by high-performance liquid chromatography (HPLC)

The ppGpp extraction was done as follows. Briefly, bacterial cultures of *F*. *tularensis* LVS, Δ*relA*, relA/spoT, and Δ*relA*Δ*spoT* mutants were grown to OD_600_ of 0.5 at 37°C in 10 ml MH-broth. Then 5 ml of cultures were directly mixed with 0.5 ml of 11M formic acid followed by freezing on dry ice and kept at -80°C until use. After that, the mixtures were thawed and allowed to sit on ice for 30 minutes. 1ml aliquots of mixtures were centrifuged at 4C at maximum speed for 5 minutes. The supernatant was then filtered through 0.2μM filters and stored at -80°C until HPLC analysis.

The ppGpp was quantified by HPLC. Isocratic BEH HILIC C18-HPLC column (5 μm, 4.6×150 mm) was used to determine the retention time and absorbance spectrum of a ppGpp standard (TriLink Biosciences). The presence of degradation products—ppGpp, and most likely, pppGpp—was apparent at 2.01 min retention time. The column was run with buffer containing 0.36 M NH_4_H_2_PO_4,_ pH 3.4, 2.5% acetonitrile at 26°C with a flow rate of 1.5 mL/minute with a 30 minutes run time. Samples were run for three separate times. Relative levels from samples were represented for comparison.

### Bacterial growth curves

Wild type *F*. *tularensis* LVS, the Δ*relA* mutant, the Δ*relA*Δ*spoT* mutant and the corresponding transcomplemented strains were grown on MH chocolate agar plates. The bacterial cultures were then resuspended to an OD_600_ of 0.05 in MHB. The cultures were either left untreated or treated with 500μM serine hydroxamate, 0.5 and 1mM hydrogen peroxide (H_2_O_2_). All bacterial cultures were incubated at 37°C with shaking (150 rpm), and the OD_600_ values were recorded for 28 hours.

### Disc diffusion assays

For disc diffusion assays, bacterial cultures of the wild type *F*. *tularensis* LVS, the Δ*relA* mutant, the Δ*relA*Δ*spoT* mutant, and the corresponding transcomplemented strains grown on MH-chocolate agar plates adjusted to an OD_600_ of 2.0 in MHB. Two hundred microliters of the bacterial suspensions were spread onto MH-chocolate agar plates using a sterile cotton swab. Sterile filter paper discs were placed onto the agar and were impregnated with 10μL of paraquat (0.88μg/mL), pyrogallol (40μg/mL), menadione (0.39μg/μL), tert-butyl hydroperoxide (TBH; 21.9% solution), and cumene hydroperoxide (CHP; 1.25% solution). The plates were incubated for 72 hours at 37°C and 5% CO_2_. The zones of inhibition around the discs were measured to determine the sensitivity to the compounds tested.

### Bacterial killing assays

For bacterial killing assays, equal numbers of bacteria (1x10^9^ CFU/mL) were diluted in MHB and exposed to H_2_O_2_ (1.0mM), paraquat (1.0mM), pyrogallol (1.0mM), or a temperature of 37 or 48°C. Both treated and untreated bacterial suspensions were allowed to incubate for 1 hour and 3 hours. The cultures were then serially diluted 10-fold in PBS and plated on MH-chocolate agar plates and incubated for 72 hours at 37°C with 5% CO_2_. Viable bacteria were enumerated by counting the colonies and were expressed as Log_10_ CFU/mL.

### Macrophage cell culture assays

RAW macrophage cell line and Bone Marrow-Derived Macrophages (BMDMs) isolated from wild type C57BL/6 or *phox*^*-/-*^ mice were infected with wild type *F*. *tularensis* LVS, the Δ*relA* mutant, the Δ*relA*Δ*spoT* mutant, and the corresponding transcomplemented strains at a multiplicity of infection (MOI) of 100. The extracellular bacteria were killed by treating with gentamycin after 2 hours of infection to allow the intracellular bacteria to replicate for 4 or 24 hours. The cells were lysed at both 4- and 24-hours post-infection, diluted 10-fold and plated for enumeration of bacterial colonies.

### Mouse survival studies

Mice experiments were conducted at the Animal Resource Facility at New York Medical College according to approved IACUC protocols. All *in vivo* survival studies were conducted in wild type C57BL/6 mice aged 6 to 8 weeks. Before inoculation, mice were anesthetized with a cocktail of Ketamine and Xylazine. The mice were then intranasally inoculated with doses ranging from 1x10^5^ to 1x10^6^ of the wild type *F*. *tularensis* LVS, the Δ*relA* mutant, the Δ*relA*Δ*spoT* mutant in 20μL PBS. Mice were monitored up to 21 days post-infection for morbidity and mortality. Mice were weighed daily to monitor the infection. The survival results were expressed as Kaplan-Meier survival curves, and the data were analyzed using the Log-rank test.

### RNA sequencing

Overnight cultures of wild type *F*. *tularensis* LVS and the Δ*relA*Δ*spoT* mutant were adjusted to an OD_600_ of 0.2 and were grown for 3 hours at 37°C with shaking in 10 ml MH-broth in the absence or presence 1mM H_2_O_2_. The bacterial cells were pelleted, and total RNA was purified using the Purelink RNA Mini Kit (Ambion). The contaminating DNA from RNA preparations was removed using TURBO DNA-free kits (Invitrogen). The experiments were repeated three times, and cumulative data were analyzed for differential expression of genes. The RNA samples were submitted to the Genomics Core Facility at New York Medical College for RNA sequencing. EDGE-pro software was used to determine expression levels from RNA-seq data. The comparisons were made for differential expression of genes between untreated *F*. *tularensis* LVS and the Δ*relA*Δ*spoT* mutant (-H_2_O_2_) or upon treatment with H_2_O_2_ (+H_2_O_2_). DESeq2 software was used for pairwise detection and quantification of differential gene expression. A cutoff for multiple testing corrected *P*-value (adjusted *P*-value) of <0.05 was used to determine statistical significance and filter differentially expressed genes.

### Transcriptional analysis of the target genes

The RNA from wild type *F*. *tularensis* LVS, the Δ*relA*, Δ*relA*Δ*spoT* mutants and the corresponding complemented strains were isolated as described above. cDNA was synthesized using the iScript cDNA Synthesis Kit (Ambion). Quantitative real-time PCR (qPCR) was performed using iQ SYBR Green Supermix (BioRad) to determine the transcriptional levels of genes of interest. The expression of *tul4* gene was used as an internal control [18]. Relative levels are represented as fold change and were calculated as follows: 2^−ΔΔ*CT*^ = 2^-[Δ*CT* (mutant) − Δ*CT* (WT)]^, where Δ*C*_*T*_ = *C*_*T*_ target gene − *C*_*T*_ internal control. The primer sequences used for qRT-PCR are shown in Table 4.

**Table 4:** List of primers used for transcriptional analysis by qRT-PCR.

***tul4***		
MP029	5’-TCGCAGGTTTAGCGAGCTGTTCTA-3’	F-primer
MP030	5’-ACAGCAGCAGCTTGCTCAGTAGTA-3’	R-primer
**Peroxidase/Catalase (*FTL_1504*, *katG*)**		
MP077	5’-CCTGCCAAATAAAGTTTTGCTC-3’	F-primer
MP078	5’-AGCTCACCAATGGACTCCTAC-3’	R-primer
**Superoxide dismutase [Fe](*FTL_1791*,*sodB*)**		
MP101	5’-GGCGGAATATTTAATAACGCTGC-3’	F-primer
MP102	5’-GTGCTCCCAAACATCAAAAG-3’	R-primer
**ClpB protein (*FTL_0094*)**		
MP284	5’-TTTCATAATCCTGACCTTGCA-3’	F-primer
MP285	5’-GGCGGCTATCTAACTGAACAT-3’	R-primer
**Universal stress protein (*FTL_0166*)**		
MP290	5’-TTCCATGAGTCGCATGTGAA-3’	F-primer
MP291	5’-TTGACTGTGTTGCTCCATTTG-3	R-primer
**RNA polymerase sigma-32 factor (*FTL_0851*,*rpoH*)**		
MP296	5’-TCAATAGCGGACCTTATCCA-3’	F-primer
MP297	5’-TTCTCCGAGCTTAACCAACCA-3’	R-primer
**Oxidative stress transcriptional regulator (*FTL_1014*)**		
MP528	5’-ATGCCTAAAATTCTCCCTGC-3’	F-primer
MP529	5’-GACCTTCATCAAGAAGCATAAGA-3’	R-primer
**Thioredoxin 1 (*FTL_1224*)**		
MP286	5’-CAGACGAAGCTAATTTTGACAAAC-3’	F-primer
MP287	5’-AGTCTCAAGTTGCTTACGATTTTT-3’	R-primer
**Glutaredoxin 2 (*FTL_0923*)**		
MP534	5’-TAGAATTGGCTCAAAACGAGTACC-3’	F-primer
MP535	5’-CTAAGTAAAGCATCGAAATCACCA-3	R-primer
**Glutaredoxin 1 (*FTL_0985*)**		
MP538	5’-GTTGTCCATATTGTGTTTGGGC-3’	F-primer
MP539	5’-CCTTAAGCTCTGTAAAGCCACCTA-3	R-primer
**Ferric uptake regulation protein (*FTL_1831*,*Fur*)**		
MP542	5’-AGCATTTGAGTCCTGATGATGT-3’	F-primer
MP543	5’-CTTCGATTCCTGGGCTATAGAACT-3	R-primer
**Glutathione peroxidase (*FTL_1383*)**		
MP544	5’-GTAAGTGCGGTTTTACTAAGCAG-3’	F-primer
MP545	5’-TGGCTCAGCATCTTTACCAT-3’	R-primer
**ATP-dependent Clp protease subunit X (*FTL_0893*,*clpX*)**		
MP550	5’- TACTGAGGCTGGTTATGTTGGTGA -3’	F-primer
MP551	5’- TTAATAATGCCTGCTGCACAC -3’	F-primer
**ATP-dependent protease, ATP-binding subunit (*FTL_0964*,*hlsU*)**		
MP556	5’- TGGCTCCAAAAACTATAGGCGTA -3’	F-primer
MP557	5’- TTCTAGAGCACGAGCCTTTATGTC -3	R-primer
**Chaperone protein dnaJ (*FTL_1192*)**		
MP564	5’- GCCAAGGTACTATTCGTAGACAAC -3’	F-primer
MP565	5’- ATTAGAGCCAGAATCACCCTCA -3’	R-primer
**Aminotransferase,class II (*FTL_1219*)**		
MP570	5’- CATGGAATGTCAATAATTGGTAAG -3’	F-primer
MP571	5’- AGGACCTGAAAATGTGTACGTAGT -3’	R-primer
**Intracellular growth locus, subunit A (*FTL_0111*, *iglA1*)**		
MP572	5’- TCGCATATAGAGAGGTCAGAAGAG -3’	F-primer
MP573	5’- CGCTCTGATTTCAGGAACTTT -3’	R-primer
**Intracellular growth locus, subunit A (*FTL_1157*, *iglA2*)**		
MP574	5’- GGATGCAAAAAAAGAGTTCGC -3’	F-primer
MP575	5’- GCAACAGCATCAGGTCTAAAATC -3’	R-primer
**Conserved hypothetical protein (*FTL_0126*, *pdpA*)**		
MP576	5’- AGATAGTGCGATACAAAGATCAAGT -3’	F-primer
MP577	5’- CCAGCCATTTATGCCGTAGATA -3’	R-primer
**Hypothetical protein (*FTL_0449*, *pigR*)**		
MP578	5’- GCTCAGCGAGAGAGATCTTATT -3’	F-primer
MP579	5’- TCATATTTATCCAGCAGCGACTA -3’	R-primer
**Two-component response regulator(*FTL_0552*, *pmrA*)**		
MP580	5’- TCTGGATTGTATGATATAGTCGTCTTAG -3’	F-primer
MP581	5’- CAGATCTAGTATCAATACGACGAGAAA -3’	R-primer
**Macrophage growth locus, subunit A *(FTL_1185*, *mglA)***		
MP582	5’- ATCCCTTTCCTCCGATGTTTC -3’	F-primer
MP583	5’- GCATCTGGATTACTTTCTGCATTTA -3’	R-primer

### Statistical analysis

All results were expressed as means ± S.E.M. or S.D. One-Way ANOVA followed by Tukey-Kramer Multiple Comparison tests and Student’s t-test were used for statistical analysis of the data. Survival data were analyzed using the Log-rank test and graphed using Kaplan-Meier survival curves. A *P* value of less than 0.05 was considered significant.

## Results

### Generation and characterization of *ΔrelA* and *ΔrelAΔspoT* mutants of *F*. *tularensis*

**Fig 1:**
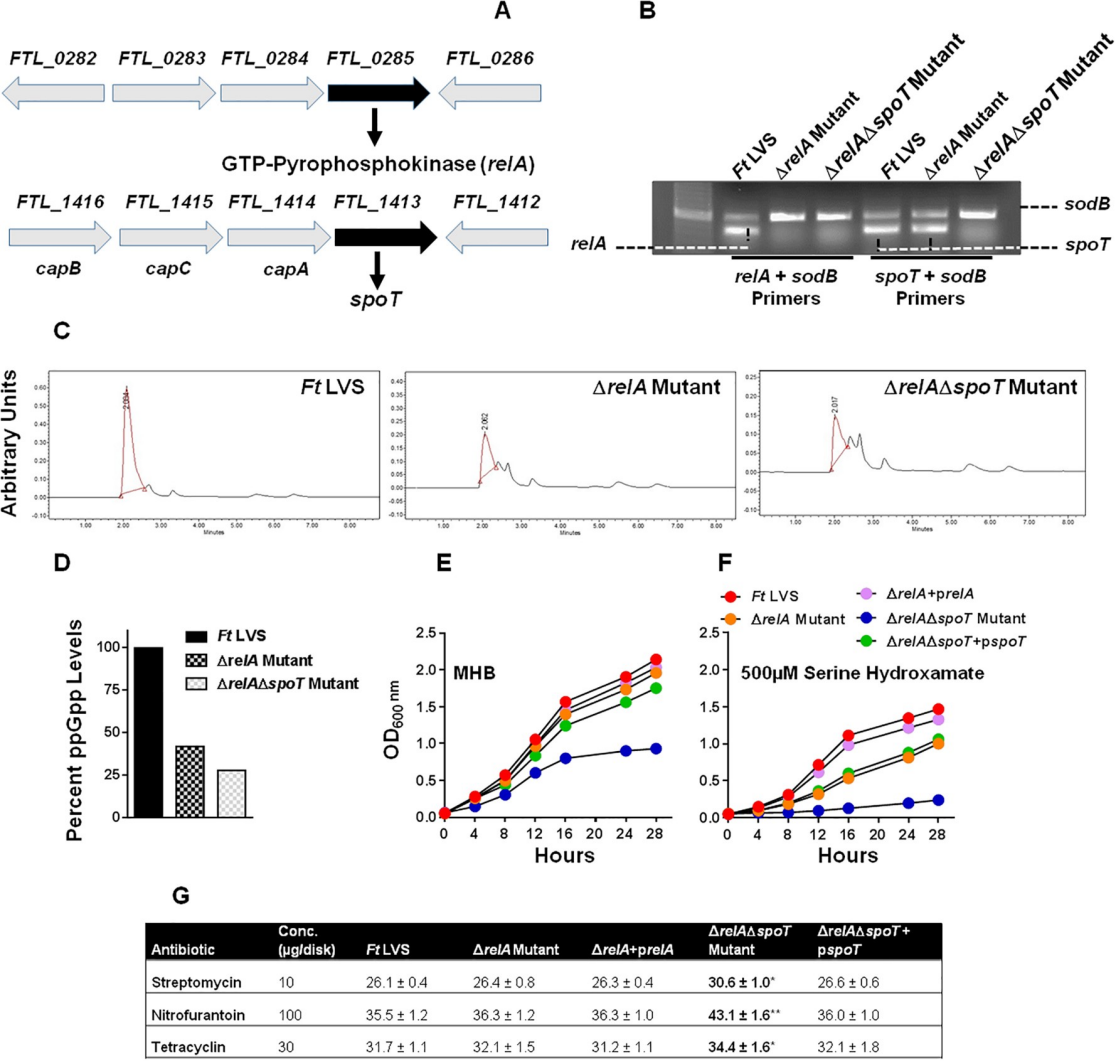
Generation and characterization of *ΔrelA* and *ΔrelAΔspoT* mutants of *F*. *tularensis*. (A) The genomic organization of the *relA* and the *spoT* genes of the *F*. *tularensis* LVS. (B) Multiplex colony PCR using *relA* and *spoT* gene-specific primers and *sodB* gene primers as internal controls. Amplification of the *sodB* gene confirmed the presence of the DNA template in the reaction, whereas the absence of the *relA* gene product in the Δ*relA* and Δ*relA*Δ*spoT* mutants confirmed the deletion of the *relA* gene and that of *spoT* confirmed *spoT* gene deletion in the Δ*relA*Δ*spoT* mutant. (C and D) Determination of ppGpp production and its quantitation in the *F*. *tularensis* (*Ft*) LVS, the Δ*relA* and the Δ*relA*Δ*spoT* mutants by HPLC. (E and F) Growth curves of the *Ft* LVS, the Δ*relA*, the Δ*relA*Δ*spoT* mutants, and the transcomplemented strains in the presence of the indicated concentrations of serine hydroxamate. (G) The susceptibility of the *Ft* LVS, the Δ*relA* and the Δ*relA*Δ*spoT* mutants and the transcomplemented strains to indicated antibiotics was tested by disk diffusion assay. The plates were incubated for 48–72 hours, and the zones of inhibition around the discs were measured. The results shown in C, D, E, F, and G are representative of three independent experiments with identical results. The data in G are represented as Mean±S.D. (n = 3 biological replicates) and were analyzed using one-way ANOVA (*P<0.05; **P<0.01).

The *relA* gene (*FTL_0285*) encoding GTP Pyrophosphokinase in *Francisella* is 647 base pairs in length and is transcribed as a single transcriptional unit; while the *spoT* gene (*FTL_1413*) encoding a guanosine-3’ 5’-bis (diphosphate) 3’-phosphohydrolase/(p)ppGpp synthase is 704 bp in length is transcribed as an operon along with *capA*, *capB* and *capC* genes (Fig 1A). To investigate the role of stringent response in oxidative stress resistance of *F*. *tularensis* LVS, we constructed unmarked, in-frame *relA* single gene deletion (Δ*relA*) and *relA spoT* (Δ*relA*Δ*spoT*) double gene deletion mutants. The gene deletions in single and double mutants were confirmed by PCR (Fig 1B), followed by DNA sequencing. The transcomplemented strains were constructed by providing full copies of the *relA* gene in the Δ*relA* mutant, and *spoT* gene in the Δ*relA*Δ*spoT* mutant. To determine the ability of the wild type *F*. *tularensis* LVS, Δ*relA* and the Δ*relA*Δ*spoT* mutants to produce ppGpp, the bacterial strains were grown to late exponential phase, and ppGpp production was determined by HPLC. The production of ppGpp was dropped to 45 and 28% in the Δ*relA* and the Δ*relA*Δ*spoT* mutants respectively, as compared to that observed for the wild type *F*. *tularensis* LVS, indicating that Δ*relA* and more so the Δ*relA*Δ*spoT* mutant is deficient in ppGpp production (Fig 1C and 1D). The growth characteristics of the wild type *F*. *tularensis* LVS, the Δ*relA*, the Δ*relA*Δ*spoT* double mutant and the transcomplemented strains were determined by growing in MH-broth in the absence or presence of 500μM of serine hydroxamate, a serine homolog responsible for inducing amino acid starvation-like conditions. The Δ*relA* mutant did not exhibit any growth defect and grew similarly to the wild type *F*. *tularensis* LVS when grown in the MH-broth. The Δ*relA*Δ*spoT* mutant showed retarded growth and entered the stationary phase after 16 hours of growth. Transcomplementation with *spoT* gene restored the growth of the Δ*relA*Δ*spoT* mutant as well as prevented its early entry into the stationary phase (Fig 1E). Growth of all *Francisella* strains was slightly reduced in the presence of serine hydroxamate; however, the Δ*relA*Δ*spoT* mutant failed to grow in its presence. Transcomplementation of the Δ*relA*Δ*spoT* with the *spoT* gene restored its growth in the presence of serine hydroxamate (Fig 1F). Sensitivities of the wild type *F*. *tularensis* LVS, the Δ*relA*, the Δ*relA*Δ*spoT* mutants and the transcomplemented strains were also tested against streptomycin, nitrofurantoin, and tetracycline by disc diffusion assays. It was observed that the Δ*relA*Δ*spoT* mutant exhibited enhanced sensitivities towards all the three antibiotics tested as compared to the wild type *F*. *tularensis* LVS or the Δ*relA* mutant. Transcomplementation of the Δ*relA*Δ*spoT* mutant restored the sensitivities similar to those observed for the wild type or the Δ*relA* mutant (Fig 1G). Collectively, these results demonstrate that induction of RelA/SpoT-mediated stringent response associated with the production of ppGpp is required for growth of *F*. *tularensis* under normal growth conditions, amino acid starvation, as well as resistance towards antibiotics.

### The transcriptional profile of the Δ*relA*Δ*spoT* mutant of *F*. *tularensis*

**Fig 2:**
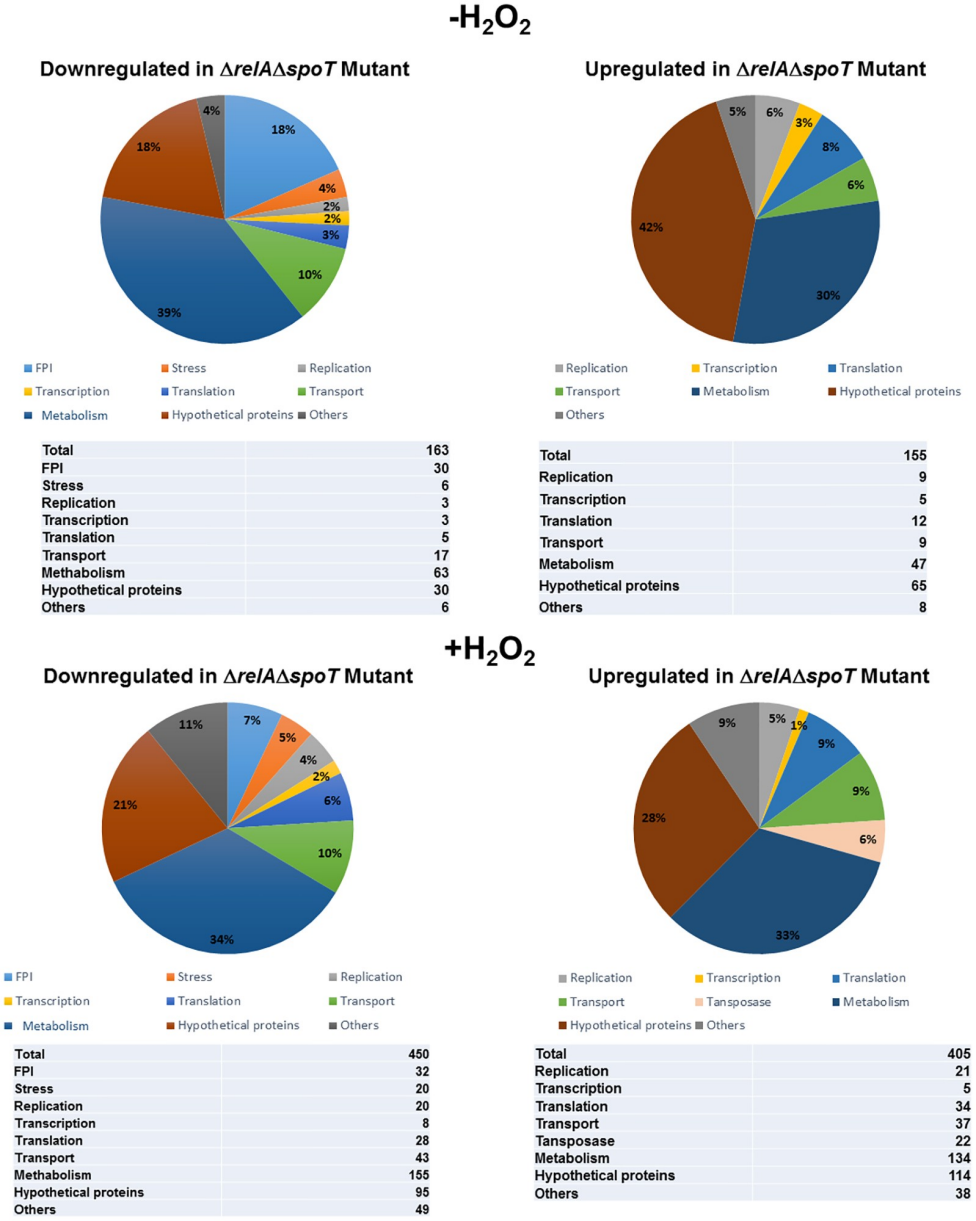
The transcriptional profile of the Δ*relA*Δ*spoT* mutant of *F*. *tularensis*. A total number of differentially expressed genes in the Δ*relA*Δ*spoT* mutant compared to the wild-type *F*. *tularensis* LVS.

RNA sequencing was used to obtain the transcriptional profiles of the wild type *F*. *tularensis* LVS and the Δ*relA*Δ*spoT* mutant with or without the treatment with H_2_O_2_. Since the Δ*relA*Δ*spoT* mutant grows slower than the wild type *F*. *tularensis* LVS in the presence of H_2_O_2,_ 3-hour treatment with H_2_O_2_ was selected. At this time point post-treatment, the viability of the mutant strain is not affected significantly. An adjusted *P* value of *<0*.*05* was used as a cut-off to verify differentially expressed genes in the Δ*relA*Δ*spoT* mutant. A total of 318 genes were differentially expressed in untreated Δ*relA*Δ*spoT* mutant as compared to the wild type *F*. *tularensis* LVS. Of these 163 genes (51%) were downregulated while 155 genes (49%) were upregulated in the Δ*relA*Δ*spoT* mutant. The majority of the downregulated genes were involved in metabolism (n = 63), hypothetical proteins (n = 30), Francisella Pathogenicity Island (FPI) genes (n = 30) as well as transport, replication, transcription, translation and stress response genes. The upregulated genes mostly belonged to the hypothetical proteins (n = 65) metabolism (n = 47) and translation (n = 12) categories (Fig 2). Treatment of the Δ*relA*Δ*spoT* mutant with H_2_O_2_ resulted in differential expression of a total of 855 genes in the Δ*relA*Δ*spoT* mutant as compared to the wild type *F*. *tularensis* LVS. Of the 450 (53%) downregulated genes, majority of the genes belonged to metabolism (n = 155), hypothetical proteins (n = 95), others (n = 49) and FPI (n = 32) categories. The genes that were upregulated following exposure to H_2_O_2_ also belonged to the similar categories except for the FPI genes (Fig 2). The overall gene transcription profiles of the Δ*relA*Δ*spoT* mutant as compared to the wild type *F*. *tularensis* in the absence or the presence of oxidative stress-induced upon exposure to H_2_O_2_ are also shown in S1 and S2 Tables. Collectively these results demonstrate that RelA and SpoT not only control the expression of several genes of *F*. *tularensis* under normal growth conditions but also regulate genes both positively and negatively when the bacteria are exposed to oxidative stress.

### Expression of the FPI genes in Δ*relA*Δ*spoT* mutant of *F*. *tularensis*

**Fig 3:**
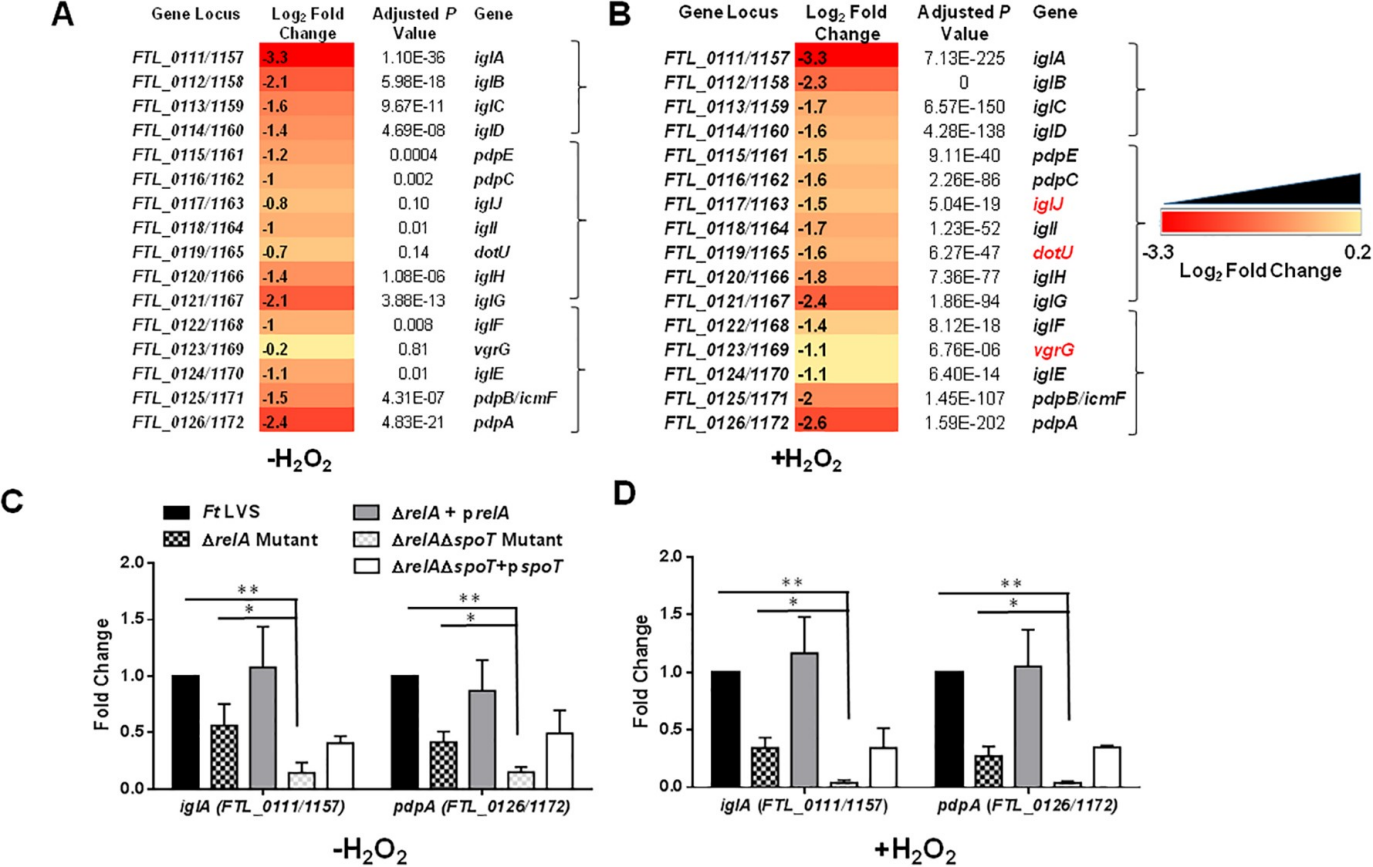
Expression of the FPI genes in Δ*relA*Δ*spoT* mutant of *F*. *tularensis*. RNAseq analysis comparing the differential expression of genes encoded on FPI in untreated (A) and H_2_O_2_ treated (B) Δ*relA*Δ*spoT* mutant compared to that of wild type *F*. *tularensis* (*Ft*) LVS. The data are represented as Log_2_ fold change and are cumulative of three independent experiments. The brackets on the right represent operon and genes in red fonts indicate significantly downregulated expression in the Δ*relA*Δ*spoT* mutant compared to that of wild type *Ft* LVS upon treatment with H_2_O_2_. (C and D) Quantitative reverse transcriptase PCR (qRT-PCR) was performed to evaluate the transcription of select genes. The amount of target gene amplification was normalized to a *tul4* gene internal control. The relative mRNA levels are presented as Mean±SD (n = 3 biological replicates). A and B, DESeq2 software was used for pairwise detection and quantification of differential gene expression. A cutoff for multiple testing corrected *P*-value (adjusted *P*-value) of <0.05 was used to determine statistical significance and filter differentially expressed genes. Data shown in C and D were analyzed using ANOVA. (*P<0.05, **P<0.01).

We further analyzed the differential expression of the FPI genes in the Δ*relA*Δ*spoT* mutant under normal as well as oxidative stress conditions created by exposing the bacteria to H_2_O_2._ The expression of *iglA*, *B*, *C*, *D*, *pdpE*, *C*, *IglI*, *H*, *G*, *F*, *E*, *pdpB/icmF* and *pdpA* genes encoded on FPI was significantly downregulated in untreated Δ*relA*Δ*spoT* mutant as compared to the wild type *F*. *tularensis* LVS. However, the expression of FPI encoded genes *iglJ*, *dotU* and *vgrG* in untreated Δ*relA*Δ*spoT* mutant did not alter significantly from that observed in untreated *F*. *tularensis* LVS (Fig 3A). The expression of all FPI genes including *iglJ*, *dotU*, and *vgrG* genes was significantly downregulated when the gene expression profile of the Δ*relA*Δ*spoT* mutant was compared with *F*. *tularensis* LVS upon exposure to H_2_O_2_ (Fig 3B)_._ We also confirmed the expression profiles of select FPI genes in the wild type *F*. *tularensis* LVS, the Δ*relA*, and the Δ*relA*Δ*spoT* mutants by quantitative real-time PCR (qRT-PCR). It was observed that deletion of the *relA* gene affected the expression of *iglA* as well as the *pdpA* gene. However, these genes were significantly downregulated in the Δ*relA*Δ*spoT* mutant as compared to the wild type *F*. *tularensis* LVS and the Δ*relA* mutant both in untreated, as well as H_2_O_2_ treated bacteria. Transcomplementation of the Δ*relA* mutant completely restored the expression levels of the FPI genes, while the expression of these genes was only partially restored in the Δ*relA*Δ*spoT* mutant transcomplemented with the *spoT* gene (Fig 3C and 3D). These results indicate that stringent response induced by RelA/SpoT positively regulates the expression of virulence-associated genes encoded on the FPI under normal as well as oxidative stress conditions.

### Expression of stress and heat shock proteins in Δ*relA*Δ*spoT* mutant of *F*. *tularensis*

**Fig 4:**
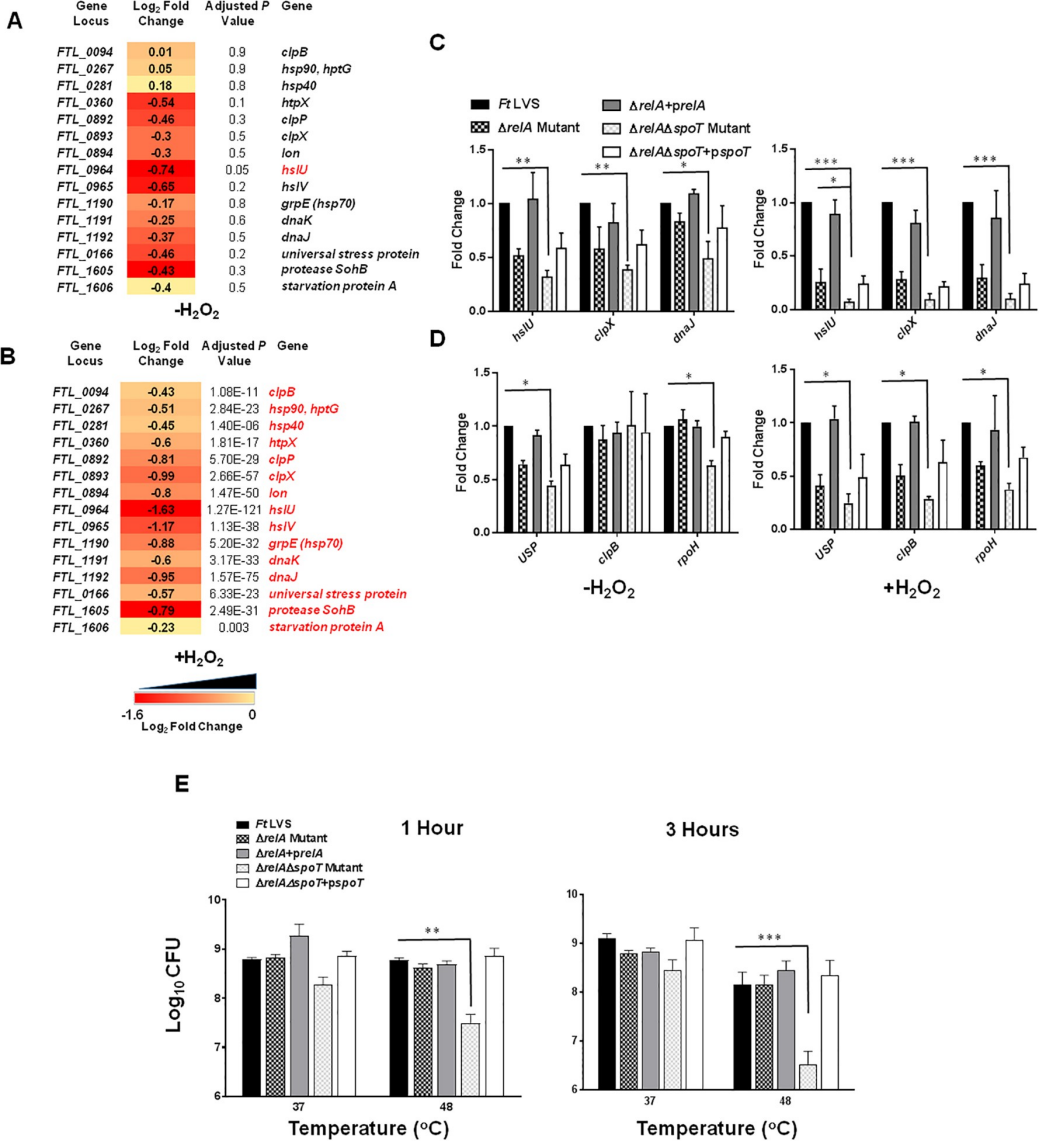
Expression of stress and heat shock proteins in Δ*relA*Δ*spoT* mutant of *F*. *tularensis*. RNAseq analysis comparing the differential expression of important stress response genes in untreated (A) and H_2_O_2_ treated (B) Δ*relA*Δ*spoT* mutant compared to that of wild type *Ft* LVS. The data are represented as Log_2_ fold change and are cumulative of three independent experiments. Genes in red fonts indicate significantly downregulated expression in the Δ*relA*Δ*spoT* mutant compared to that of wild type *Ft* LVS. A cutoff for multiple testing corrected *P*-value (adjusted *P*-value) of <0.05 was used to determine statistical significance and filter differentially expressed genes. (C and D) Quantitative reverse transcriptase PCR (qRT-PCR) was performed to evaluate the transcription of select genes. The amount of target gene amplification was normalized to a *tul4* internal control. The relative mRNA levels are presented as Mean±SD (n = 3 biological replicates). (E) A cell viability assay was performed by growing the indicated bacterial strains at 37 and 48°C for 1 and 3 hours. The cultures were diluted 10-fold and spotted on MH-chocolate agar plates. The data in C, D and E were analyzed by ANOVA, and the *P* values were recorded. *P<0.05; **P<0.01; ***P<0.001.

Induction of stress-related proteins is a hallmark of the RelA-SpoT-dependent stringent response. We studied the expression profiles of genes involved in degradation and disaggregation accumulated or misfolded proteins, and DNA damage repair mechanisms and heat shock in the Δ*relA*Δ*spoT* mutant in the absence or presence of oxidative stress and compared those with the wild type *F*. *tularensis* LVS. All genes examined except *clpB*, *hsp90* and *hsp40* were downregulated in untreated Δ*relA*Δ*spoT* mutant as compared to *F*. *tularensis* LVS. However, none of the up- or downregulated genes except the *hslU* gene achieved statistical significance (Fig 4A). On the contrary, all stress-related genes examined were found to be significantly downregulated in the Δ*relA*Δ*spoT* mutant as compared to the wild type *F*. *tularensis* LVS when exposed to the oxidative stress (Fig 4B). The differential expression of select genes was also confirmed by qRT-PCR. The expression profile of the representative stress response genes was similar to that observed by RNAseq. However, all these genes were significantly downregulated in untreated as well as H_2_O_2_ treated Δ*relA*Δ*spoT* mutant as compared to the *F*. *tularensis* LVS. Transcomplementation of the Δ*relA* mutant restored the wild type phenotype, while only a partial restoration of the gene expression was observed in the transcomplemented Δ*relA*Δ*spoT* mutant (Fig 4C and 4D).

Since RelA-SpoT-dependent induction of heat shock proteins is associated with survival at higher temperatures, we also examined the effect of the loss of *relA* and *relA/spoT* on bacterial viability when exposed to a higher temperature of 48°C. It was observed that the viability of the Δ*relA* mutant was unaffected and remained similar to the wild type *F*. *tularensis* when exposed to a temperature of 48°C for 1 hour and 5–7 fold fewer bacteria were recovered after 3 hours of exposure. On the other hand, nearly 10-100-fold less viable bacteria were recovered when the Δ*relA*Δ*spoT* mutant was exposed to a temperature of 48°C for 1 and 3 hours, respectively. Transcomplementation of the Δ*relA*Δ*spoT* mutant restored the wild type phenotype (Fig 4E). Collectively, these results indicate that RelA-SpoT-mediated stringent response induces protective mechanisms by altering the expression of genes involved in degradation and disaggregation accumulated or misfolded proteins, and DNA damage repair mechanisms under the conditions of oxidative stress. Also, the RelA-SpoT-mediated stringent response facilitates bacterial survival at higher temperatures.

### Expression of MglA-dependent genes and transcriptional regulators in Δ*relA*Δ*spoT* mutant of *F*. *tularensis*

**Fig 5:**
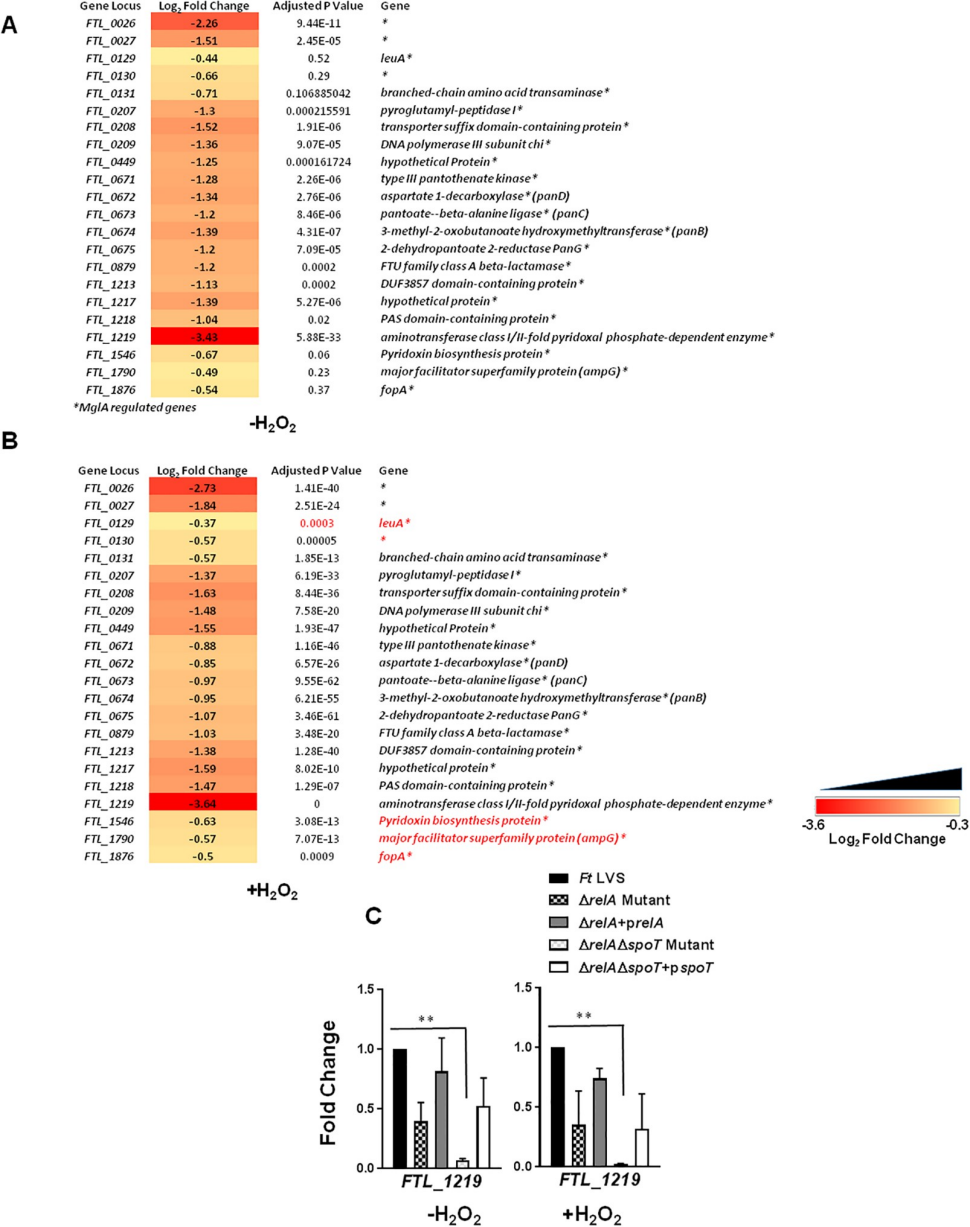
Expression of MglA-dependent genes in Δ*relA*Δ*spoT* mutant of *F*. *tularensis*. RNAseq analysis comparing the differential expression of MglA regulated genes in untreated (A) and H_2_O_2_ treated (B) Δ*relA*Δ*spoT* mutant compared to that of wild type *Ft* LVS. The data are represented as Log_2_ fold change and are cumulative of three independent experiments. Genes in red fonts indicate significantly downregulated expression in the Δ*relA*Δ*spoT* mutant compared to that of wild type *Ft* LVS. A cutoff for multiple testing corrected *P*-value (adjusted *P*-value) of <0.05 was used to determine statistical significance and filter differentially expressed genes. (C) qRT-PCR was performed to evaluate the transcription of a select gene. The amount of target gene amplification was normalized to a *tul4* internal control. The relative mRNA levels are presented as Mean±SD (n = 3 biological replicates). The data in C were analyzed by ANOVA, and the *P* values were recorded. **P<0.01.

MglA is a major transcriptional regulator of *F*. *tularensis* [24]. It has been reported the ppGpp produced by RelA and SpoT plays an important role in MglA-dependent gene regulation of *F*. *tularensis* [18]. We next investigated if the transcription of the MglA-dependent genes is altered in untreated or H_2_O_2_ treated Δ*relA*Δ*spoT* mutant. It was observed that all major MglA-regulated genes were significantly downregulated in untreated Δ*relA*Δ*spoT* mutant as compared to untreated *F*. *tularensis* LVS. However, the downregulation observed for *FTL_0129*, *FTL_0130*, *FTL_1546*, *FTL_1790*, and *FTL_1876* genes in the Δ*relA*Δ*spoT* mutant did not achieve statistical significance. Upon exposure to oxidative stress, all MglA regulated genes examined including *FTL_0129*, *FTL_0130*, *FTL_1546*, *FTL_1790*, and *FTL_1876* genes were significantly downregulated in the Δ*relA*Δ*spoT* mutant (Fig 5A and 5B). These results were also confirmed by qRT-PCR for the highly downregulated MglA-dependent gene *FTL_1219* (Fig 5C).

**Fig 6:**
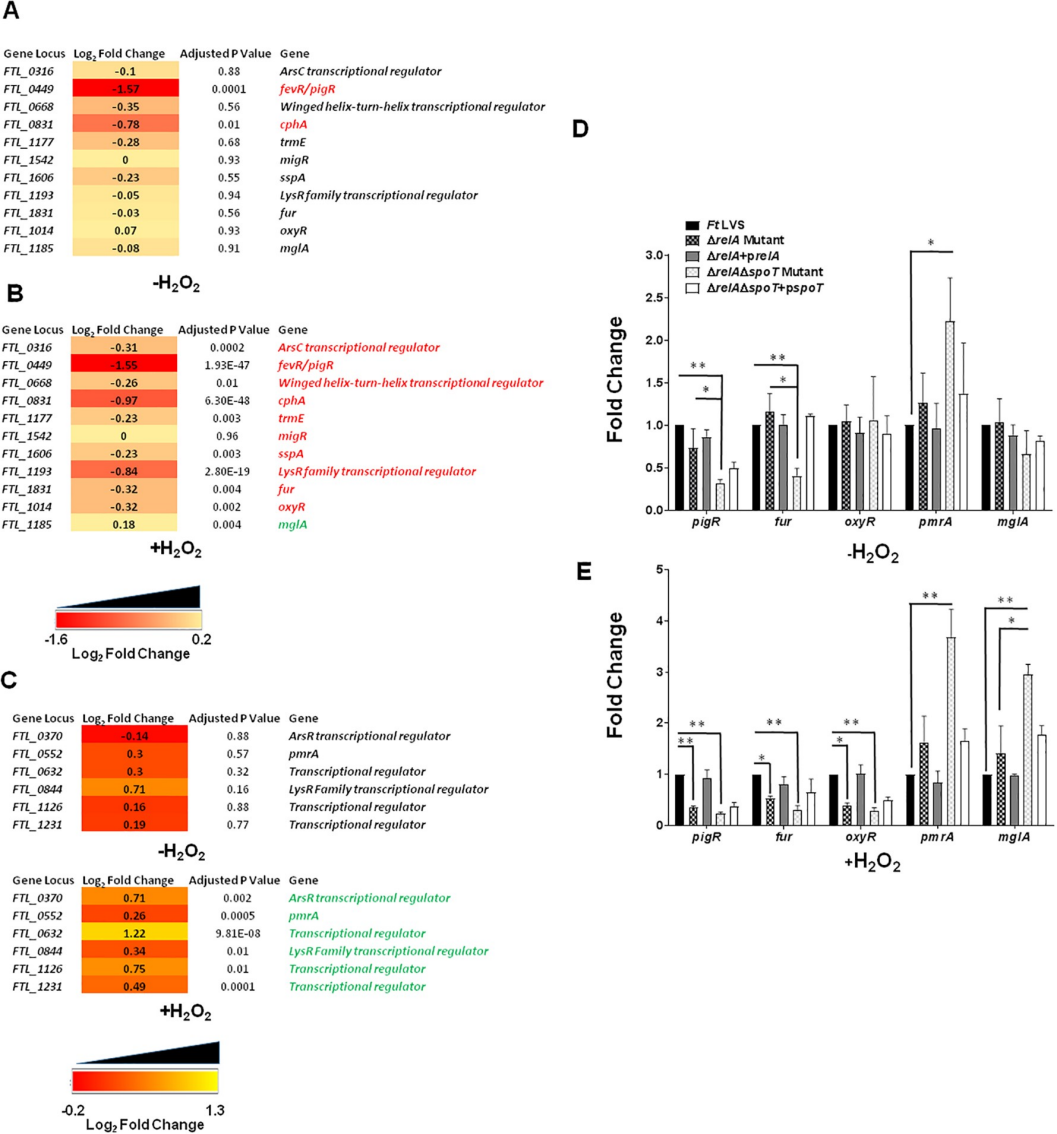
Expression of transcriptional regulators in Δ*relA*Δ*spoT* mutant of *F*. *tularensis*. (A, B and C) RNAseq analysis comparing the differential expression of transcriptional regulator genes in untreated and H_2_O_2_ treated Δ*relA*Δ*spoT* mutant compared to that of wild type *Ft* LVS. The data are represented as Log_2_ fold change and are cumulative of three independent experiments. Genes in red fonts indicate significantly downregulated expression in the Δ*relA*Δ*spoT* mutant compared to that of wild type *Ft* LVS. Genes in green fonts indicate significantly upregulated expression in the Δ*relA*Δ*spoT* mutant compared to that of wild type *Ft* LVS. A cutoff for multiple testing corrected *P*-value (adjusted *P*-value) of <0.05 was used to determine statistical significance and filter differentially expressed genes. (D, E) qRT-PCR was performed to evaluate the transcription of select genes. The amount of target gene amplification was normalized to a *tul4* internal control. The relative mRNA levels are presented as Mean±SD (n = 3 biological replicates). The data in D and E were analyzed by ANOVA, and the *P* values were recorded. *P<0.05; **P<0.01.

We also investigated if the loss of stringent response mediated by RelA/SpoT affects the expression of other transcription regulators in the absence or the presence of oxidative stress. It was observed that the expression of the majority of the transcriptional regulators was downregulated in untreated Δ*relA*Δ*spoT* mutant. However, only *fevR/pigR* and *cphA* achieved statistical significance (Fig 6A). On the other hand, expression of all the transcriptional regulator genes examined except *migR* was significantly downregulated in the Δ*relA*Δ*spoT* mutant as compared to the wild type *F*. *tularensis* LVS upon exposure to the oxidative stress (Fig 6B). Expression of several regulators including *pmrA*, *FTL_0632*, *FTL_ 1126*, and *mglA* remained unaltered in untreated Δ*relA*Δ*spoT* mutant (Fig 6A and 6C) however, was significantly upregulated in H_2_O_2_ treated Δ*relA*Δ*spoT* mutant (Fig 6B and 6C). These results were also confirmed by qRT-PCR (Fig 6D and 6E). Taken together, these results demonstrate that RelA-SpoT mediates the differential expression of several transcriptional regulators under normal growth as well as the conditions of oxidative stress.

### Expression of genes involved in antioxidant defense mechanisms in Δ*relA*Δ*spoT* mutant of *F*. *tularensis*

**Fig 7:**
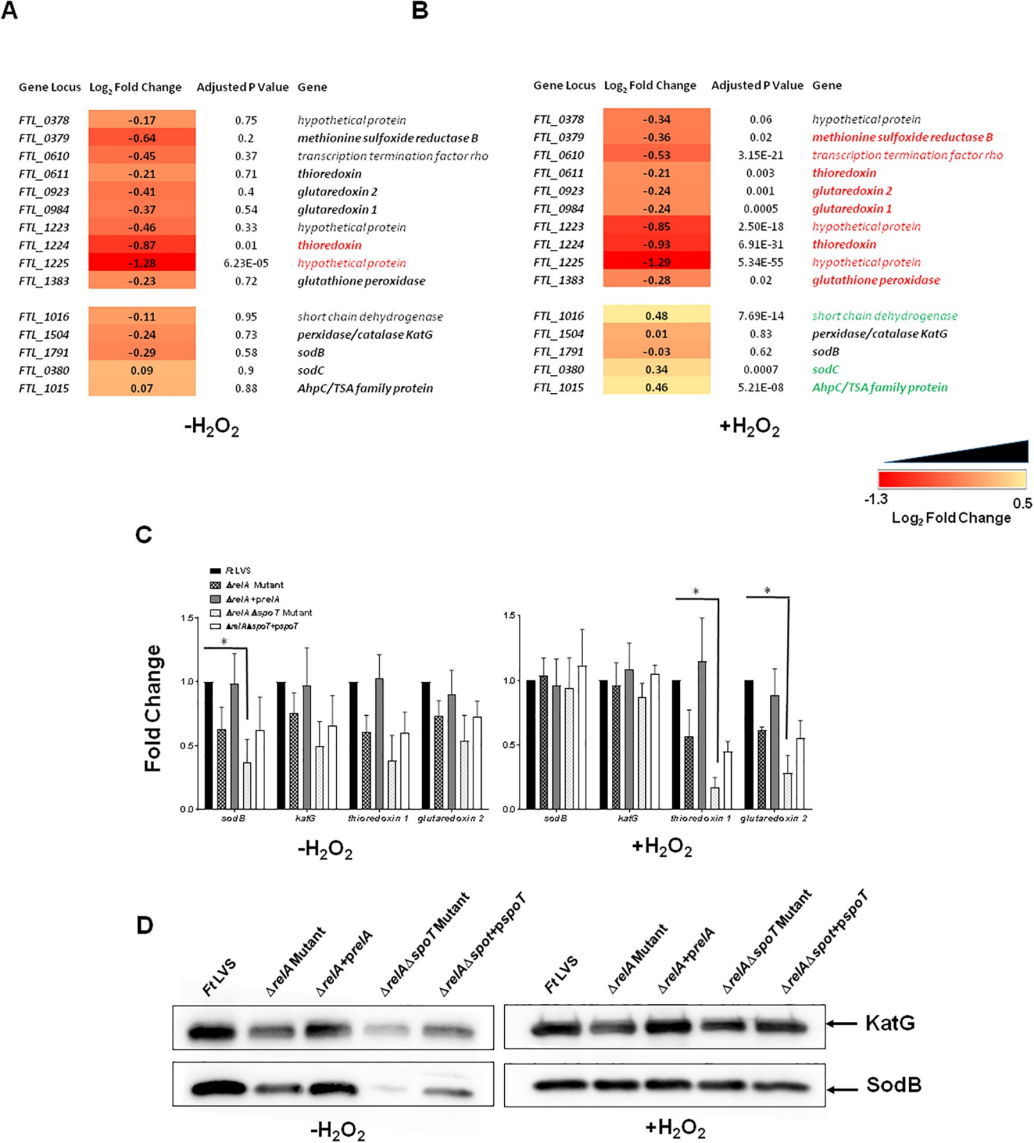
Expression of genes involved in antioxidant defense mechanisms in Δ*relA*Δ*spoT* mutant of *F*. *tularensis*. RNAseq analysis comparing the differential expression of antioxidant enzyme genes in untreated (A) and H_2_O_2_ (B) treated Δ*relA*Δ*spoT* mutant compared to that of wild type *Ft* LVS. The data are represented as Log_2_ fold change and are cumulative of three independent experiments. Genes in red fonts indicate significantly downregulated expression in the Δ*relA*Δ*spoT* mutant compared to that of wild type *Ft* LVS. Genes in green fonts indicate significantly upregulated expression in the Δ*relA*Δ*spoT* mutant compared to that of wild type *Ft* LVS. A cutoff for multiple testing corrected *P*-value (adjusted *P*-value) of <0.05 was used to determine statistical significance and filter differentially expressed genes. (C) qRT-PCR was performed to evaluate the transcription of select genes. The amount of target gene amplification was normalized to a *tul4* internal control. The relative mRNA levels are presented as Mean±SD (n = 3 biological replicates). The data were analyzed by ANOVA, and the *P* values were recorded. *P<0.05. (D) The western blots of the lysates of the indicated *Francisella* strains in the absence or presence of H_2_O_2_ probed with anti-KatG antibodies, that were stripped and re-probed with antibodies against SodB.

We next investigated the effect of loss of stringent response in the expression of genes encoding antioxidant enzymes of *F*. *tularensis*. Expression of *methionine sulfoxide reductase B* (*msrB*), *thioredoxin* (*FTL_0611*) *glutaredoxin 2 and 1*, *glutathione peroxidase*, *katG*, and *sodB* were downregulated in untreated Δ*relA*Δ*spoT* mutant. However, except *thioredoxin* and *hypothetical protein* genes (*FTL_1224* and *1225*) expression of none of the other antioxidant enzyme genes achieved statistical significance as compared to those observed for untreated *F*. *tularensis* LVS (Fig 7A). The expression of *methionine sulfoxide reductase B* (*msrB*), *thioredoxin* (*FTL_0611*) *glutaredoxin 2 and 1*, *glutathione peroxidase*, *FTL_1224*, and *FTL_1225* was significantly downregulated in the Δ*relA*Δ*spoT* mutant when exposed to oxidative stress. The expression of *katG and sodB* remained unaltered while those of *short chain dehydrogenase*, *sodC* and *ahpC* were significantly upregulated in the Δ*relA*Δ*spoT* mutant as compared to the *F*. *tularensis* LVS upon exposure to oxidative stress (Fig 7B).

The expression levels of *sodB*, *katG*, *thioredoxin 1*, and *glutaredoxin 2* were also confirmed by qRT-PCR, and the results similar to those obtained from the RNAseq were observed (Fig 7C). We also tested the protein levels of two primary antioxidant enzymes KatG and SodB in bacterial lysates from the wild type *F*. *tularensis* LVS, the Δ*relA*, Δ*relA*Δ*spoT* mutant and the transcomplemented strains by western blot analysis using anti-KatG and anti-SodB antibodies. Diminished protein levels of both KatG and SodB were observed in the Δ*relA* mutant and more so in the Δ*relA*Δ*spoT* mutant, and these levels were marginally improved in the transcomplemented strains (Fig 7D). However, the levels of both KatG and SodB in the Δ*relA* mutant and in the Δ*relA*Δ*spoT* mutant were similar to those observed for *F*. *tularensis* LVS when treated with H_2_O_2_, further confirming the results observed with RNA sequencing. Collectively, these results indicate that the loss of stringent response is associated with differential expression of genes involved in antioxidant defense mechanisms of *F*. *tularensis*.

### Δ*relA*Δ*spoT* mutant of *F*. *tularensis LVS* is sensitive to oxidative stress

**Fig 8:**
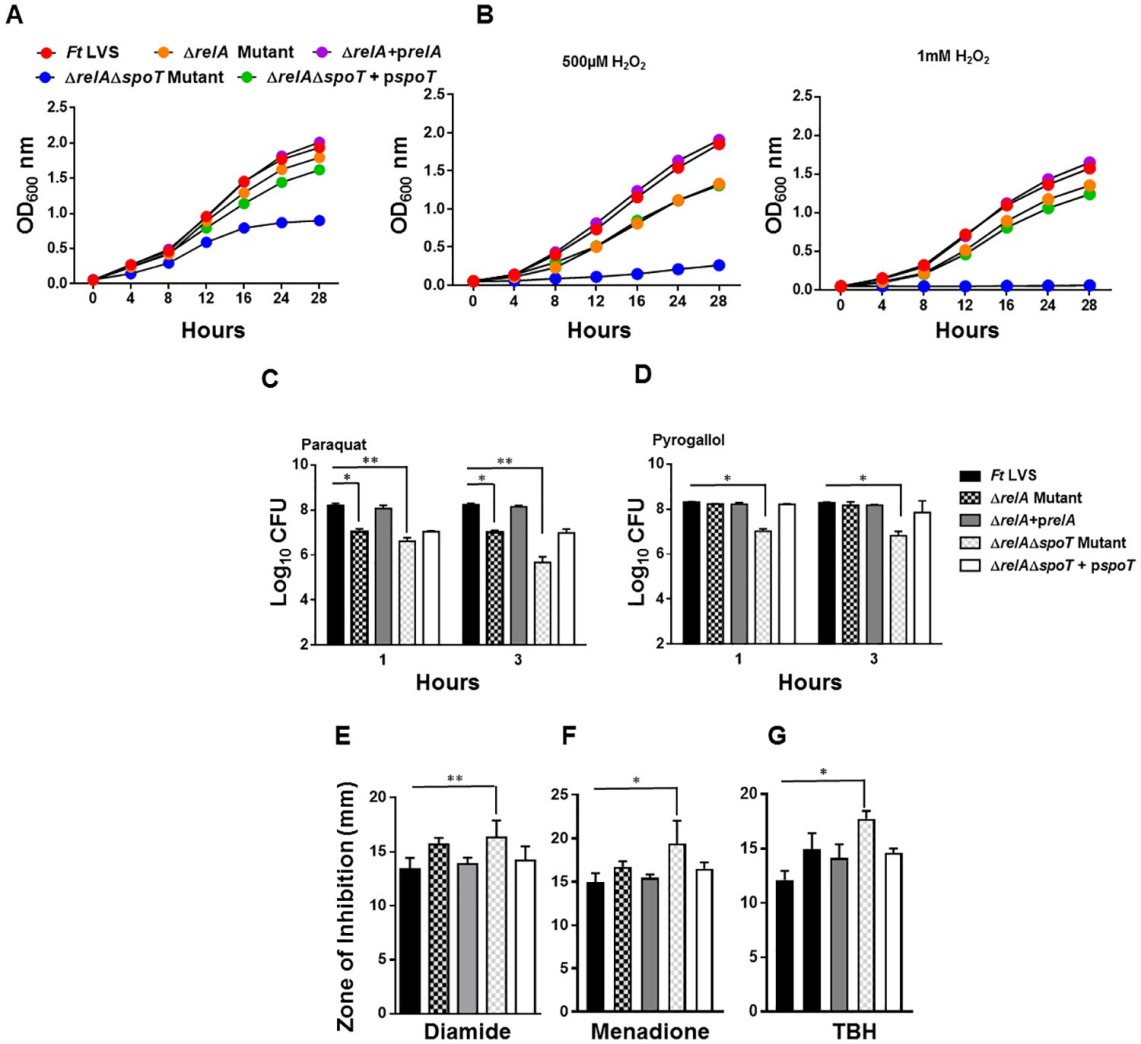
Δ*relA*Δ*spoT* mutant of *F*. *tularensis LVS* is sensitive to oxidative stress. *F*. *tularensis* (*Ft*) LVS, *the* Δ*relA and* Δ*relA*Δ*spoT mutants* and the transcomplemented strains were grown in (A) MH-broth or (B) MH-broth containing 500 μM and 1mM of H_2_O_2_. The cultures were grown for 28 hours, and OD_600_ readings were recorded every 4 hours. The bacterial cultures were exposed to (C) 1mM Paraquat or (D) 1mM Pyrogallol for 1 and 3 hours (n = 3 biological replicates). The cultures were diluted 10-fold and plated on MH-chocolate agar plates for bacterial enumeration. The results are expressed as Log_10_ CFU/ml. The data are representative of three independent experiments conducted with identical results. Disc diffusion assay with diamide (E) and superoxide-generating compounds, menadione (F) and organic peroxide tert-butyl hydroperoxide (TBH) (G). The results are expressed as a zone of inhibitions around the discs impregnated with compounds. The data in C, D, E, F, and G were analyzed by ANOVA, and the *P* values were recorded. *P<0.05; **P<0.01.

We next investigated the sensitivity of the Δ*relA*Δ*spoT* mutant by performing growth curve analysis, bacterial killing, and disc diffusion assays in the presence of oxidants. As observed earlier, the Δ*relA*Δ*spoT* mutant grew slowly and entered the stationary phase earlier than the wild type *F*. *tularensis* LVS or the Δ*relA* mutant. Transcomplementation of the Δ*relA*Δ*spoT* mutant restored its growth (Fig 8A). However, the Δ*relA*Δ*spoT* mutant failed to grow when the growth curves were generated in the presence of 0.5 and 1.0 mM H_2_O_2_ and again, transcomplementation of the Δ*relA*Δ*spoT* mutant restored its growth similar to that observed for the Δ*relA* mutant (Fig 8B). We confirmed these findings by performing bacterial killing assays by exposing wild type *F*. *tularensis*, the Δ*relA* and the Δ*relA*Δ*spoT* mutants and the transcomplemented strains to superoxide generating compounds paraquat and pyrogallol for 1 and 3 hours and counting the colonies for bacterial viability. The viability of both the Δ*relA* and the Δ*relA*Δ*spoT* mutant was similarly affected after 1 hour of treatment. However, the viability of the Δ*relA*Δ*spoT* mutant was further reduced after 3 hours of exposure. Nearly 100- and 10-fold less viable Δ*relA*Δ*spoT* mutant bacteria treated with paraquat and pyrogallol, respectively, were recovered as compared to those observed for the wild type *F*. *tularensis* LVS and the Δ*relA* mutant (Fig 8C and 8D). The Δ*relA*Δ*spoT* mutant also exhibited enhanced sensitivity as compared to the wild type *F*. *tularensis* LVS or the Δ*relA* mutant towards diamide and superoxide-generating compounds menadione and organic peroxide TBH when tested by disc diffusion assays (Fig 8E, 8F and 8G). Collectively, these results demonstrate that the loss of *relA* and *spoT* is associated with enhanced susceptibility of the Δ*relA*Δ*spoT* mutant towards oxidative stress. These results also indicate that the stringent response induced by the RelA and SpoT is also linked with the oxidative stress resistance of *F*. *tularensis*.

### Δ*relA*Δ*spoT* mutant of *F*. *tularensis* LVS is attenuated for intramacrophage growth and virulence in mice, and its replication is partially restored in *phox*^*-/-*^ BMDMs

**Fig 9:**
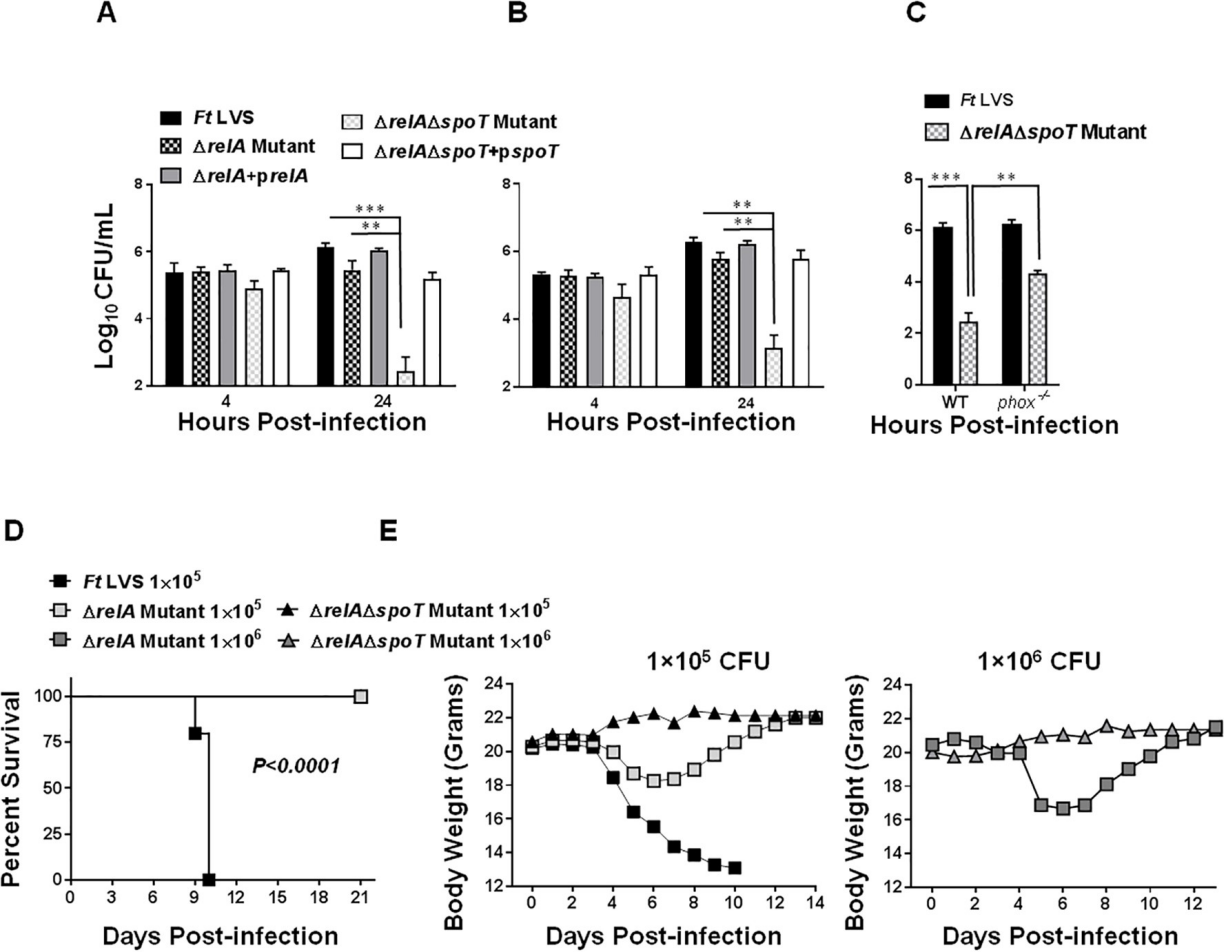
Δ*relA*Δ*spoT* mutant of *F*. *tularensis* LVS is attenuated for intramacrophage growth and virulence in mice, and its replication is partially restored in *phox*^*-/-*^ BMDMs. RAW macrophage cell line (A) and Bone Marrow-Derived Macrophages (BMDMs) (B) or the BMDMs derived from *phox*^*-/-*^ macrophages (C) were infected with wild type *F*. *tularensis* (*Ft*) LVS, the indicated mutants and the transcomplemented strains at a multiplicity of infection (MOI) of 100. The cells were lysed 4 and 24 hours post-infection, diluted 10-fold, and plated for enumeration of bacterial colonies. The data are represented as Mean±SD (n = 3 biological replicates) and are representative of 4 independent experiments. The data were analyzed using ANOVA and *P* values were determined. **P<0.01; ***P<0.001. (D and E) C57BL/6 mice (n = 5/group) were infected intranasally with 1x10^5^ CFUs of *F*. *tularensis* LVS and 1x10^5^ or 1x10^6^ CFUs of the Δ*relA* and the Δ*relA*Δ*spoT* mutants. A higher challenge dose (1x10^6^ CFUs) of *F*. *tularensis* LVS was not used and hence not shown. The mice were observed for mortality and morbidity for 21 days. The body weights of the infected mice were recorded daily to monitor the progress of infection. The data in D were analyzed using the Log-rank test.

We next performed cell culture assays using the Raw 264.7 cell line and BMDMs to investigate if the downregulated expression of the FPI genes is associated with attenuated intramacrophage survival. Except for the Δ*relA*Δ*spoT* mutant, equal numbers of bacteria were taken up by the infected Raw cells or BMDMs after 4 hours post-infection. Nearly 5-fold fewer bacteria were taken up by the macrophages infected with the Δ*relA*Δ*spoT* mutant at 4-hour time-point. At 24 hours post-infection, the Δ*relA* mutant was found to be only partially attenuated for intramacrophage growth. However, nearly 1000-fold less Δ*relA*Δ*spoT* mutant bacteria were recovered at 24 hours post-infection as compared to the wild type *F*. *tularensis* indicating an attenuation of the intramacrophage growth. Transcomplementation of both the Δ*relA* and Δ*relA*Δ*spoT* mutants restored the intramacrophage replication (Fig 9A and 9B).

Our preceding results demonstrated that the Δ*relA*Δ*spoT* mutant is sensitive to oxidants and attenuated for intramacrophage growth. We next investigated if the replication-deficient phenotype of the Δ*relA*Δ*spoT* mutant is restored by infecting *phox*^*-/-*^ macrophages which are incapable of generating reactive oxygen species. It was observed that nearly 100-fold higher Δ*relA*Δ*spoT* mutant bacteria were recovered from the *phox*^*-/-*^macrophages than those recovered from the wild type macrophages, indicating that the mutant bacteria can replicate in the absence of oxidative stress **(**Fig 9C**)**. These results demonstrate that the stringent response mediated by RelA/SpoT of *F*. *tularensis* is required to overcome the oxidative stress in macrophages to establish its intracellular replicative niche.

We also investigated if the loss of *relA* and *relA/spoT* is associated with the attenuation of virulence in mice. It was observed that 100% of C57BL/6 mice infected intranasally either with 1×10^5^ or 1×10^6^ CFUs of the Δ*relA* or the Δ*relA*Δ*spoT* mutant survived the infection. Mice infected with the Δ*relA* mutant exhibited initial loss of body weight from days 4–8 post-infection and then regained their initial body weight. On the other hand, mice infected with the Δ*relA*Δ*spoT* mutant did not show any bodyweight loss. All the control mice infected with 1×10^5^ CFU of *F*. *tularensis* LVS succumbed to infection by day ten post-infection and experienced severe body weight loss (Fig 8D and 8E). Collectively, these results demonstrate that RelA and SpoT mediated stringent response contributes to virulence in mice.

## Discussion

The synthesis of ppGpp by RelA in Gram-negative bacteria on the stalled ribosome diminishes the synthesis of stable RNA and ribosomal proteins, reprogram transcription machinery by inducing the expression of alternative sigma factors and enhance their association with core RNA polymerases. This response described as stringent response alters the half-life of RNA polymerase–promoter complexes to up- or downregulate the expression of several genes [16]. The SpoT is a bifunctional synthetase and hydrolase that regulates the intracellular concentrations of (p)ppGpp. The SpoT-dependent accumulation of ppGpp occurs under carbon, iron, and fatty acid starvation conditions [15]. The SpoT in-turn, is regulated by ribosome-associated proteases and acyl carrier proteins [25,26]. In *Francisella*, although not mechanistically demonstrated, it has been proposed that SpoT is regulated by *migR*, *trmE*, and *chpA* genes [19]. These genes, through unidentified mechanisms, are involved in SpoT-mediated ppGpp production, and regulation of FPI encoded virulence-associated genes. An additional protein mediates the promoter interaction that affects the gene transcription in *E*. *coli* is known as DksA [27]. The DksA homolog is absent in *F*. *tularensis*.

A very limited number of studies conducted to date on the stringent response of *Francisella* have primarily been focused on understanding the regulation of virulence gene expression encoded on the FPI. The Δ*relA* mutant of *F*. *novicida* fails to produce ppGpp under the conditions of amino acid starvation but grows better than the wild type strain at different stages of growth, including the stationary phase [17]. Moreover, the Δ*relA* mutant of *F*. *novicida* exhibits reduced viability at higher temperatures and attenuated virulence in mice. The expression of virulence genes requires the association of MglA and SspA proteins with RNA polymerase to form a complex which acts in conjunction with a DNA binding protein known as PigR. Additionally, RelA/SpoT-mediated production of ppGpp is required for stable interactions of PigR with MglA-SspA-RNA polymerase complex for the expression of virulence genes [18]. Similarly, MigR, TrmE, and CphA proteins of *F*. *tularensis* are required for accumulation ppGpp and virulence gene expression [19]. Transcriptome analysis of *F*. *tularensis* SchuS4 under starvation-like conditions induced artificially by exposing *Francisella* to serine hydroxamate has shown upregulation of FPI encoded genes involved in virulence, stress response, and metabolism and downregulation of genes involved in transport and cell division [20]. Collectively, this handful of studies have demonstrated the association of nutritional stress with virulence gene expression in *Francisella*.

Herein, we report a critical role of the stringent response in oxidative stress resistance of *F*. *tularensis*. The unique intracellular lifestyle of *Francisella* in addition to the nutritional stress also exposes bacteria to oxidative stress. However, how stringent response governs the oxidative stress response of *F*. *tularensis* is not known. To address this question, we adopted a genetic approach and generated a single gene deletion mutant Δ*relA* and a Δ*relA*Δ*spoT* double gene mutant. Unlike previous studies that mostly used amino acid starvation as a means to induce stringent response, in this study, we investigated the role of stringent response in a nutritionally rich environment and when the bacteria are exposed to oxidative stress. We observed that ppGpp production was reduced drastically in both the Δ*relA* and the Δ*relA*Δ*spoT* mutants as compared to wild type *F*. *tularensis* LVS even when the bacteria were still in the exponential phase of growth and not exposed to any stress, indicating that expression of ppGpp is also required under homeostatic growth conditions. Our initial characterization revealed that the Δ*relA*Δ*spoT* but not the Δ*relA* mutant has a growth defect, enter stationary phase early, and exhibit high resistance towards streptomycin, nitrofurantoin, and tetracycline. These phenotypic attributes, although have been reported for the Δ*relA*Δ*spoT* mutants of several other Gram-negative bacteria but have not been reported for the Δ*relA*Δ*spoT* mutant of *F*. *tularensis* LVS.

Our results show that a large number of genes are regulated by RelA/SpoT-dependent ppGpp even when bacteria are growing in a rich environment and not exposed to any nutritional stress. The majority of the genes that are regulated under the homeostatic growth conditions included those involved in metabolism, transport, and FPI genes. A number of genes encoding hypothetical proteins were also both up-and down-regulated in the Δ*relA*Δ*spoT* mutant. These results indicate that stringent response plays an essential role in the growth and survival of *F*. *tularensis* even in a nutritionally rich environment. When the bacteria were exposed to oxidative stress, twice the numbers of genes belonging to similar categories were differentially expressed in the Δ*relA*Δ*spoT* mutant indicating that stringent response regulates the oxidative stress response experienced by the bacteria in a nutritionally rich environment.

*Francisella* encounters oxidative stress in phagosomes as well as in the cytosol of the phagocytic cells during its intracellular residence. These results suggest that the expression of T6SS components encoded on FPI is regulated by stringent response under normal growth conditions as well as when the bacteria are exposed to oxidative stress. Specifically, the expression of genes that constitute the membrane complex of T6SS such as *pdpB*, *iglE* and *dotU* and the genes that encode T6SS effector proteins such as *pdpA*, *vgrG*, *iglG*, and *iglF* is regulated by the stringent response when the bacteria sense oxidative stress conditions. Coherent with the expression profile of the FPI genes, the Δ*relA*Δ*spoT* mutant but not the Δ*relA* was severely attenuated for intramacrophage growth. It was observed that both the Δ*relA* and the Δ*relA*Δ*spoT* mutants were highly attenuated for virulence upon intranasal infection in C57BL/6 mice. A previous study has reported the attenuation of Δ*relA*Δ*spoT* mutant of *F*. *tularensis* LVS in BALB/c mice upon intradermal challenge [18].

Similar to the FPI genes, the general stress proteins were also differentially expressed in the Δ*relA*Δ*spoT* mutant of *F*. *tularensis* LVS and three transcriptional profiles emerged when the bacteria were exposed to oxidative stress. The expression profile of *clpB*, *hsp90*, and *hsp40* genes involved in disaggregation and reactivation of the aggregated proteins, the stress response genes that serve as disaggregation or degradation machines and are transcribed as operons (*clpP*, *clpX*, and *lon*; *hslU*, *hslV; grpE*, *dnaK*, and *dnaJ*) and *usp*, *sohB and starvation protein A* genes involved in response to DNA damage were significantly downregulated when Δ*relA*Δ*spoT* mutant bacteria were exposed to oxidative stress. These results indicate that RelA/SpoT-dependent stringent response regulates the expression of the stress response genes in the presence of oxidative stress to facilitate bacterial survival. The differential expression of these stress response genes was also reflected in the enhanced sensitivity of the Δ*relA*Δ*spoT* mutant towards high temperature. Contrary to what has been reported for the Δ*relA* mutant of *F*. *novicida* [17], the viability of the Δ*relA* mutant of *F*. *tularensis* LVS is not affected by exposure to a higher temperature and remains similar to its wild type counterpart.

MglA is a master regulator of *F*. *tularensis* [24,28,29]. Several MglA-regulated genes were downregulated in the Δ*relA*Δ*spoT* mutant and remained similarly downregulated under oxidative stress conditions indicating that positive regulation of the MglA-dependent genes by RelA/SpoT is independent of the oxidative stress. In addition to MglA, in this study, it was also observed that a number of transcriptional regulators are also regulated both positively and negatively by RelA/SpoT. The positive regulation of the *fevR* and *cphA* genes is independent of the oxidative stress. *FevR* regulates the virulence gene expression of *F*. *tularensis* [30]; while *cphA* regulate FPI genes as well as the production of ppGpp [19]. On the other hand, a *LySR* family transcriptional regulator (*FTL_1193*), *fur* and *oxyR* are regulated positively by RelA/SpoT only under oxidative stress conditions. However, the transcription of *migR* and *mglA* genes is independent of RelA/SpoT-dependent stringent response. The expression of several transcriptional regulators, including *pmrA*, is negatively regulated by RelA/SpoT under oxidative stress conditions. Collectively, these results indicate that stringent response mediated by RelA/SpoT regulates expression of a number of transcriptional regulators under oxidative stress response and thus, serves as a master regulator to overcome oxidative stress.

Induction of stringent response is associated with increased transcription of oxidative stress response genes in Gram-positive bacterial pathogens such as *Enterococcus faecalis*, *Streptococci* and *Staphylococcus aureus* [31–34]. A similar association has also been demonstrated in Gram-negative pathogen *Pseudomonas aeruginosa*. It has been reported that stringent response mediated oxidative stress response protects *P*. *aeruginosa* against antibiotics exerting their bactericidal activities primarily by the induction of oxidative stress [35,36]. Recent reports have shown that ppGpp-mediated stringent response is required for the optimal catalase activity that provides H_2_O_2_ tolerance and protects from antibiotic-mediated killing of nutrient-starved *P*. *aeruginosa* during planktonic and biofilm growth [37,38]. These studies demonstrated a link between stringent and oxidative stress responses.

In the present study, it was observed that RelA/SpoT positively regulated a number of genes encoding antioxidant enzymes in *F*. *tularensis*. The most prominent ones being the *methionine sulfoxide reductase B* (*msrB*), *thioredoxin*, *glutaredoxin 1* and *3* under oxidative stress conditions. In *E*. *coli*, the *trx1* and *grx2* genes are positively regulated by ppGpp during the stationary phase of growth [39]. On the other hand, expression of primary antioxidant enzyme genes *katG* and *sodB* are not regulated by the stringent response. Moreover, the result indicates that expression of *sodC* and *ahpC* is negatively regulated by RelA/SpoT under the conditions of oxidative stress. Superoxide dismutase B (SodB) and catalase (KatG) activities are regulated by the stringent response in *Pseudomonas aeruginosa* [37,38]; however, the expression profiles of both KatG and SodB under oxidative stress conditions are not known. Similar to *P*. *aeruginosa*, we observed that in addition to the transcript levels, the protein levels of primary antioxidant enzymes SodB and KatG of *F*. *tularensis* were reduced in untreated Δ*relA* and Δ*relA*Δ*spoT* mutants. However, exposure to H_2_O_2_ restored the transcript as well as the protein levels of SodB and KatG, similar to those observed for the wild type *F*. *tularensis* LVS, indicating that under oxidative stress conditions, the expression of these primary antioxidant enzymes is not regulated by the stringent response. Despite the levels of primary antioxidant enzymes SodB, KatG, SodC, and AhpC remained unaltered or slightly elevated under oxidative stress, the Δ*relA*Δ*spoT* mutant exhibited enhanced sensitivity towards peroxides and superoxide-generating compounds. These observations indicate that thioredoxin and glutaredoxin systems, which are regulated by stringent response under oxidative stress conditions play an essential role in maintaining the redox-homeostasis and serves as an oxidative stress defense mechanism. Collectively, these observations indicate that stringent response of *F*. *tularensis* regulates the expression of antioxidant enzymes to prevent damage from oxidative stress.

To conclude, the unique intracellular lifestyle of *Francisella* in addition to the nutritional stress, also exposes bacteria to oxidative stress. This study demonstrates how stringent response governs the complex oxidative stress response of *F*. *tularensis* by regulating FPI genes, stress response genes, transcriptional regulators, and antioxidant defense mechanisms. This study provides evidence that in addition to nutritional stresses, the stringent response also plays an essential role in the oxidative stress resistance of *F*. *tularensis*.

## Supporting information

S1 Table Gene expression profile of untreated (-H_2_O_2_) Δ*relA*Δ*spoT* mutant as compared to the wild type *F*. *tularensis* LVS as determined by RNAseq analysis. (PDF)

S2 Table Gene expression profile of Δ*relA*Δ*spoT* mutant as compared to the wild type *F*. *tularensis* LVS following treatment with hydrogen peroxide (+H_2_O_2_) as determined by RNAseq analysis. (PDF)
