# Supplementary material for: Stringent response governs the oxidative stress resistance and virulence of Francisella tularensis
Source: PLoS One. 2019 Oct 24;14(10):e0224094. doi: 10.1371/journal.pone.0224094 (PMC6812791; doi:10.1371/journal.pone.0224094)
Supplement: S1 Table — (PDF) [file pone.0224094.s001.pdf]

S1 Table

Locus	Base Mean	Log_2 Fold-Change	lfcSE	Stat	P-value	P-Adjusted
FTL_0001	10288.01	-0.30305	0.264798	-1.14445	0.252438	0.618781
FTL_0002	7313.033	-0.51397	0.268966	-1.91089	0.056018	0.330196
FTL_0003	2458.877	-0.42376	0.233522	-1.81465	0.069578	0.378915
FTL_0004	1135.136	0.191905	0.259489	0.739552	0.459572	0.77919
FTL_0005	102.6272	0.725366	0.363299	1.996609	0.045868	0.294549
FTL_0006	403.3891	0.633272	0.313905	2.017398	0.043654	0.287687
FTL_0007	223.4013	0.01129	0.308095	0.036646	0.970768	0.994309
FTL_0008	1899.742	0.388548	0.237065	1.638995	0.101214	0.448176
FTL_0009	7142.729	-0.25269	0.278797	-0.90636	0.364746	0.712387
FTL_0010	3351.172	0.170535	0.250673	0.680308	0.496309	0.799344
FTL_0011	4606.351	-0.00684	0.248656	-0.0275	0.978058	0.994309
FTL_0012	11782.67	-0.08206	0.251271	-0.32656	0.743998	0.913853
FTL_0013	2337.749	0.07109	0.237814	0.298931	0.764993	0.920636
FTL_0014	2929.035	-0.4032	0.244502	-1.64906	0.099135	0.446841
FTL_0015	3574.754	0.297241	0.237076	1.253776	0.209923	0.587629
FTL_0016	3689.186	0.05715	0.233007	0.245271	0.806246	0.939549
FTL_0017	1138.627	-0.0585	0.248476	-0.23545	0.813863	0.939669
FTL_0018	1145.513	0.346645	0.263013	1.317978	0.187511	0.575235
FTL_0019	401.1857	-0.02188	0.283	-0.07733	0.938363	0.984914
FTL_0020	13278.45	-0.29527	0.240679	-1.22682	0.219889	0.593256
FTL_0021	117.9679	0.400082	0.349474	1.144814	0.252286	0.618781
FTL_0022	33.60423	0.54873	0.400887	1.368792	0.171064	0.563671
FTL_0023	1056.578	0.352846	0.259842	1.357926	0.174487	0.565576
FTL_0024	14694.5	-0.09898	0.240354	-0.4118	0.680488	0.884928
FTL_0025	16.36876	-0.21342	0.384182	-0.55551	0.578546	0.833908
FTL_0026	232.1184	-2.26722	0.313981	-7.22089	5.16E-13	9.44E-11
FTL_0027	359.0494	-1.51485	0.297083	-5.09907	3.41E-07	2.45E-05
FTL_0028	1151.787	-0.56331	0.27539	-2.04549	0.040807	0.274419
FTL_0029	5794.555	-0.64355	0.247008	-2.60537	0.009178	0.099256
FTL_0030	2748.758	-0.51999	0.246437	-2.11003	0.034856	0.250338
FTL_0031	512.8271	0.288865	0.292268	0.988355	0.322979	0.677157
FTL_0032	583.9096	0.412728	0.289768	1.42434	0.154348	0.544009
FTL_0033	3632.782	0.188078	0.23017	0.817128	0.413855	0.754738
FTL_0034	8351.464	0.247022	0.273171	0.904278	0.365848	0.713244
FTL_0035	45.30396	0.509949	0.395961	1.287876	0.197789	0.583892
FTL_0036	330.6475	-0.00894	0.313934	-0.02848	0.977281	0.994309
FTL_0037	1277.816	-0.17629	0.278096	-0.63391	0.52614	0.812401
FTL_0038	2737.473	0.166078	0.290439	0.571816	0.567447	0.830108
FTL_0039	3594.16	0.111647	0.301446	0.370373	0.711105	0.902349

FTL_0040	331.3294	-0.07165	0.292351	-0.24509	0.806389	0.939549
FTL_0041	161.1934	-0.01603	0.332547	-0.0482	0.961558	0.992291
FTL_0042	254.282	0.342139	0.330787	1.034317	0.300988	0.652888
FTL_0043	194.2414	0.17832	0.319537	0.558058	0.576805	0.833908
FTL_0044	921.2492	-0.09186	0.253831	-0.36191	0.717421	0.904215
FTL_0045	1928.583	-0.79771	0.259753	-3.07101	0.002133	0.03863
FTL_0046	2386.816	-0.59634	0.235606	-2.53107	0.011371	0.114885
FTL_0047	144.6489	0.26053	0.365558	0.71269	0.476037	0.788167
FTL_0048	667.5111	0.190913	0.290865	0.656364	0.51159	0.804396
FTL_0049	208.1281	0.421511	0.356023	1.183945	0.236435	0.609208
FTL_0050	108.7259	0.756628	0.369229	2.049209	0.040442	0.274419
FTL_0051	98.73949	-0.03536	0.362732	-0.09748	0.922348	0.977806
FTL_0052	62.76539	-0.12584	0.382242	-0.32922	0.74199	0.91279
FTL_0053	104.9927	0.263572	0.358869	0.734452	0.462673	0.77919
FTL_0054	870.7011	0.322251	0.259496	1.241836	0.214297	0.588439
FTL_0055	378.6663	0.495628	0.312645	1.585274	0.112904	0.471999
FTL_0056	426.1998	-0.38562	0.283148	-1.36192	0.173224	0.565576
FTL_0057	1556.978	-0.04217	0.243267	-0.17336	0.862365	0.956083
FTL_0058	2069.614	0.122435	0.243893	0.502	0.615667	0.855803
FTL_0059	416.8317	-0.20642	0.282145	-0.73162	0.464399	0.77919
FTL_0060	540.3536	-0.21294	0.273493	-0.7786	0.436215	0.771103
FTL_0061	634.6256	0.188255	0.315358	0.596958	0.550535	0.824572
FTL_0062	670.5559	0.386288	0.315838	1.223059	0.221308	0.593974
FTL_0063	286.212	0.186451	0.303357	0.614626	0.538802	0.822868
FTL_0064	43.40439	0.163424	0.397739	0.410883	0.681159	0.884928
FTL_0065	118.4492	0.279028	0.357343	0.780841	0.434896	0.771103
FTL_0066	33.17167	-0.16442	0.398362	-0.41273	0.679806	0.884928
FTL_0067	80.70766	-0.85103	0.377935	-2.25179	0.024336	0.200471
FTL_0068	1048.799	0.052834	0.247247	0.213691	0.830788	0.945344
FTL_0069	1567.504	-0.01213	0.24001	-0.05054	0.959692	0.992291
FTL_0070	4986.401	-0.41623	0.288168	-1.44441	0.148625	0.53615
FTL_0071	4247.928	-0.02438	0.231125	-0.10546	0.916008	0.97571
FTL_0072	3103.641	0.169403	0.241824	0.700521	0.483602	0.792208
FTL_0073	5366.324	-0.32216	0.246018	-1.30949	0.190369	0.575235
FTL_0074	899.3549	-0.30222	0.269493	-1.12142	0.262108	0.628129
FTL_0075	942.4028	-0.59225	0.277732	-2.13244	0.03297	0.243641
FTL_0076	1648.967	-1.00811	0.283575	-3.555	0.000378	0.011284
FTL_0077	574.7554	-0.54679	0.327351	-1.67034	0.094852	0.444411
FTL_0078	1096.955	-0.32246	0.321646	-1.00252	0.316094	0.668085
FTL_0079	319.7901	0.501642	0.331484	1.513322	0.130198	0.506185
FTL_0080	877.6224	0.326236	0.261082	1.249552	0.211463	0.587629
FTL_0081	135.1692	0.28714	0.336148	0.854209	0.392989	0.738706
FTL_0082	655.8892	-0.38988	0.261056	-1.49347	0.135315	0.513178

FTL_0083	157.8364	-0.15573	0.33316	-0.46743	0.640192	0.867785
FTL_0084	634.9284	-0.15365	0.27197	-0.56495	0.57211	0.832153
FTL_0085	1091.649	0.067018	0.29007	0.23104	0.817284	0.940321
FTL_0086	1365.329	0.342704	0.295567	1.15948	0.246261	0.617
FTL_0087	2086.578	-0.08398	0.240391	-0.34936	0.726818	0.907275
FTL_0088	3103.458	0.154082	0.242707	0.63485	0.525526	0.812401
FTL_0089	1163.433	-0.39571	0.269471	-1.46846	0.141979	0.523071
FTL_0090	533.6965	0.04101	0.269137	0.152377	0.87889	0.962183
FTL_0091	419.5443	0.00379	0.278797	0.013595	0.989153	0.996358
FTL_0092	1530.279	-0.2969	0.240611	-1.23395	0.217223	0.591625
FTL_0093	3906.732	0.250909	0.232056	1.081242	0.279589	0.641523
FTL_0094	24802.77	0.015007	0.262862	0.057092	0.954472	0.991603
FTL_0095	524.6233	0.093211	0.270276	0.344874	0.730189	0.90878
FTL_0096	695.7297	0.140127	0.271044	0.516989	0.605164	0.851082
FTL_0097	654.4295	-0.59595	0.302321	-1.97123	0.048697	0.307805
FTL_0098	2047.968	-0.68593	0.235734	-2.90977	0.003617	0.057699
FTL_0099	2029.596	-0.64417	0.236764	-2.72073	0.006514	0.08082
FTL_0100	713.901	-0.6821	0.26426	-2.58118	0.009846	0.103078
FTL_0101	857.2333	-0.07232	0.261147	-0.27692	0.781844	0.929687
FTL_0102	786.747	0.039194	0.269305	0.145539	0.884286	0.96389
FTL_0103	1540.101	0.043601	0.289541	0.150588	0.880301	0.962584
FTL_0104	6379.462	-0.07715	0.267494	-0.28842	0.773022	0.922668
FTL_0105	2229.187	-0.23014	0.281192	-0.81846	0.413096	0.754738
FTL_0106	84.5436	0.459558	0.38713	1.187089	0.235192	0.608818
FTL_0107	1025.871	0.302268	0.256515	1.178365	0.238651	0.609208
FTL_0108	135.8162	0.646607	0.375624	1.721422	0.085174	0.422501
FTL_0109	139.744	0.563355	0.352801	1.596806	0.110309	0.466602
FTL_0110	23.70616	-0.01026	0.398731	-0.02573	0.979469	0.994309
FTL_0111	1329.711	-3.32054	0.252467	-13.1524	1.65E-39	1.10E-36
FTL_0112	8386.004	-2.19196	0.237128	-9.24375	2.38E-20	5.98E-18
FTL_0113	5400.34	-1.64977	0.229297	-7.19489	6.25E-13	9.67E-11
FTL_0114	4025.139	-1.49529	0.238301	-6.2748	3.50E-10	4.69E-08
FTL_0115	775.4876	-1.20401	0.27094	-4.44382	8.84E-06	0.000444
FTL_0116	4441.558	-1.07054	0.268154	-3.99225	6.54E-05	0.00253
FTL_0117	546.3502	-0.808	0.312227	-2.58786	0.009657	0.101684
FTL_0118	1332.791	-1.03353	0.298449	-3.46299	0.000534	0.013766
FTL_0119	1095.822	-0.7711	0.318696	-2.41955	0.01554	0.141978
FTL_0120	1455.947	-1.45003	0.253558	-5.71873	1.07E-08	1.08E-06
FTL_0121	1059.159	-2.11588	0.266297	-7.94558	1.93E-15	3.88E-13
FTL_0122	588.4174	-1.0108	0.27835	-3.6314	0.000282	0.008853
FTL_0123	236.9564	-0.22197	0.351198	-0.63204	0.527364	0.812401
FTL_0124	283.8096	-1.11583	0.317637	-3.51292	0.000443	0.012203
FTL_0125	1953.657	-1.54704	0.262647	-5.89017	3.86E-09	4.31E-07

FTL_0126	2162.746	-2.41671	0.24154	-10.0055	1.44E-23	4.83E-21
FTL_0127	2468.295	-0.27621	0.24483	-1.12815	0.259255	0.6272
FTL_0128	1332.48	0.164228	0.251411	0.653225	0.513611	0.804396
FTL_0129	1709.712	-0.44667	0.30523	-1.46337	0.143366	0.523937
FTL_0130	665.4003	-0.66647	0.333475	-1.99857	0.045655	0.294122
FTL_0131	2191.516	-0.71287	0.27811	-2.56326	0.010369	0.106885
FTL_0132	4540.891	-0.04952	0.243959	-0.20298	0.839149	0.949983
FTL_0133	9704.659	0.245819	0.303447	0.810087	0.41789	0.758935
FTL_0134	145.0975	0.30242	0.351953	0.859261	0.390197	0.736428
FTL_0135	933.9047	0.339217	0.26481	1.280985	0.200199	0.584137
FTL_0136	487.3913	-0.09449	0.318207	-0.29694	0.766509	0.920898
FTL_0137	3178.485	0.369992	0.265611	1.392984	0.163625	0.55555
FTL_0138	2589.846	0.055335	0.233679	0.236801	0.812811	0.939669
FTL_0139	659.0523	-0.38591	0.260194	-1.48316	0.138031	0.517618
FTL_0140	653.6025	-0.31654	0.261359	-1.21112	0.225849	0.596555
FTL_0141	394.0302	-0.15995	0.28422	-0.56277	0.573593	0.832153
FTL_0142	357.0257	-0.15372	0.284554	-0.54023	0.589038	0.842174
FTL_0143	1918.687	0.991757	0.344501	2.878823	0.003992	0.061245
FTL_0144	3623.291	0.485038	0.277283	1.749254	0.080247	0.406289
FTL_0145	2605.94	0.294236	0.256971	1.145019	0.252201	0.618781
FTL_0146	4767.52	0.064587	0.251818	0.256482	0.797579	0.937924
FTL_0147	1097.984	-0.0339	0.277715	-0.12208	0.902837	0.970429
FTL_0148	3569.493	0.216453	0.230937	0.937282	0.348614	0.703126
FTL_0149	1863.177	-0.15485	0.275275	-0.56253	0.573755	0.832153
FTL_0150	1293.216	0.008513	0.25626	0.03322	0.973499	0.994309
FTL_0151	701.101	-0.25668	0.257846	-0.9955	0.319494	0.673149
FTL_0152	715.8203	0.352717	0.329872	1.069253	0.284956	0.643901
FTL_0153	1352.255	0.277567	0.259556	1.069392	0.284893	0.643901
FTL_0154	275.0253	0.230509	0.334233	0.689668	0.490403	0.793769
FTL_0155	469.3977	0.255009	0.326533	0.780961	0.434825	0.771103
FTL_0156	2009.577	-0.05238	0.242889	-0.21567	0.829248	0.945294
FTL_0157	1730.364	-0.13497	0.239218	-0.56422	0.572604	0.832153
FTL_0158	4324.134	-0.40757	0.259837	-1.56856	0.11675	0.477939
FTL_0159	1447.414	0.297819	0.293359	1.015201	0.31001	0.660785
FTL_0160	690.8944	-0.18315	0.265361	-0.69017	0.490085	0.793769
FTL_0161	1001.318	-0.2253	0.253109	-0.89013	0.373395	0.720965
FTL_0162	191.1737	0.356031	0.326847	1.089289	0.276026	0.638231
FTL_0163	73.35431	0.057147	0.377365	0.151438	0.87963	0.96247
FTL_0164	1001.413	0.281846	0.259508	1.08608	0.277444	0.638231
FTL_0165	436.6554	-0.4086	0.282355	-1.44712	0.147863	0.535503
FTL_0166	7375.022	-0.46546	0.234163	-1.98776	0.046838	0.29961
FTL_0167	655.9053	0.027163	0.265437	0.102335	0.918491	0.977325
FTL_0168	493.9412	0.16446	0.293072	0.561159	0.574689	0.832223

FTL_0169	408.3187	0.080867	0.279462	0.289365	0.772302	0.922357
FTL_0170	234.2117	-0.94728	0.349852	-2.70766	0.006776	0.083251
FTL_0171	502.6655	-0.15731	0.270719	-0.58106	0.561197	0.828816
FTL_0172	2641.636	0.344784	0.256116	1.346207	0.178236	0.569084
FTL_0173	1268.306	0.018993	0.25202	0.075363	0.939926	0.985525
FTL_0174	1300.371	0.122684	0.253656	0.483661	0.628627	0.861561
FTL_0175	962.7461	-0.00846	0.251849	-0.0336	0.9732	0.994309
FTL_0176	9956.774	0.153804	0.228368	0.67349	0.500636	0.800092
FTL_0177	2666.758	0.029401	0.239523	0.122746	0.902308	0.970429
FTL_0178	8915.669	-0.22938	0.230164	-0.99661	0.318952	0.672712
FTL_0179	2646.065	-0.22492	0.259806	-0.86574	0.386635	0.735977
FTL_0180	3401.3	0.144984	0.23756	0.610305	0.54166	0.824572
FTL_0181	978.8527	0.13469	0.257318	0.523439	0.600669	0.847854
FTL_0182	2180.397	-0.349	0.2378	-1.46764	0.142202	0.523071
FTL_0183	1676.218	-0.36392	0.256264	-1.42011	0.155575	0.544009
FTL_0184	596.3355	-0.11084	0.263108	-0.42127	0.673561	0.881992
FTL_0185	1170.957	0.292921	0.254558	1.150704	0.249854	0.618143
FTL_0186	617.0523	0.798698	0.295619	2.701781	0.006897	0.083511
FTL_0187	3708.971	0.354772	0.23845	1.487823	0.136798	0.514913
FTL_0188	8088.405	-0.43971	0.293292	-1.49924	0.133811	0.509395
FTL_0189	15719.88	-0.19534	0.252576	-0.77338	0.439299	0.773875
FTL_0190	1175.857	0.391573	0.267191	1.465518	0.14278	0.523071
FTL_0191	4704.688	-0.29498	0.232083	-1.27102	0.20372	0.585804
FTL_0192	14234.63	-0.35366	0.232312	-1.52235	0.127921	0.502191
FTL_0193	2980.847	-0.42077	0.257324	-1.63516	0.102015	0.448686
FTL_0194	1969.861	-0.41299	0.250273	-1.65016	0.09891	0.446841
FTL_0195	1316.499	-0.33211	0.252376	-1.31592	0.188202	0.575235
FTL_0196	5324.106	-0.25649	0.276163	-0.92875	0.353016	0.706735
FTL_0197	1579.21	0.226443	0.243109	0.931446	0.351623	0.705351
FTL_0198	3184.657	0.368386	0.245035	1.503399	0.132736	0.509395
FTL_0199	1557.095	0.055084	0.255402	0.215676	0.82924	0.945294
FTL_0200	2040.595	0.293614	0.274894	1.0681	0.285475	0.643901
FTL_0201	978.2393	0.026629	0.264729	0.100588	0.919878	0.977743
FTL_0202	242.9337	0.220964	0.313111	0.705706	0.480371	0.79199
FTL_0203	1199.716	0.357674	0.25491	1.40314	0.160575	0.550778
FTL_0204	1314.975	0.401917	0.273389	1.470133	0.141526	0.523071
FTL_0205	951.2528	0.238978	0.278954	0.856691	0.391615	0.73772
FTL_0206	2013.127	-0.04256	0.237302	-0.17937	0.85765	0.954771
FTL_0207	1064.426	-1.30995	0.282892	-4.63056	3.65E-06	0.000216
FTL_0208	628.4307	-1.52648	0.272	-5.61205	2E-08	1.91E-06
FTL_0209	407.8315	-1.36819	0.283547	-4.82526	1.40E-06	9.07E-05
FTL_0210	6889.728	-0.16821	0.235073	-0.71557	0.474256	0.787814
FTL_0211	1823.933	-0.07012	0.2385	-0.29401	0.768749	0.920898

FTL_0212	1618.268	0.127179	0.249441	0.509854	0.610154	0.851082
FTL_0213	667.3356	-0.28688	0.26751	-1.07239	0.283545	0.643901
FTL_0214	1581.316	-0.21712	0.251454	-0.86347	0.38788	0.735977
FTL_0215	926.0038	-0.63105	0.265318	-2.37846	0.017385	0.155558
FTL_0216	98.75216	-0.14925	0.356365	-0.41881	0.675356	0.883191
FTL_0217	1178.068	-0.35014	0.24935	-1.4042	0.160259	0.550633
FTL_0218	4314.271	0.025817	0.25516	0.101178	0.919409	0.977743
FTL_0219	1095.148	0.707926	0.28117	2.51779	0.011809	0.117509
FTL_0220	248.5959	0.301209	0.326692	0.921995	0.356531	0.708807
FTL_0221	1591.339	-0.91541	0.270082	-3.38936	0.000701	0.016928
FTL_0222	6759.702	0.09462	0.290991	0.325166	0.745056	0.913853
FTL_0223	1329.216	0.080619	0.247156	0.326185	0.744284	0.913853
FTL_0224	20683.65	-0.12761	0.22553	-0.5658	0.571528	0.832153
FTL_0225	9870.207	-0.37219	0.281855	-1.32049	0.186672	0.575235
FTL_0226	9658.995	-0.27498	0.263733	-1.04264	0.297113	0.646318
FTL_0227	2149.711	-0.61223	0.287519	-2.12935	0.033225	0.244625
FTL_0228	2730.341	0.226285	0.249493	0.906981	0.364417	0.712387
FTL_0229	891.9913	0.14848	0.264012	0.562398	0.573845	0.832153
FTL_0230	716.7174	0.038848	0.28039	0.138549	0.889807	0.967119
FTL_0231	876.6073	0.07056	0.278513	0.253346	0.8	0.937924
FTL_0232	10381.46	-0.29755	0.238479	-1.24769	0.212146	0.587629
FTL_0233	5055.868	-0.23458	0.228839	-1.02508	0.305324	0.656278
FTL_0234	37526.98	-0.41142	0.254214	-1.6184	0.105576	0.454038
FTL_0235	6261.554	-0.3968	0.239592	-1.65616	0.09769	0.446264
FTL_0236	21698.59	-0.41926	0.252152	-1.66274	0.096364	0.445547
FTL_0237	15656.27	-0.43461	0.254171	-1.70992	0.08728	0.426846
FTL_0238	7402.367	-0.39216	0.246003	-1.59413	0.110908	0.466602
FTL_0239	15316.78	-0.42611	0.259424	-1.64254	0.100478	0.448176
FTL_0240	11036.17	-0.09609	0.227308	-0.42272	0.672498	0.881992
FTL_0241	15705.11	-0.14722	0.225131	-0.65394	0.51315	0.804396
FTL_0242	17915.38	-0.31626	0.237271	-1.3329	0.182563	0.571974
FTL_0243	16614.91	-0.31865	0.242688	-1.313	0.189182	0.575235
FTL_0244	9243.894	0.010987	0.228706	0.048041	0.961683	0.992291
FTL_0245	4574.611	-0.2896	0.237601	-1.21884	0.222906	0.595007
FTL_0246	11244.34	-0.15934	0.24292	-0.65594	0.511865	0.804396
FTL_0247	16139.23	0.000298	0.22452	0.001326	0.998942	0.99895
FTL_0248	21218.74	-0.43605	0.297393	-1.46625	0.142581	0.523071
FTL_0249	17719.16	-0.33062	0.262894	-1.25762	0.20853	0.586556
FTL_0250	20471.98	-0.19593	0.240704	-0.81398	0.415659	0.75672
FTL_0251	16713.25	-0.34574	0.254459	-1.35871	0.174238	0.565576
FTL_0252	12621.35	-0.32381	0.244118	-1.32644	0.184695	0.574669
FTL_0253	25309.18	0.048336	0.226025	0.213852	0.830663	0.945344
FTL_0254	17324.19	0.176287	0.239478	0.73613	0.461651	0.77919

FTL_0255	11181.8	-0.01102	0.226033	-0.04877	0.961106	0.992291
FTL_0256	24615.52	-0.44121	0.281046	-1.56988	0.116444	0.477814
FTL_0257	1699.396	-0.44849	0.296205	-1.51413	0.129993	0.506185
FTL_0258	10522.76	-0.33509	0.27474	-1.21965	0.222596	0.594971
FTL_0259	11293.6	-0.34154	0.271132	-1.25969	0.207781	0.586556
FTL_0260	14132.67	-0.41836	0.264828	-1.57973	0.114168	0.474128
FTL_0261	18648.18	-0.40681	0.278498	-1.46071	0.144095	0.525645
FTL_0262	9994.989	-0.42885	0.264602	-1.62073	0.105075	0.453221
FTL_0263	1950.177	0.056972	0.243345	0.234121	0.814891	0.939669
FTL_0264	429.7891	0.30546	0.288725	1.057961	0.290073	0.643901
FTL_0265	4929.121	-0.65286	0.235219	-2.77554	0.005511	0.072876
FTL_0266	1057.818	0.171686	0.259239	0.662271	0.507798	0.804313
FTL_0267	36441.95	0.050961	0.285021	0.178799	0.858096	0.954771
FTL_0268	1508.375	0.327319	0.243617	1.34358	0.179084	0.569084
FTL_0269	17799.6	-0.13964	0.24154	-0.57813	0.563177	0.829294
FTL_0270	182.8262	0.427681	0.331717	1.289293	0.197296	0.583892
FTL_0271	304.0252	0.269068	0.291474	0.923129	0.35594	0.708356
FTL_0272	402.1099	0.725061	0.293662	2.469034	0.013548	0.130293
FTL_0273	664.4107	0.226065	0.260544	0.867667	0.385577	0.735977
FTL_0274	274.4096	0.014788	0.297776	0.049661	0.960393	0.992291
FTL_0275	240.2185	0.242083	0.309617	0.781878	0.434286	0.771103
FTL_0276	161.8585	-0.39798	0.327886	-1.21378	0.224831	0.596453
FTL_0277	680.7462	-0.38557	0.266829	-1.44502	0.148453	0.53615
FTL_0278	253.1173	-0.17511	0.301105	-0.58156	0.560866	0.828816
FTL_0279	446.436	-0.15934	0.282141	-0.56477	0.572231	0.832153
FTL_0280	605.5578	-0.51196	0.275497	-1.8583	0.063127	0.35646
FTL_0281	1684.584	0.188405	0.299514	0.629035	0.529326	0.812401
FTL_0282	2218.001	0.368424	0.297	1.240485	0.214796	0.588548
FTL_0283	912.8784	0.884619	0.307924	2.872848	0.004068	0.061477
FTL_0284	472.0097	0.123699	0.282078	0.438529	0.661003	0.879296
FTL_0285	11465.59	-0.61681	0.250869	-2.45868	0.013945	0.132214
FTL_0286	915.9623	0.579012	0.288384	2.007781	0.044667	0.291493
FTL_0287	353.4478	0.590457	0.31775	1.858245	0.063134	0.35646
FTL_0288	255.2739	0.293822	0.313807	0.936314	0.349112	0.703126
FTL_0289	970.3251	0.442916	0.280371	1.57975	0.114164	0.474128
FTL_0290	1201.606	-0.20928	0.250149	-0.83662	0.402808	0.748883
FTL_0291	1215.141	-0.06093	0.243685	-0.25004	0.802553	0.937924
FTL_0292	555.7603	0.656979	0.304435	2.158029	0.030926	0.235785
FTL_0293	4444.372	-0.02106	0.255601	-0.08241	0.934323	0.982726
FTL_0294	3380.034	0.213631	0.235201	0.908289	0.363726	0.712387
FTL_0295	9645.91	-0.11256	0.22567	-0.49876	0.617946	0.856601
FTL_0296	2418.102	-0.07664	0.238061	-0.32195	0.74749	0.914459
FTL_0297	1874.075	0.316567	0.264185	1.19828	0.230808	0.604483

FTL_0298	1216.937	0.084633	0.262025	0.322996	0.746698	0.914459
FTL_0299	999.1473	0.126805	0.262642	0.482804	0.629235	0.861561
FTL_0300	685.6188	-0.19604	0.265894	-0.73728	0.460954	0.77919
FTL_0301	149.8104	0.395527	0.349562	1.131492	0.257848	0.625181
FTL_0302	172.902	0.471043	0.34285	1.373905	0.169471	0.563239
FTL_0303	552.2544	0.875842	0.368843	2.374568	0.01757	0.15626
FTL_0304	2091.747	0.112863	0.237295	0.475624	0.634342	0.865012
FTL_0305	1900.734	-0.13071	0.251072	-0.5206	0.602648	0.848928
FTL_0306	6275.537	0.26361	0.250458	1.05251	0.292566	0.643901
FTL_0307	6034.745	-0.26102	0.246681	-1.05814	0.289993	0.643901
FTL_0308	689.4745	0.26194	0.257968	1.015396	0.309917	0.660785
FTL_0309	20233.85	0.010374	0.236613	0.043843	0.96503	0.994309
FTL_0310	8828.68	-0.18674	0.259685	-0.7191	0.47208	0.786405
FTL_0311	13681.54	-0.09895	0.253909	-0.38971	0.696753	0.896555
FTL_0312	1648.996	-0.14902	0.288395	-0.51672	0.605355	0.851082
FTL_0313	239.5323	0.072086	0.302627	0.238199	0.811726	0.939669
FTL_0314	1051.258	0.298277	0.255306	1.168311	0.242681	0.612533
FTL_0315	901.4674	-0.33044	0.250841	-1.31732	0.187731	0.575235
FTL_0316	3074.253	-0.10517	0.242387	-0.43391	0.664353	0.881666
FTL_0317	6625.283	-0.86869	0.253924	-3.42106	0.000624	0.015672
FTL_0318	712.7872	0.550533	0.26826	2.052239	0.040146	0.27354
FTL_0319	2087.742	0.203933	0.244225	0.835024	0.403704	0.748883
FTL_0320	448.6589	0.165083	0.275358	0.599522	0.548825	0.824572
FTL_0321	20.44091	0.416468	0.394712	1.055117	0.291372	0.643901
FTL_0322	231.5117	0.097723	0.30884	0.316418	0.751685	0.918813
FTL_0323	189.885	0.15971	0.316379	0.504804	0.613696	0.854837
FTL_0324	730.5645	-0.33436	0.262122	-1.27559	0.202101	0.58465
FTL_0325	6500.356	-0.38165	0.243828	-1.56523	0.117529	0.478205
FTL_0326	1558.821	0.087695	0.253061	0.346538	0.728938	0.90788
FTL_0327	7348.28	0.46379	0.231413	2.004161	0.045053	0.292117
FTL_0328	1330.5	0.22298	0.244495	0.912	0.361769	0.712199
FTL_0329	2103.271	-0.00389	0.244736	-0.01589	0.987323	0.996244
FTL_0330	656.5101	-0.07165	0.265006	-0.27038	0.786868	0.931452
FTL_0331	3216.538	0.140472	0.260361	0.539529	0.589522	0.842174
FTL_0332	1724.208	-0.1026	0.257036	-0.39916	0.689778	0.892184
FTL_0333	1818.489	-0.05349	0.245591	-0.21781	0.827578	0.945294
FTL_0334	2660.27	-0.09428	0.245079	-0.38469	0.700465	0.897755
FTL_0335	588.8383	-0.53356	0.3014	-1.77027	0.076683	0.395437
FTL_0336	2584.826	-0.6913	0.260493	-2.65381	0.007959	0.093007
FTL_0337	9631.535	-0.00657	0.247627	-0.02653	0.978835	0.994309
FTL_0338	882.3327	0.119626	0.277247	0.431479	0.66612	0.881666
FTL_0339	1181.31	0.365097	0.255466	1.429143	0.152963	0.543488
FTL_0340	228.7099	0.831404	0.35875	2.317502	0.020476	0.175887

FTL_0341	241.9001	0.551418	0.339686	1.623316	0.104522	0.451804
FTL_0342	100.5912	0.477722	0.365443	1.307241	0.191131	0.575235
FTL_0343	197.6057	0.644814	0.330414	1.951537	0.050993	0.319864
FTL_0344	43.75337	0.177372	0.395514	0.44846	0.653821	0.875536
FTL_0345	740.9276	-0.25434	0.290547	-0.8754	0.381357	0.730723
FTL_0346	364.3489	0.182214	0.288822	0.630889	0.528113	0.812401
FTL_0347	333.3715	0.079536	0.285422	0.278663	0.780503	0.929092
FTL_0348	227.5487	-1.03355	0.349129	-2.96038	0.003073	0.051466
FTL_0349	110.7092	-0.47659	0.376658	-1.26532	0.205757	0.586521
FTL_0350	205.0022	0.489765	0.367577	1.332412	0.182725	0.571974
FTL_0351	294.1516	0.612558	0.34396	1.780898	0.074929	0.390561
FTL_0352	94.88211	0.411578	0.369326	1.114403	0.265106	0.630978
FTL_0353	954.3198	0.305542	0.256427	1.191535	0.233443	0.6078
FTL_0354	435.9727	-0.44225	0.276856	-1.59741	0.110175	0.466602
FTL_0355	639.1771	-0.28256	0.269221	-1.04954	0.293928	0.643956
FTL_0356	825.6493	-0.16528	0.255511	-0.64686	0.517719	0.805788
FTL_0357	3159.19	0.200832	0.266291	0.754183	0.450739	0.778339
FTL_0358	2189.759	0.286321	0.239187	1.19706	0.231283	0.604483
FTL_0359	2405.194	0.430699	0.237999	1.809671	0.070347	0.378915
FTL_0360	3023.517	-0.54332	0.238737	-2.27579	0.022859	0.191932
FTL_0361	1684.372	-0.40114	0.256121	-1.5662	0.117301	0.478205
FTL_0362	380.6231	0.30748	0.292183	1.052353	0.292638	0.643901
FTL_0363	476.592	-0.09515	0.272207	-0.34955	0.72668	0.907275
FTL_0364	223.243	0.447444	0.32235	1.388071	0.165115	0.556849
FTL_0365	431.5606	0.493659	0.29065	1.698466	0.08942	0.430505
FTL_0366	206.8368	0.306341	0.338208	0.905777	0.365054	0.712387
FTL_0367	218.6367	0.406823	0.319825	1.272018	0.203367	0.585626
FTL_0368	641.4447	0.482582	0.316696	1.5238	0.127559	0.501748
FTL_0369	219.6777	-0.37708	0.310857	-1.21303	0.225118	0.596453
FTL_0370	132.3894	-0.14116	0.344476	-0.40978	0.681967	0.884928
FTL_0371	632.8443	1.144961	0.322308	3.55238	0.000382	0.011284
FTL_0372	1939.893	-0.12033	0.239218	-0.503	0.614965	0.855419
FTL_0373	837.0992	-0.10788	0.253699	-0.42521	0.670681	0.881666
FTL_0374	1872.516	-0.09273	0.243782	-0.3804	0.703647	0.899129
FTL_0375	2831.582	0.149229	0.242335	0.615799	0.538027	0.822384
FTL_0376	1435.827	0.048139	0.255998	0.188043	0.850843	0.952754
FTL_0377	3217.67	-0.20993	0.256984	-0.81688	0.413995	0.754738
FTL_0378	370.7621	-0.1745	0.281932	-0.61894	0.535956	0.820906
FTL_0379	346.1759	-0.6472	0.288163	-2.24594	0.024708	0.202707
FTL_0380	1629.528	0.090255	0.253826	0.355577	0.722158	0.905632
FTL_0381	4529.767	0.853496	0.242211	3.523766	0.000425	0.012203
FTL_0382	17961.43	0.490499	0.26968	1.818819	0.068939	0.378915
FTL_0383	1008.928	-0.14177	0.247443	-0.57294	0.566684	0.829991

FTL_0384	596.241	-0.46259	0.264027	-1.75205	0.079766	0.406289
FTL_0385	732.9382	0.036671	0.287599	0.127509	0.898537	0.969455
FTL_0386	1626.415	0.298561	0.277728	1.075011	0.28237	0.643901
FTL_0387	9578.527	-0.70934	0.270126	-2.62595	0.008641	0.097574
FTL_0388	238.2827	-0.32606	0.302746	-1.07702	0.281471	0.643901
FTL_0389	2433.006	0.220595	0.243045	0.907632	0.364073	0.712387
FTL_0390	2079.96	0.583496	0.256986	2.270537	0.023175	0.193286
FTL_0391	850.7782	0.09514	0.281732	0.337695	0.735593	0.911008
FTL_0392	14206.07	-0.19952	0.235766	-0.84626	0.397409	0.743754
FTL_0393	1093.537	0.015801	0.252793	0.062504	0.950161	0.990573
FTL_0394	4640.824	0.107927	0.238958	0.451657	0.651516	0.874878
FTL_0395	4323.376	-0.54404	0.245056	-2.22008	0.026413	0.214074
FTL_0396	9288.366	-0.57396	0.286117	-2.00602	0.044854	0.291772
FTL_0397	1068.106	-0.6213	0.318832	-1.94867	0.051335	0.319864
FTL_0398	936.2679	-0.58809	0.272114	-2.16118	0.030682	0.235382
FTL_0399	2394.8	-0.7459	0.266648	-2.79731	0.005153	0.071328
FTL_0400	309.5914	0.211686	0.315062	0.671886	0.501656	0.800092
FTL_0401	705.3647	0.329236	0.260914	1.261853	0.207002	0.586521
FTL_0402	1843.592	0.082976	0.266565	0.311276	0.75559	0.919801
FTL_0403	1235.863	0.258209	0.261285	0.988226	0.323042	0.677157
FTL_0404	2852.025	0.224181	0.251537	0.891243	0.372799	0.720505
FTL_0405	1708.256	-0.00082	0.248486	-0.00332	0.997353	0.998347
FTL_0406	1361.933	-0.04353	0.24627	-0.17676	0.859697	0.954771
FTL_0407	2842.312	-0.0363	0.243465	-0.14911	0.881463	0.962584
FTL_0408	764.6457	-0.02476	0.26711	-0.09268	0.926159	0.980295
FTL_0409	597.7465	0.429341	0.312779	1.372667	0.169856	0.563239
FTL_0410	2073.438	0.296185	0.243522	1.216256	0.223888	0.596045
FTL_0411	3122.846	0.036449	0.236345	0.154221	0.877436	0.961115
FTL_0412	1692.217	0.178915	0.250766	0.713477	0.475551	0.788011
FTL_0413	2162.787	-0.2415	0.261033	-0.92516	0.354883	0.707654
FTL_0414	3476.232	-0.18934	0.257326	-0.73581	0.461848	0.77919
FTL_0415	401.5674	0.970751	0.333957	2.906818	0.003651	0.057788
FTL_0416	266.678	0.484646	0.31355	1.545673	0.122184	0.488249
FTL_0417	934.3111	0.267923	0.255709	1.047767	0.294746	0.643956
FTL_0418	282.3714	-0.3513	0.295912	-1.18717	0.235161	0.608818
FTL_0419	2902.561	-0.27234	0.236908	-1.14954	0.250332	0.618143
FTL_0420	638.8331	-0.34677	0.266669	-1.30038	0.19347	0.578684
FTL_0421	12631.05	0.138555	0.232058	0.597072	0.550459	0.824572
FTL_0422	2417.674	0.274499	0.252782	1.085909	0.277519	0.638231
FTL_0423	2344.059	0.089654	0.234456	0.382392	0.70217	0.897813
FTL_0424	4459.603	0.046256	0.229697	0.20138	0.840401	0.950146
FTL_0425	3944.388	0.11746	0.243075	0.483226	0.628935	0.861561
FTL_0426	7928.145	0.089923	0.26565	0.338504	0.734984	0.911008

FTL_0427	3278.543	-0.11129	0.245765	-0.45284	0.650665	0.874878
FTL_0428	2987.969	0.15328	0.232667	0.658795	0.510027	0.804396
FTL_0429	3077.579	0.349736	0.251023	1.393243	0.163546	0.55555
FTL_0430	2718.139	0.087437	0.246204	0.355142	0.722483	0.905632
FTL_0431	395.3481	-0.29225	0.295359	-0.98946	0.322438	0.677157
FTL_0432	243.9319	0.10707	0.303614	0.352652	0.724349	0.907129
FTL_0433	860.7814	-0.03782	0.262793	-0.14392	0.88556	0.964756
FTL_0434	1048.045	-0.18169	0.247478	-0.73415	0.462857	0.77919
FTL_0435	3149.047	-0.00703	0.236571	-0.02971	0.976301	0.994309
FTL_0436	17444.63	-0.26087	0.254307	-1.02579	0.304992	0.656278
FTL_0437	3247.646	-0.09008	0.248888	-0.36194	0.717394	0.904215
FTL_0438	20643.03	0.172507	0.227794	0.757294	0.448874	0.77779
FTL_0439	20870.19	-0.05751	0.25929	-0.22181	0.82446	0.945294
FTL_0440	1719.092	0.326651	0.243829	1.339673	0.180352	0.569084
FTL_0441	387.5463	0.788388	0.297871	2.64674	0.008127	0.094044
FTL_0442	618.5958	1.141041	0.279794	4.078154	4.54E-05	0.001984
FTL_0443	3625.422	0.548865	0.255304	2.149851	0.031567	0.237581
FTL_0444	15802.5	0.857256	0.246115	3.48315	0.000496	0.013106
FTL_0445	1460.986	0.927967	0.315997	2.936638	0.003318	0.054219
FTL_0446	601.4127	1.487612	0.279821	5.316294	1.06E-07	8.46E-06
FTL_0447	1538.691	0.964534	0.267136	3.610651	0.000305	0.009445
FTL_0448	1218.526	0.280886	0.244004	1.151149	0.249671	0.618143
FTL_0449	1100.285	-1.23759	0.263551	-4.69584	2.66E-06	0.000162
FTL_0450	6399.924	0.435151	0.26238	1.658473	0.097222	0.446156
FTL_0451	4444.996	0.898491	0.235089	3.821916	0.000132	0.004839
FTL_0452	3692.883	0.524614	0.237133	2.212315	0.026945	0.216637
FTL_0453	10303.85	0.634955	0.225652	2.813873	0.004895	0.068802
FTL_0454	10755.13	0.37662	0.23603	1.595643	0.110569	0.466602
FTL_0455	6599.243	0.615753	0.246311	2.499901	0.012423	0.120627
FTL_0456	3640.582	0.112776	0.302708	0.372558	0.709477	0.902349
FTL_0457	4763.952	0.059847	0.298224	0.200679	0.84095	0.950146
FTL_0458	271.3076	0.172996	0.329128	0.525618	0.599153	0.846309
FTL_0459	1935.552	0.524614	0.25229	2.079412	0.03758	0.264108
FTL_0460	2215.497	0.536763	0.252579	2.12513	0.033576	0.245315
FTL_0461	1979.512	0.636843	0.240667	2.64616	0.008141	0.094044
FTL_0462	7051.152	0.587678	0.229054	2.565679	0.010297	0.10669
FTL_0463	1882.791	0.51287	0.248875	2.060752	0.039327	0.270219
FTL_0464	1182.045	0.551088	0.247054	2.230633	0.025705	0.209182
FTL_0465	949.1012	0.616556	0.262363	2.350016	0.018773	0.164773
FTL_0466	3224.384	0.370306	0.235597	1.571778	0.116002	0.477814
FTL_0467	363.9333	0.547439	0.285249	1.91916	0.054964	0.326617
FTL_0468	292.3272	0.578656	0.313283	1.84707	0.064737	0.362455
FTL_0469	242.4142	1.048701	0.357721	2.931613	0.003372	0.05466

FTL_0470	1121.008	0.226243	0.271943	0.831949	0.405438	0.749791
FTL_0471	318.9288	0.800933	0.351412	2.279185	0.022656	0.191932
FTL_0472	7806.377	0.493064	0.237708	2.074243	0.038057	0.265604
FTL_0473	618.018	-0.09843	0.282988	-0.34781	0.72798	0.907717
FTL_0474	3816.88	0.736465	0.232897	3.162186	0.001566	0.031163
FTL_0475	1729.242	0.513895	0.243269	2.112451	0.034648	0.250338
FTL_0476	5919.078	0.493885	0.238787	2.068311	0.038611	0.268538
FTL_0477	6224.604	0.29611	0.233683	1.267141	0.205105	0.586521
FTL_0478	2682.305	0.184699	0.232996	0.792715	0.427944	0.768006
FTL_0479	14121.98	0.067493	0.231769	0.291207	0.770893	0.92177
FTL_0480	7356.685	-0.26104	0.270521	-0.96496	0.334566	0.687604
FTL_0481	2740.077	0.205213	0.261431	0.784958	0.432478	0.771103
FTL_0482	9205.086	0.529461	0.260537	2.032189	0.042134	0.282265
FTL_0483	8991.706	0.668327	0.240388	2.7802	0.005433	0.072876
FTL_0484	2843.346	0.333443	0.234811	1.420051	0.155593	0.544009
FTL_0485	5366.47	0.734208	0.25755	2.850735	0.004362	0.06431
FTL_0486	3380.75	0.406192	0.24979	1.626134	0.103921	0.451804
FTL_0487	7998.954	0.433181	0.232288	1.864839	0.062204	0.355199
FTL_0488	2409.251	0.238446	0.260431	0.915583	0.359886	0.712199
FTL_0489	3081.747	0.647262	0.236071	2.741812	0.00611	0.077241
FTL_0490	2398.819	0.721303	0.235319	3.065212	0.002175	0.039036
FTL_0491	1349.669	-0.11234	0.243926	-0.46055	0.645124	0.872239
FTL_0492	6897.456	0.916195	0.24556	3.73105	0.000191	0.006724
FTL_0493	3310.323	0.498341	0.232428	2.144067	0.032027	0.239313
FTL_0494	594.8741	0.760218	0.269265	2.823311	0.004753	0.067279
FTL_0495	54.00074	1.077854	0.391912	2.750247	0.005955	0.075857
FTL_0496	3.98687	0.171621	0.254356	0.674729	0.499848	0.800092
FTL_0497	11.67577	0.480423	0.361557	1.328764	0.183926	0.573164
FTL_0498	133.8292	1.123269	0.351911	3.19191	0.001413	0.028695
FTL_0499	1120.853	0.506285	0.276773	1.829243	0.067363	0.371978
FTL_0500	1038.895	1.123418	0.268854	4.178542	2.93E-05	0.00134
FTL_0501	229.5278	1.200286	0.343917	3.490043	0.000483	0.012943
FTL_0502	280.9967	1.314828	0.328507	4.002433	6.27E-05	0.00252
FTL_0503	913.8853	0.34832	0.261651	1.331237	0.183111	0.571974
FTL_0504	23.47687	0.962513	0.390825	2.462771	0.013787	0.131959
FTL_0505	103.096	0.926512	0.373869	2.478174	0.013206	0.127613
FTL_0506	673.3565	0.405625	0.267902	1.514082	0.130005	0.506185
FTL_0507	1135.805	0.372549	0.249838	1.491161	0.135919	0.514496
FTL_0508	3927.175	0.828096	0.249357	3.320928	0.000897	0.020037
FTL_0509	1173.339	0.759114	0.252385	3.007765	0.002632	0.045602
FTL_0510	1299.39	0.394005	0.246217	1.600233	0.109547	0.466502
FTL_0511	2207.034	1.310958	0.297949	4.399944	1.08E-05	0.000518
FTL_0512	831.7591	0.683013	0.252513	2.704857	0.006833	0.083251

FTL_0513	1220.963	1.10541	0.266892	4.141791	3.45E-05	0.001539
FTL_0514	4427.176	0.783209	0.229426	3.413777	0.000641	0.015899
FTL_0515	1549.452	0.288663	0.24376	1.18421	0.23633	0.609208
FTL_0516	1854.932	0.3561	0.246839	1.442642	0.149121	0.536197
FTL_0517	4076.187	0.694586	0.239276	2.902859	0.003698	0.058066
FTL_0518	3061.344	0.677922	0.250635	2.704823	0.006834	0.083251
FTL_0519	10229.36	-0.00556	0.253438	-0.02192	0.982512	0.995965
FTL_0520	17275.67	0.313654	0.228623	1.371926	0.170087	0.563239
FTL_0521	12392.08	0.572312	0.226122	2.530988	0.011374	0.114885
FTL_0522	12417.88	0.396544	0.233551	1.697894	0.089528	0.430505
FTL_0523	2897.807	0.552667	0.2427	2.277161	0.022777	0.191932
FTL_0524	1676.839	0.503531	0.259088	1.943472	0.051959	0.319864
FTL_0525	9320.001	0.638216	0.240167	2.657385	0.007875	0.092565
FTL_0526	1221.518	0.715241	0.271499	2.634416	0.008428	0.09571
FTL_0527	293.5375	1.260449	0.310168	4.063765	4.83E-05	0.002022
FTL_0528	3293.955	0.917373	0.249394	3.67841	0.000235	0.007609
FTL_0529	435.0271	0.47796	0.290564	1.64494	0.099982	0.448176
FTL_0530	76.82581	0.768588	0.379438	2.025595	0.042806	0.284903
FTL_0531	42.77617	0.943813	0.400767	2.355018	0.018522	0.164003
FTL_0532	159.053	0.735878	0.333986	2.203321	0.027572	0.218715
FTL_0533	33029.48	0.690022	0.23288	2.962995	0.003047	0.05146
FTL_0534	6710.671	0.416096	0.256286	1.623558	0.10447	0.451804
FTL_0535	10143.31	0.733328	0.237728	3.084739	0.002037	0.037917
FTL_0536	4444.644	0.587307	0.234112	2.50866	0.012119	0.118249
FTL_0537	9350.041	0.760714	0.230812	3.295811	0.000981	0.021677
FTL_0538	7872.643	0.390537	0.229688	1.700292	0.089076	0.430505
FTL_0539	15465.58	0.259009	0.243541	1.063514	0.287549	0.643901
FTL_0540	10876.33	0.424798	0.237928	1.785407	0.074195	0.38938
FTL_0541	2663.841	0.668721	0.235666	2.837584	0.004546	0.065732
FTL_0542	2136.314	1.063539	0.261533	4.066555	4.77E-05	0.002022
FTL_0543	2124.739	0.995632	0.333425	2.986078	0.002826	0.048135
FTL_0544	3398.84	0.835832	0.236647	3.531973	0.000412	0.012015
FTL_0545	2257.839	0.787734	0.252873	3.115136	0.001839	0.034538
FTL_0546	1886.564	0.358637	0.23893	1.501011	0.133353	0.509395
FTL_0547	3952.807	0.457017	0.234167	1.951668	0.050978	0.319864
FTL_0548	515.3755	0.697713	0.287775	2.424512	0.015329	0.141336
FTL_0549	930.1984	0.658596	0.25557	2.576967	0.009967	0.103803
FTL_0550	113.4634	0.451218	0.368648	1.22398	0.22096	0.593974
FTL_0551	980.9235	1.338059	0.363215	3.683931	0.00023	0.007609
FTL_0552	5601.339	0.307607	0.23112	1.330942	0.183208	0.571974
FTL_0553	4987.937	0.377541	0.230015	1.641378	0.100719	0.448176
FTL_0554	2224.087	0.228465	0.242433	0.942387	0.345995	0.701515
FTL_0555	1947.402	0.376177	0.255762	1.470812	0.141342	0.523071

FTL_0556	7960.666	0.246068	0.26904	0.914616	0.360393	0.712199
FTL_0557	1185.468	0.942538	0.280662	3.358272	0.000784	0.018331
FTL_0558	219.3531	0.881012	0.317285	2.776721	0.005491	0.072876
FTL_0559	139.3303	1.333015	0.359621	3.706722	0.00021	0.007276
FTL_0560	107.0105	1.463957	0.381394	3.838432	0.000124	0.004609
FTL_0561	202.3831	1.099166	0.349246	3.147253	0.001648	0.032162
FTL_0562	203.5356	0.861889	0.32609	2.643102	0.008215	0.094355
FTL_0563	151.0039	1.137771	0.347286	3.276175	0.001052	0.022742
FTL_0564	109.2229	1.636999	0.366436	4.467348	7.92E-06	0.000419
FTL_0565	70.23264	0.840948	0.388914	2.162299	0.030595	0.235382
FTL_0566	321.5599	1.025887	0.315515	3.251471	0.001148	0.02455
FTL_0567	125.4892	0.38882	0.34186	1.137366	0.255385	0.622966
FTL_0568	258.294	0.475554	0.315302	1.508248	0.131491	0.507288
FTL_0569	2715.076	-0.27276	0.26986	-1.01074	0.312142	0.663911
FTL_0570	803.4717	0.018993	0.291866	0.065075	0.948115	0.989719
FTL_0571	2317.209	0.382693	0.249054	1.536588	0.124394	0.494135
FTL_0572	7414.049	0.512674	0.241103	2.126374	0.033472	0.245315
FTL_0573	4152.783	0.357755	0.254656	1.404855	0.160064	0.550633
FTL_0574	3831.766	0.78411	0.231557	3.386258	0.000709	0.016928
FTL_0575	552.418	0.348732	0.267139	1.305434	0.191745	0.575235
FTL_0576	128.9909	0.895634	0.367897	2.434471	0.014914	0.139425
FTL_0577	111.1354	1.050227	0.371687	2.825571	0.00472	0.067279
FTL_0578	6304.635	0.514823	0.228465	2.253404	0.024234	0.200452
FTL_0579	2166.912	0.652814	0.242458	2.692488	0.007092	0.08536
FTL_0580	1745.717	0.362308	0.287095	1.261979	0.206956	0.586521
FTL_0581	2680.384	0.471792	0.270178	1.746229	0.080771	0.406893
FTL_0582	2230.143	0.157074	0.292088	0.537761	0.590742	0.843318
FTL_0583	6502.52	0.263429	0.27168	0.969629	0.332232	0.685589
FTL_0584	20152.15	0.268742	0.272974	0.984497	0.324871	0.678056
FTL_0585	16084.13	0.154854	0.260185	0.59517	0.55173	0.825132
FTL_0586	5221.572	1.052828	0.242936	4.333768	1.47E-05	0.000685
FTL_0587	715.8863	0.718843	0.258873	2.776819	0.005489	0.072876
FTL_0588	22635.79	0.689431	0.225775	3.053621	0.002261	0.039865
FTL_0589	7168.189	0.464605	0.241108	1.926961	0.053984	0.326617
FTL_0590	8975.911	0.688317	0.250054	2.752671	0.005911	0.075857
FTL_0591	186.2138	0.511794	0.340163	1.504553	0.132439	0.509395
FTL_0592	9073.212	0.162485	0.226932	0.716007	0.473987	0.787814
FTL_0593	4083.116	-0.03342	0.233897	-0.14289	0.886376	0.965122
FTL_0594	3631.323	-0.36075	0.257354	-1.40177	0.160985	0.551243
FTL_0595	5980.772	-0.25001	0.236591	-1.05672	0.290637	0.643901
FTL_0596	10301.61	-0.4846	0.26808	-1.80769	0.070655	0.378915
FTL_0597	7596.476	-0.25362	0.245359	-1.03368	0.301285	0.652888
FTL_0598	1760.186	0.170123	0.286474	0.593851	0.552612	0.825669

FTL_0599	1518.942	-0.47435	0.267996	-1.77	0.076727	0.395437
FTL_0600	5766.326	-0.72084	0.257949	-2.7945	0.005198	0.071328
FTL_0601	4159.7	-0.66437	0.273313	-2.43078	0.015066	0.1402
FTL_0602	3417.15	-0.66056	0.268436	-2.46077	0.013864	0.132069
FTL_0603	3456.995	-0.68298	0.270816	-2.52194	0.011671	0.116709
FTL_0604	3855.603	-0.6618	0.276693	-2.3918	0.016766	0.151118
FTL_0605	3691.665	-0.74416	0.260003	-2.86212	0.004208	0.063123
FTL_0606	3788.817	-0.63598	0.229968	-2.76552	0.005683	0.074176
FTL_0607	1211.672	0.189012	0.25429	0.743294	0.457304	0.77919
FTL_0608	2247.446	0.044563	0.235876	0.188926	0.850151	0.952754
FTL_0609	1551.95	-0.10591	0.258012	-0.41049	0.681447	0.884928
FTL_0610	12749.41	-0.45169	0.250086	-1.80613	0.070898	0.378915
FTL_0611	4700.018	-0.21092	0.231036	-0.91294	0.361276	0.712199
FTL_0612	1327.013	0.151133	0.250789	0.60263	0.546755	0.824572
FTL_0613	680.4629	0.544829	0.320803	1.698332	0.089445	0.430505
FTL_0614	79.30218	0.450575	0.384819	1.170874	0.24165	0.612533
FTL_0615	296.515	1.358672	0.358393	3.791018	0.00015	0.005385
FTL_0616	16079.14	-0.01891	0.239836	-0.07883	0.937165	0.98417
FTL_0617	20272.75	-0.25189	0.234813	-1.07274	0.28339	0.643901
FTL_0618	131.9238	-0.4822	0.339711	-1.41944	0.155772	0.544009
FTL_0619	196.3878	-0.16656	0.319992	-0.52053	0.602696	0.848928
FTL_0620	974.8452	-0.01337	0.25184	-0.05307	0.957676	0.992291
FTL_0621	1549.176	0.429029	0.261247	1.642234	0.100542	0.448176
FTL_0622	374.7079	0.235126	0.303845	0.773835	0.439029	0.773875
FTL_0623	904.1755	0.009241	0.264273	0.034967	0.972106	0.994309
FTL_0624	1165.53	0.067766	0.247288	0.274035	0.784057	0.930316
FTL_0625	1954.405	-0.02173	0.240399	-0.0904	0.927969	0.981177
FTL_0626	628.7943	0.249653	0.290382	0.85974	0.389933	0.736428
FTL_0627	589.5569	-0.18853	0.282545	-0.66726	0.504605	0.802678
FTL_0628	662.0328	-0.11628	0.259041	-0.44888	0.653518	0.875536
FTL_0629	688.9116	0.1012	0.262023	0.386225	0.69933	0.897755
FTL_0630	256.8022	0.098475	0.304223	0.323693	0.74617	0.914459
FTL_0631	906.8286	0.320237	0.260744	1.228166	0.219385	0.592692
FTL_0632	137.6689	0.691047	0.358238	1.929016	0.053729	0.326288
FTL_0633	241.3204	1.296136	0.35024	3.700706	0.000215	0.007325
FTL_0634	117.8341	1.169377	0.380567	3.072726	0.002121	0.03863
FTL_0635	137.1307	0.729284	0.348396	2.093264	0.036326	0.258916
FTL_0636	139.2867	0.636002	0.337692	1.883381	0.059649	0.344523
FTL_0637	710.3779	-0.38201	0.264618	-1.44363	0.148842	0.53615
FTL_0638	889.258	0.470559	0.283241	1.661336	0.096646	0.445547
FTL_0639	2217.659	0.456954	0.26653	1.714454	0.086445	0.42483
FTL_0640	1394.003	-0.10734	0.242379	-0.44287	0.657859	0.87702
FTL_0641	109.49	0.319754	0.351483	0.909728	0.362966	0.712387

FTL_0642	74.32009	0.230074	0.37911	0.60688	0.543931	0.824572
FTL_0643	957.0762	-0.09448	0.261054	-0.36193	0.717406	0.904215
FTL_0644	2972.551	0.15179	0.240475	0.631209	0.527904	0.812401
FTL_0645	18712.34	0.590453	0.259561	2.274812	0.022917	0.191932
FTL_0646	4147.371	0.648331	0.293426	2.209521	0.027138	0.217323
FTL_0647	179.1795	1.01992	0.344904	2.95711	0.003105	0.051585
FTL_0648	962.974	0.49415	0.261731	1.888007	0.059025	0.341903
FTL_0649	1583.992	-0.07383	0.244829	-0.30154	0.763	0.920389
FTL_0650	7745.461	0.196503	0.23913	0.82174	0.411225	0.752775
FTL_0651	98.82225	0.458473	0.356444	1.28624	0.198359	0.583892
FTL_0652	1290.724	0.360829	0.268915	1.341796	0.179662	0.569084
FTL_0653	679.865	-0.15933	0.263836	-0.6039	0.545908	0.824572
FTL_0654	1049.837	0.096233	0.251225	0.383056	0.701678	0.897755
FTL_0655	1861.066	-0.00452	0.245904	-0.01839	0.985328	0.995965
FTL_0656	1807.761	-0.29181	0.259524	-1.12439	0.260849	0.628129
FTL_0657	1216.599	-0.20902	0.244823	-0.85375	0.393242	0.738706
FTL_0659	1323.634	0.172985	0.281797	0.613864	0.539305	0.822868
FTL_0660	424.3091	0.217061	0.279235	0.77734	0.436958	0.771103
FTL_0661	3992.148	-0.08947	0.247305	-0.36177	0.717524	0.904215
FTL_0662	3381.098	0.363722	0.369695	0.983844	0.325192	0.678056
FTL_0663	448.4645	-0.01142	0.357041	-0.03199	0.974482	0.994309
FTL_0664	1139.568	0.188747	0.328386	0.574771	0.565446	0.829596
FTL_0665	1232.968	-0.10493	0.248815	-0.42174	0.673218	0.881992
FTL_0666	2378.83	-0.21336	0.241545	-0.88331	0.377067	0.725961
FTL_0667	359.5086	-0.17535	0.297039	-0.59034	0.554965	0.826281
FTL_0668	1108.421	-0.35777	0.263623	-1.35714	0.174738	0.565576
FTL_0669	2446.815	-0.16959	0.241865	-0.70117	0.483195	0.792187
FTL_0670	1341.155	-0.57151	0.24507	-2.33203	0.019699	0.171914
FTL_0671	9757.021	-1.28582	0.230649	-5.57478	2.48E-08	2.26E-06
FTL_0672	2386.119	-1.34774	0.24362	-5.53214	3.16E-08	2.76E-06
FTL_0673	14861.93	-1.20642	0.227479	-5.30341	1.14E-07	8.46E-06
FTL_0674	9262.1	-1.39799	0.236891	-5.90141	3.60E-09	4.31E-07
FTL_0675	5272.46	-1.20047	0.245978	-4.88038	1.06E-06	7.09E-05
FTL_0676	3232.905	0.041531	0.237332	0.174991	0.861087	0.955707
FTL_0677	416.3757	0.421388	0.306004	1.377068	0.168491	0.563239
FTL_0678	1100.689	0.317979	0.257372	1.235485	0.21665	0.590864
FTL_0679	2051.652	0.086558	0.261775	0.330658	0.740903	0.91279
FTL_0680	3878.573	0.251036	0.24719	1.015558	0.30984	0.660785
FTL_0681	6216.867	-0.04089	0.256289	-0.15953	0.873248	0.958064
FTL_0682	875.6257	0.334548	0.258481	1.294285	0.195567	0.582355
FTL_0683	874.0009	0.862304	0.331201	2.603568	0.009226	0.099256
FTL_0684	2239.063	0.391241	0.253555	1.543022	0.122825	0.48984
FTL_0685	4068.672	0.004556	0.260674	0.017479	0.986055	0.995965

FTL_0686	8633.951	-0.12427	0.309516	-0.4015	0.688055	0.891677
FTL_0687	9021.926	-0.06147	0.289134	-0.2126	0.831638	0.945471
FTL_0688	6660.31	0.226536	0.257275	0.880521	0.378577	0.726782
FTL_0689	550.6881	0.190123	0.274879	0.69166	0.489151	0.793769
FTL_0690	2525.69	-0.03001	0.23685	-0.12671	0.899166	0.969455
FTL_0691	6057.98	-0.79725	0.28216	-2.82554	0.00472	0.067279
FTL_0692	425.3475	-0.06879	0.286227	-0.24034	0.810069	0.939549
FTL_0693	558.7053	0.26342	0.284016	0.927486	0.353674	0.707282
FTL_0694	1844.15	0.03388	0.254007	0.133384	0.89389	0.96895
FTL_0695	984.4727	-0.06321	0.251974	-0.25088	0.80191	0.937924
FTL_0696	1168.719	-0.1415	0.263558	-0.53687	0.591354	0.843592
FTL_0697	1756.03	0.139219	0.242193	0.574827	0.565408	0.829596
FTL_0698	799.8905	0.151258	0.25584	0.591219	0.554374	0.826281
FTL_0699	1623.355	-0.20908	0.246156	-0.84937	0.395675	0.74145
FTL_0700	754.9204	-0.1369	0.268428	-0.51002	0.610037	0.851082
FTL_0701	1373.601	0.161045	0.246312	0.653823	0.513226	0.804396
FTL_0702	597.7261	0.047096	0.290219	0.162279	0.871086	0.958064
FTL_0703	8546.224	-0.3499	0.243446	-1.43728	0.150639	0.539722
FTL_0704	1221.458	-0.27089	0.251085	-1.07888	0.280639	0.643199
FTL_0705	1427.604	0.174607	0.24535	0.711666	0.476672	0.788568
FTL_0706	1352.221	0.336256	0.270225	1.244356	0.213369	0.587852
FTL_0707	2160.392	-0.18666	0.246047	-0.75864	0.448067	0.77779
FTL_0708	1250.659	-0.02616	0.249168	-0.10498	0.916389	0.97571
FTL_0709	818.1323	-0.14061	0.256795	-0.54754	0.584006	0.838244
FTL_0710	332.6715	0.289046	0.304626	0.948854	0.342695	0.698762
FTL_0711	182.3303	0.231182	0.331781	0.696792	0.485933	0.793441
FTL_0712	147.6177	0.276668	0.372133	0.743465	0.4572	0.77919
FTL_0713	1079.368	-0.36522	0.24792	-1.47312	0.140719	0.523071
FTL_0714	4111.949	-0.62521	0.245805	-2.54352	0.010974	0.111969
FTL_0715	3113.772	-0.34443	0.253448	-1.35898	0.174151	0.565576
FTL_0716	4096.332	-0.23509	0.246271	-0.95458	0.33979	0.69623
FTL_0717	15173.45	0.104795	0.235683	0.444641	0.656579	0.87702
FTL_0718	3130.859	0.669688	0.288945	2.317699	0.020466	0.175887
FTL_0719	374.3439	-0.15159	0.283205	-0.53525	0.592478	0.844596
FTL_0720	625.4026	0.404286	0.28775	1.404988	0.160025	0.550633
FTL_0721	2241.677	0.177607	0.272265	0.652331	0.514187	0.804396
FTL_0722	1331.943	-0.13309	0.260561	-0.51078	0.609506	0.851082
FTL_0723	1153.716	0.310299	0.273255	1.135565	0.256138	0.623375
FTL_0724	1847.172	0.117627	0.274669	0.428251	0.668468	0.881666
FTL_0725	825.8357	0.058194	0.252799	0.230197	0.817938	0.940536
FTL_0726	1638.135	0.170189	0.243495	0.698944	0.484587	0.79253
FTL_0727	1419.062	-0.16981	0.256364	-0.66237	0.507734	0.804313
FTL_0728	255.15	0.031858	0.303821	0.104859	0.916488	0.97571

FTL_0729	1612.237	0.383818	0.307189	1.249455	0.211499	0.587629
FTL_0730	2172.289	0.066501	0.241368	0.275516	0.78292	0.930064
FTL_0731	3547.369	-0.06523	0.269786	-0.24177	0.808957	0.939549
FTL_0732	2032.899	0.629988	0.28919	2.178461	0.029372	0.227943
FTL_0733	1100.31	0.292072	0.253988	1.149941	0.250168	0.618143
FTL_0734	473.3317	0.748269	0.305528	2.449097	0.014321	0.135146
FTL_0735	324.8553	0.374034	0.335386	1.115235	0.26475	0.630978
FTL_0736	2596.451	-0.26415	0.239691	-1.10206	0.270436	0.634024
FTL_0737	2183.732	-0.1662	0.242392	-0.68567	0.492921	0.79605
FTL_0738	882.9216	-0.01446	0.260719	-0.05548	0.955758	0.991777
FTL_0739	10073.7	0.097214	0.226675	0.42887	0.668018	0.881666
FTL_0740	465.5641	0.250243	0.27228	0.919067	0.35806	0.710465
FTL_0741	785.2654	0.301086	0.270241	1.114139	0.265219	0.630978
FTL_0742	363.4132	0.291037	0.334104	0.871096	0.383702	0.733885
FTL_0743	1500.152	0.176277	0.243305	0.724511	0.468752	0.783202
FTL_0744	2357.372	-0.04159	0.240223	-0.17312	0.862557	0.956083
FTL_0745	2456.267	-0.39457	0.278062	-1.41901	0.155895	0.544009
FTL_0746	947.5189	0.053042	0.259155	0.204672	0.837828	0.949983
FTL_0747	913.7833	-0.10503	0.268546	-0.3911	0.695724	0.896414
FTL_0748	1449.789	0.382945	0.250306	1.529906	0.12604	0.497722
FTL_0749	182.7498	0.471839	0.335657	1.405717	0.159808	0.550633
FTL_0750	1540.61	0.534612	0.304255	1.757118	0.078898	0.404552
FTL_0751	965.7406	0.120708	0.250459	0.481946	0.629844	0.861561
FTL_0752	745.6725	-0.1769	0.258326	-0.6848	0.493472	0.79605
FTL_0753	193.7042	-0.40991	0.320665	-1.2783	0.201143	0.58465
FTL_0754	1827.229	0.407878	0.246272	1.65621	0.097679	0.446264
FTL_0755	1435.94	0.506539	0.243146	2.083269	0.037227	0.262546
FTL_0756	2511.677	0.422378	0.237232	1.780444	0.075003	0.390561
FTL_0757	702.3449	-0.20817	0.303509	-0.68588	0.492787	0.79605
FTL_0758	384.2312	-0.09017	0.290339	-0.31056	0.756136	0.919801
FTL_0759	343.5899	-0.2415	0.300718	-0.80307	0.421932	0.762684
FTL_0760	2110.97	-0.03298	0.238783	-0.13813	0.890135	0.967119
FTL_0761	1246.354	0.136441	0.252832	0.539649	0.589439	0.842174
FTL_0762	790.8748	-0.20188	0.289432	-0.69751	0.485484	0.793352
FTL_0763	1686.68	0.056827	0.243294	0.233575	0.815315	0.939669
FTL_0764	1566.524	0.141996	0.240491	0.590443	0.554894	0.826281
FTL_0765	998.2887	-0.27697	0.297214	-0.9319	0.35139	0.705351
FTL_0766	1471.715	-0.67624	0.256329	-2.63817	0.008335	0.095195
FTL_0767	791.9414	-0.61358	0.256393	-2.39311	0.016706	0.151118
FTL_0768	8173.362	-0.18374	0.265581	-0.69183	0.489045	0.793769
FTL_0769	287.1369	0.664864	0.341945	1.944359	0.051852	0.319864
FTL_0770	113.392	0.492761	0.373637	1.318825	0.187228	0.575235
FTL_0771	376.273	0.284486	0.298911	0.951741	0.341229	0.697731

FTL_0772	49.67755	0.497258	0.396075	1.255466	0.20931	0.587587
FTL_0773	33.48287	0.567506	0.400869	1.415691	0.156866	0.545742
FTL_0774	13.49951	0.25378	0.374526	0.677603	0.498024	0.800092
FTL_0775	12.83605	0.308729	0.371332	0.831409	0.405743	0.749791
FTL_0776	77.50632	-0.2074	0.375382	-0.55249	0.58061	0.834783
FTL_0777	15.40926	-0.27285	0.382117	-0.71405	0.475196	0.788011
FTL_0778	68.12506	0.025108	0.377586	0.066497	0.946982	0.989719
FTL_0779	166.0117	0.274523	0.330038	0.831791	0.405527	0.749791
FTL_0780	135.6773	0.443039	0.352378	1.257285	0.208651	0.586556
FTL_0781	177.1012	0.416928	0.334449	1.24661	0.21254	0.587629
FTL_0782	122.3537	0.686503	0.35789	1.918196	0.055086	0.326617
FTL_0783	934.0351	0.2886	0.257022	1.122859	0.261497	0.628129
FTL_0784	3567.816	-0.02285	0.233367	-0.09792	0.921998	0.977806
FTL_0785	1060.123	-0.09338	0.260635	-0.35828	0.720131	0.905629
FTL_0786	2507.461	0.35233	0.234905	1.499884	0.133644	0.509395
FTL_0787	1233.738	0.186724	0.254501	0.733685	0.463141	0.77919
FTL_0788	1546.236	0.16964	0.244301	0.694389	0.487438	0.793769
FTL_0789	2877.038	-0.27319	0.270469	-1.01006	0.312468	0.663911
FTL_0790	1493.648	-0.18768	0.257967	-0.72753	0.466903	0.780761
FTL_0791	186.1907	-0.10612	0.320165	-0.33145	0.740307	0.91279
FTL_0792	1004.289	-0.19352	0.256489	-0.75448	0.45056	0.778339
FTL_0793	309.1507	-0.066	0.294629	-0.224	0.822755	0.943914
FTL_0794	90.07018	0.157156	0.369503	0.425317	0.670605	0.881666
FTL_0795	4923.22	-0.36669	0.232433	-1.5776	0.114658	0.475181
FTL_0796	2956.105	0.103246	0.232573	0.443931	0.657092	0.87702
FTL_0797	1073.972	0.431975	0.261382	1.65266	0.0984	0.446841
FTL_0798	806.3313	0.150937	0.257675	0.585764	0.558034	0.828397
FTL_0799	1588.541	0.434657	0.251792	1.726254	0.084302	0.42041
FTL_0800	4717.027	-0.04817	0.255286	-0.1887	0.850328	0.952754
FTL_0801	5856.464	-0.1	0.238251	-0.41972	0.674693	0.882899
FTL_0802	6590.3	-0.10946	0.22716	-0.48187	0.629901	0.861561
FTL_0803	3980.89	-0.17369	0.280827	-0.6185	0.536244	0.820906
FTL_0804	181.5601	0.070971	0.322102	0.220337	0.825609	0.945294
FTL_0805	17650.96	-0.13925	0.242561	-0.57409	0.565908	0.829669
FTL_0806	3035.946	-0.99546	0.252543	-3.94175	8.09E-05	0.003068
FTL_0807	2107.977	-0.39723	0.255443	-1.55506	0.119933	0.483237
FTL_0808	1714.785	-0.4535	0.269846	-1.68058	0.092844	0.441172
FTL_0809	2294.69	-0.43179	0.240959	-1.79195	0.073141	0.386875
FTL_0810	109.3684	0.101189	0.369022	0.274209	0.783924	0.930316
FTL_0811	57.3972	0.254232	0.394354	0.64468	0.519135	0.806384
FTL_0812	34.369	-0.149	0.400409	-0.37213	0.709795	0.902349
FTL_0813	176.7907	-0.15424	0.332465	-0.46392	0.642703	0.870508
FTL_0814	63.9314	-0.59792	0.381535	-1.56715	0.117079	0.478205

FTL_0815	32.4588	-1.10245	0.400918	-2.74982	0.005963	0.075857
FTL_0816	29.24583	-1.19674	0.400453	-2.98846	0.002804	0.048135
FTL_0817	67.83377	0.41368	0.380763	1.086451	0.277279	0.638231
FTL_0818	43.2942	0.220202	0.396617	0.555201	0.578757	0.833908
FTL_0819	58.01788	0.285632	0.386359	0.739292	0.45973	0.77919
FTL_0820	206.2125	0.395056	0.32344	1.221418	0.221928	0.593974
FTL_0821	910.7159	-0.10769	0.250388	-0.4301	0.667121	0.881666
FTL_0822	1059.787	0.029577	0.258964	0.114213	0.909069	0.972447
FTL_0823	1167.355	-0.00699	0.259057	-0.027	0.978459	0.994309
FTL_0824	1253.031	0.117198	0.259942	0.450861	0.652089	0.874967
FTL_0825	992.8775	-0.12462	0.259094	-0.48099	0.630526	0.861561
FTL_0826	351.7743	-0.18953	0.284423	-0.66638	0.505168	0.802678
FTL_0827	3411.474	-0.17304	0.235558	-0.7346	0.462582	0.77919
FTL_0828	7422.828	0.030743	0.230365	0.133452	0.893836	0.96895
FTL_0829	1696.958	0.302387	0.251295	1.203314	0.228855	0.601305
FTL_0830	2183.989	0.310773	0.256807	1.210143	0.226224	0.596733
FTL_0831	4173.349	-0.78926	0.233271	-3.38343	0.000716	0.016928
FTL_0832	3030.095	-1.31286	0.247313	-5.3085	1.11E-07	8.46E-06
FTL_0833	1258.863	-0.47651	0.247482	-1.92542	0.054177	0.326617
FTL_0834	2173.444	-0.43007	0.237426	-1.81137	0.070084	0.378915
FTL_0835	265.4615	0.154459	0.346301	0.446024	0.65558	0.876723
FTL_0836	384.2466	0.075664	0.296434	0.255247	0.798532	0.937924
FTL_0837	2650.793	-0.53661	0.24327	-2.20583	0.027396	0.218518
FTL_0838	1719.635	-0.49213	0.257834	-1.90871	0.056299	0.330881
FTL_0839	1302.794	-0.41731	0.250208	-1.66783	0.095349	0.445199
FTL_0840	344.6252	-0.23465	0.285627	-0.82154	0.411337	0.752775
FTL_0841	431.1144	-0.17359	0.274028	-0.63348	0.526423	0.812401
FTL_0842	135.2448	0.164765	0.34436	0.478469	0.632316	0.863421
FTL_0843	2929.693	0.163712	0.235998	0.6937	0.48787	0.793769
FTL_0844	801.1082	0.714914	0.304119	2.350771	0.018735	0.164773
FTL_0845	114.8305	0.017842	0.347359	0.051365	0.959034	0.992291
FTL_0846	545.1951	-0.13631	0.283051	-0.48158	0.630101	0.861561
FTL_0847	1665.136	-0.24327	0.259414	-0.93777	0.348364	0.703126
FTL_0848	8435.38	-0.19126	0.251407	-0.76078	0.44679	0.776858
FTL_0849	2522.912	-0.30371	0.278133	-1.09196	0.274849	0.638231
FTL_0850	3104.5	-0.15545	0.282767	-0.54974	0.582499	0.836901
FTL_0851	5351.437	-0.20134	0.278375	-0.72327	0.469515	0.783825
FTL_0852	2936.3	-0.03713	0.264471	-0.14038	0.88836	0.966759
FTL_0853	761.3182	0.137258	0.26657	0.514903	0.606621	0.851082
FTL_0854	528.3597	0.364688	0.269775	1.351824	0.176432	0.569084
FTL_0855	1206.226	-0.05662	0.2601	-0.21768	0.827675	0.945294
FTL_0856	1330.591	-0.25565	0.243306	-1.05074	0.293377	0.643901
FTL_0857	411.2281	-0.17206	0.294802	-0.58363	0.559469	0.828816

FTL_0858	2345.372	0.093134	0.243078	0.383143	0.701614	0.897755
FTL_0859	656.6755	-0.1237	0.259976	-0.47582	0.634204	0.865012
FTL_0860	315.0028	0.214041	0.309751	0.69101	0.489559	0.793769
FTL_0861	183.9008	0.468724	0.335653	1.396451	0.162579	0.554467
FTL_0862	968.1224	0.60737	0.291506	2.083562	0.0372	0.262546
FTL_0863	3507.062	0.000326	0.247784	0.001316	0.99895	0.99895
FTL_0864	1706.364	-0.03017	0.241474	-0.12493	0.90058	0.969558
FTL_0865	3627.647	-0.11432	0.233436	-0.48973	0.624324	0.860048
FTL_0866	646.5391	0.346345	0.315705	1.097052	0.272618	0.63686
FTL_0867	4140.974	0.69528	0.240617	2.889573	0.003858	0.059645
FTL_0868	205.1454	0.439203	0.335471	1.309215	0.190461	0.575235
FTL_0869	32.91326	0.252665	0.400798	0.630405	0.528429	0.812401
FTL_0870	17.7195	0.386347	0.376695	1.025622	0.30507	0.656278
FTL_0871	64.34951	0.39537	0.388814	1.016862	0.309219	0.660785
FTL_0872	107.4146	0.762112	0.391584	1.946226	0.051628	0.319864
FTL_0873	116.8626	0.694456	0.379513	1.829861	0.067271	0.371978
FTL_0874	73.97706	-0.28613	0.373838	-0.76539	0.44404	0.775733
FTL_0875	4161.651	0.286754	0.229299	1.250565	0.211093	0.587629
FTL_0876	2779.246	-0.04834	0.251304	-0.19235	0.847466	0.952704
FTL_0877	7325.362	-0.49892	0.238268	-2.09394	0.036265	0.258916
FTL_0878	1257.364	-0.29933	0.244853	-1.22251	0.221516	0.593974
FTL_0879	793.965	-1.20259	0.260233	-4.62121	3.82E-06	0.000219
FTL_0880	327.558	-0.12138	0.331281	-0.36641	0.714059	0.903815
FTL_0881	142.7729	-0.38975	0.338617	-1.15101	0.249728	0.618143
FTL_0882	1826.01	-0.07824	0.265314	-0.29491	0.768064	0.920898
FTL_0883	1224.313	-0.21168	0.290542	-0.72857	0.466265	0.780343
FTL_0884	1649.487	0.044726	0.252799	0.176923	0.859568	0.954771
FTL_0885	7061.538	0.200156	0.237278	0.843548	0.398922	0.745891
FTL_0886	8586.56	-0.00693	0.253333	-0.02735	0.978184	0.994309
FTL_0887	943.7363	0.215961	0.272073	0.793762	0.427334	0.767597
FTL_0888	572.0898	0.464021	0.283496	1.636784	0.101676	0.448176
FTL_0889	132.3757	0.054793	0.341831	0.160294	0.87265	0.958064
FTL_0890	486.1876	0.266792	0.289569	0.921342	0.356872	0.708807
FTL_0891	8642.891	0.195354	0.262861	0.743181	0.457372	0.77919
FTL_0892	3187.045	-0.46633	0.263977	-1.76655	0.077303	0.397389
FTL_0893	7822.602	-0.30611	0.24769	-1.23587	0.216506	0.590864
FTL_0894	17543.79	-0.30422	0.239097	-1.27237	0.203242	0.585626
FTL_0895	3329.993	-0.88942	0.26774	-3.32196	0.000894	0.020037
FTL_0896	3153.94	-0.31577	0.233274	-1.35364	0.17585	0.568262
FTL_0897	1138.692	-0.36282	0.26949	-1.34633	0.178196	0.569084
FTL_0898	4416.505	-0.13489	0.27464	-0.49115	0.623324	0.860048
FTL_0899	12522.25	-0.0476	0.246897	-0.1928	0.847117	0.952704
FTL_0900	1518.882	-0.08732	0.265672	-0.32868	0.742396	0.91279

FTL_0901	479.2631	0.011067	0.285754	0.038728	0.969108	0.994309
FTL_0902	206.0559	-0.19685	0.313048	-0.62881	0.529475	0.812401
FTL_0903	6129.317	-0.09097	0.248005	-0.36683	0.713749	0.903815
FTL_0904	3671.743	-0.25717	0.292829	-0.87822	0.379824	0.728479
FTL_0905	2576.141	-0.10461	0.277494	-0.37697	0.706198	0.9001
FTL_0906	3290.678	-0.13542	0.272378	-0.49717	0.619069	0.856976
FTL_0907	830.4073	-0.22532	0.280475	-0.80335	0.421772	0.762684
FTL_0908	3242.895	0.507318	0.264447	1.918412	0.055059	0.326617
FTL_0909	141.0815	0.323137	0.338521	0.954557	0.339802	0.69623
FTL_0910	66.50453	0.503215	0.38037	1.322962	0.185848	0.575235
FTL_0911	207.8826	-0.00233	0.323532	-0.00721	0.994245	0.996724
FTL_0912	805.2751	0.625986	0.294947	2.122365	0.033807	0.245315
FTL_0913	5872.859	-0.34845	0.265498	-1.31244	0.189371	0.575235
FTL_0914	1638.976	-0.03031	0.254852	-0.11891	0.905344	0.971565
FTL_0915	1334.619	0.003416	0.257587	0.013263	0.989418	0.996358
FTL_0916	6055.02	-0.22168	0.269007	-0.82408	0.409894	0.752409
FTL_0917	8126.539	0.172595	0.23242	0.742601	0.457723	0.77919
FTL_0918	4583.504	-0.09598	0.241605	-0.39725	0.691183	0.892747
FTL_0919	1345.3	0.009637	0.249508	0.038624	0.96919	0.994309
FTL_0920	1438.784	0.362859	0.287673	1.261361	0.207179	0.586521
FTL_0921	1040.485	-0.32467	0.259242	-1.2524	0.210423	0.587629
FTL_0922	2771.757	-0.27436	0.232942	-1.17782	0.23887	0.609208
FTL_0923	3417.253	-0.41506	0.237041	-1.751	0.079946	0.406289
FTL_0924	171.9613	-1.03395	0.330963	-3.12406	0.001784	0.033824
FTL_0925	1826.78	0.441136	0.269413	1.637395	0.101548	0.448176
FTL_0926	2298.001	-0.39718	0.254898	-1.5582	0.119186	0.483237
FTL_0927	1824.179	-0.07781	0.246189	-0.31605	0.751964	0.918813
FTL_0928	3175.814	0.054279	0.231893	0.234069	0.814931	0.939669
FTL_0929	3281.933	-0.07354	0.241327	-0.30472	0.76058	0.919801
FTL_0930	1759.166	-0.14643	0.26344	-0.55582	0.578335	0.833908
FTL_0931	2230.236	0.006388	0.237723	0.026871	0.978563	0.994309
FTL_0932	1080.454	-0.00222	0.246162	-0.00903	0.992798	0.996724
FTL_0933	1671.979	0.109765	0.244594	0.448766	0.6536	0.875536
FTL_0934	195.3178	0.082429	0.323293	0.254966	0.798749	0.937924
FTL_0935	431.9648	-0.12502	0.273942	-0.45638	0.648118	0.874307
FTL_0936	174.3508	0.401621	0.343687	1.168564	0.242579	0.612533
FTL_0937	871.8105	-0.37631	0.28277	-1.33079	0.183259	0.571974
FTL_0938	1757.837	-0.46879	0.245944	-1.9061	0.056637	0.330934
FTL_0939	904.7463	-0.66295	0.284611	-2.32931	0.019843	0.171914
FTL_0940	127.3534	-0.75059	0.344269	-2.18025	0.029239	0.227794
FTL_0941	886.3988	-0.88712	0.276458	-3.2089	0.001332	0.02761
FTL_0942	1501.826	-0.62904	0.281805	-2.23217	0.025604	0.209182
FTL_0943	802.3816	0.273202	0.292662	0.933509	0.350557	0.704956

FTL_0944	252.324	0.062556	0.314503	0.198906	0.842337	0.950644
FTL_0945	295.9682	0.024972	0.298211	0.083739	0.933264	0.982421
FTL_0946	206.4992	-0.00999	0.327021	-0.03056	0.975618	0.994309
FTL_0947	144.0388	-0.08014	0.341737	-0.2345	0.814594	0.939669
FTL_0948	780.401	0.411325	0.299235	1.374591	0.169258	0.563239
FTL_0949	7579.236	-0.36045	0.262892	-1.37109	0.170345	0.563239
FTL_0950	3635.679	-0.24903	0.275644	-0.90346	0.366281	0.713396
FTL_0951	3709.382	-0.08349	0.246288	-0.339	0.734611	0.911008
FTL_0952	128.9442	-0.17277	0.345199	-0.5005	0.616724	0.856147
FTL_0953	1134.513	-1.19251	0.258473	-4.61367	3.96E-06	0.000221
FTL_0954	379.4026	-0.05401	0.288303	-0.18734	0.851394	0.952841
FTL_0955	2249.452	-0.17385	0.258397	-0.67279	0.501078	0.800092
FTL_0956	570.5161	-0.04595	0.268166	-0.17136	0.863943	0.956083
FTL_0957	696.8533	0.006832	0.262262	0.02605	0.979218	0.994309
FTL_0958	1011.436	0.121975	0.250133	0.48764	0.625805	0.860048
FTL_0959	1515.799	0.181202	0.257659	0.703262	0.481893	0.79199
FTL_0960	5229.43	0.061682	0.235187	0.262266	0.793116	0.935304
FTL_0961	269.8278	0.459291	0.302413	1.518754	0.128824	0.50475
FTL_0962	163.4179	-0.10909	0.330336	-0.33024	0.741215	0.91279
FTL_0963	852.8863	-0.263	0.261	-1.00767	0.313614	0.664617
FTL_0964	3998.468	-0.74819	0.258688	-2.89225	0.003825	0.059598
FTL_0965	1229.068	-0.6538	0.305197	-2.14222	0.032176	0.239532
FTL_0966	752.2448	0.049767	0.260734	0.190874	0.848625	0.952754
FTL_0967	816.8328	-0.07071	0.252566	-0.27996	0.779508	0.928756
FTL_0968	5195.173	0.265863	0.24134	1.101612	0.27063	0.634024
FTL_0969	1006.981	0.434777	0.266653	1.630498	0.102996	0.45103
FTL_0970	187.3425	0.051376	0.322342	0.159383	0.873367	0.958064
FTL_0971	413.8756	0.274184	0.276285	0.992396	0.321004	0.675622
FTL_0972	254.1106	0.253678	0.301274	0.842017	0.399778	0.745959
FTL_0973	259.4356	0.084495	0.299188	0.282414	0.777626	0.927063
FTL_0974	821.7616	-0.18298	0.260807	-0.70159	0.482934	0.792187
FTL_0975	1103.577	-0.18819	0.265392	-0.70909	0.478267	0.789323
FTL_0976	938.3341	0.267922	0.259563	1.032204	0.301977	0.652888
FTL_0977	48.68099	0.140108	0.393827	0.355759	0.722021	0.905632
FTL_0978	42.37633	0.454578	0.396264	1.14716	0.251315	0.618781
FTL_0979	111.9806	0.45512	0.366084	1.243209	0.213791	0.587852
FTL_0980	301.2884	0.603018	0.31551	1.911248	0.055973	0.330196
FTL_0981	557.8552	0.141457	0.266516	0.530763	0.595583	0.845587
FTL_0982	550.7944	-0.29396	0.27057	-1.08645	0.277281	0.638231
FTL_0983	1367.175	0.097223	0.245269	0.396395	0.691814	0.892747
FTL_0984	7413.875	-0.37455	0.265003	-1.41336	0.157548	0.545987
FTL_0985	2031.089	-0.25494	0.260795	-0.97756	0.32829	0.681554
FTL_0986	8736.913	-0.15718	0.241212	-0.65161	0.514654	0.804396

FTL_0987	7436.997	-0.28298	0.240397	-1.17715	0.239137	0.609208
FTL_0988	514.7637	-0.18299	0.281318	-0.65048	0.515379	0.804446
FTL_0989	1273.338	-0.26943	0.243217	-1.10776	0.267965	0.632171
FTL_0990	697.6594	-0.19034	0.259272	-0.73413	0.462868	0.77919
FTL_0991	39.86487	0.346926	0.398825	0.869871	0.384371	0.734397
FTL_0992	1076.933	0.305636	0.253444	1.205933	0.227843	0.600217
FTL_0993	497.9585	0.028756	0.268813	0.106974	0.914809	0.97571
FTL_0994	3503.774	-0.16527	0.244589	-0.67571	0.499226	0.800092
FTL_0995	1826.073	-0.37753	0.246034	-1.53448	0.124911	0.49521
FTL_0996	736.3612	-0.22105	0.279277	-0.7915	0.428651	0.768588
FTL_0997	333.7578	0.768412	0.37518	2.048117	0.040549	0.274419
FTL_0998	238.0274	0.888016	0.332088	2.674036	0.007494	0.089135
FTL_0999	421.5321	-0.01858	0.326437	-0.05693	0.954603	0.991603
FTL_1000	612.7949	0.490903	0.265302	1.850355	0.064262	0.360803
FTL_1001	1097.959	0.389101	0.250215	1.555065	0.119931	0.483237
FTL_1002	180.8186	0.692879	0.349532	1.982304	0.047445	0.301788
FTL_1003	893.7237	-0.0484	0.255818	-0.18918	0.849951	0.952754
FTL_1004	1273.891	0.440521	0.252593	1.743998	0.081159	0.407826
FTL_1005	1700.652	0.086849	0.242138	0.358678	0.719836	0.905629
FTL_1006	749.8995	-0.18514	0.257667	-0.71854	0.472424	0.786405
FTL_1007	525.6563	-0.02298	0.283643	-0.08103	0.93542	0.982852
FTL_1008	151.4297	-0.16402	0.336166	-0.48793	0.625602	0.860048
FTL_1009	167.9807	0.455472	0.337716	1.348686	0.177438	0.569084
FTL_1010	68.85824	0.407287	0.378758	1.075323	0.28223	0.643901
FTL_1011	28.3785	0.464391	0.400714	1.15891	0.246493	0.617
FTL_1012	538.29	0.386786	0.271885	1.422611	0.154849	0.544009
FTL_1013	1097.54	0.004715	0.256593	0.018377	0.985338	0.995965
FTL_1014	1330.009	0.070924	0.260362	0.272404	0.785311	0.931254
FTL_1015	3454.375	-0.11513	0.279404	-0.41207	0.680291	0.884928
FTL_1016	4936.517	0.048794	0.242674	0.201068	0.840645	0.950146
FTL_1017	2967.298	0.244365	0.23184	1.054026	0.291871	0.643901
FTL_1018	2098.39	-0.07051	0.254962	-0.27653	0.78214	0.929687
FTL_1019	32.97034	-0.05094	0.400582	-0.12716	0.898812	0.969455
FTL_1020	1038.355	0.303859	0.254008	1.196259	0.231596	0.604483
FTL_1021	1150.884	0.168425	0.245764	0.685313	0.493146	0.79605
FTL_1022	2327.412	0.081627	0.237301	0.343981	0.730861	0.909053
FTL_1023	368.7536	0.427683	0.305783	1.39865	0.161918	0.553495
FTL_1024	13356.36	-0.14478	0.238746	-0.60644	0.544226	0.824572
FTL_1025	5193.458	-0.26964	0.260719	-1.03422	0.301031	0.652888
FTL_1026	8457.061	-0.38201	0.266917	-1.43118	0.152377	0.543047
FTL_1027	6591.179	-0.01967	0.235243	-0.08361	0.933364	0.982421
FTL_1028	3919.137	-0.16676	0.256756	-0.64948	0.516025	0.804663
FTL_1029	2991.683	-0.1875	0.24459	-0.76661	0.443316	0.775733

FTL_1030	3099.081	-0.28125	0.242175	-1.16133	0.245506	0.616834
FTL_1031	708.6574	-0.30798	0.261395	-1.17821	0.238713	0.609208
FTL_1032	402.6949	-0.25412	0.284049	-0.89462	0.370988	0.718388
FTL_1033	164.6627	0.053118	0.329833	0.161046	0.872057	0.958064
FTL_1034	1279.28	0.01017	0.249469	0.040767	0.967482	0.994309
FTL_1035	484.2993	-0.0429	0.270492	-0.15861	0.873977	0.958064
FTL_1036	235.6249	0.34896	0.313918	1.111626	0.266299	0.630978
FTL_1037	1330.385	0.076081	0.268863	0.282972	0.777198	0.927063
FTL_1038	903.0838	0.285581	0.259246	1.101583	0.270643	0.634024
FTL_1039	266.6648	0.531998	0.338914	1.569712	0.116482	0.477814
FTL_1040	794.9534	0.194983	0.258163	0.755274	0.450085	0.778339
FTL_1041	2391.719	-0.0605	0.23672	-0.25556	0.798289	0.937924
FTL_1042	3932.168	-0.14294	0.236506	-0.60436	0.545602	0.824572
FTL_1043	2581.777	-0.19152	0.239087	-0.80106	0.423096	0.762761
FTL_1044	1246.719	-0.06789	0.243862	-0.27839	0.780715	0.929092
FTL_1045	1839.117	-0.13634	0.261418	-0.52155	0.601985	0.848928
FTL_1046	7211.941	0.103034	0.240684	0.42809	0.668586	0.881666
FTL_1047	575.4513	0.778717	0.30421	2.559803	0.010473	0.107403
FTL_1048	2847.704	0.166101	0.250365	0.663435	0.507052	0.804313
FTL_1049	6005.155	0.016386	0.229476	0.071407	0.943074	0.986767
FTL_1050	9888.794	0.116393	0.245577	0.473957	0.635531	0.865459
FTL_1051	1922.162	-0.02239	0.257826	-0.08686	0.930783	0.982421
FTL_1052	207.1143	0.072124	0.319087	0.226034	0.821175	0.943654
FTL_1053	322.338	0.971148	0.34113	2.846855	0.004415	0.06431
FTL_1054	264.0211	0.559997	0.341315	1.640704	0.100859	0.448176
FTL_1055	352.73	-0.05051	0.283027	-0.17848	0.858346	0.954771
FTL_1056	98.79035	-0.13485	0.356267	-0.37851	0.705054	0.899212
FTL_1057	314.5729	-0.39036	0.29097	-1.34157	0.179737	0.569084
FTL_1058	1905.516	0.014963	0.24294	0.061593	0.950887	0.990816
FTL_1059	1469.107	-0.13786	0.261161	-0.52787	0.597588	0.846141
FTL_1060	8607.256	-0.0429	0.234101	-0.18326	0.854591	0.954733
FTL_1061	2492.437	0.014527	0.238726	0.060852	0.951477	0.990916
FTL_1062	1375.52	0.345892	0.2446	1.41411	0.15733	0.545987
FTL_1063	1211.197	0.110063	0.257524	0.427391	0.669095	0.881666
FTL_1064	2770.128	0.024489	0.244888	0.100001	0.920343	0.977743
FTL_1065	1515.842	-0.11002	0.266924	-0.41217	0.680216	0.884928
FTL_1066	897.2897	-0.06912	0.259728	-0.26614	0.790135	0.933121
FTL_1067	2549.239	-0.41666	0.264408	-1.57584	0.115064	0.475881
FTL_1068	526.7776	-0.52211	0.294869	-1.77066	0.076617	0.395437
FTL_1069	420.14	-0.35363	0.275332	-1.28436	0.199016	0.583892
FTL_1070	13.92569	0.095882	0.377632	0.253903	0.79957	0.937924
FTL_1071	4801.309	-0.23557	0.272117	-0.8657	0.386657	0.735977
FTL_1072	7400.867	-0.15797	0.279615	-0.56496	0.572104	0.832153

FTL_1073	1388.943	-0.11186	0.287448	-0.38914	0.697172	0.896555
FTL_1074	2306.707	0.130189	0.234855	0.554338	0.579347	0.834161
FTL_1075	3921.615	-0.04249	0.232494	-0.18276	0.854984	0.954733
FTL_1076	905.8479	-0.10735	0.25478	-0.42133	0.673515	0.881992
FTL_1077	2289.43	-0.08566	0.234675	-0.36504	0.715085	0.904215
FTL_1078	637.3297	0.225171	0.289159	0.778708	0.436152	0.771103
FTL_1079	69.32893	-0.35837	0.379018	-0.94552	0.344394	0.699931
FTL_1080	125.3172	-0.43513	0.347316	-1.25282	0.21027	0.587629
FTL_1081	34.47274	-0.35729	0.400565	-0.89196	0.372413	0.720453
FTL_1082	15.49332	0.247714	0.382286	0.64798	0.516998	0.805555
FTL_1083	65.0324	-0.45495	0.380536	-1.19556	0.231869	0.604483
FTL_1084	78.88915	0.29973	0.385333	0.777846	0.43666	0.771103
FTL_1085	78.3085	0.283543	0.388238	0.730331	0.465188	0.77919
FTL_1086	441.7028	-0.11122	0.278108	-0.39993	0.68921	0.892023
FTL_1087	391.1794	0.505583	0.325302	1.554196	0.120138	0.483237
FTL_1088	1570.887	0.012449	0.24313	0.051204	0.959163	0.992291
FTL_1089	1550.962	-0.34954	0.260743	-1.34054	0.18007	0.569084
FTL_1090	696.0243	0.173085	0.262543	0.659262	0.509728	0.804396
FTL_1091	86.50878	-0.12229	0.368932	-0.33148	0.740282	0.91279
FTL_1092	482.1974	0.04751	0.289797	0.163942	0.869777	0.958064
FTL_1093	2155.634	-0.39089	0.240634	-1.62442	0.104287	0.451804
FTL_1094	1905.804	0.002441	0.240451	0.010153	0.991899	0.996535
FTL_1095	340.3014	-0.51151	0.302693	-1.68985	0.091056	0.434915
FTL_1096	6252.451	-0.44534	0.260966	-1.7065	0.087914	0.428901
FTL_1097	1742.155	-0.47526	0.276723	-1.71744	0.085898	0.423959
FTL_1098	459.5159	0.379752	0.315129	1.20507	0.228176	0.600307
FTL_1099	1610.322	0.070139	0.241797	0.290072	0.771761	0.922259
FTL_1100	2233.522	-0.19385	0.251007	-0.77231	0.439931	0.77402
FTL_1101	1137.893	0.683399	0.27234	2.509362	0.012095	0.118249
FTL_1102	724.7857	0.447946	0.256369	1.747273	0.08059	0.406893
FTL_1103	289.5438	0.606972	0.296759	2.045338	0.040822	0.274419
FTL_1104	600.5384	0.863249	0.343382	2.513964	0.011938	0.117943
FTL_1105	2185.331	0.15192	0.23521	0.64589	0.51835	0.805788
FTL_1106	7737.515	0.17479	0.231405	0.755341	0.450044	0.778339
FTL_1107	2572.255	-0.03797	0.257475	-0.14746	0.882767	0.96328
FTL_1108	2898.63	-0.40827	0.272094	-1.50049	0.133487	0.509395
FTL_1109	3368.348	-0.24016	0.239984	-1.00073	0.316957	0.669205
FTL_1110	1252.311	-0.19666	0.246913	-0.79647	0.425761	0.766827
FTL_1111	294.6899	-0.03408	0.297603	-0.11452	0.908823	0.972447
FTL_1112	282.7292	0.36908	0.308106	1.197898	0.230957	0.604483
FTL_1113	218.1328	-0.02686	0.312218	-0.08602	0.931448	0.982421
FTL_1114	289.0131	0.198149	0.297178	0.666767	0.504921	0.802678
FTL_1115	1631.049	0.024052	0.245323	0.098043	0.921898	0.977806

FTL_1116	1770.595	0.121569	0.256164	0.474575	0.63509	0.865445
FTL_1117	1999.017	0.197653	0.239335	0.825842	0.408893	0.752409
FTL_1118	2974.806	0.02881	0.245878	0.11717	0.906725	0.972009
FTL_1119	1673.437	0.034006	0.245715	0.138396	0.889928	0.967119
FTL_1120	550.8935	-0.12769	0.269968	-0.47298	0.636228	0.865822
FTL_1121	293.2398	-0.17149	0.307284	-0.55808	0.576793	0.833908
FTL_1122	166.8781	0.116606	0.336587	0.346438	0.729014	0.90788
FTL_1123	106.4629	0.209678	0.359707	0.582914	0.559951	0.828816
FTL_1124	90.90156	0.405987	0.385345	1.053568	0.292081	0.643901
FTL_1125	185.649	-0.05705	0.33524	-0.17018	0.864869	0.956208
FTL_1126	57.97782	0.168697	0.392766	0.429511	0.667551	0.881666
FTL_1127	947.8417	0.455919	0.274307	1.662076	0.096498	0.445547
FTL_1128	197.3986	0.214819	0.325038	0.660906	0.508673	0.804396
FTL_1129	1660.942	-0.07478	0.246352	-0.30356	0.761467	0.919801
FTL_1130	976.1016	-0.02721	0.252615	-0.10771	0.914229	0.97571
FTL_1131	1137.887	0.15892	0.257542	0.617065	0.537192	0.821732
FTL_1132	3159.689	-0.17571	0.249696	-0.70371	0.481616	0.79199
FTL_1133	996.3733	-0.22813	0.296587	-0.76918	0.441784	0.775534
FTL_1134	4881.108	-0.27032	0.247752	-1.0911	0.275229	0.638231
FTL_1135	1595.752	0.28164	0.255144	1.103849	0.269659	0.633935
FTL_1136	6119.037	-0.07358	0.251762	-0.29224	0.770102	0.92177
FTL_1137	46667.64	-0.3757	0.274731	-1.36753	0.17146	0.564051
FTL_1138	19172.49	-0.44336	0.258904	-1.71246	0.086812	0.425592
FTL_1139	15110.02	0.105686	0.246856	0.428129	0.668557	0.881666
FTL_1140	18066.11	0.059385	0.256828	0.231223	0.817141	0.940321
FTL_1141	8612.188	-0.09736	0.287145	-0.33906	0.734568	0.911008
FTL_1142	20850.51	-0.1483	0.257101	-0.57682	0.564063	0.829541
FTL_1143	9324.58	-0.16164	0.270446	-0.59767	0.550058	0.824572
FTL_1144	5624.625	-0.17841	0.243403	-0.73296	0.46358	0.77919
FTL_1145	9078.619	-0.44524	0.277218	-1.6061	0.108252	0.463936
FTL_1146	4725.687	-0.90818	0.297258	-3.0552	0.002249	0.039865
FTL_1147	5617.99	-0.82244	0.259138	-3.17375	0.001505	0.030247
FTL_1148	7422.075	-0.66073	0.241175	-2.73962	0.006151	0.077273
FTL_1149	3586.098	-0.79752	0.255179	-3.12533	0.001776	0.033824
FTL_1150	482.5301	0.100668	0.299287	0.336359	0.7366	0.911338
FTL_1151	175.9738	0.271987	0.320305	0.849149	0.395798	0.74145
FTL_1152	32.81515	0.18459	0.400503	0.460895	0.644874	0.872239
FTL_1153	1031.8	0.29049	0.254381	1.14195	0.253475	0.620566
FTL_1154	135.7252	0.618849	0.374684	1.651656	0.098605	0.446841
FTL_1155	139.744	0.563355	0.352801	1.596806	0.110309	0.466602
FTL_1156	23.70616	-0.01026	0.398731	-0.02573	0.979469	0.994309
FTL_1157	1329.711	-3.32054	0.252467	-13.1524	1.65E-39	1.10E-36
FTL_1158	8386.004	-2.19196	0.237128	-9.24375	2.38E-20	5.98E-18

FTL_1159	5400.34	-1.64977	0.229297	-7.19489	6.25E-13	9.67E-11
FTL_1160	4025.139	-1.49529	0.238301	-6.2748	3.50E-10	4.69E-08
FTL_1161	775.4876	-1.20401	0.27094	-4.44382	8.84E-06	0.000444
FTL_1162	4441.558	-1.07054	0.268154	-3.99225	6.54E-05	0.00253
FTL_1163	546.3502	-0.808	0.312227	-2.58786	0.009657	0.101684
FTL_1164	1332.791	-1.03353	0.298449	-3.46299	0.000534	0.013766
FTL_1165	1095.822	-0.7711	0.318696	-2.41955	0.01554	0.141978
FTL_1166	1455.947	-1.45003	0.253558	-5.71873	1.07E-08	1.08E-06
FTL_1167	1059.159	-2.11588	0.266297	-7.94558	1.93E-15	3.88E-13
FTL_1168	588.4174	-1.0108	0.27835	-3.6314	0.000282	0.008853
FTL_1169	236.9564	-0.22197	0.351198	-0.63204	0.527364	0.812401
FTL_1170	283.8096	-1.11583	0.317637	-3.51292	0.000443	0.012203
FTL_1171	1953.657	-1.54704	0.262647	-5.89017	3.86E-09	4.31E-07
FTL_1172	2162.746	-2.41671	0.24154	-10.0055	1.44E-23	4.83E-21
FTL_1173	2512.778	-0.00348	0.245025	-0.0142	0.98867	0.996358
FTL_1174	2229.821	-0.62139	0.238698	-2.60326	0.009234	0.099256
FTL_1175	242.8976	-0.16655	0.372015	-0.44769	0.654378	0.875699
FTL_1176	201.0035	-0.26424	0.348732	-0.75772	0.448616	0.77779
FTL_1177	3359.886	-0.28226	0.290643	-0.97117	0.331464	0.684969
FTL_1178	489.2424	0.069409	0.294202	0.235923	0.813492	0.939669
FTL_1179	3733.563	-0.015	0.252083	-0.05952	0.952541	0.991454
FTL_1180	3083.641	0.174274	0.233199	0.74732	0.454871	0.77919
FTL_1181	2890.296	0.093718	0.243463	0.384935	0.700286	0.897755
FTL_1182	1506.556	-0.13852	0.269284	-0.51438	0.606984	0.851082
FTL_1183	4687.914	0.274783	0.233156	1.178537	0.238582	0.609208
FTL_1184	4744.791	-0.11206	0.258498	-0.43351	0.664646	0.881666
FTL_1185	8554.135	-0.08954	0.265159	-0.33768	0.735605	0.911008
FTL_1186	14485.6	-0.14003	0.251499	-0.55677	0.577684	0.833908
FTL_1187	14064.4	-0.21354	0.258368	-0.8265	0.408521	0.752409
FTL_1188	2215.393	-0.1388	0.246408	-0.5633	0.573231	0.832153
FTL_1189	1768.843	0.145587	0.243611	0.597622	0.550092	0.824572
FTL_1190	2748.025	-0.17825	0.329905	-0.54031	0.588981	0.842174
FTL_1191	65285.97	-0.25982	0.263065	-0.98767	0.323312	0.677157
FTL_1192	21196.49	-0.37219	0.271648	-1.37011	0.170653	0.563239
FTL_1193	2460.947	-0.05364	0.258905	-0.20719	0.835862	0.948663
FTL_1194	129.0098	1.301234	0.377152	3.450154	0.00056	0.014255
FTL_1195	1045.734	0.28628	0.254543	1.124683	0.260724	0.628129
FTL_1196	207.0908	0.67853	0.329833	2.057196	0.039667	0.271195
FTL_1197	4175.686	0.071342	0.232009	0.307495	0.758466	0.919801
FTL_1198	8265.283	0.041765	0.254493	0.164112	0.869643	0.958064
FTL_1199	1645.116	0.135396	0.238659	0.56732	0.570496	0.832153
FTL_1200	306.5572	-0.07076	0.294163	-0.24054	0.809912	0.939549
FTL_1201	490.5141	0.26165	0.276607	0.945925	0.344187	0.699931

FTL_1202	687.2954	0.108346	0.262221	0.413186	0.67947	0.884928
FTL_1203	1637.538	0.151125	0.249836	0.604897	0.545248	0.824572
FTL_1204	869.496	0.091121	0.254628	0.35786	0.720448	0.905629
FTL_1205	90.97719	0.265831	0.385515	0.689548	0.490478	0.793769
FTL_1206	229.1756	0.058468	0.306512	0.190752	0.84872	0.952754
FTL_1207	2092.735	0.033893	0.25686	0.131953	0.895022	0.96895
FTL_1208	1637.13	-0.27655	0.264601	-1.04514	0.295956	0.644964
FTL_1209	1155.175	0.054839	0.248249	0.220903	0.825168	0.945294
FTL_1210	522.173	-0.22378	0.285911	-0.78269	0.433807	0.771103
FTL_1211	1393.77	-0.28409	0.288755	-0.98383	0.325197	0.678056
FTL_1212	12561.7	-0.22005	0.263247	-0.83591	0.403208	0.748883
FTL_1213	1507.592	-1.13049	0.247752	-4.563	5.04E-06	0.000274
FTL_1214	36.41898	0.293623	0.40083	0.732536	0.463841	0.77919
FTL_1215	998.1817	-0.31671	0.264698	-1.19649	0.231505	0.604483
FTL_1216	2782.86	-0.36582	0.245808	-1.48823	0.136689	0.514913
FTL_1217	1691.41	-1.39433	0.257722	-5.4102	6.3E-08	5.27E-06
FTL_1218	2616.357	-1.04289	0.313421	-3.32745	0.000876	0.020019
FTL_1219	6385.531	-3.43587	0.27566	-12.4642	1.17E-35	5.88E-33
FTL_1220	430.6434	0.054444	0.308011	0.176759	0.859698	0.954771
FTL_1221	409.3125	0.975342	0.349838	2.787984	0.005304	0.07203
FTL_1222	469.666	-0.01347	0.277553	-0.04853	0.96129	0.992291
FTL_1223	1137.238	-0.46757	0.24676	-1.89483	0.058115	0.337605
FTL_1224	2039.241	-0.87123	0.25646	-3.39712	0.000681	0.016693
FTL_1225	1982.362	-1.28127	0.260817	-4.91253	8.99E-07	6.23E-05
FTL_1226	396.6556	-0.36505	0.284998	-1.28089	0.200234	0.584137
FTL_1227	913.4803	-0.70005	0.267438	-2.61761	0.008855	0.097791
FTL_1228	1753.816	-0.63276	0.271629	-2.32949	0.019833	0.171914
FTL_1229	1137.528	-0.50053	0.258772	-1.93427	0.05308	0.324291
FTL_1230	4957.757	-0.08808	0.260349	-0.33832	0.735123	0.911008
FTL_1231	1142.667	0.199035	0.270414	0.736036	0.461709	0.77919
FTL_1232	4226.514	0.067528	0.229721	0.293959	0.768789	0.920898
FTL_1233	2978.647	-0.2134	0.238471	-0.89487	0.370859	0.718388
FTL_1234	350.7713	0.175884	0.301547	0.583273	0.55971	0.828816
FTL_1235	2791.696	0.438549	0.25955	1.689654	0.091094	0.434915
FTL_1236	1976.307	-0.24418	0.251421	-0.97119	0.331455	0.684969
FTL_1237	1691.695	0.039692	0.250645	0.158359	0.874174	0.958064
FTL_1238	204.3523	0.285604	0.322451	0.885726	0.375765	0.724149
FTL_1239	11837.1	-0.10677	0.241186	-0.4427	0.657983	0.87702
FTL_1240	11682.4	-0.27461	0.268354	-1.02332	0.306155	0.656746
FTL_1241	1708.069	0.072644	0.23894	0.304028	0.761106	0.919801
FTL_1242	404.2325	-0.51569	0.277296	-1.8597	0.062927	0.35646
FTL_1243	934.497	-0.92773	0.26555	-3.49363	0.000476	0.012943
FTL_1244	885.235	0.016951	0.26171	0.06477	0.948357	0.989719

FTL_1245	631.7399	-0.10636	0.275628	-0.38589	0.699577	0.897755
FTL_1246	524.7409	-0.02561	0.291625	-0.08781	0.930029	0.982322
FTL_1247	1091.121	-0.11779	0.250565	-0.47012	0.638272	0.866842
FTL_1248	2575.245	-0.16014	0.243954	-0.65643	0.51155	0.804396
FTL_1249	539.0139	-0.52864	0.316135	-1.67218	0.094488	0.443941
FTL_1250	203.1291	0.383095	0.327649	1.169225	0.242313	0.612533
FTL_1251	2648.378	-0.96481	0.274493	-3.51487	0.00044	0.012203
FTL_1252	2284.806	0.171021	0.279142	0.612668	0.540096	0.823043
FTL_1253	1016.399	0.446364	0.25513	1.749553	0.080195	0.406289
FTL_1254	211.6113	0.022905	0.311821	0.073455	0.941444	0.985574
FTL_1255	364.2765	0.235764	0.298217	0.790581	0.429189	0.768867
FTL_1256	758.5604	-0.33982	0.280592	-1.21109	0.22586	0.596555
FTL_1257	561.7546	-0.20798	0.269629	-0.77135	0.440499	0.77402
FTL_1258	2124.786	-0.08277	0.236092	-0.35058	0.725903	0.907275
FTL_1259	538.5234	0.255971	0.271849	0.941592	0.346401	0.701515
FTL_1260	327.2259	0.609264	0.316852	1.922864	0.054497	0.326617
FTL_1261	532.7239	-0.35949	0.273634	-1.31376	0.188926	0.575235
FTL_1262	2430.242	-0.08392	0.238808	-0.35142	0.725276	0.907159
FTL_1263	1108.339	0.260148	0.24753	1.050974	0.29327	0.643901
FTL_1264	836.0506	0.744162	0.296123	2.513017	0.01197	0.117943
FTL_1265	6068.959	0.528822	0.261307	2.023759	0.042995	0.285077
FTL_1266	7643.615	-0.38904	0.240483	-1.61775	0.105716	0.454038
FTL_1267	2758.368	-0.57709	0.271896	-2.12247	0.033798	0.245315
FTL_1268	214.376	0.098799	0.316908	0.311759	0.755223	0.919801
FTL_1269	337.6073	0.203304	0.302678	0.671685	0.501784	0.800092
FTL_1270	530.5062	0.345806	0.278844	1.240143	0.214923	0.588548
FTL_1271	2938.489	0.335414	0.243705	1.376313	0.168725	0.563239
FTL_1272	8234.603	-0.17271	0.263635	-0.65509	0.512408	0.804396
FTL_1273	9289.456	-0.13012	0.269148	-0.48344	0.628782	0.861561
FTL_1274	4291.86	-0.20048	0.261655	-0.76619	0.443561	0.775733
FTL_1275	2965.054	-0.60261	0.279974	-2.15238	0.031368	0.237025
FTL_1276	890.1537	-0.19005	0.250579	-0.75842	0.448197	0.77779
FTL_1277	753.1046	0.483175	0.260809	1.852602	0.063939	0.359995
FTL_1278	475.5652	0.373268	0.27448	1.359909	0.173859	0.565576
FTL_1279	437.4066	0.175827	0.279176	0.629806	0.528822	0.812401
FTL_1280	83.74869	0.11122	0.37161	0.299293	0.764717	0.920636
FTL_1281	212.7929	-0.0013	0.325365	-0.00399	0.996816	0.998306
FTL_1282	1461.433	0.043432	0.252394	0.17208	0.863375	0.956083
FTL_1283	4295.476	-0.17089	0.242715	-0.70406	0.481394	0.79199
FTL_1284	3152.407	-0.20465	0.253757	-0.80648	0.419964	0.761848
FTL_1285	4259.507	0.343704	0.242822	1.415456	0.156935	0.545742
FTL_1286	1086.656	0.108924	0.269025	0.404886	0.685561	0.889018
FTL_1287	5338.841	0.001497	0.286609	0.005225	0.995831	0.997817

FTL_1288	380.3215	-0.09072	0.295619	-0.30688	0.758935	0.919801
FTL_1289	151.1531	0.972012	0.347973	2.79335	0.005217	0.071328
FTL_1290	906.1208	0.332716	0.258706	1.286076	0.198417	0.583892
FTL_1291	69.68186	0.365739	0.37621	0.972168	0.330967	0.684969
FTL_1292	71.89298	1.044845	0.377616	2.76695	0.005658	0.074176
FTL_1293	1935.734	0.351635	0.253554	1.386822	0.165496	0.557198
FTL_1294	1797.622	0.293049	0.278835	1.050974	0.293271	0.643901
FTL_1295	818.6	0.170598	0.256863	0.664159	0.506589	0.8043
FTL_1296	574.8686	0.225077	0.267474	0.841494	0.400071	0.745959
FTL_1297	141.4821	0.718253	0.334311	2.148456	0.031678	0.237581
FTL_1298	1067.765	0.298503	0.25376	1.176323	0.239466	0.609274
FTL_1299	276.2401	0.122316	0.308729	0.396193	0.691963	0.892747
FTL_1300	170.613	0.255171	0.325426	0.784115	0.432973	0.771103
FTL_1301	202.8528	0.453967	0.328251	1.382988	0.166668	0.560207
FTL_1302	2059.42	-0.05978	0.237089	-0.25215	0.800922	0.937924
FTL_1303	6938.179	0.193432	0.252613	0.765724	0.44384	0.775733
FTL_1304	3348.624	-0.13974	0.240496	-0.58106	0.561203	0.828816
FTL_1305	1425.481	-0.0967	0.257439	-0.37563	0.707193	0.900705
FTL_1306	3535.062	-0.47869	0.264984	-1.80648	0.070844	0.378915
FTL_1307	595.0662	-0.03488	0.299565	-0.11644	0.9073	0.972107
FTL_1308	8137.123	0.432493	0.232261	1.862101	0.062589	0.356384
FTL_1309	11694.65	-0.12763	0.240818	-0.52999	0.596118	0.845587
FTL_1310	4115.735	-0.18851	0.247602	-0.76132	0.446463	0.776858
FTL_1311	6599.778	-0.02609	0.230264	-0.11329	0.909803	0.972715
FTL_1312	462.7862	-0.93848	0.28035	-3.34752	0.000815	0.018838
FTL_1313	473.4338	-0.84439	0.290189	-2.90978	0.003617	0.057699
FTL_1314	209.715	0.169784	0.3103	0.547161	0.584268	0.838244
FTL_1315	122.3077	-1.26932	0.353357	-3.59218	0.000328	0.009987
FTL_1316	1088.925	0.282939	0.255044	1.109372	0.26727	0.631272
FTL_1317	5335.804	-0.66647	0.234033	-2.84777	0.004403	0.06431
FTL_1318	66.77052	0.437678	0.378357	1.156785	0.24736	0.617632
FTL_1319	187.2961	0.395455	0.319689	1.237	0.216087	0.590864
FTL_1320	417.8983	0.110998	0.312773	0.354882	0.722678	0.905632
FTL_1321	139.1712	1.006912	0.386571	2.604726	0.009195	0.099256
FTL_1322	80.37751	0.310197	0.373189	0.831206	0.405857	0.749791
FTL_1323	162.5227	0.356333	0.331262	1.075682	0.282069	0.643901
FTL_1324	30.48553	0.31466	0.400918	0.784849	0.432542	0.771103
FTL_1325	301.7455	0.501003	0.306904	1.63244	0.102587	0.450217
FTL_1326	320.7788	-0.00567	0.288444	-0.01966	0.984312	0.995965
FTL_1327	1216.39	-0.22193	0.245888	-0.90257	0.366755	0.713628
FTL_1328	25625.02	-0.40032	0.252523	-1.5853	0.112898	0.471999
FTL_1329	711.5079	-0.42243	0.291654	-1.4484	0.147505	0.53517
FTL_1330	1942.858	-0.0285	0.249068	-0.11441	0.908915	0.972447

FTL_1331	2264.047	0.047724	0.235468	0.202677	0.839388	0.949983
FTL_1332	1451.69	0.145023	0.244392	0.593405	0.55291	0.825669
FTL_1333	3158.498	0.055861	0.240449	0.232318	0.816291	0.940255
FTL_1334	3150.341	-0.00647	0.240161	-0.02692	0.978521	0.994309
FTL_1335	1408.783	-0.04114	0.251041	-0.16387	0.869832	0.958064
FTL_1336	1970.408	-0.33138	0.248163	-1.33531	0.181773	0.571775
FTL_1337	1180.36	-0.29796	0.274265	-1.08639	0.277306	0.638231
FTL_1338	1234.651	-0.19593	0.244509	-0.80132	0.422947	0.762761
FTL_1339	1519.138	-0.16697	0.241539	-0.69127	0.489399	0.793769
FTL_1340	1279.704	-0.3662	0.249075	-1.47023	0.141501	0.523071
FTL_1341	407.1977	-0.47557	0.28825	-1.64984	0.098975	0.446841
FTL_1342	774.0046	0.093223	0.254338	0.366529	0.71397	0.903815
FTL_1343	414.2804	0.295002	0.285968	1.031593	0.302263	0.652888
FTL_1344	294.3089	0.090296	0.293367	0.307792	0.758241	0.919801
FTL_1345	1288.446	-0.3606	0.275446	-1.30916	0.19048	0.575235
FTL_1346	293.027	-0.17451	0.291751	-0.59814	0.549747	0.824572
FTL_1347	82.47625	0.239391	0.375845	0.636942	0.524163	0.812401
FTL_1348	247.488	0.448238	0.314321	1.426053	0.153853	0.543488
FTL_1349	943.971	0.275297	0.257064	1.070928	0.284202	0.643901
FTL_1350	5435.884	-0.17398	0.245184	-0.70959	0.477961	0.789323
FTL_1351	294.4107	-0.48422	0.291761	-1.65966	0.096983	0.446076
FTL_1352	554.0664	0.167276	0.288915	0.578979	0.562604	0.829057
FTL_1353	71.88795	0.201694	0.382439	0.527388	0.597924	0.846141
FTL_1354	1627.813	-0.15094	0.251459	-0.60027	0.548324	0.824572
FTL_1355	1313.494	0.31261	0.245548	1.273111	0.202979	0.585626
FTL_1356	1324.43	-0.07749	0.250566	-0.30924	0.757137	0.919801
FTL_1357	1118.171	0.082114	0.247894	0.331247	0.740458	0.91279
FTL_1358	3494.316	0.301987	0.234196	1.289464	0.197237	0.583892
FTL_1359	275.8013	0.133033	0.294598	0.451573	0.651576	0.874878
FTL_1360	479.6873	-0.28837	0.27496	-1.04878	0.29428	0.643956
FTL_1361	3041.928	-0.03036	0.31126	-0.09755	0.922291	0.977806
FTL_1362	2857.699	0.235695	0.247563	0.952059	0.341067	0.697731
FTL_1363	1452.83	0.113429	0.250138	0.453467	0.650212	0.874878
FTL_1364	4567.908	-0.25464	0.229326	-1.11039	0.266831	0.630978
FTL_1365	2651.452	0.445877	0.245714	1.814616	0.069583	0.378915
FTL_1366	1428.143	0.059149	0.243658	0.242753	0.808197	0.939549
FTL_1367	1113.264	-0.13481	0.253221	-0.53239	0.594457	0.845587
FTL_1368	710.9214	-0.27484	0.2723	-1.00934	0.312811	0.663939
FTL_1369	971.0341	0.242214	0.255496	0.948012	0.343123	0.698762
FTL_1370	1196.406	0.298807	0.246284	1.213261	0.22503	0.596453
FTL_1371	1253.193	-0.30602	0.27558	-1.11046	0.2668	0.630978
FTL_1372	4180.604	-1.05148	0.238517	-4.40841	1.04E-05	0.00051
FTL_1373	72.96617	-0.21831	0.37649	-0.57986	0.562008	0.829057

FTL_1374	1849.505	-0.02295	0.241355	-0.09507	0.924255	0.979311
FTL_1375	197.3567	-0.38296	0.313445	-1.22179	0.221787	0.593974
FTL_1376	583.0331	0.16186	0.263848	0.61346	0.539572	0.822868
FTL_1377	203.6582	0.015808	0.311747	0.050708	0.959558	0.992291
FTL_1378	62.18405	0.192896	0.385453	0.500438	0.616766	0.856147
FTL_1379	316.7535	0.248777	0.302275	0.823017	0.410499	0.752775
FTL_1380	70.10203	0.050237	0.38672	0.129906	0.896641	0.96895
FTL_1381	172.5558	-0.04095	0.339153	-0.12074	0.903894	0.970527
FTL_1382	466.3397	-0.38117	0.329224	-1.15777	0.246959	0.617397
FTL_1383	754.189	-0.23667	0.268296	-0.88212	0.37771	0.726505
FTL_1384	395.5935	-0.08279	0.281112	-0.29449	0.76838	0.920898
FTL_1385	334.5285	0.658083	0.315515	2.085745	0.037002	0.262546
FTL_1386	296.3703	0.14246	0.311234	0.457725	0.64715	0.873587
FTL_1387	405.0109	0.030467	0.285709	0.106636	0.915078	0.97571
FTL_1388	2834.48	-0.33929	0.262855	-1.29079	0.196776	0.583892
FTL_1389	4789.73	-0.05967	0.243579	-0.24496	0.80649	0.939549
FTL_1390	7348.175	-0.21435	0.241188	-0.88872	0.374155	0.721739
FTL_1391	3457.332	-0.03871	0.230745	-0.16776	0.866775	0.957789
FTL_1392	20263.05	-0.26103	0.247723	-1.0537	0.29202	0.643901
FTL_1393	22247.08	0.300055	0.243498	1.232267	0.217849	0.592527
FTL_1394	3876.914	-0.72122	0.250989	-2.87352	0.004059	0.061477
FTL_1395	2539.107	-0.76103	0.247105	-3.07979	0.002071	0.038198
FTL_1396	4479.593	-0.15185	0.231176	-0.65685	0.511275	0.804396
FTL_1397	5313.234	-0.07228	0.232645	-0.31067	0.75605	0.919801
FTL_1398	1937.824	0.177001	0.263619	0.671427	0.501948	0.800092
FTL_1399	1248.284	-0.31911	0.273256	-1.16782	0.24288	0.612533
FTL_1400	610.4127	-0.35728	0.286497	-1.24707	0.212371	0.587629
FTL_1401	121.6298	0.187239	0.363683	0.51484	0.606665	0.851082
FTL_1402	987.9552	0.132194	0.259204	0.510001	0.610051	0.851082
FTL_1403	1842.399	0.265503	0.270923	0.979995	0.327089	0.680588
FTL_1404	3929.596	-0.23258	0.246314	-0.94423	0.34505	0.700557
FTL_1405	4239.398	-0.45175	0.267713	-1.68742	0.091522	0.435922
FTL_1406	22369.44	-0.27344	0.240189	-1.13843	0.254942	0.622641
FTL_1407	22428.76	-0.10288	0.227839	-0.45156	0.651588	0.874878
FTL_1408	983.208	-0.10721	0.251527	-0.42625	0.669926	0.881666
FTL_1409	1340.176	-0.53456	0.26322	-2.03086	0.04227	0.282265
FTL_1410	1102.77	-0.14153	0.25442	-0.5563	0.578007	0.833908
FTL_1411	894.16	-0.59502	0.275989	-2.15597	0.031086	0.235785
FTL_1412	2144.512	-0.56811	0.257955	-2.20238	0.027639	0.218715
FTL_1413	2058.947	-4.56389	0.256356	-17.8029	6.71E-71	1.35E-67
FTL_1414	5077.116	-0.08521	0.22994	-0.3706	0.710938	0.902349
FTL_1415	2037.525	-0.08282	0.241323	-0.34321	0.731443	0.909215
FTL_1416	3272.571	0.132631	0.235242	0.563805	0.572887	0.832153

FTL_1417	4513.48	0.697835	0.319528	2.183953	0.028966	0.227426
FTL_1418	1707.265	-0.11557	0.246679	-0.46851	0.639422	0.867785
FTL_1419	5103.766	-0.04239	0.245829	-0.17245	0.863087	0.956083
FTL_1420	1204.944	-0.68293	0.255118	-2.67691	0.007431	0.088901
FTL_1421	590.2938	-0.22149	0.299953	-0.73842	0.460256	0.77919
FTL_1422	629.0581	-0.19413	0.293809	-0.66074	0.508781	0.804396
FTL_1423	842.5967	0.008064	0.290515	0.027757	0.977856	0.994309
FTL_1424	1015.199	-0.11494	0.272679	-0.42152	0.673376	0.881992
FTL_1425	1197.151	0.102965	0.268241	0.383853	0.701087	0.897755
FTL_1426	1466.439	-0.1498	0.251598	-0.59538	0.551592	0.825132
FTL_1427	2399.142	-0.27534	0.256443	-1.0737	0.282956	0.643901
FTL_1428	1214.448	-0.04011	0.2484	-0.16147	0.871723	0.958064
FTL_1429	1242.492	-0.27628	0.245834	-1.12386	0.261072	0.628129
FTL_1430	2115.504	-0.47627	0.259074	-1.83837	0.066008	0.368545
FTL_1431	1419.204	-0.00441	0.244724	-0.01803	0.985618	0.995965
FTL_1432	2226.023	0.330535	0.25349	1.303938	0.192255	0.575905
FTL_1433	1699.078	0.017683	0.263452	0.067121	0.946485	0.989719
FTL_1434	810.6607	0.239356	0.307197	0.77916	0.435885	0.771103
FTL_1435	43.54104	0.596482	0.400615	1.488913	0.13651	0.514913
FTL_1436	21.02248	0.2529	0.391365	0.646199	0.51815	0.805788
FTL_1437	39.8425	0.133733	0.397869	0.336123	0.736778	0.911338
FTL_1438	13.56543	0.113635	0.372286	0.305235	0.760187	0.919801
FTL_1439	44.53063	0.26905	0.396688	0.678242	0.497618	0.800092
FTL_1440	76.02196	0.351909	0.383617	0.917344	0.358962	0.711552
FTL_1441	500.3887	0.267635	0.281646	0.950254	0.341983	0.698563
FTL_1442	5147.881	-0.56078	0.243874	-2.29945	0.021479	0.183559
FTL_1443	1705.148	-0.08526	0.244366	-0.34889	0.727174	0.907275
FTL_1444	799.8559	-0.24947	0.258228	-0.96608	0.334006	0.687156
FTL_1445	276.2696	0.428957	0.308799	1.389113	0.164798	0.556713
FTL_1446	910.1449	0.299503	0.259218	1.15541	0.247923	0.618143
FTL_1447	236.7486	0.092866	0.309379	0.300171	0.764047	0.920636
FTL_1448	890.383	-0.20366	0.252887	-0.80532	0.420634	0.762375
FTL_1449	758.0959	-0.00255	0.256364	-0.00994	0.992073	0.996535
FTL_1450	2994.021	-0.11873	0.243613	-0.48737	0.625995	0.860048
FTL_1451	1134.786	-0.20338	0.246613	-0.82469	0.40955	0.752409
FTL_1452	4289.754	-0.16193	0.256753	-0.63067	0.528259	0.812401
FTL_1453	6094.953	-0.15412	0.260049	-0.59265	0.553414	0.825807
FTL_1454	204.8381	-0.04455	0.326057	-0.13663	0.89132	0.967361
FTL_1455	341.3333	0.173209	0.290092	0.597081	0.550453	0.824572
FTL_1456	252.6459	-0.03654	0.30249	-0.12079	0.903858	0.970527
FTL_1457	177.518	0.027722	0.332445	0.083387	0.933544	0.982421
FTL_1458	12008.91	-0.11993	0.26173	-0.45822	0.646796	0.873587
FTL_1459	951.3093	-0.02323	0.253455	-0.09166	0.926972	0.980638

FTL_1460	1501.347	0.309421	0.261763	1.182064	0.23718	0.609208
FTL_1461	8660.043	-0.14179	0.237428	-0.59717	0.550391	0.824572
FTL_1462	1176.553	0.132081	0.250916	0.526395	0.598614	0.846141
FTL_1463	200.0624	0.495946	0.32824	1.510922	0.130808	0.507288
FTL_1464	4029.176	0.055018	0.234385	0.234732	0.814417	0.939669
FTL_1465	103.4428	-0.17539	0.358877	-0.48873	0.625036	0.860048
FTL_1466	271.7125	0.312587	0.29753	1.050606	0.29344	0.643901
FTL_1467	203.1197	0.496257	0.323972	1.531791	0.125574	0.496858
FTL_1468	4028.982	0.055147	0.234379	0.235288	0.813985	0.939669
FTL_1469	103.4428	-0.17539	0.358877	-0.48873	0.625036	0.860048
FTL_1470	271.7125	0.312587	0.29753	1.050606	0.29344	0.643901
FTL_1471	207.0684	0.410153	0.328904	1.247027	0.212388	0.587629
FTL_1472	438.6303	0.173026	0.293681	0.589163	0.555752	0.82684
FTL_1473	8103.217	-0.31753	0.258445	-1.2286	0.21922	0.592692
FTL_1474	3598.158	-0.18014	0.233564	-0.77129	0.440537	0.77402
FTL_1475	2511.433	-0.07762	0.235165	-0.33008	0.741336	0.91279
FTL_1476	3660.854	-0.46612	0.254692	-1.83012	0.067232	0.371978
FTL_1477	932.4066	0.04206	0.252289	0.166713	0.867596	0.958064
FTL_1478	13477.82	0.10209	0.239495	0.426272	0.66991	0.881666
FTL_1479	7462.352	-0.3059	0.242961	-1.25904	0.208015	0.586556
FTL_1480	2120.138	0.184249	0.252093	0.730877	0.464854	0.77919
FTL_1481	1426.612	-0.21868	0.274855	-0.79561	0.42626	0.767039
FTL_1482	2038.97	-0.43083	0.274242	-1.57099	0.116185	0.477814
FTL_1483	1788.753	-0.47959	0.268863	-1.78379	0.074458	0.389743
FTL_1484	696.6779	0.077444	0.260016	0.297844	0.765823	0.920636
FTL_1485	1068.329	0.172182	0.272985	0.630739	0.528211	0.812401
FTL_1486	1614.361	0.297638	0.273248	1.089262	0.276039	0.638231
FTL_1487	1886.35	-0.17958	0.244115	-0.73564	0.46195	0.77919
FTL_1488	1718.473	0.68614	0.345364	1.986717	0.046954	0.29961
FTL_1489	7800.894	-0.04821	0.241262	-0.19984	0.84161	0.950357
FTL_1490	6292.595	-0.44014	0.255821	-1.7205	0.085341	0.422501
FTL_1491	5275.467	-0.39784	0.25847	-1.53922	0.123751	0.492554
FTL_1492	1591.93	-0.32769	0.248577	-1.31828	0.187411	0.575235
FTL_1493	1232.519	0.124536	0.249325	0.499494	0.617432	0.856479
FTL_1494	1272.084	-0.16619	0.26484	-0.62751	0.530324	0.813082
FTL_1495	1479.026	0.065335	0.260404	0.2509	0.801892	0.937924
FTL_1496	1316.739	0.094541	0.259321	0.364571	0.715432	0.904215
FTL_1497	1015.61	0.354921	0.273314	1.298583	0.194087	0.579666
FTL_1498	775.3859	-0.14436	0.254688	-0.56681	0.570844	0.832153
FTL_1499	1738.496	0.028089	0.238153	0.117944	0.906112	0.97187
FTL_1500	192.4934	0.488086	0.328504	1.485782	0.137337	0.515976
FTL_1501	23.61798	0.211901	0.398593	0.531623	0.594987	0.845587
FTL_1502	398.7346	0.141925	0.321153	0.441922	0.658546	0.877188

FTL_1503	1804.909	-0.70345	0.238866	-2.94497	0.00323	0.053213
FTL_1504	22133.14	-0.24406	0.284455	-0.85799	0.390896	0.737055
FTL_1505	895.6118	0.303475	0.259432	1.169767	0.242095	0.612533
FTL_1506	158.9079	-0.63164	0.350054	-1.80441	0.071167	0.378915
FTL_1507	157.9371	-0.84832	0.34966	-2.42614	0.015261	0.141336
FTL_1508	105.9702	-0.23138	0.355791	-0.65032	0.515486	0.804446
FTL_1509	556.5976	-0.90201	0.27472	-3.28338	0.001026	0.022409
FTL_1510	2994.094	-0.7062	0.269356	-2.62179	0.008747	0.097673
FTL_1511	4973.91	-0.54184	0.246466	-2.19845	0.027917	0.220055
FTL_1512	567.7865	0.285157	0.283091	1.007297	0.313792	0.664617
FTL_1513	146.3831	-0.24887	0.339496	-0.73307	0.463519	0.77919
FTL_1514	46.78092	-0.19882	0.399167	-0.49809	0.618423	0.856671
FTL_1515	73.98113	0.143939	0.379481	0.379304	0.704462	0.899212
FTL_1516	190.0077	0.512719	0.331268	1.547747	0.121683	0.487218
FTL_1517	483.1171	0.633005	0.326166	1.940744	0.052289	0.320432
FTL_1518	1125.981	0.479909	0.249909	1.920336	0.054815	0.326617
FTL_1519	434.6712	0.010015	0.278438	0.03597	0.971307	0.994309
FTL_1520	1763.631	-0.23223	0.250557	-0.92687	0.353993	0.707282
FTL_1521	8896.079	-0.66836	0.277616	-2.4075	0.016062	0.146084
FTL_1522	2724.74	-0.94877	0.257901	-3.67882	0.000234	0.007609
FTL_1523	3022.858	-0.80113	0.24773	-3.2339	0.001221	0.025709
FTL_1524	1741.047	-0.50109	0.249006	-2.01237	0.044181	0.290208
FTL_1525	2010.006	-0.30108	0.268294	-1.1222	0.261779	0.628129
FTL_1526	1389.11	-0.02943	0.267545	-0.10999	0.912415	0.974989
FTL_1527	12300.22	-0.25617	0.245014	-1.04554	0.295772	0.644964
FTL_1528	2048.541	-0.16139	0.256238	-0.62985	0.528795	0.812401
FTL_1529	512.2364	0.285542	0.279423	1.0219	0.306828	0.657489
FTL_1530	1939.593	0.392948	0.239277	1.64223	0.100542	0.448176
FTL_1531	3790.102	0.004628	0.23758	0.019479	0.984459	0.995965
FTL_1532	1257.482	-0.0433	0.245092	-0.17667	0.859769	0.954771
FTL_1533	869.2159	-0.34214	0.274833	-1.24489	0.213171	0.587852
FTL_1534	2752.829	-0.36044	0.251846	-1.4312	0.152373	0.543047
FTL_1535	4777.182	-0.34308	0.256073	-1.33977	0.180321	0.569084
FTL_1536	1839.685	0.032398	0.247587	0.130857	0.895889	0.96895
FTL_1537	25048.04	-0.02987	0.227657	-0.13119	0.895629	0.96895
FTL_1538	4435.609	0.009038	0.242467	0.037276	0.970265	0.994309
FTL_1539	3278.279	-0.55342	0.267759	-2.06684	0.038749	0.268569
FTL_1540	1149.027	-0.34964	0.244869	-1.42789	0.153324	0.543488
FTL_1541	2210.852	-0.35018	0.259774	-1.34802	0.177653	0.569084
FTL_1542	8791.609	-0.06309	0.235533	-0.26788	0.788791	0.932418
FTL_1543	2138.063	-0.22242	0.25535	-0.87103	0.383738	0.733885
FTL_1544	1449.025	0.111456	0.244811	0.455273	0.648913	0.874792
FTL_1545	1968.197	-0.51272	0.247112	-2.07483	0.038002	0.265604

FTL_1546	1741.789	-0.67196	0.238997	-2.81157	0.00493	0.068814
FTL_1547	15058.5	-0.21334	0.233825	-0.91237	0.361572	0.712199
FTL_1548	1707.367	-0.083	0.255376	-0.32501	0.745177	0.913853
FTL_1549	1860.372	-0.20525	0.241723	-0.84913	0.395809	0.74145
FTL_1550	1887.756	-0.31739	0.243024	-1.30602	0.191546	0.575235
FTL_1551	152.0259	0.25852	0.330532	0.782131	0.434137	0.771103
FTL_1552	4749.237	-0.24132	0.238847	-1.01035	0.312327	0.663911
FTL_1553	10815.67	-0.37866	0.252923	-1.49713	0.134359	0.510512
FTL_1554	13190.71	-0.36159	0.246348	-1.4678	0.142159	0.523071
FTL_1555	1013.959	0.032991	0.263935	0.124998	0.900525	0.969558
FTL_1556	1040.424	-0.09311	0.252858	-0.36825	0.71269	0.90379
FTL_1557	416.3722	0.322994	0.298262	1.082917	0.278845	0.640547
FTL_1558	213.6094	0.178898	0.323245	0.553444	0.579959	0.834444
FTL_1559	230.2567	-0.09566	0.304654	-0.314	0.75352	0.919801
FTL_1560	101.0007	0.1682	0.369946	0.454661	0.649353	0.874798
FTL_1561	39.57454	0.721636	0.400325	1.802625	0.071447	0.378915
FTL_1562	191.9343	0.004028	0.336069	0.011985	0.990438	0.996492
FTL_1563	82.23933	0.158534	0.379398	0.417857	0.676052	0.883527
FTL_1564	73.86327	0.529483	0.379321	1.395869	0.162754	0.554467
FTL_1565	210.6459	0.117319	0.330616	0.354849	0.722703	0.905632
FTL_1566	377.8078	-0.06168	0.282428	-0.2184	0.827119	0.945294
FTL_1567	365.9855	0.114035	0.307889	0.370376	0.711102	0.902349
FTL_1568	163.0966	-0.24084	0.324522	-0.74214	0.458002	0.77919
FTL_1569	876.1701	-0.28934	0.263428	-1.09838	0.27204	0.636555
FTL_1570	1342.619	0.065769	0.251099	0.261925	0.793379	0.935304
FTL_1571	3794.96	-0.17239	0.256293	-0.67261	0.501194	0.800092
FTL_1572	203.1438	0.456486	0.334635	1.364131	0.172526	0.565338
FTL_1573	327.5566	0.315221	0.326049	0.966793	0.333648	0.687123
FTL_1574	211.1364	0.62962	0.358898	1.754315	0.079377	0.405972
FTL_1575	1082.187	0.263656	0.250285	1.053426	0.292146	0.643901
FTL_1576	1323.79	-0.38012	0.244603	-1.55402	0.12018	0.483237
FTL_1577	711.9552	-0.33174	0.25787	-1.28646	0.198283	0.583892
FTL_1578	730.225	-0.28944	0.261873	-1.10526	0.269046	0.633234
FTL_1579	5334.316	-0.65152	0.248344	-2.62345	0.008704	0.097673
FTL_1580	2437.63	0.154996	0.263812	0.587524	0.556852	0.827864
FTL_1581	1376.724	0.041228	0.259095	0.159122	0.873573	0.958064
FTL_1582	2454.607	0.257084	0.243621	1.055262	0.291306	0.643901
FTL_1583	3483.765	0.054772	0.24984	0.219229	0.826472	0.945294
FTL_1584	1816.014	0.036187	0.246904	0.146564	0.883476	0.963531
FTL_1585	3062.477	-0.08368	0.237897	-0.35173	0.725039	0.907159
FTL_1586	1068.18	-0.09586	0.24898	-0.38502	0.700221	0.897755
FTL_1587	1136.323	-0.51925	0.263121	-1.97342	0.048448	0.307194
FTL_1588	463.041	-0.03402	0.277836	-0.12244	0.902553	0.970429

FTL_1589	210.6706	0.335068	0.327063	1.024477	0.30561	0.656278
FTL_1590	3061.302	-0.16464	0.243709	-0.67554	0.499332	0.800092
FTL_1591	13342.08	-0.03105	0.226265	-0.13722	0.890855	0.967361
FTL_1592	4418.955	0.030274	0.233023	0.12992	0.896629	0.96895
FTL_1593	2010.872	0.046087	0.235774	0.19547	0.845025	0.951924
FTL_1594	1507.529	-0.14452	0.250624	-0.57666	0.56417	0.829541
FTL_1595	1201.307	-0.31345	0.25122	-1.24772	0.212132	0.587629
FTL_1596	2221.459	-0.08836	0.245188	-0.36038	0.718562	0.904956
FTL_1597	8124.958	0.04327	0.23865	0.181313	0.856122	0.954771
FTL_1598	7254.441	-0.24305	0.236201	-1.02901	0.303476	0.654492
FTL_1599	2440.003	0.122952	0.243886	0.504136	0.614166	0.854898
FTL_1600	469.2423	-0.20454	0.274283	-0.74571	0.45584	0.77919
FTL_1601	1324.283	-0.87046	0.277952	-3.13168	0.001738	0.033592
FTL_1602	5350.809	-0.74088	0.245151	-3.02212	0.00251	0.043872
FTL_1603	1124.126	-0.65825	0.26964	-2.4412	0.014638	0.137491
FTL_1604	2952.474	-0.53725	0.251808	-2.13356	0.032878	0.243641
FTL_1605	3794.418	-0.43088	0.240756	-1.7897	0.073502	0.387766
FTL_1606	4700.389	0.408187	0.293834	1.389173	0.16478	0.556713
FTL_1607	501.8212	0.135476	0.287014	0.472018	0.636914	0.866168
FTL_1608	1017.948	-0.32937	0.251989	-1.30706	0.191192	0.575235
FTL_1609	3371.848	0.013702	0.244602	0.056018	0.955328	0.991777
FTL_1610	1012.764	0.30552	0.259054	1.179368	0.238252	0.609208
FTL_1611	2738.76	-0.30647	0.258255	-1.18669	0.235349	0.608818
FTL_1612	931.4832	0.282922	0.265916	1.063952	0.28735	0.643901
FTL_1613	2148.919	0.020042	0.23575	0.085013	0.932251	0.982421
FTL_1614	2114.126	0.00709	0.24152	0.029357	0.97658	0.994309
FTL_1615	2434.339	0.091839	0.233884	0.392668	0.694565	0.895494
FTL_1616	7764.129	-0.09503	0.250883	-0.37878	0.70485	0.899212
FTL_1617	4554.279	-0.19908	0.231126	-0.86137	0.389034	0.735977
FTL_1618	526.6197	-0.05279	0.268995	-0.19626	0.844407	0.951911
FTL_1619	359.7902	0.407726	0.322482	1.264335	0.20611	0.586521
FTL_1620	541.5614	0.081859	0.271176	0.301867	0.762754	0.920389
FTL_1621	3447.061	0.200529	0.238704	0.840077	0.400865	0.746747
FTL_1622	2257.643	0.066246	0.247638	0.267511	0.789076	0.932418
FTL_1623	1031.357	-0.11726	0.266911	-0.43933	0.660426	0.87911
FTL_1624	2385.109	0.546998	0.270445	2.022583	0.043116	0.285077
FTL_1625	187.0937	0.01436	0.328628	0.043698	0.965145	0.994309
FTL_1626	51.58833	0.154881	0.390959	0.396156	0.69199	0.892747
FTL_1627	1045.902	0.323058	0.255372	1.265051	0.205853	0.586521
FTL_1628	171.9847	0.246141	0.336501	0.73147	0.464492	0.77919
FTL_1629	587.5488	0.778401	0.338705	2.298169	0.021552	0.183559
FTL_1630	985.738	0.357728	0.264895	1.35045	0.176872	0.569084
FTL_1631	220.2873	0.349563	0.330825	1.05664	0.290676	0.643901

FTL_1632	70.66481	0.310662	0.376836	0.824396	0.409714	0.752409
FTL_1633	375.4502	0.262236	0.280074	0.936309	0.349114	0.703126
FTL_1634	817.462	0.186359	0.259476	0.718214	0.472626	0.786405
FTL_1635	318.773	0.12124	0.294736	0.411351	0.680815	0.884928
FTL_1636	90.35506	0.195626	0.361594	0.541009	0.588501	0.842174
FTL_1637	4939.215	-0.08039	0.249332	-0.32242	0.747135	0.914459
FTL_1638	999.6188	-0.31562	0.253719	-1.24398	0.213508	0.587852
FTL_1639	1069.335	-0.05144	0.252554	-0.20366	0.838619	0.949983
FTL_1640	1109.47	-0.67309	0.253086	-2.65954	0.007825	0.092516
FTL_1641	484.9382	-0.56929	0.283523	-2.00791	0.044653	0.291493
FTL_1642	903.6712	-0.29622	0.257556	-1.15013	0.250091	0.618143
FTL_1643	1340.894	0.223216	0.248898	0.896818	0.369816	0.718193
FTL_1644	4053.664	-0.163	0.231716	-0.70346	0.481768	0.79199
FTL_1645	1237.879	0.016616	0.254118	0.065386	0.947866	0.989719
FTL_1646	1459.048	0.890089	0.323554	2.750978	0.005942	0.075857
FTL_1647	204.656	0.666661	0.369226	1.805566	0.070986	0.378915
FTL_1648	102.2249	-0.18162	0.354764	-0.51195	0.608688	0.851082
FTL_1649	46.41597	0.210697	0.394342	0.534301	0.593134	0.844932
FTL_1650	31.52384	0.156365	0.400897	0.390037	0.69651	0.896555
FTL_1651	51.21965	0.098272	0.39133	0.251123	0.801719	0.937924
FTL_1652	20.39091	0.331216	0.394876	0.838784	0.40159	0.747404
FTL_1653	21.7885	0.186818	0.396627	0.471017	0.637628	0.866554
FTL_1654	225.0622	0.349505	0.307814	1.135443	0.25619	0.623375
FTL_1655	207.1293	0.317485	0.326988	0.970938	0.331579	0.684969
FTL_1656	3002.611	-0.23941	0.235393	-1.01705	0.309131	0.660785
FTL_1657	2848.366	0.147813	0.256947	0.575267	0.565111	0.829596
FTL_1658	4922.929	-0.25351	0.233261	-1.08681	0.277121	0.638231
FTL_1659	3963.747	0.121177	0.237204	0.510856	0.609452	0.851082
FTL_1660	569.5252	-0.30868	0.274969	-1.1226	0.261606	0.628129
FTL_1661	1671.927	-0.41536	0.255237	-1.62734	0.103664	0.451804
FTL_1662	2595.296	-0.04955	0.237281	-0.20883	0.834583	0.947747
FTL_1663	1952.887	-0.35996	0.264868	-1.35902	0.174142	0.565576
FTL_1664	4075.845	-0.27303	0.255449	-1.06882	0.28515	0.643901
FTL_1665	2241.724	-0.26265	0.236227	-1.11187	0.266195	0.630978
FTL_1666	4912.943	-0.33797	0.260455	-1.29762	0.194417	0.57979
FTL_1667	1829.307	-0.07602	0.247411	-0.30725	0.758652	0.919801
FTL_1668	7502.933	-0.3301	0.250552	-1.31749	0.187674	0.575235
FTL_1669	663.3793	-0.32591	0.268741	-1.21274	0.225228	0.596453
FTL_1670	1488.316	0.202764	0.277433	0.730859	0.464865	0.77919
FTL_1671	4670.803	-0.29325	0.238531	-1.22942	0.218915	0.592692
FTL_1672	6951.221	-0.33017	0.251385	-1.31341	0.189046	0.575235
FTL_1673	4995.831	-0.00384	0.229278	-0.01675	0.986634	0.996049
FTL_1674	238.854	0.447651	0.327807	1.365594	0.172066	0.56512

FTL_1675	55.15709	0.459889	0.395899	1.161631	0.245385	0.616834
FTL_1676	117.6644	0.633756	0.381409	1.661615	0.09659	0.445547
FTL_1677	190.1788	0.392384	0.342506	1.145626	0.25195	0.618781
FTL_1678	8365.271	-0.72522	0.227136	-3.1929	0.001409	0.028695
FTL_1679	126.1464	0.150147	0.354202	0.423903	0.671637	0.881992
FTL_1680	227.9749	0.309879	0.322512	0.960829	0.336638	0.691157
FTL_1681	1060.934	0.301193	0.255072	1.180816	0.237676	0.609208
FTL_1682	52.88346	0.38447	0.396783	0.968967	0.332562	0.685589
FTL_1683	3067.654	-0.15754	0.259432	-0.60723	0.543696	0.824572
FTL_1684	2291.941	-0.0663	0.276998	-0.23935	0.810833	0.939669
FTL_1685	1997.599	-0.5043	0.259571	-1.94282	0.052038	0.319864
FTL_1686	827.2681	-0.21834	0.277295	-0.78738	0.431059	0.770845
FTL_1687	1195.653	0.332627	0.254706	1.305922	0.191579	0.575235
FTL_1688	403.623	0.682168	0.35364	1.928991	0.053732	0.326288
FTL_1689	1132.874	0.11365	0.275806	0.412064	0.680293	0.884928
FTL_1690	695.3952	0.347238	0.265343	1.308636	0.190658	0.575235
FTL_1691	294.5732	0.508423	0.303655	1.674347	0.094062	0.443941
FTL_1692	471.9195	0.234819	0.272721	0.861023	0.389225	0.735977
FTL_1693	519.4988	0.428733	0.323927	1.323549	0.185653	0.575235
FTL_1694	558.9812	0.116777	0.267144	0.437133	0.662015	0.880059
FTL_1695	555.0245	0.365952	0.279523	1.309201	0.190466	0.575235
FTL_1696	469.1732	-0.1679	0.298845	-0.56184	0.574227	0.832153
FTL_1697	48.01885	0.31601	0.394513	0.801014	0.423124	0.762761
FTL_1698	65.00555	0.599008	0.392769	1.525092	0.127236	0.50146
FTL_1699	3094.161	0.472978	0.251752	1.878744	0.060279	0.34519
FTL_1700	2284.458	-0.41528	0.251837	-1.64899	0.09915	0.446841
FTL_1701	6201.636	-0.45454	0.23382	-1.94398	0.051898	0.319864
FTL_1702	3207.665	-0.27649	0.242522	-1.14004	0.254268	0.621749
FTL_1703	6163.879	-0.44792	0.266874	-1.6784	0.093268	0.442144
FTL_1704	754.3204	0.129577	0.254656	0.508831	0.610871	0.851491
FTL_1705	4319.973	-0.1274	0.256832	-0.49606	0.619853	0.85747
FTL_1706	1112.683	0.066526	0.256916	0.25894	0.795682	0.937468
FTL_1707	482.2951	-0.17706	0.292391	-0.60555	0.544812	0.824572
FTL_1708	4028.247	0.171526	0.233153	0.735679	0.461926	0.77919
FTL_1709	3218.574	0.17593	0.279687	0.629024	0.529333	0.812401
FTL_1710	4713.702	-0.11572	0.23608	-0.49017	0.624015	0.860048
FTL_1711	402.0198	1.155213	0.357395	3.232316	0.001228	0.025709
FTL_1712	179.362	1.475257	0.367306	4.016429	5.91E-05	0.002424
FTL_1713	2588.486	0.161527	0.237024	0.68148	0.495568	0.79879
FTL_1714	126410.9	-0.01298	0.26723	-0.04856	0.961272	0.992291
FTL_1715	16920.57	0.151636	0.307317	0.493419	0.621717	0.859457
FTL_1716	511.5536	0.803043	0.337716	2.377864	0.017413	0.155558
FTL_1717	1326.43	-0.06705	0.246745	-0.27173	0.785828	0.931317

FTL_1718	1154.106	0.195611	0.248245	0.787976	0.430711	0.770845
FTL_1719	350.8052	0.203501	0.29315	0.694188	0.487564	0.793769
FTL_1720	1633.347	-0.00484	0.240245	-0.02013	0.983938	0.995965
FTL_1721	2848.531	-0.24836	0.240827	-1.03129	0.302407	0.652888
FTL_1722	2777.082	-0.13964	0.263461	-0.53003	0.596089	0.845587
FTL_1723	2142.115	-0.00189	0.242421	-0.00779	0.993782	0.996724
FTL_1724	4389.924	-0.11342	0.264151	-0.42939	0.667639	0.881666
FTL_1725	2395.752	-0.07969	0.260675	-0.30571	0.759825	0.919801
FTL_1726	3577.672	-0.29339	0.231213	-1.26892	0.20447	0.586521
FTL_1727	685.4824	-0.58432	0.263709	-2.21576	0.026708	0.215592
FTL_1728	1704.248	0.141067	0.267693	0.526975	0.598211	0.846141
FTL_1729	1760.447	-0.27331	0.254565	-1.07364	0.282985	0.643901
FTL_1730	416.7434	0.434646	0.303112	1.433945	0.151588	0.542156
FTL_1731	644.4872	0.456377	0.302367	1.509348	0.13121	0.507288
FTL_1732	1208.672	0.048202	0.245341	0.19647	0.844242	0.951911
FTL_1733	3471.592	0.056269	0.23388	0.240591	0.809872	0.939549
FTL_1734	3237.834	-0.05427	0.254362	-0.21335	0.831056	0.945344
FTL_1735	6553.101	-0.34211	0.272043	-1.25754	0.208558	0.586556
FTL_1736	17086.29	-0.00411	0.226205	-0.01817	0.9855	0.995965
FTL_1737	4189.395	-0.04977	0.255195	-0.19504	0.845365	0.951924
FTL_1738	4322.556	-0.32626	0.265045	-1.23097	0.218333	0.592692
FTL_1739	2679.882	0.437186	0.232691	1.878829	0.060268	0.34519
FTL_1740	22453.54	-0.40957	0.272494	-1.50305	0.132827	0.509395
FTL_1741	4645.741	-0.01774	0.234829	-0.07556	0.939768	0.985525
FTL_1742	582.732	-0.29939	0.27046	-1.10697	0.268305	0.632232
FTL_1743	78863.49	-0.34143	0.269993	-1.2646	0.206013	0.586521
FTL_1744	76434.73	-0.16937	0.249966	-0.67758	0.498035	0.800092
FTL_1745	10198.52	-0.47101	0.294146	-1.6013	0.109311	0.466502
FTL_1746	18594.07	-0.49085	0.293576	-1.67197	0.094531	0.443941
FTL_1747	18983.2	-0.39063	0.268942	-1.45248	0.146367	0.532002
FTL_1748	17591.46	0.017384	0.233622	0.07441	0.940684	0.985574
FTL_1749	9901.053	-0.22027	0.255522	-0.86205	0.38866	0.735977
FTL_1750	4721.95	-0.28126	0.265494	-1.05938	0.289427	0.643901
FTL_1751	168643.1	0.148108	0.226388	0.654223	0.512968	0.804396
FTL_1752	514.2352	-0.05548	0.294665	-0.18827	0.850668	0.952754
FTL_1753	1072.294	-0.28812	0.254078	-1.134	0.256793	0.623375
FTL_1754	981.8198	-0.27194	0.254851	-1.06707	0.28594	0.643901
FTL_1755	1970.369	-0.64758	0.250255	-2.58768	0.009662	0.101684
FTL_1756	5223.731	-0.33457	0.228344	-1.46519	0.142869	0.523071
FTL_1757	1041.34	0.290967	0.253034	1.149911	0.250181	0.618143
FTL_1758	198.8904	0.362744	0.330781	1.096628	0.272804	0.63686
FTL_1759	213.4296	0.047048	0.316022	0.148877	0.881651	0.962584
FTL_1760	61.12197	0.439074	0.387041	1.134438	0.256611	0.623375

FTL_1761	17.55327	0.500014	0.386894	1.292382	0.196225	0.583449
FTL_1762	2209.573	0.342513	0.268546	1.275436	0.202155	0.58465
FTL_1763	848.2634	0.595041	0.312021	1.907054	0.056514	0.330934
FTL_1764	365.6055	0.225429	0.306834	0.734693	0.462527	0.77919
FTL_1765	242.7458	-0.44805	0.32684	-1.37086	0.170419	0.563239
FTL_1766	101.4568	-0.60363	0.354731	-1.70167	0.088818	0.430505
FTL_1767	52.72004	0.015864	0.388833	0.040799	0.967456	0.994309
FTL_1768	22.22549	0.10737	0.397037	0.270427	0.786831	0.931452
FTL_1769	69.21754	0.361496	0.39137	0.92367	0.355658	0.708356
FTL_1770	230.3618	0.31726	0.32129	0.987457	0.323418	0.677157
FTL_1771	581.8697	0.167847	0.280049	0.599349	0.54894	0.824572
FTL_1772	25463.77	-0.18733	0.245113	-0.76427	0.444709	0.775733
FTL_1773	1226.458	-0.1641	0.248793	-0.65959	0.509516	0.804396
FTL_1774	138.1116	-0.05034	0.337546	-0.14915	0.881438	0.962584
FTL_1775	1349.714	-0.07329	0.243412	-0.30111	0.763328	0.920389
FTL_1776	855.987	0.146949	0.25369	0.579245	0.562424	0.829057
FTL_1777	396.6718	0.21703	0.27733	0.782571	0.433879	0.771103
FTL_1778	946.6954	0.451999	0.249215	1.813686	0.069726	0.378915
FTL_1779	2697.5	-0.21354	0.279084	-0.76516	0.444179	0.775733
FTL_1780	3715.528	-0.267	0.284319	-0.9391	0.347679	0.703053
FTL_1781	7444.405	-0.25717	0.283399	-0.90746	0.364162	0.712387
FTL_1782	3756.895	-0.47963	0.309434	-1.55003	0.121134	0.485989
FTL_1783	20652.93	-0.06883	0.230744	-0.29829	0.765485	0.920636
FTL_1784	28442.4	-0.19096	0.234774	-0.81337	0.416008	0.75672
FTL_1785	11787.71	-0.23711	0.240892	-0.98428	0.324977	0.678056
FTL_1786	12626	-0.24107	0.25935	-0.92951	0.352625	0.706656
FTL_1787	1892.887	-0.3394	0.265881	-1.2765	0.201777	0.58465
FTL_1788	8339.107	0.504931	0.268474	1.880744	0.060007	0.34519
FTL_1789	27931.02	-0.0556	0.264607	-0.21012	0.833577	0.94714
FTL_1790	7343.915	-0.49748	0.229856	-2.16432	0.03044	0.235325
FTL_1791	5425.855	-0.29115	0.228071	-1.27659	0.201748	0.58465
FTL_1792	1702.485	-0.18982	0.255283	-0.74355	0.457147	0.77919
FTL_1793	5800.313	0.003255	0.240512	0.013533	0.989203	0.996358
FTL_1794	3886.77	-0.4185	0.293273	-1.42699	0.153583	0.543488
FTL_1795	14359.97	-0.4986	0.286136	-1.74252	0.081418	0.408103
FTL_1796	8929.407	-0.17629	0.251234	-0.70169	0.48287	0.792187
FTL_1797	21277.78	-0.3622	0.256787	-1.41049	0.158394	0.547973
FTL_1798	4981.641	-0.29259	0.248952	-1.17527	0.239886	0.609572
FTL_1799	4165.806	-0.33894	0.263909	-1.28433	0.199028	0.583892
FTL_1800	8445.358	-0.32918	0.245498	-1.34086	0.179967	0.569084
FTL_1801	6266.646	-0.22319	0.261071	-0.85491	0.392599	0.738706
FTL_1802	925.1472	-0.01122	0.255348	-0.04394	0.964955	0.994309
FTL_1803	2914.364	-0.30991	0.242292	-1.27908	0.200869	0.58465

FTL_1804	1532.449	-0.45952	0.250397	-1.83515	0.066483	0.37017
FTL_1805	1707.469	-0.4334	0.240318	-1.80344	0.071319	0.378915
FTL_1806	1779.919	-0.49968	0.249866	-1.99979	0.045523	0.294122
FTL_1807	3556.365	-0.35838	0.246376	-1.45461	0.145778	0.530823
FTL_1808	1021.196	-0.30835	0.275347	-1.11984	0.262781	0.628796
FTL_1809	15854.98	-0.27724	0.242216	-1.14461	0.252371	0.618781
FTL_1810	9883.607	-0.1485	0.24435	-0.60774	0.543359	0.824572
FTL_1811	5887.277	0.115097	0.233802	0.492281	0.62252	0.859977
FTL_1812	1295.17	-0.45073	0.249933	-1.80342	0.071322	0.378915
FTL_1813	46.82485	-0.15774	0.394232	-0.40012	0.689065	0.892023
FTL_1814	372.2384	0.277963	0.300413	0.925269	0.354826	0.707654
FTL_1815	1026.615	0.280706	0.257669	1.089403	0.275976	0.638231
FTL_1816	773.0477	0.132334	0.257521	0.513875	0.607339	0.851082
FTL_1817	9944.436	-0.14352	0.236359	-0.6072	0.543715	0.824572
FTL_1818	6199.974	-0.43741	0.289849	-1.5091	0.131273	0.507288
FTL_1819	7848.07	-0.42769	0.282308	-1.51497	0.12978	0.506185
FTL_1820	826.8843	-0.42729	0.319184	-1.33869	0.18067	0.569196
FTL_1821	2419.703	-0.03876	0.239146	-0.16208	0.871243	0.958064
FTL_1822	1750.859	-0.22557	0.289829	-0.77827	0.436408	0.771103
FTL_1823	4249.955	-0.27754	0.292629	-0.94845	0.342899	0.698762
FTL_1824	13126.36	-0.14064	0.266933	-0.52686	0.59829	0.846141
FTL_1825	8532.227	-0.0219	0.236267	-0.09268	0.926159	0.980295
FTL_1826	2183.948	-0.06085	0.270587	-0.2249	0.822059	0.943654
FTL_1827	6835.789	0.002729	0.242921	0.011232	0.991038	0.996492
FTL_1828	4534.324	0.241509	0.233974	1.032205	0.301976	0.652888
FTL_1829	3261.552	0.005549	0.284231	0.019524	0.984423	0.995965
FTL_1830	2988.59	0.347156	0.236591	1.467324	0.142288	0.523071
FTL_1831	874.4237	-0.35526	0.264765	-1.3418	0.17966	0.569084
FTL_1832	4841.02	-0.16726	0.326342	-0.51253	0.608282	0.851082
FTL_1833	826.3327	-0.26922	0.339003	-0.79416	0.427103	0.767597
FTL_1834	936.1466	-0.34194	0.366422	-0.93319	0.350724	0.704956
FTL_1835	479.6716	-0.75776	0.367827	-2.06009	0.03939	0.270219
FTL_1836	480.1194	0.237046	0.292675	0.809929	0.417981	0.758935
FTL_1837	82.55315	0.031084	0.369032	0.084231	0.932873	0.982421
FTL_1838	105.2611	0.172831	0.369824	0.467333	0.640262	0.867785
FTL_1839	686.9213	0.022831	0.258857	0.088201	0.929717	0.982322
FTL_1840	2348.851	-0.05891	0.235661	-0.24998	0.802601	0.937924
FTL_1841	7043.235	0.13398	0.229865	0.582863	0.559986	0.828816
FTL_1842	7176.849	0.078843	0.235195	0.335222	0.737458	0.911617
FTL_1843	1080.231	0.047431	0.264756	0.179152	0.857819	0.954771
FTL_1844	162.9326	0.219469	0.324615	0.67609	0.498984	0.800092
FTL_1845	211.4184	0.536669	0.31264	1.716572	0.086057	0.423959
FTL_1846	86.06853	0.451501	0.366978	1.230323	0.218576	0.592692

FTL_1847	362.8412	0.05267	0.283484	0.185796	0.852605	0.953665
FTL_1848	1149.895	0.20974	0.256851	0.816583	0.414167	0.754738
FTL_1849	2954.217	0.191573	0.233314	0.821095	0.411592	0.752775
FTL_1850	2916.461	-0.3554	0.239786	-1.48214	0.138303	0.51767
FTL_1851	244.3571	0.363294	0.315204	1.152568	0.249088	0.618143
FTL_1852	457.6539	-0.26748	0.273349	-0.97851	0.32782	0.681404
FTL_1853	209.5601	0.455279	0.333882	1.363595	0.172695	0.565338
FTL_1854	531.4953	0.035799	0.271828	0.131696	0.895225	0.96895
FTL_1855	220.0116	-0.09549	0.311518	-0.30654	0.759192	0.919801
FTL_1856	39.10646	0.003026	0.399058	0.007584	0.993949	0.996724
FTL_1857	58.00135	0.177312	0.385188	0.460325	0.645283	0.872239
FTL_1858	223.0554	0.078118	0.307895	0.253715	0.799716	0.937924
FTL_1859	225.5355	-0.52405	0.314353	-1.66707	0.0955	0.445199
FTL_1860	3888.833	-0.4518	0.253978	-1.77889	0.075258	0.390874
FTL_1861	3073.754	-0.39616	0.243395	-1.62765	0.103599	0.451804
FTL_1862	170.9484	-0.1445	0.335948	-0.43014	0.667094	0.881666
FTL_1863	586.9402	-0.3048	0.290726	-1.04842	0.294444	0.643956
FTL_1864	1262.351	-0.1479	0.243351	-0.60774	0.543357	0.824572
FTL_1865	8837.945	0.135118	0.226044	0.597751	0.550006	0.824572
FTL_1866	2927.686	-0.20325	0.245637	-0.82742	0.407998	0.752409
FTL_1867	4550.289	-0.26692	0.245279	-1.08825	0.276484	0.638231
FTL_1868	3496.599	0.178486	0.256498	0.695857	0.486518	0.793752
FTL_1869	2428.359	-0.27215	0.260506	-1.04468	0.29617	0.644964
FTL_1870	426.4057	0.051381	0.286547	0.179311	0.857693	0.954771
FTL_1871	470.7971	-0.06926	0.270621	-0.25592	0.79801	0.937924
FTL_1872	434.1071	0.034726	0.275018	0.126269	0.899519	0.969455
FTL_1873	3792.773	-0.02639	0.250255	-0.10543	0.916032	0.97571
FTL_1874	2006.075	-0.37898	0.272675	-1.38984	0.164577	0.556713
FTL_1875	594.3506	-0.06123	0.271578	-0.22548	0.82161	0.943654
FTL_1876	446.014	-0.5414	0.297941	-1.81713	0.069197	0.378915
FTL_1877	190.1263	0.290326	0.344526	0.842682	0.399406	0.745959
FTL_1878	872.7517	-0.05279	0.286009	-0.18459	0.853549	0.95419
FTL_1879	3308.801	-0.74534	0.236236	-3.15507	0.001605	0.03162
FTL_1880	175.5597	-0.45867	0.321261	-1.42771	0.153376	0.543488
FTL_1881	1044.785	-0.22735	0.249068	-0.9128	0.361348	0.712199
FTL_1882	2415.638	-0.42177	0.247851	-1.70171	0.08881	0.430505
FTL_1883	3052.178	-0.52085	0.252243	-2.06487	0.038935	0.268932
FTL_1884	39.74154	0.319869	0.398315	0.803056	0.421943	0.762684
FTL_1885	339.4178	0.270839	0.31386	0.862928	0.388177	0.735977
FTL_1886	919.9744	0.290473	0.259066	1.121232	0.262189	0.628129
FTL_1887	13.66128	0.48011	0.374463	1.282127	0.199798	0.584137
FTL_1888	39.51046	-0.09915	0.39783	-0.24923	0.803184	0.938059
FTL_1889	120.2617	0.681873	0.355228	1.919538	0.054916	0.326617

FTL_1890	10.74982	0.097001	0.360261	0.269254	0.787734	0.931928
FTL_1891	1045.706	0.270316	0.255103	1.059631	0.289312	0.643901
FTL_1892	5335.804	-0.66647	0.234033	-2.84777	0.004403	0.06431
FTL_1893	66.72978	0.438789	0.378464	1.159395	0.246295	0.617
FTL_1894	368.86	0.618868	0.317491	1.949246	0.051266	0.319864
FTL_1895	162.0148	0.328442	0.336174	0.976998	0.32857	0.681554
FTL_1896	950.6969	0.019366	0.263264	0.073563	0.941358	0.985574
FTL_1897	696.7587	0.30739	0.28189	1.090462	0.27551	0.638231
FTL_1898	1689.261	0.445272	0.286551	1.5539	0.120208	0.483237
FTL_1899	6806.55	-0.13695	0.233668	-0.58607	0.557826	0.828397
FTL_1900	4642.405	-0.20958	0.274338	-0.76393	0.444907	0.775733
FTL_1901	1149.839	-0.29872	0.253407	-1.1788	0.238478	0.609208
FTL_1902	1520.497	-0.46405	0.243722	-1.90402	0.056907	0.331547
FTL_1903	6129.976	0.013412	0.229441	0.058455	0.953386	0.991454
FTL_1904	2988.358	0.129061	0.236936	0.544708	0.585955	0.840063
FTL_1905	3185.48	-0.00667	0.231876	-0.02875	0.977065	0.994309
FTL_1906	6601.407	-0.4891	0.273517	-1.78819	0.073745	0.38803
FTL_1907	9858.55	-0.44911	0.259931	-1.7278	0.084023	0.420117
FTL_1908	3548.936	-0.28419	0.255587	-1.11192	0.266173	0.630978
FTL_1909	1607.281	0.212173	0.246189	0.861829	0.388782	0.735977
FTL_1910	5034	-0.03785	0.228813	-0.16543	0.868606	0.958064
FTL_1911	994.2032	0.283777	0.251568	1.128036	0.259305	0.6272
FTL_1912	55510.9	-0.35237	0.289708	-1.21629	0.223874	0.596045
FTL_1913	999.8395	-0.25216	0.275557	-0.9151	0.360141	0.712199
FTL_1914	20249.09	0.251952	0.238767	1.05522	0.291325	0.643901
FTL_1915	1407.446	0.232945	0.322861	0.721502	0.4706	0.784985
FTL_1916	923.9445	0.361714	0.285576	1.266614	0.205293	0.586521
FTL_1917	1221.635	0.660013	0.253857	2.599937	0.009324	0.099688
FTL_1918	438.2656	0.593746	0.281419	2.109831	0.034873	0.250338
FTL_1919	50.39561	0.441787	0.397159	1.112368	0.26598	0.630978
FTL_1920	36.24552	0.537827	0.400382	1.343287	0.179179	0.569084
FTL_1921	349.0168	0.041109	0.318277	0.129159	0.897232	0.969068
FTL_1922	577.0766	-0.41866	0.264125	-1.58507	0.112951	0.471999
FTL_1923	2636.196	-0.62045	0.237258	-2.61507	0.008921	0.097983
FTL_1924	715.5606	0.215553	0.282211	0.763802	0.444985	0.775733
FTL_1925	968.4818	0.279533	0.265778	1.051752	0.292913	0.643901
FTL_1926	188.1372	0.286413	0.330899	0.865561	0.386731	0.735977
FTL_1927	64.9073	-0.12202	0.390079	-0.31282	0.75442	0.919801
FTL_1928	215.984	0.247779	0.347098	0.713859	0.475314	0.788011
FTL_1929	2633.526	-0.74552	0.272742	-2.73342	0.006268	0.078252
FTL_1930	6190.184	-0.38127	0.264481	-1.44157	0.149424	0.536327
FTL_1931	3137.376	-0.26152	0.246097	-1.06267	0.287931	0.643901
FTL_1932	512.7134	0.010748	0.269902	0.039822	0.968235	0.994309

FTL_1933	771.3651	-0.24331	0.258492	-0.94126	0.34657	0.701515
FTL_1934	1335.195	-0.20437	0.268294	-0.76175	0.44621	0.776858
FTL_1935	5167.714	0.237344	0.236573	1.003259	0.315736	0.66803
FTL_1936	8075.437	-0.25108	0.256172	-0.98012	0.327029	0.680588
FTL_1937	174.1932	0.021318	0.336158	0.063417	0.949435	0.990329
FTL_1938	1751.36	-0.13643	0.264974	-0.5149	0.606624	0.851082
FTL_1939	1012.369	0.326317	0.254218	1.28361	0.199278	0.583892
FTL_1940	1384.581	0.217981	0.242718	0.898085	0.36914	0.717574
FTL_1941	1254.294	0.18288	0.246886	0.740749	0.458845	0.77919
FTL_1942	559.4591	-0.1217	0.280391	-0.43403	0.664264	0.881666
FTL_1943	1276.872	-0.1093	0.246375	-0.44362	0.657316	0.87702
FTL_1944	1010.596	0.306657	0.258038	1.188417	0.234669	0.608818
FTL_1945	319.9671	0.351386	0.302392	1.16202	0.245227	0.616834
FTL_1946	832.8467	-0.19967	0.285494	-0.6994	0.484303	0.79253
FTL_1947	5891.303	-0.22912	0.252597	-0.90708	0.364365	0.712387
FTL_1948	405.8623	0.016771	0.287433	0.058346	0.953473	0.991454
FTL_1949	27.08852	0.34966	0.397093	0.88055	0.378561	0.726782
FTL_1950	386.6372	-0.02297	0.281339	-0.08164	0.934935	0.982852
FTL_1951	135.2699	0.442258	0.372092	1.188572	0.234608	0.608818
FTL_1952	209.5572	0.077231	0.319935	0.241395	0.809249	0.939549
FTL_1953	69.35792	-0.07333	0.381262	-0.19233	0.84748	0.952704
FTL_1954	153.942	-0.01341	0.344578	-0.03892	0.968956	0.994309
FTL_1955	172.8699	0.291136	0.33663	0.864854	0.387119	0.735977
FTL_1956	1810.679	-0.68166	0.260195	-2.6198	0.008798	0.097704
FTL_1957	4794.748	0.452772	0.342764	1.320944	0.18652	0.575235
FTL_1958	4776.91	0.524629	0.310093	1.691844	0.090676	0.434915
FTL_1959	109.7455	0.593452	0.362338	1.637843	0.101454	0.448176
FTL_1960	634.2958	-0.17717	0.264282	-0.67036	0.502626	0.800538
FTL_1961	415.1988	-0.31658	0.284282	-1.11362	0.265441	0.630978
FTL_1962	323.8302	-0.4612	0.289354	-1.59388	0.110963	0.466602
FTL_1963	266.8921	-0.43004	0.302443	-1.42188	0.15506	0.544009
FTL_1964	1936.773	-0.34018	0.269362	-1.26292	0.206618	0.586521
FTL_1965	1616.39	-0.48968	0.28385	-1.72515	0.0845	0.42041
FTL_1966	5961.734	-0.35954	0.261627	-1.37424	0.169368	0.563239
FTL_1967	1215.51	-0.30424	0.248353	-1.22502	0.220568	0.593974
FTL_1968	744.6049	0.086712	0.280901	0.308691	0.757557	0.919801
FTL_R0001	502537.7	-0.34854	0.331504	-1.0514	0.293077	0.643901
FTL_R0002	347.3459	0.117421	0.323415	0.363066	0.716556	0.904215
FTL_R0004	87.03874	0.09185	0.376572	0.243912	0.807299	0.939549
FTL_R0005	151.3881	0.076648	0.35615	0.215213	0.829601	0.945294
FTL_R0008	87.85612	0.149517	0.364352	0.410364	0.681539	0.884928
FTL_R0009	512.6628	0.510718	0.3225	1.583621	0.11328	0.472392
FTL_R0010	36390.7	0.009416	0.266876	0.035283	0.971854	0.994309

FTL_R0011	847.58	0.487938	0.304759	1.601061	0.109363	0.466502
FTL_R0012	1278.293	0.409665	0.298064	1.374418	0.169312	0.563239
FTL_R0013	44.71057	0.292406	0.39536	0.739595	0.459546	0.77919
FTL_R0014	204.9449	0.72686	0.33711	2.156148	0.031072	0.235785
FTL_R0015	462.9794	0.731972	0.323306	2.264021	0.023573	0.195791
FTL_R0017	87.03874	0.09185	0.376572	0.243912	0.807299	0.939549
FTL_R0018	151.3881	0.076648	0.35615	0.215213	0.829601	0.945294
FTL_R0021	743.8036	1.255237	0.266766	4.705382	2.53E-06	0.000159
FTL_R0022	2259.57	-0.05111	0.251026	-0.2036	0.838664	0.949983
FTL_R0023	766.6341	0.618068	0.283334	2.181412	0.029153	0.227794
FTL_R0024	115.4438	0.331784	0.370597	0.895268	0.370644	0.718388
FTL_R0025	249.0703	0.304729	0.368677	0.826547	0.408494	0.752409
FTL_R0026	398.4823	0.322156	0.353533	0.911245	0.362166	0.712284
FTL_R0027	156.7978	0.527655	0.356667	1.479406	0.139032	0.519431
FTL_R0029	87.03874	0.09185	0.376572	0.243912	0.807299	0.939549
FTL_R0030	151.3881	0.076648	0.35615	0.215213	0.829601	0.945294
FTL_R0033	31.07615	0.284212	0.400846	0.70903	0.478306	0.789323
FTL_R0034	9.009369	0.18454	0.346767	0.532173	0.594606	0.845587
FTL_R0035	492.5292	0.48038	0.286943	1.674127	0.094106	0.443941
FTL_R0036	1027.607	0.161144	0.281453	0.572544	0.566954	0.829991
FTL_R0037	1481.804	0.083456	0.274827	0.303669	0.76138	0.919801
FTL_R0038	1152.862	0.003194	0.273332	0.011685	0.990677	0.996492
FTL_R0039	642.3901	0.104857	0.279528	0.375123	0.707569	0.900705
FTL_R0040	176.2696	0.533133	0.31998	1.666145	0.095685	0.445199
FTL_R0041	84.45934	0.122823	0.373829	0.328554	0.742493	0.91279
FTL_R0042	41.38448	0.469314	0.398007	1.179161	0.238334	0.609208
FTL_R0043	286.7049	0.886999	0.351378	2.524348	0.011591	0.116493
FTL_R0044	112.1893	0.2578	0.34798	0.740848	0.458786	0.77919
FTL_R0045	979.4545	-0.05051	0.295507	-0.17093	0.86428	0.956083
FTL_R0046	2638.134	-0.20589	0.246655	-0.83472	0.403875	0.748883
FTL_R0047	1121.302	-0.08454	0.290077	-0.29146	0.770703	0.92177
FTL_R0048	1159.707	0.076519	0.288339	0.265379	0.790718	0.93326
FTL_R0049	284.4868	0.088185	0.29945	0.294491	0.768382	0.920898
FTL_R0050	329.6806	-0.22306	0.342284	-0.65167	0.514616	0.804396
FTL_R0051	1029.584	0.225636	0.307241	0.734393	0.462709	0.77919
FTL_R0052	1330.381	0.227806	0.307248	0.741438	0.458428	0.77919
