# Supplementary material for: Stringent response governs the oxidative stress resistance and virulence of Francisella tularensis
Source: PLoS One. 2019 Oct 24;14(10):e0224094. doi: 10.1371/journal.pone.0224094 (PMC6812791; doi:10.1371/journal.pone.0224094)
Supplement: S2 Table — (PDF) [file pone.0224094.s002.pdf]

S2 Table:

Locus	Base Mean	Log_2FoldChange	lfcSE	Stat	P-value	P - adjusted
FTL_0001	8397.741	-0.33443	0.057809	-5.78517	7.24E-09	4.51E-08
FTL_0002	7021.027	-0.3463	0.060489	-5.72499	1.03E-08	6.36E-08
FTL_0003	2308.848	-0.19906	0.07903	-2.51877	0.011776	0.028348
FTL_0004	951.648	0.21377	0.102881	2.07784	0.037724	0.077058
FTL_0005	83.41796	0.607408	0.262473	2.314176	0.020658	0.046085
FTL_0006	373.5301	0.682205	0.149349	4.567864	4.93E-06	2.26E-05
FTL_0007	263.3427	0.17398	0.173092	1.005132	0.314833	0.437934
FTL_0008	2205.505	0.30232	0.079886	3.784377	0.000154	0.000573
FTL_0009	9312.46	0.069018	0.065201	1.058537	0.289811	0.408785
FTL_0010	2897.783	0.218911	0.076073	2.877654	0.004006	0.011108
FTL_0011	4698.115	0.200738	0.065442	3.067397	0.002159	0.006411
FTL_0012	11945.99	0.12247	0.056327	2.174261	0.029686	0.062611
FTL_0013	2252.899	0.159561	0.076456	2.086961	0.036892	0.075666
FTL_0014	3439.02	-0.34369	0.073274	-4.69042	2.73E-06	1.28E-05
FTL_0015	3679.528	0.500812	0.073305	6.831889	8.38E-12	7.00E-11
FTL_0016	3698.905	0.341132	0.069263	4.92519	8.43E-07	4.18E-06
FTL_0017	1145.834	0.4269	0.101118	4.221789	2.42E-05	0.000103
FTL_0018	1037.016	0.322887	0.101679	3.175544	0.001496	0.004596
FTL_0019	335.1207	-0.22509	0.161206	-1.39631	0.162621	0.25838
FTL_0020	14308.44	-0.20583	0.055574	-3.70368	0.000212	0.000775
FTL_0021	129.9213	0.277969	0.22401	1.240874	0.214652	0.320068
FTL_0022	33.63559	1.123636	0.324085	3.467102	0.000526	0.001795
FTL_0023	950.7154	0.204487	0.103963	1.966922	0.049192	0.095997
FTL_0024	14580.62	0.059973	0.075351	0.79591	0.426085	0.550405
FTL_0025	16.11557	-0.67831	0.332082	-2.0426	0.041092	0.082513
FTL_0026	231.2065	-2.7362	0.201151	-13.6027	3.86E-42	1.41E-40
FTL_0027	307.3496	-1.84969	0.17673	-10.4661	1.24E-25	2.51E-24
FTL_0028	1333.145	-0.07321	0.098668	-0.74194	0.458126	0.580968
FTL_0029	7136.619	0.401907	0.05953	6.751309	1.47E-11	1.19E-10
FTL_0030	3308.457	-0.02365	0.070751	-0.33432	0.738138	0.818344
FTL_0031	518.7688	0.10709	0.130464	0.820835	0.41174	0.536704
FTL_0032	505.9101	0.02036	0.132218	0.153985	0.877622	0.918282
FTL_0033	4585.857	0.426165	0.078621	5.420518	5.94E-08	3.36E-07
FTL_0034	6001.855	-0.39335	0.157821	-2.49239	0.012689	0.030076
FTL_0035	22.12713	0.324644	0.332779	0.975556	0.329285	0.451167
FTL_0036	353.1426	0.013622	0.15177	0.089752	0.928484	0.949781
FTL_0037	1270.678	-1.00218	0.1014	-9.88339	4.91E-23	8.44E-22
FTL_0038	2465.509	-0.96948	0.075605	-12.8229	1.22E-37	3.72E-36
FTL_0039	3632.852	-0.55902	0.084784	-6.59353	4.29E-11	3.40E-10

FTL_0040	309.8169	0.101771	0.159859	0.636633	0.524364	0.642116
FTL_0041	149.6423	0.179885	0.214334	0.839273	0.401316	0.526875
FTL_0042	168.5185	0.285076	0.20463	1.393129	0.163581	0.259304
FTL_0043	202.388	0.309479	0.192355	1.608892	0.10764	0.183127
FTL_0044	971.5878	-0.06062	0.106541	-0.56895	0.56939	0.681641
FTL_0045	2159.444	-0.70581	0.078999	-8.93441	4.09E-19	5.38E-18
FTL_0046	2607.377	-0.4914	0.072786	-6.75129	1.47E-11	1.19E-10
FTL_0047	120.5081	-0.25719	0.229793	-1.11922	0.263048	0.378473
FTL_0048	628.3562	0.06305	0.121031	0.520944	0.602406	0.70724
FTL_0049	219.1888	0.243033	0.192232	1.264268	0.206134	0.311292
FTL_0050	85.67877	0.200041	0.26013	0.769005	0.44189	0.565372
FTL_0051	108.8023	0.342711	0.238944	1.434272	0.151495	0.242633
FTL_0052	78.36907	0.228209	0.269974	0.845301	0.397943	0.523472
FTL_0053	120.9826	0.523523	0.241828	2.164861	0.030398	0.063779
FTL_0054	828.4349	0.26168	0.11216	2.333102	0.019643	0.044295
FTL_0055	294.2309	0.089543	0.16439	0.544698	0.585961	0.695678
FTL_0056	462.7882	-0.19237	0.138757	-1.3864	0.165623	0.260896
FTL_0057	1655.517	-0.30256	0.088357	-3.42431	0.000616	0.002075
FTL_0058	1984.902	-0.19651	0.085672	-2.29372	0.021806	0.048432
FTL_0059	446.1357	-0.01767	0.140274	-0.12599	0.899743	0.930389
FTL_0060	520.7594	-0.04446	0.139905	-0.31776	0.750669	0.828126
FTL_0061	515.4259	-0.0663	0.137634	-0.48168	0.630034	0.728217
FTL_0062	523.2048	0.080064	0.131933	0.606853	0.543949	0.660626
FTL_0063	273.7266	0.158357	0.179401	0.882697	0.3774	0.502699
FTL_0064	32.94038	0.365977	0.324848	1.126608	0.259908	0.375568
FTL_0065	126.7631	0.387888	0.232162	1.670766	0.094768	0.165207
FTL_0066	31.60497	-0.34839	0.322998	-1.0786	0.280764	0.39798
FTL_0067	73.77593	-1.74675	0.280864	-6.21918	5.00E-10	3.51E-09
FTL_0068	1091.635	0.120262	0.103571	1.161153	0.24558	0.358993
FTL_0069	1373.109	-0.19145	0.104619	-1.82994	0.067259	0.12506
FTL_0070	5577.506	-0.44316	0.080389	-5.51268	3.53E-08	2.03E-07
FTL_0071	4898.67	0.160528	0.068629	2.339058	0.019332	0.043759
FTL_0072	2843.434	0.226148	0.079385	2.848739	0.004389	0.012036
FTL_0073	5575.666	-0.4141	0.066669	-6.21119	5.26E-10	3.68E-09
FTL_0074	898.7394	-0.24986	0.111901	-2.23287	0.025557	0.055535
FTL_0075	780.0989	-0.42504	0.113113	-3.75763	0.000172	0.000635
FTL_0076	1337.344	-0.27425	0.091675	-2.99154	0.002776	0.00802
FTL_0077	382.5273	-0.00919	0.152949	-0.06007	0.952098	0.965548
FTL_0078	845.2175	0.228975	0.111111	2.060774	0.039325	0.07944
FTL_0079	206.293	0.120327	0.188097	0.639706	0.522364	0.641387
FTL_0080	785.9003	0.320698	0.112851	2.841774	0.004486	0.012252
FTL_0081	117.8751	0.343451	0.2366	1.451609	0.14661	0.236127
FTL_0082	747.6885	-0.44276	0.113557	-3.89905	9.66E-05	0.00037

FTL_0083	139.6589	-0.1633	0.221394	-0.73762	0.460746	0.583554
FTL_0084	736.719	0.038132	0.117367	0.324892	0.745263	0.824424
FTL_0085	925.6354	-0.18486	0.104011	-1.77727	0.075523	0.137751
FTL_0086	1150.199	0.208163	0.095301	2.184258	0.028943	0.06151
FTL_0087	2024.4	0.066625	0.082069	0.811824	0.416893	0.542015
FTL_0088	2700.887	0.126021	0.075145	1.677039	0.093535	0.163483
FTL_0089	1404.834	-0.13888	0.107002	-1.29795	0.194303	0.297902
FTL_0090	527.4778	0.212232	0.131521	1.613675	0.106598	0.181732
FTL_0091	372.3235	-0.0773	0.155803	-0.49617	0.619777	0.719779
FTL_0092	1728.042	-0.02848	0.086188	-0.33045	0.741061	0.820547
FTL_0093	4078.232	0.631709	0.073057	8.646754	5.30E-18	6.49E-17
FTL_0094	26511.54	-0.43736	0.06159	-7.10122	1.24E-12	1.08E-11
FTL_0095	555.1667	0.169769	0.133558	1.271126	0.203684	0.308286
FTL_0096	761.3554	0.268425	0.113648	2.361893	0.018182	0.041572
FTL_0097	532.1024	-0.99967	0.135135	-7.39754	1.39E-13	1.30E-12
FTL_0098	2165.096	-0.89402	0.077911	-11.4748	1.77E-30	4.08E-29
FTL_0099	2468.057	-0.68065	0.077032	-8.83602	9.92E-19	1.27E-17
FTL_0100	694.3216	-0.51285	0.11733	-4.37104	1.24E-05	5.41E-05
FTL_0101	807.9117	-0.06636	0.111377	-0.59581	0.551302	0.666336
FTL_0102	737.9917	-0.07006	0.119522	-0.58618	0.557753	0.670658
FTL_0103	1675.183	0.125422	0.09446	1.32778	0.184251	0.287199
FTL_0104	7231.97	0.167064	0.057903	2.885248	0.003911	0.010905
FTL_0105	2665.57	0.026697	0.089777	0.297373	0.766182	0.839387
FTL_0106	54.19204	0.274317	0.293252	0.935433	0.349565	0.471245
FTL_0107	952.1148	0.237822	0.103424	2.299487	0.021477	0.047807
FTL_0108	98.91351	0.608082	0.247656	2.45535	0.014075	0.033011
FTL_0109	130.7403	0.492862	0.225724	2.183471	0.029001	0.06151
FTL_0110	21.04544	-0.08154	0.333075	-0.2448	0.806613	0.869326
FTL_0111	1428.076	-3.34829	0.104003	-32.1942	2.13E-227	7.13E-225
FTL_0112	8955.224	-2.33584	0.060534	-38.5875	0	0
FTL_0113	6273.312	-1.75204	0.066669	-26.2798	3.27E-152	6.57E-150
FTL_0114	4240.683	-1.66231	0.065918	-25.2178	2.55E-140	4.28E-138
FTL_0115	808.1356	-1.55155	0.115256	-13.4617	2.63E-41	9.11E-40
FTL_0116	3726.811	-1.64911	0.082736	-19.9322	2.14E-88	2.26E-86
FTL_0117	399.9976	-1.50344	0.163421	-9.19983	3.59E-20	5.04E-19
FTL_0118	1079.904	-1.73816	0.111917	-15.5308	2.15E-54	1.23E-52
FTL_0119	906.5453	-1.67294	0.11418	-14.6518	1.31E-48	6.27E-47
FTL_0120	1244.548	-1.87157	0.099557	-18.799	7.69E-79	7.36E-77
FTL_0121	1049.44	-2.4451	0.11728	-20.8485	1.57E-96	1.86E-94
FTL_0122	475.5017	-1.45576	0.163815	-8.88662	6.30E-19	8.12E-18
FTL_0123	138.7758	-1.12546	0.233178	-4.82663	1.39E-06	6.76E-06

FTL_0124	215.0953	-1.58121	0.20273	-7.79956	6.21E-15	6.40E-14
FTL_0125	1666.158	-2.01225	0.090431	-22.2518	1.08E-109	1.45E-107
FTL_0126	1997.492	-2.62209	0.08584	-30.5463	6.33E-205	1.59E-202
FTL_0127	2615.819	-0.46127	0.079303	-5.81653	6E-09	3.8E-08
FTL_0128	1232.574	0.023543	0.092646	0.254116	0.799406	0.864339
FTL_0129	1809.37	-0.37844	0.095896	-3.94633	7.94E-05	0.000307
FTL_0130	750.7476	-0.51364	0.117558	-4.36922	1.25E-05	5.45E-05
FTL_0131	2512.002	-0.57651	0.075255	-7.66069	1.85E-14	1.85E-13
FTL_0132	4784.951	0.102488	0.062231	1.646893	0.09958	0.171623
FTL_0133	13363.65	0.479921	0.057405	8.360274	6.26E-17	7.15E-16
FTL_0134	141.9552	0.307378	0.234981	1.308097	0.190841	0.294696
FTL_0135	848.3278	0.263667	0.112095	2.352176	0.018664	0.042485
FTL_0136	380.6493	-0.43831	0.155316	-2.82204	0.004772	0.012927
FTL_0137	2877.702	0.047203	0.087044	0.54229	0.587618	0.696001
FTL_0138	2453.826	0.017767	0.074309	0.239096	0.811031	0.870819
FTL_0139	781.1665	0.085129	0.11234	0.757779	0.448584	0.572478
FTL_0140	613.3336	-0.22164	0.123005	-1.8019	0.071561	0.131962
FTL_0141	381.8168	-0.25512	0.14786	-1.72539	0.084456	0.150375
FTL_0142	319.8716	-0.13353	0.159011	-0.83973	0.401061	0.526875
FTL_0143	1172.418	0.057223	0.096295	0.594252	0.552344	0.667194
FTL_0144	3237.731	-0.16645	0.076276	-2.18223	0.029093	0.061619
FTL_0145	2794.486	0.521345	0.07383	7.061392	1.65E-12	1.43E-11
FTL_0146	5353.935	0.567883	0.061233	9.274207	1.79E-20	2.59E-19
FTL_0147	920.694	-0.4283	0.104701	-4.09067	4.3E-05	0.000176
FTL_0148	2987.523	0.121613	0.07906	1.538242	0.123989	0.205966
FTL_0149	1783.828	0.105945	0.083658	1.266396	0.205371	0.310373
FTL_0150	1069.424	0.058584	0.097632	0.600053	0.548471	0.665101
FTL_0151	631.6235	-0.04509	0.126039	-0.35771	0.720557	0.805517
FTL_0152	481.977	-0.34112	0.136768	-2.49414	0.012626	0.030034
FTL_0153	1193	0.150549	0.096941	1.552994	0.120425	0.201041
FTL_0154	214.2094	0.21819	0.186698	1.168682	0.242532	0.355128
FTL_0155	418.3357	-0.0334	0.141806	-0.23556	0.813775	0.871901
FTL_0156	2266.448	0.199279	0.085424	2.33283	0.019657	0.044295
FTL_0157	1972.751	0.126479	0.082807	1.52739	0.126664	0.209027
FTL_0158	4139.623	-0.41833	0.068248	-6.12965	8.81E-10	6E-09
FTL_0159	1246.458	0.080046	0.092698	0.863515	0.387855	0.513562
FTL_0160	696.5041	-0.09904	0.115681	-0.85618	0.391899	0.518235
FTL_0161	1019.629	-0.29493	0.102223	-2.88519	0.003912	0.010905
FTL_0162	197.2691	0.559281	0.19894	2.811309	0.004934	0.013276
FTL_0163	77.92332	0.586499	0.267802	2.19005	0.028521	0.060921
FTL_0164	918.3992	0.200002	0.105076	1.903409	0.056987	0.108986

FTL_0165	413.8886	-0.50474	0.142459	-3.54304	0.000396	0.001371
FTL_0166	9033.987	-0.57649	0.056816	-10.1465	3.43E-24	6.33E-23
FTL_0167	640.6417	0.173309	0.123849	1.399358	0.161706	0.257408
FTL_0168	445.9517	-0.15972	0.138834	-1.15044	0.249965	0.363815
FTL_0169	445.0699	0.168525	0.142416	1.183329	0.236679	0.348516
FTL_0170	214.5189	-1.2054	0.190956	-6.31244	2.75E-10	2E-09
FTL_0171	555.5151	0.023752	0.128977	0.184157	0.853891	0.901438
FTL_0172	2543.399	0.340181	0.074064	4.593064	4.37E-06	2.00E-05
FTL_0173	1414.391	0.163638	0.094696	1.728029	0.083983	0.149783
FTL_0174	1448.464	0.222346	0.098257	2.262908	0.023641	0.052082
FTL_0175	858.6638	-0.23328	0.10722	-2.17571	0.029577	0.062504
FTL_0176	10224.64	0.091764	0.06716	1.366347	0.17183	0.269406
FTL_0177	2697.476	0.27205	0.087662	3.103405	0.001913	0.005739
FTL_0178	8300.106	-0.1734	0.0627	-2.76551	0.005683	0.014941
FTL_0179	2260.913	-0.2198	0.07937	-2.76932	0.005617	0.014798
FTL_0180	3085.232	0.1416	0.074332	1.904975	0.056783	0.1087
FTL_0181	961.2137	-0.19432	0.106358	-1.82705	0.067693	0.125735
FTL_0182	2375.434	-0.31772	0.076961	-4.12837	3.65E-05	0.000151
FTL_0183	1775.101	-0.47805	0.083648	-5.71505	1.1E-08	6.7E-08
FTL_0184	602.1557	-0.3475	0.123892	-2.80487	0.005034	0.013526
FTL_0185	1068.504	0.181047	0.098653	1.835185	0.066478	0.123838
FTL_0186	554.6676	0.94411	0.128346	7.355979	1.90E-13	1.76E-12
FTL_0187	3735.491	0.387686	0.070323	5.512965	3.53E-08	2.03E-07
FTL_0188	9014.526	-0.30467	0.057469	-5.30152	1.15E-07	6.21E-07
FTL_0189	17690.83	-0.01183	0.052551	-0.22516	0.821857	0.875893
FTL_0190	1028.118	0.094201	0.105085	0.896427	0.370025	0.495464
FTL_0191	5103.214	-0.07243	0.067371	-1.07503	0.282362	0.399682
FTL_0192	15701.62	-0.0734	0.052585	-1.39575	0.16279	0.25838
FTL_0193	3256.843	-0.09233	0.073551	-1.25526	0.209385	0.314077
FTL_0194	2105.828	-0.17953	0.077475	-2.31725	0.02049	0.045774
FTL_0195	1218.084	-0.24693	0.104684	-2.35882	0.018333	0.04178
FTL_0196	5747.691	-0.30893	0.069038	-4.47474	7.65E-06	3.43E-05
FTL_0197	1415.688	0.006178	0.08898	0.069434	0.944645	0.959772
FTL_0198	3104.801	0.215488	0.075343	2.860108	0.004235	0.011693
FTL_0199	1790.985	0.244637	0.087945	2.781713	0.005407	0.014301
FTL_0200	2136.42	0.246729	0.077664	3.176884	0.001489	0.004582
FTL_0201	990.0222	0.130541	0.102774	1.270173	0.204023	0.308567
FTL_0202	227.9781	-0.03382	0.186658	-0.18116	0.85624	0.903392
FTL_0203	1025.226	0.088168	0.100261	0.87938	0.379195	0.504756
FTL_0204	982.6833	0.103588	0.106319	0.974317	0.329899	0.451701
FTL_0205	734.2858	-0.01943	0.120493	-0.16122	0.87192	0.914501
FTL_0206	1683.086	-0.17292	0.085701	-2.01774	0.043618	0.086734
FTL_0207	1100.936	-1.37018	0.112058	-12.2275	2.22E-34	6.19E-33

FTL_0208	598.698	-1.63724	0.128331	-12.758	2.81E-37	8.44E-36
FTL_0209	364.4309	-1.48163	0.157525	-9.40573	5.17E-21	7.58E-20
FTL_0210	7442.998	0.143325	0.05864	2.444155	0.014519	0.033895
FTL_0211	1753.658	0.167217	0.089417	1.870076	0.061473	0.116129
FTL_0212	1321.759	0.004881	0.093397	0.052265	0.958317	0.970387
FTL_0213	585.3274	-0.39775	0.128728	-3.08986	0.002003	0.005981
FTL_0214	1431.531	-0.38794	0.093469	-4.15044	3.32E-05	0.000139
FTL_0215	1015.885	-0.46167	0.103843	-4.4459	8.75E-06	3.90E-05
FTL_0216	101.8531	-0.22822	0.249624	-0.91424	0.360593	0.484528
FTL_0217	1145.962	-0.30646	0.102863	-2.97931	0.002889	0.008319
FTL_0218	5280.642	0.350794	0.061187	5.733108	9.86E-09	6.08E-08
FTL_0219	832.2712	0.645765	0.113975	5.66586	1.46E-08	8.8E-08
FTL_0220	173.608	0.24855	0.206127	1.205812	0.22789	0.337553
FTL_0221	1443.278	-0.93602	0.091001	-10.2858	8.17E-25	1.55E-23
FTL_0222	5774.62	0.03011	0.059853	0.503064	0.614919	0.71693
FTL_0223	1237.884	0.021659	0.095278	0.227323	0.820173	0.875416
FTL_0224	22186.09	0.216156	0.057031	3.790146	0.000151	0.000561
FTL_0225	10780.23	0.135271	0.066247	2.041916	0.04116	0.082566
FTL_0226	9574.833	0.067155	0.054928	1.22261	0.221477	0.328781
FTL_0227	2128.377	-0.34006	0.080913	-4.20282	2.64E-05	0.000112
FTL_0228	2360.575	0.144838	0.078376	1.847977	0.064606	0.121023
FTL_0229	842.9358	0.071298	0.112013	0.636518	0.524439	0.642116
FTL_0230	785.6443	-0.08526	0.113451	-0.75156	0.452318	0.576146
FTL_0231	829.6171	-0.13191	0.118124	-1.11675	0.264103	0.379177
FTL_0232	11784.34	0.077589	0.061472	1.262174	0.206886	0.311959
FTL_0233	5663.657	-0.07785	0.068712	-1.13294	0.257238	0.37305
FTL_0234	41860.98	0.012793	0.049481	0.258546	0.795985	0.861103
FTL_0235	6300.215	-0.1224	0.05932	-2.06345	0.03907	0.079085
FTL_0236	23945.35	-0.0721	0.050631	-1.42403	0.154437	0.246756
FTL_0237	17646.66	-0.10473	0.051972	-2.01514	0.04389	0.087173
FTL_0238	8064.458	-0.12815	0.05891	-2.17535	0.029604	0.062504
FTL_0239	16680.7	-0.15307	0.053135	-2.88072	0.003968	0.01103
FTL_0240	10355.28	-0.02325	0.060743	-0.38274	0.701913	0.79039
FTL_0241	15487.93	0.01728	0.062719	0.275518	0.782918	0.85109
FTL_0242	19760.46	0.042562	0.054945	0.77463	0.438558	0.562901
FTL_0243	17635.38	0.060696	0.056422	1.075744	0.282042	0.399509
FTL_0244	9517.233	0.185104	0.075654	2.446712	0.014417	0.033695
FTL_0245	4705.637	0.033098	0.067537	0.490078	0.624079	0.72258
FTL_0246	10669.19	0.100004	0.055383	1.805681	0.070968	0.131229
FTL_0247	16850.89	0.059398	0.054919	1.081545	0.279455	0.396609
FTL_0248	21772.04	-0.0062	0.07048	-0.08793	0.929935	0.949781
FTL_0249	18215.08	0.029514	0.052483	0.562343	0.573882	0.686201
FTL_0250	21497.65	0.150503	0.054043	2.784848	0.005355	0.014215

FTL_0251	17482.82	0.125872	0.053824	2.33858	0.019357	0.043766
FTL_0252	13147.82	0.123114	0.057537	2.139739	0.032376	0.067227
FTL_0253	23597.29	0.104537	0.050929	2.052601	0.040111	0.080866
FTL_0254	15803.62	0.047237	0.057648	0.8194	0.412559	0.537422
FTL_0255	10763.44	0.063298	0.057485	1.101116	0.270846	0.386648
FTL_0256	26509.34	0.008624	0.056475	0.152699	0.878636	0.918379
FTL_0257	1557.974	-0.19283	0.092566	-2.08315	0.037238	0.07622
FTL_0258	10303.34	-0.09914	0.062388	-1.58904	0.112052	0.189264
FTL_0259	10993.14	-0.09893	0.061912	-1.59788	0.11007	0.186508
FTL_0260	14943.94	-0.11676	0.056399	-2.07031	0.038423	0.078327
FTL_0261	18351.71	-0.10706	0.059765	-1.79142	0.073225	0.134384
FTL_0262	10093.13	-0.23011	0.072174	-3.18822	0.001432	0.004427
FTL_0263	1568.611	0.033123	0.094859	0.349178	0.726956	0.809394
FTL_0264	306.5614	0.18835	0.171205	1.100143	0.27127	0.386978
FTL_0265	4393.782	-0.77347	0.077592	-9.96844	2.09E-23	3.66E-22
FTL_0266	909.6822	0.203895	0.104674	1.947909	0.051426	0.099967
FTL_0267	38554.71	-0.51318	0.050188	-10.2253	1.53E-24	2.84E-23
FTL_0268	1226.604	0.109486	0.09702	1.128482	0.259117	0.375234
FTL_0269	23562.24	0.476407	0.061347	7.765817	8.11E-15	8.28E-14
FTL_0270	130.9315	0.14006	0.223139	0.627681	0.530213	0.647465
FTL_0271	301.3746	0.528042	0.164506	3.209855	0.001328	0.004151
FTL_0272	373.1504	0.668985	0.149671	4.469706	7.83E-06	3.51E-05
FTL_0273	661.6393	0.327447	0.118412	2.765326	0.005687	0.014941
FTL_0274	308.1671	0.000994	0.172541	0.005762	0.995402	0.99689
FTL_0275	239.0279	-0.12777	0.187209	-0.68249	0.49493	0.61667
FTL_0276	169.0394	-0.22837	0.205244	-1.11265	0.265858	0.381424
FTL_0277	775.1927	-0.312	0.115022	-2.71252	0.006677	0.017141
FTL_0278	246.9984	-0.37117	0.179031	-2.0732	0.038154	0.077857
FTL_0279	447.1861	-0.71799	0.140216	-5.12057	3.05E-07	1.59E-06
FTL_0280	624.1023	-0.6184	0.136574	-4.52796	5.96E-06	2.70E-05
FTL_0281	2488.945	-0.45602	0.088613	-5.14615	2.66E-07	1.40E-06
FTL_0282	2515.83	0.189008	0.075758	2.494889	0.0126	0.030006
FTL_0283	868.0239	1.162392	0.118095	9.84285	7.36E-23	1.22E-21
FTL_0284	479.9692	0.134563	0.134565	0.99999	0.317316	0.440472
FTL_0285	16430.17	0.122575	0.07469	1.641114	0.100774	0.173272
FTL_0286	824.7074	0.481899	0.110655	4.354985	1.33E-05	5.79E-05
FTL_0287	289.4509	0.365059	0.165685	2.203331	0.027571	0.059271
FTL_0288	249.3672	0.303627	0.176418	1.721071	0.085238	0.15095
FTL_0289	801.712	-0.01024	0.109661	-0.09337	0.925607	0.948002
FTL_0290	1241.522	-0.34048	0.094541	-3.60141	0.000316	0.001122
FTL_0291	1257.91	-0.04615	0.093029	-0.49612	0.619811	0.719779
FTL_0292	429.0773	0.522738	0.141859	3.684917	0.000229	0.00083
FTL_0293	4716.985	-0.2249	0.065263	-3.44607	0.000569	0.001918

FTL_0294	3346.343	0.226323	0.068898	3.284887	0.00102	0.00325
FTL_0295	12273.23	0.120835	0.060603	1.993865	0.046167	0.090976
FTL_0296	2856.631	0.199748	0.073886	2.703446	0.006862	0.017549
FTL_0297	1805.805	0.439154	0.089501	4.906709	9.26E-07	4.57E-06
FTL_0298	1173.226	0.343735	0.095169	3.611838	0.000304	0.001083
FTL_0299	981.1457	0.240249	0.108912	2.205896	0.027391	0.058947
FTL_0300	669.7242	-0.17047	0.117432	-1.45168	0.146591	0.236127
FTL_0301	123.1712	0.480063	0.234841	2.044204	0.040933	0.082276
FTL_0302	122.9038	0.083801	0.230175	0.364077	0.715801	0.801537
FTL_0303	238.0614	0.400828	0.205153	1.953799	0.050725	0.0987
FTL_0304	1812.062	0.081484	0.086707	0.939763	0.347339	0.469503
FTL_0305	1977.708	0.216701	0.08561	2.531254	0.011366	0.027458
FTL_0306	5911.734	0.290656	0.063837	4.553073	5.29E-06	2.40E-05
FTL_0307	6422.75	-0.31872	0.059614	-5.34637	8.97E-08	4.98E-07
FTL_0308	670.3213	-0.01563	0.118532	-0.1319	0.895062	0.927836
FTL_0309	25168.66	0.470976	0.052519	8.96774	3.03E-19	4.00E-18
FTL_0310	11037.65	0.414945	0.057006	7.278901	3.37E-13	3.03E-12
FTL_0311	16938.27	0.375532	0.056059	6.698863	2.10E-11	1.70E-10
FTL_0312	2185.804	0.35867	0.088009	4.075373	4.59E-05	0.000187
FTL_0313	246.7004	0.465567	0.176548	2.637054	0.008363	0.020856
FTL_0314	969.8627	0.233013	0.103351	2.254568	0.02416	0.052843
FTL_0315	965.6742	-0.11443	0.103752	-1.10296	0.270043	0.38605
FTL_0316	3144.788	-0.31408	0.079131	-3.9691	7.21E-05	0.000283
FTL_0317	7375.871	-0.91515	0.065047	-14.069	5.89E-45	2.52E-43
FTL_0318	620.2958	0.079159	0.12233	0.647089	0.517574	0.638236
FTL_0319	1988.572	-0.01325	0.082378	-0.16088	0.872188	0.914501
FTL_0320	499.6526	0.153895	0.133223	1.155175	0.248019	0.361507
FTL_0321	17.65331	0.411822	0.333049	1.236519	0.216266	0.321999
FTL_0322	218.1578	-0.00422	0.188945	-0.02236	0.982164	0.987075
FTL_0323	171.5484	-0.28947	0.210139	-1.37753	0.16835	0.264982
FTL_0324	694.2336	-0.63274	0.125546	-5.03994	4.66E-07	2.37E-06
FTL_0325	7303.093	-0.53236	0.063075	-8.44009	3.17E-17	3.76E-16
FTL_0326	1632.697	0.156808	0.089461	1.75281	0.079635	0.143686
FTL_0327	7484.581	0.467854	0.062779	7.452364	9.17E-14	8.78E-13
FTL_0328	1374.908	0.396365	0.09355	4.236919	2.27E-05	9.65E-05
FTL_0329	2170.996	0.267407	0.080355	3.327815	0.000875	0.002851
FTL_0330	696.3123	-0.02057	0.122049	-0.16851	0.866185	0.911535
FTL_0331	2867.098	-0.05927	0.074795	-0.79243	0.428109	0.55246
FTL_0332	1596.227	-0.37435	0.085691	-4.36859	1.25E-05	5.45E-05
FTL_0333	1511.419	-0.1621	0.091341	-1.77464	0.075958	0.138146
FTL_0334	2380.991	-0.23856	0.084032	-2.83896	0.004526	0.01231
FTL_0335	600.9322	-0.21383	0.133383	-1.60314	0.108904	0.18488
FTL_0336	3010.769	-0.23775	0.072552	-3.27699	0.001049	0.003337

FTL_0337	11180.38	0.250637	0.05498	4.558687	5.15E-06	2.35E-05
FTL_0338	778.5796	0.155221	0.126501	1.227035	0.21981	0.32703
FTL_0339	1032.642	0.23558	0.100613	2.341439	0.01921	0.043555
FTL_0340	133.9446	0.496147	0.227009	2.185579	0.028846	0.061486
FTL_0341	217.2134	0.563032	0.188016	2.994595	0.002748	0.007959
FTL_0342	99.2741	1.018993	0.252421	4.036886	5.42E-05	0.000217
FTL_0343	281.0679	1.382128	0.174869	7.903803	2.71E-15	2.88E-14
FTL_0344	49.7903	0.62183	0.30149	2.062522	0.039158	0.079183
FTL_0345	710.5928	-0.07662	0.114917	-0.66675	0.504935	0.627122
FTL_0346	287.2957	0.183902	0.166298	1.105858	0.268788	0.384803
FTL_0347	297.4706	0.124967	0.165378	0.755646	0.449861	0.573745
FTL_0348	186.4552	-1.72656	0.204573	-8.43981	3.18E-17	3.76E-16
FTL_0349	109.1567	-0.67807	0.247299	-2.7419	0.006108	0.015945
FTL_0350	155.9905	0.109613	0.213778	0.512743	0.608131	0.711907
FTL_0351	226.6808	0.362735	0.182031	1.992712	0.046293	0.091135
FTL_0352	62.11636	0.066889	0.283315	0.236094	0.81336	0.871901
FTL_0353	879.8922	0.183332	0.106376	1.723431	0.084811	0.150591
FTL_0354	458.4461	-0.47539	0.140172	-3.39146	0.000695	0.002298
FTL_0355	643.836	-0.35306	0.119254	-2.96056	0.003071	0.008768
FTL_0356	755.6212	-0.06369	0.125735	-0.50655	0.612469	0.714488
FTL_0357	3450.637	0.115328	0.082644	1.395486	0.162869	0.25838
FTL_0358	1966.834	0.265367	0.079805	3.325213	0.000884	0.002874
FTL_0359	2160.765	0.430673	0.085961	5.010099	5.44E-07	2.75E-06
FTL_0360	3680.654	-0.603	0.068559	-8.79533	1.43E-18	1.81E-17
FTL_0361	2246.098	-0.32514	0.090876	-3.57786	0.000346	0.001209
FTL_0362	400.6246	-0.06397	0.145312	-0.44024	0.659761	0.75147
FTL_0363	514.0814	-0.01937	0.137706	-0.1407	0.888111	0.923966
FTL_0364	221.0703	0.630407	0.184833	3.410684	0.000648	0.002164
FTL_0365	423.5215	0.625086	0.153504	4.072107	4.66E-05	0.00019
FTL_0366	166.0051	0.844675	0.21581	3.91398	9.08E-05	0.000348
FTL_0367	154.4061	0.237205	0.213549	1.110776	0.266665	0.382036
FTL_0368	531.657	1.121424	0.132011	8.494926	1.98E-17	2.38E-16
FTL_0369	228.4613	0.679367	0.196852	3.451157	0.000558	0.001889
FTL_0370	158.9521	0.719429	0.210524	3.417318	0.000632	0.002119
FTL_0371	720.9782	1.227484	0.124658	9.846806	7.08E-23	1.19E-21
FTL_0372	2044.91	-0.20131	0.078955	-2.54975	0.01078	0.026233
FTL_0373	876.3197	-0.24913	0.1067	-2.33483	0.019552	0.044158
FTL_0374	1782.653	-0.26975	0.088126	-3.06093	0.002207	0.006541
FTL_0375	2605.601	-0.08278	0.073162	-1.13145	0.257866	0.373693
FTL_0376	1338.735	-0.11858	0.090144	-1.3155	0.188342	0.292556
FTL_0377	3488.135	-0.1673	0.070666	-2.36748	0.01791	0.041114
FTL_0378	319.0668	-0.34361	0.160762	-2.13738	0.032567	0.067484
FTL_0379	418.69	-0.36355	0.143324	-2.53657	0.011195	0.027142

FTL_0380	2222.586	0.345342	0.092656	3.727163	0.000194	0.000712
FTL_0381	4302.15	0.640286	0.065559	9.766587	1.57E-22	2.54E-21
FTL_0382	21027.79	0.644472	0.055586	11.59413	4.41E-31	1.04E-29
FTL_0383	1229.394	-0.08066	0.100422	-0.80326	0.421826	0.547366
FTL_0384	666.3025	-0.57372	0.119563	-4.79844	1.60E-06	7.69E-06
FTL_0385	643.2544	-0.42005	0.121539	-3.45612	0.000548	0.001861
FTL_0386	1160.753	-0.2656	0.106512	-2.49359	0.012646	0.030045
FTL_0387	10910.2	-0.65639	0.064879	-10.1172	4.64E-24	8.47E-23
FTL_0388	234.0564	-0.19043	0.191739	-0.99317	0.320627	0.442361
FTL_0389	2660.399	0.369295	0.077738	4.750525	2.03E-06	9.69E-06
FTL_0390	2053.508	0.581962	0.081884	7.107129	1.18E-12	1.04E-11
FTL_0391	1079.619	0.350682	0.106593	3.289911	0.001002	0.003208
FTL_0392	15611.91	-0.05486	0.055236	-0.99311	0.320657	0.442361
FTL_0393	1239.482	-0.00013	0.093195	-0.00143	0.998858	0.999003
FTL_0394	4748.549	-0.17143	0.066617	-2.57329	0.010074	0.024663
FTL_0395	5065.585	-0.65191	0.066068	-9.8673	5.77E-23	9.83E-22
FTL_0396	9959.501	-0.74106	0.057178	-12.9606	2.05E-38	6.43E-37
FTL_0397	1136.363	-0.73065	0.100261	-7.28746	3.16E-13	2.86E-12
FTL_0398	1006.355	-0.53791	0.105926	-5.07813	3.81E-07	1.97E-06
FTL_0399	2814.017	-0.58621	0.071547	-8.19342	2.54E-16	2.82E-15
FTL_0400	233.8135	-0.29379	0.184803	-1.58974	0.111894	0.189264
FTL_0401	635.9271	0.108354	0.146322	0.740521	0.458984	0.581689
FTL_0402	1688.768	-0.02796	0.091844	-0.30444	0.760792	0.835624
FTL_0403	1041.842	0.127913	0.130438	0.980645	0.326768	0.448636
FTL_0404	2911.344	0.058771	0.070982	0.827978	0.407683	0.533144
FTL_0405	2106.031	0.011663	0.089642	0.13011	0.896479	0.928348
FTL_0406	1429.487	-0.13683	0.090118	-1.51837	0.12892	0.212227
FTL_0407	2962.985	-0.09125	0.070484	-1.29468	0.195432	0.298615
FTL_0408	758.5922	0.217414	0.11429	1.902307	0.057131	0.109053
FTL_0409	524.1847	0.379416	0.130126	2.915758	0.003548	0.009975
FTL_0410	1900.844	-0.16108	0.081542	-1.97538	0.048225	0.094385
FTL_0411	3057.521	-0.14529	0.075241	-1.931	0.053483	0.103266
FTL_0412	1534.826	-0.04714	0.086917	-0.54237	0.58756	0.696001
FTL_0413	2369.024	0.076569	0.077441	0.98873	0.322795	0.444396
FTL_0414	3870.488	0.086923	0.0657	1.32303	0.185825	0.289318
FTL_0415	273.7852	0.685216	0.173412	3.951386	7.77E-05	0.000302
FTL_0416	173.7774	0.201326	0.204834	0.98287	0.325672	0.447437
FTL_0417	846.6391	0.295183	0.108075	2.731283	0.006309	0.01632
FTL_0418	271.9292	-0.1749	0.168374	-1.03875	0.298923	0.418909
FTL_0419	3351.077	-0.06008	0.070905	-0.84739	0.39678	0.522969
FTL_0420	656.5412	-0.18223	0.119169	-1.52918	0.126219	0.208464
FTL_0421	15454.6	0.105411	0.061653	1.709757	0.087311	0.154078
FTL_0422	2039.08	-0.06276	0.094644	-0.66312	0.507254	0.628982

FTL_0423	2340.715	0.043323	0.077379	0.559883	0.575559	0.687797
FTL_0424	4930.183	0.184955	0.062252	2.971047	0.002968	0.008534
FTL_0425	3135.824	-0.05791	0.072313	-0.8009	0.423191	0.547957
FTL_0426	8480.511	0.333377	0.06346	5.253346	1.49E-07	8.05E-07
FTL_0427	3565.06	0.119426	0.071647	1.666873	0.09554	0.16612
FTL_0428	2946.251	0.03077	0.081821	0.376062	0.706871	0.794636
FTL_0429	2701.959	0.174621	0.075168	2.323068	0.020175	0.045209
FTL_0430	2327.784	0.072058	0.085096	0.846791	0.397111	0.523063
FTL_0431	394.2098	-0.2602	0.152881	-1.70199	0.088757	0.156218
FTL_0432	213.4423	-0.03631	0.188293	-0.19283	0.847089	0.896131
FTL_0433	848.304	-0.10723	0.108283	-0.99026	0.322048	0.443672
FTL_0434	993.1847	-0.25318	0.110361	-2.29414	0.021782	0.048432
FTL_0435	2969.711	-0.23273	0.070131	-3.31847	0.000905	0.00293
FTL_0436	18455.26	-0.2292	0.052872	-4.33495	1.46E-05	6.33E-05
FTL_0437	3097.882	-0.20072	0.071139	-2.82146	0.004781	0.012933
FTL_0438	22770.02	0.216459	0.053861	4.018865	5.85E-05	0.000233
FTL_0439	21142.83	0.177179	0.050763	3.490335	0.000482	0.001658
FTL_0440	1361.487	0.294378	0.094539	3.113839	0.001847	0.005565
FTL_0441	266.446	0.777336	0.181246	4.288854	1.80E-05	7.75E-05
FTL_0442	470.4857	1.385813	0.13955	9.930564	3.07E-23	5.31E-22
FTL_0443	3356.221	0.46605	0.071949	6.477467	9.33E-11	7.21E-10
FTL_0444	13977.19	0.255532	0.054882	4.656065	3.22E-06	1.49E-05
FTL_0445	820.6063	0.680744	0.119705	5.686828	1.29E-08	7.88E-08
FTL_0446	614.9026	1.785338	0.129371	13.8001	2.54E-43	1.02E-41
FTL_0447	1258.57	1.044114	0.105999	9.850235	6.84E-23	1.16E-21
FTL_0448	1179.784	0.265458	0.095408	2.782347	0.005397	0.014292
FTL_0449	951.6534	-1.55971	0.105837	-14.7369	3.74E-49	1.93E-47
FTL_0450	4784.888	-0.00024	0.158583	-0.00153	0.998779	0.999003
FTL_0451	4277.393	0.935602	0.06762	13.83617	1.54E-43	6.46E-42
FTL_0452	3757.783	0.89096	0.069169	12.88083	5.77E-38	1.78E-36
FTL_0453	10391.63	0.736045	0.056675	12.98701	1.45E-38	4.63E-37
FTL_0454	11232.39	0.4851	0.061888	7.838321	4.57E-15	4.78E-14
FTL_0455	5471.018	0.569125	0.061818	9.206432	3.37E-20	4.81E-19
FTL_0456	3643.67	0.140835	0.078257	1.799654	0.071915	0.132493
FTL_0457	4910.953	0.091163	0.072732	1.253421	0.210052	0.314814
FTL_0458	230.9606	0.510316	0.190468	2.679275	0.007378	0.018749
FTL_0459	2206.545	0.743476	0.084879	8.759246	1.97E-18	2.45E-17
FTL_0460	2197.389	0.583141	0.084228	6.923342	4.41E-12	3.74E-11
FTL_0461	1699.837	0.367752	0.083978	4.379164	1.19E-05	5.25E-05
FTL_0462	7013.049	0.537971	0.061674	8.722869	2.71E-18	3.37E-17
FTL_0463	1774.369	0.281822	0.084109	3.350679	0.000806	0.002643
FTL_0464	1064.507	0.231635	0.102555	2.258634	0.023906	0.052458
FTL_0465	943.7848	0.611768	0.104587	5.849383	4.93E-09	3.14E-08

FTL_0466	3171.686	0.442231	0.06938	6.373998	1.84E-10	1.37E-09
FTL_0467	314.4748	0.684131	0.160971	4.250031	2.14E-05	9.14E-05
FTL_0468	223.5311	0.407106	0.185104	2.199335	0.027854	0.059751
FTL_0469	175.7627	1.190654	0.205268	5.800476	6.61E-09	4.14E-08
FTL_0470	1018.262	-0.03588	0.105693	-0.33943	0.734287	0.814524
FTL_0471	179.5664	0.73193	0.203758	3.59216	0.000328	0.001158
FTL_0472	7606.689	0.467497	0.061248	7.632789	2.30E-14	2.26E-13
FTL_0473	569.0614	-0.406	0.126067	-3.22052	0.00128	0.004012
FTL_0474	3727.949	0.755048	0.072916	10.35505	3.97E-25	7.90E-24
FTL_0475	1772.171	0.741486	0.087385	8.485249	2.15E-17	2.58E-16
FTL_0476	6043.277	0.525014	0.062377	8.416747	3.87E-17	4.52E-16
FTL_0477	6991.982	0.214662	0.069833	3.073955	0.002112	0.0063
FTL_0478	3283.062	0.331215	0.070365	4.707085	2.51E-06	1.19E-05
FTL_0479	15615.38	0.093802	0.05469	1.715157	0.086316	0.152591
FTL_0480	9077.33	0.117542	0.062025	1.895063	0.058084	0.110349
FTL_0481	2994.876	0.439535	0.070976	6.192732	5.91E-10	4.11E-09
FTL_0482	10145.67	0.86575	0.058158	14.88618	4.05E-50	2.20E-48
FTL_0483	10333.46	0.794273	0.057542	13.80339	2.43E-43	9.97E-42
FTL_0484	3423.722	0.567295	0.067898	8.35516	6.53E-17	7.42E-16
FTL_0485	5152.661	0.601475	0.063626	9.453299	3.28E-21	4.89E-20
FTL_0486	3932.512	0.520241	0.06515	7.985282	1.40E-15	1.51E-14
FTL_0487	8371.949	0.734175	0.060546	12.12586	7.70E-34	2.12E-32
FTL_0488	2526.118	0.518734	0.074047	7.005483	2.46E-12	2.11E-11
FTL_0489	3655.475	0.912394	0.073964	12.33561	5.82E-35	1.67E-33
FTL_0490	2352.969	0.810688	0.075504	10.73708	6.82E-27	1.46E-25
FTL_0491	1328.224	-0.19129	0.091946	-2.08041	0.037488	0.076653
FTL_0492	6095.056	0.700554	0.06019	11.63894	2.61E-31	6.33E-30
FTL_0493	3325.95	0.561935	0.069642	8.068915	7.09E-16	7.66E-15
FTL_0494	526.0413	0.789307	0.136946	5.76366	8.23E-09	5.11E-08
FTL_0495	53.73295	1.367008	0.302816	4.514315	6.35E-06	2.86E-05
FTL_0496	9.908036	0.728691	0.314478	2.317144	0.020496	0.045774
FTL_0497	13.2485	0.865568	0.32543	2.659771	0.007819	0.019729
FTL_0498	126.9068	0.990794	0.239285	4.140653	3.46E-05	0.000144
FTL_0499	1129.312	0.835297	0.103133	8.099251	5.53E-16	6.04E-15
FTL_0500	970.0915	1.399974	0.105304	13.29455	2.49E-40	8.48E-39
FTL_0501	167.9673	1.318254	0.210192	6.271659	3.57E-10	2.57E-09
FTL_0502	249.9751	1.168185	0.180018	6.489259	8.63E-11	6.69E-10
FTL_0503	847.784	0.27563	0.10814	2.548826	0.010809	0.02627
FTL_0504	13.00985	0.212887	0.328088	0.64887	0.516422	0.63743
FTL_0505	92.79914	0.721866	0.260435	2.77177	0.005575	0.014706
FTL_0506	683.8559	0.237017	0.122708	1.931554	0.053415	0.103234
FTL_0507	1246.016	0.405942	0.098268	4.130951	3.61E-05	0.00015
FTL_0508	4006.451	0.636842	0.070651	9.013964	1.99E-19	2.66E-18

FTL_0509	1128.682	0.730434	0.102368	7.135357	9.65E-13	8.51E-12
FTL_0510	1280.035	0.380147	0.093903	4.048287	5.16E-05	0.000208
FTL_0511	1700.255	1.090472	0.090854	12.00249	3.45E-33	9.08E-32
FTL_0512	645.4775	0.692978	0.137639	5.034739	4.79E-07	2.42E-06
FTL_0513	873.7174	0.883971	0.112132	7.883277	3.19E-15	3.36E-14
FTL_0514	4348.816	0.737703	0.067969	10.85357	1.92E-27	4.24E-26
FTL_0515	1466.595	0.419045	0.09058	4.626267	3.72E-06	1.72E-05
FTL_0516	1545.915	0.57547	0.087821	6.552728	5.65E-11	4.44E-10
FTL_0517	3047.059	0.503404	0.073778	6.823201	8.90E-12	7.39E-11
FTL_0518	2298.26	0.428808	0.091711	4.67567	2.93E-06	1.37E-05
FTL_0519	10387.13	0.200634	0.060605	3.310509	0.000931	0.003
FTL_0520	16691.42	0.464237	0.060438	7.681264	1.58E-14	1.59E-13
FTL_0521	12752.92	0.585246	0.059876	9.774226	1.45E-22	2.37E-21
FTL_0522	12579.06	0.539829	0.056808	9.502608	2.05E-21	3.09E-20
FTL_0523	3263.575	0.467426	0.070973	6.585945	4.52E-11	3.56E-10
FTL_0524	1585.259	0.473841	0.085256	5.557834	2.73E-08	1.60E-07
FTL_0525	9357.388	0.714387	0.059523	12.0018	3.48E-33	9.08E-32
FTL_0526	1056.62	0.703006	0.103277	6.806991	9.97E-12	8.21E-11
FTL_0527	236.4819	1.192039	0.184536	6.459648	1.05E-10	8.02E-10
FTL_0528	2951.434	1.010736	0.074225	13.6172	3.16E-42	1.20E-40
FTL_0529	413.4825	0.53687	0.143966	3.72914	0.000192	0.000709
FTL_0530	75.23702	0.493067	0.270514	1.822704	0.068348	0.126617
FTL_0531	41.20905	1.529784	0.317622	4.816369	1.46E-06	7.06E-06
FTL_0532	173.5728	0.766639	0.224229	3.418999	0.000629	0.002113
FTL_0533	33861.29	0.877388	0.051106	17.16807	4.61E-66	3.70E-64
FTL_0534	6493.463	0.751698	0.06928	10.85008	1.99E-27	4.35E-26
FTL_0535	9070.438	0.825635	0.05662	14.58208	3.65E-48	1.67E-46
FTL_0536	4730.679	0.743917	0.064764	11.48655	1.54E-30	3.60E-29
FTL_0537	9378.023	0.758357	0.055923	13.56075	6.84E-42	2.46E-40
FTL_0538	8752.011	0.702081	0.059031	11.89341	1.28E-32	3.26E-31
FTL_0539	17259.89	0.517161	0.051806	9.982675	1.82E-23	3.23E-22
FTL_0540	10964	0.58619	0.055574	10.54797	5.19E-26	1.08E-24
FTL_0541	2437.558	0.633286	0.075825	8.351962	6.71E-17	7.58E-16
FTL_0542	1769.811	0.887457	0.091781	9.66931	4.07E-22	6.44E-21
FTL_0543	1433.834	1.008453	0.097409	10.35274	4.07E-25	8.01E-24
FTL_0544	3284.942	0.879195	0.072777	12.08068	1.34E-33	3.63E-32
FTL_0545	1870.763	0.636047	0.093103	6.831645	8.39E-12	7.00E-11
FTL_0546	1620.29	0.647611	0.090533	7.15334	8.47E-13	7.57E-12
FTL_0547	3343.192	0.492882	0.068988	7.144503	9.03E-13	8.03E-12
FTL_0548	479.3623	0.474631	0.139572	3.400615	0.000672	0.00223
FTL_0549	987.1506	0.684115	0.10364	6.600902	4.09E-11	3.25E-10
FTL_0550	78.63146	-0.15324	0.265449	-0.57727	0.563758	0.676107
FTL_0551	421.7389	0.509537	0.142014	3.58794	0.000333	0.001173

FTL_0552	5865.389	0.26084	0.068352	3.816106	0.000136	0.00051
FTL_0553	4360.744	0.268584	0.064855	4.141313	3.45E-05	0.000144
FTL_0554	1934.026	-0.03539	0.086611	-0.40865	0.682798	0.772761
FTL_0555	1566.762	0.288734	0.085769	3.366402	0.000762	0.002501
FTL_0556	8007.289	0.29475	0.06073	4.853416	1.21E-06	5.95E-06
FTL_0557	932.1258	0.62196	0.104934	5.927139	3.08E-09	2.02E-08
FTL_0558	258.1208	1.04205	0.177346	5.875788	4.21E-09	2.71E-08
FTL_0559	142.597	1.421302	0.225952	6.290273	3.17E-10	2.29E-09
FTL_0560	95.59872	1.699047	0.262654	6.46877	9.88E-11	7.61E-10
FTL_0561	176.2121	1.208509	0.207646	5.820042	5.88E-09	3.73E-08
FTL_0562	174.1856	1.122745	0.215528	5.20928	1.90E-07	1.01E-06
FTL_0563	144.524	1.485146	0.22484	6.605339	3.97E-11	3.16E-10
FTL_0564	98.62922	1.295468	0.254735	5.085557	3.67E-07	1.90E-06
FTL_0565	86.42074	1.436719	0.269393	5.333169	9.65E-08	5.3E-07
FTL_0566	300.7154	1.21448	0.172206	7.052473	1.76E-12	1.52E-11
FTL_0567	122.3123	0.708438	0.237276	2.985717	0.002829	0.008159
FTL_0568	178.3235	0.383188	0.20137	1.90291	0.057052	0.109007
FTL_0569	2970.506	-0.11839	0.071511	-1.65558	0.097806	0.169328
FTL_0570	713.4755	-0.15175	0.116259	-1.30525	0.191809	0.295204
FTL_0571	2715.887	0.70267	0.077135	9.109616	8.27E-20	1.15E-18
FTL_0572	8717.263	0.718962	0.058023	12.39091	2.93E-35	8.53E-34
FTL_0573	4741.075	0.699183	0.063368	11.03364	2.63E-28	5.87E-27
FTL_0574	3753.121	0.918021	0.066563	13.79178	2.86E-43	1.13E-41
FTL_0575	509.0544	-0.03033	0.136003	-0.22298	0.823555	0.876772
FTL_0576	122.5018	0.990344	0.233276	4.245368	2.18E-05	9.31E-05
FTL_0577	96.33439	1.05045	0.256379	4.097259	4.18E-05	0.000171
FTL_0578	7527.428	0.701483	0.060449	11.60463	3.90E-31	9.34E-30
FTL_0579	2137.893	0.628668	0.085752	7.3312	2.28E-13	2.09E-12
FTL_0580	1801.413	0.349211	0.093798	3.722997	0.000197	0.000721
FTL_0581	2428.689	0.438932	0.082429	5.32499	1.01E-07	5.53E-07
FTL_0582	2332.529	0.214734	0.075231	2.854331	0.004313	0.011851
FTL_0583	7056.096	0.285841	0.061337	4.660176	3.16E-06	1.46E-05
FTL_0584	23337.58	0.271479	0.052878	5.134068	2.84E-07	1.49E-06
FTL_0585	19661.24	0.183849	0.054509	3.372815	0.000744	0.002452
FTL_0586	5379.408	0.971972	0.06191	15.69981	1.52E-55	9.53E-54
FTL_0587	634.8702	0.738795	0.122777	6.017379	1.77E-09	1.18E-08
FTL_0588	26986.22	0.819191	0.052311	15.66006	2.84E-55	1.73E-53
FTL_0589	7319.489	0.605405	0.0588	10.29593	7.35E-25	1.41E-23
FTL_0590	7935.239	0.792831	0.058	13.66957	1.54E-42	5.96E-41
FTL_0591	124.3312	0.203048	0.228025	0.890464	0.373217	0.498449
FTL_0592	7801.187	-0.00663	0.05817	-0.11398	0.909253	0.93627
FTL_0593	3539.72	-0.26543	0.068137	-3.89555	9.80E-05	0.000374
FTL_0594	3786.722	-0.4853	0.066055	-7.3469	2.03E-13	1.87E-12

FTL_0595	4898.199	-0.65177	0.063028	-10.3409	4.60E-25	8.89E-24
FTL_0596	10857.44	-0.3789	0.054086	-7.00559	2.46E-12	2.11E-11
FTL_0597	7283.22	-0.35614	0.064472	-5.5239	3.32E-08	1.92E-07
FTL_0598	1079.205	-0.61007	0.116509	-5.23622	1.64E-07	8.78E-07
FTL_0599	1317.579	-1.07762	0.104003	-10.3615	3.71E-25	7.46E-24
FTL_0600	5627.171	-0.9068	0.061551	-14.7324	3.99E-49	2.00E-47
FTL_0601	4390.266	-0.4828	0.069597	-6.93712	4.00E-12	3.41E-11
FTL_0602	3326.878	-0.62641	0.069621	-8.99736	2.31E-19	3.08E-18
FTL_0603	3151.492	-0.683	0.075753	-9.0161	1.95E-19	2.63E-18
FTL_0604	3824.945	-0.51847	0.067726	-7.65548	1.93E-14	1.92E-13
FTL_0605	3937.142	-0.3264	0.074596	-4.37562	1.21E-05	5.33E-05
FTL_0606	4296.756	-0.47374	0.064968	-7.29198	3.05E-13	2.78E-12
FTL_0607	1066.063	0.117898	0.09825	1.199982	0.230146	0.340393
FTL_0608	2511.226	0.125613	0.077737	1.615866	0.106123	0.18123
FTL_0609	1721.738	0.037323	0.083632	0.446278	0.655397	0.74816
FTL_0610	13908.45	-0.53104	0.054501	-9.74375	1.96E-22	3.15E-21
FTL_0611	4440.657	-0.21977	0.066612	-3.29927	0.000969	0.003108
FTL_0612	1338.782	0.154584	0.09003	1.717028	0.085974	0.15212
FTL_0613	456.7435	0.10829	0.138578	0.781435	0.434547	0.558822
FTL_0614	87.81764	0.902939	0.259349	3.481563	0.000498	0.001707
FTL_0615	356.1229	1.450298	0.167621	8.652251	5.05E-18	6.23E-17
FTL_0616	14659.66	-0.0887	0.061333	-1.44626	0.148104	0.23796
FTL_0617	25770.09	0.363165	0.058208	6.239081	4.40E-10	3.13E-09
FTL_0618	125.4857	-0.22586	0.236781	-0.95389	0.340138	0.462569
FTL_0619	184.7306	-0.10764	0.201958	-0.53297	0.594055	0.700733
FTL_0620	1000.141	0.34628	0.100302	3.452362	0.000556	0.001884
FTL_0621	1514.347	0.355988	0.09311	3.823296	0.000132	0.000498
FTL_0622	293.1671	-0.06125	0.164285	-0.37285	0.709261	0.796432
FTL_0623	952.5913	0.195218	0.104842	1.862015	0.062601	0.117596
FTL_0624	1168.683	0.250071	0.096564	2.589684	0.009606	0.023634
FTL_0625	2142.047	-0.05088	0.080772	-0.62997	0.528714	0.646421
FTL_0626	670.8691	0.096663	0.12558	0.769731	0.44146	0.565181
FTL_0627	579.5643	-0.32998	0.124187	-2.65708	0.007882	0.019828
FTL_0628	737.0429	-0.03225	0.114936	-0.2806	0.779019	0.847768
FTL_0629	697.4623	-0.163	0.119247	-1.3669	0.171658	0.269346
FTL_0630	279.9715	0.019147	0.171304	0.111773	0.911003	0.937112
FTL_0631	839.8095	0.233815	0.110655	2.113017	0.034599	0.071474
FTL_0632	159.2572	1.221644	0.216379	5.645861	1.64E-08	9.81E-08
FTL_0633	219.4739	1.834364	0.195301	9.392475	5.86E-21	8.54E-20
FTL_0634	104.0531	1.57034	0.253873	6.185525	6.19E-10	4.29E-09
FTL_0635	182.2773	1.251388	0.221788	5.642267	1.68E-08	9.95E-08
FTL_0636	180.7581	0.808923	0.222585	3.634216	0.000279	0.000997
FTL_0637	942.5453	-0.18331	0.103053	-1.77883	0.075268	0.13741

FTL_0638	827.8228	-0.14326	0.110102	-1.30118	0.193197	0.297113
FTL_0639	1926.714	-0.02012	0.082584	-0.24363	0.807521	0.869372
FTL_0640	1446.44	-0.04131	0.088506	-0.46674	0.640684	0.737557
FTL_0641	110.1439	0.365741	0.23903	1.530103	0.125991	0.208259
FTL_0642	76.09229	0.480402	0.275252	1.745318	0.08093	0.14563
FTL_0643	944.1375	-0.03639	0.104043	-0.3498	0.726487	0.809394
FTL_0644	3277.614	0.092222	0.070997	1.298965	0.193956	0.297808
FTL_0645	15909.81	0.246243	0.052249	4.712914	2.44E-06	1.16E-05
FTL_0646	3112.689	0.753399	0.092061	8.183707	2.75E-16	3.04E-15
FTL_0647	148.1267	1.035839	0.221361	4.679401	2.88E-06	1.35E-05
FTL_0648	904.9603	0.25977	0.104201	2.492967	0.012668	0.030062
FTL_0649	1701.095	-0.06746	0.098027	-0.68815	0.491357	0.612672
FTL_0650	8161.441	0.376523	0.05898	6.383905	1.73E-10	1.29E-09
FTL_0651	101.9855	0.541635	0.245384	2.207297	0.027293	0.058799
FTL_0652	1241.708	0.143229	0.095486	1.500003	0.133613	0.219056
FTL_0653	758.1234	0.000997	0.114017	0.00874	0.993027	0.995503
FTL_0654	1008.677	0.158267	0.106284	1.489093	0.136463	0.222458
FTL_0655	1926.931	-0.0095	0.079503	-0.11945	0.904922	0.932533
FTL_0656	1870.297	-0.30256	0.083712	-3.61429	0.000301	0.001075
FTL_0657	1186.274	-0.2881	0.102897	-2.79992	0.005111	0.013699
FTL_0659	1074.063	-0.23584	0.09963	-2.36711	0.017928	0.041114
FTL_0660	353.5618	0.243902	0.160241	1.5221	0.127984	0.211032
FTL_0661	4488.709	0.041835	0.063011	0.663932	0.506734	0.628725
FTL_0662	1948.463	0.544333	0.079913	6.811587	9.65E-12	7.98E-11
FTL_0663	566.048	0.556086	0.138753	4.007752	6.13E-05	0.000244
FTL_0664	1303.362	0.659715	0.105974	6.225224	4.81E-10	3.39E-09
FTL_0665	1248.876	-0.14958	0.092505	-1.61699	0.10588	0.180968
FTL_0666	2273.155	-0.18013	0.077242	-2.33202	0.0197	0.044341
FTL_0667	369.6736	0.195414	0.156364	1.249739	0.211395	0.315979
FTL_0668	1194.099	-0.26399	0.094765	-2.7857	0.005341	0.014215
FTL_0669	2241.529	-0.04781	0.088627	-0.53941	0.589607	0.697038
FTL_0670	1144.445	-0.63941	0.104151	-6.13927	8.29E-10	5.69E-09
FTL_0671	11140.15	-0.88795	0.060783	-14.6084	2.48E-48	1.16E-46
FTL_0672	2844.644	-0.8553	0.079112	-10.8113	3.04E-27	6.57E-26
FTL_0673	15934.44	-0.9711	0.057665	-16.8403	1.24E-63	9.55E-62
FTL_0674	9558.315	-0.95272	0.060017	-15.8741	9.58E-57	6.21E-55
FTL_0675	5258.143	-1.07153	0.063927	-16.7618	4.64E-63	3.46E-61
FTL_0676	3471.852	0.205937	0.069919	2.945381	0.003226	0.009157
FTL_0677	340.9703	0.248454	0.179092	1.387294	0.165352	0.260673
FTL_0678	993.0688	0.251052	0.104322	2.406518	0.016105	0.037252
FTL_0679	1920.761	0.249739	0.082402	3.030736	0.00244	0.007169
FTL_0680	3308.957	0.273652	0.073406	3.727953	0.000193	0.000711
FTL_0681	6571.384	-0.04614	0.059656	-0.77347	0.439245	0.563422

FTL_0682	819.5398	0.229104	0.11179	2.049419	0.040421	0.081328
FTL_0683	565.6774	0.634279	0.130717	4.852294	1.22E-06	5.97E-06
FTL_0684	1941.761	0.060139	0.081015	0.742321	0.457893	0.580968
FTL_0685	3666.593	-0.21028	0.067116	-3.13303	0.00173	0.005245
FTL_0686	8361.66	-0.1113	0.059909	-1.85786	0.063189	0.118591
FTL_0687	8627.936	-0.09565	0.058725	-1.6287	0.103376	0.177291
FTL_0688	6086.698	-0.03453	0.063039	-0.54773	0.583878	0.694024
FTL_0689	549.6147	-0.1264	0.126635	-0.99817	0.318197	0.441087
FTL_0690	2402.039	-0.008	0.077199	-0.10362	0.917473	0.941672
FTL_0691	6181.87	-0.80155	0.062861	-12.7511	3.07E-37	9.08E-36
FTL_0692	386.3864	-0.1902	0.159157	-1.19505	0.232067	0.342478
FTL_0693	543.0129	0.058176	0.132829	0.437979	0.661402	0.752785
FTL_0694	1856.535	-0.42549	0.084496	-5.03558	4.76E-07	2.42E-06
FTL_0695	926.3405	-0.38382	0.107192	-3.58071	0.000343	0.001201
FTL_0696	1273.58	-0.25516	0.096976	-2.63117	0.008509	0.021141
FTL_0697	1612.963	-0.08563	0.086942	-0.98489	0.324677	0.446375
FTL_0698	632.9238	-0.14949	0.122033	-1.22498	0.220584	0.327939
FTL_0699	1598.546	-0.25774	0.086042	-2.99552	0.00274	0.007947
FTL_0700	696.7808	-0.08588	0.119721	-0.71738	0.473141	0.595873
FTL_0701	1178.758	-0.08376	0.096812	-0.86518	0.386942	0.51303
FTL_0702	502.3997	0.100705	0.138788	0.725606	0.468081	0.590407
FTL_0703	11058	-0.10228	0.057137	-1.79015	0.073429	0.134542
FTL_0704	1149.882	-0.15	0.097757	-1.53444	0.124921	0.207
FTL_0705	1159.984	-0.12314	0.096436	-1.27691	0.201636	0.305647
FTL_0706	1015.203	-0.05315	0.115043	-0.46198	0.644098	0.739563
FTL_0707	2249.894	-0.07798	0.076996	-1.01271	0.311196	0.433475
FTL_0708	1197.494	-0.06287	0.099776	-0.63009	0.528635	0.646421
FTL_0709	939.7012	-0.039	0.102907	-0.37898	0.704704	0.792644
FTL_0710	256.1878	-0.08672	0.175928	-0.49291	0.622079	0.721095
FTL_0711	168.7372	-0.06425	0.2048	-0.3137	0.753745	0.830152
FTL_0712	99.10704	0.072992	0.24668	0.295898	0.767308	0.839387
FTL_0713	995.6184	-0.32391	0.103318	-3.13508	0.001718	0.005216
FTL_0714	4487.211	-0.51947	0.066475	-7.81455	5.52E-15	5.74E-14
FTL_0715	3313.219	-0.18478	0.07074	-2.61207	0.009	0.022184
FTL_0716	4240.833	0.185949	0.065491	2.839295	0.004521	0.01231
FTL_0717	17500.33	0.449802	0.055439	8.113444	4.92E-16	5.40E-15
FTL_0718	2623.095	0.322544	0.077222	4.176828	2.96E-05	0.000125
FTL_0719	428.0335	-0.07514	0.14227	-0.52816	0.597388	0.703018
FTL_0720	497.1114	0.030867	0.138239	0.223286	0.823313	0.876772
FTL_0721	1918.541	-0.08579	0.082222	-1.04334	0.296791	0.416877
FTL_0722	1236.41	-0.28478	0.093458	-3.04712	0.00231	0.006829
FTL_0723	1032.877	-0.05625	0.106505	-0.52816	0.59739	0.703018
FTL_0724	1952.586	0.245136	0.081694	3.000639	0.002694	0.007825

FTL_0725	877.8076	0.184204	0.119895	1.536382	0.124445	0.206552
FTL_0726	1629.319	-0.03144	0.088529	-0.35509	0.722525	0.806371
FTL_0727	1431.231	-0.07748	0.088655	-0.874	0.382117	0.507787
FTL_0728	212.6538	-0.5943	0.190053	-3.12703	0.001766	0.005345
FTL_0729	1149.298	-0.2232	0.102753	-2.17225	0.029837	0.062863
FTL_0730	2273.65	-0.03096	0.081252	-0.38106	0.703158	0.791348
FTL_0731	3627.643	-0.27559	0.068499	-4.02329	5.74E-05	0.000229
FTL_0732	1947.407	0.334843	0.08168	4.099443	4.14E-05	0.00017
FTL_0733	1031.317	0.216111	0.103503	2.087963	0.036801	0.075557
FTL_0734	478.5797	1.081596	0.136865	7.902667	2.73E-15	2.89E-14
FTL_0735	236.9525	0.41088	0.181592	2.262654	0.023657	0.052082
FTL_0736	2666.747	-0.27312	0.079978	-3.41492	0.000638	0.002134
FTL_0737	1574.584	-0.41491	0.11184	-3.70984	0.000207	0.000758
FTL_0738	874.514	0.190659	0.107112	1.779991	0.075077	0.137187
FTL_0739	11319.36	0.087473	0.054223	1.613215	0.106698	0.181748
FTL_0740	354.8788	0.191961	0.164044	1.170179	0.241929	0.355004
FTL_0741	566.678	0.029735	0.127085	0.233975	0.815004	0.872754
FTL_0742	277.747	-0.12472	0.171937	-0.72539	0.468213	0.590407
FTL_0743	1850.513	0.402679	0.081123	4.963781	6.91E-07	3.45E-06
FTL_0744	2441.86	-0.01164	0.075112	-0.15492	0.876883	0.91806
FTL_0745	2515.111	-0.22283	0.079089	-2.81741	0.004841	0.013062
FTL_0746	893.222	-0.05858	0.11266	-0.51996	0.603089	0.70724
FTL_0747	917.7152	-0.16559	0.105824	-1.56479	0.117631	0.197032
FTL_0748	1334.422	0.025443	0.093431	0.272322	0.785374	0.853298
FTL_0749	121.8514	0.169445	0.23122	0.732832	0.463661	0.586507
FTL_0750	1286.264	0.171762	0.096778	1.774811	0.075929	0.138146
FTL_0751	1070.438	0.366777	0.100518	3.648879	0.000263	0.000947
FTL_0752	674.8228	-0.13703	0.116662	-1.17455	0.240175	0.353147
FTL_0753	208.6569	-0.25281	0.191726	-1.3186	0.187303	0.291167
FTL_0754	1801.127	0.379421	0.087618	4.330399	1.49E-05	6.45E-05
FTL_0755	1765.65	0.806777	0.090768	8.888319	6.20E-19	8.10E-18
FTL_0756	2623.286	0.463538	0.072945	6.354586	2.09E-10	1.54E-09
FTL_0757	764.2083	0.031496	0.117036	0.269112	0.787843	0.855057
FTL_0758	339.5269	-0.28904	0.154986	-1.86497	0.062186	0.117109
FTL_0759	245.6838	-0.07222	0.180749	-0.39956	0.68948	0.779007
FTL_0760	2069.075	-0.02505	0.084288	-0.29719	0.766322	0.839387
FTL_0761	1254.846	0.058739	0.096723	0.607285	0.543662	0.660626
FTL_0762	858.0112	0.031684	0.110843	0.285847	0.774995	0.845679
FTL_0763	1809.549	0.190629	0.090199	2.113425	0.034564	0.071474
FTL_0764	1351.401	0.159401	0.090127	1.76862	0.076957	0.139418
FTL_0765	866.9877	-0.42009	0.111638	-3.76298	0.000168	0.000623
FTL_0766	1530.372	-0.52539	0.089397	-5.87697	4.18E-09	2.7E-08
FTL_0767	753.6784	-0.50805	0.113763	-4.46586	7.97E-06	3.56E-05

FTL_0768	10031.28	-0.13856	0.059629	-2.32373	0.02014	0.04518
FTL_0769	191.0636	0.208133	0.196067	1.061541	0.288444	0.407144
FTL_0770	83.28799	0.039249	0.264757	0.148244	0.88215	0.920146
FTL_0771	406.4506	-0.07773	0.143178	-0.5429	0.587201	0.696001
FTL_0772	32.72659	0.107486	0.322623	0.333164	0.739011	0.81886
FTL_0773	28.56807	0.435933	0.326943	1.333361	0.182413	0.284888
FTL_0774	14.57283	0.427854	0.329482	1.298564	0.194094	0.297808
FTL_0775	15.10423	0.3612	0.329929	1.09478	0.273613	0.389767
FTL_0776	64.31334	-0.34525	0.284465	-1.21369	0.224867	0.333566
FTL_0777	8.989687	-0.11518	0.312576	-0.3685	0.712503	0.798735
FTL_0778	52.14707	0.343771	0.297516	1.15547	0.247898	0.361507
FTL_0779	150.4615	0.095644	0.219893	0.434956	0.663595	0.754853
FTL_0780	94.44825	0.059823	0.250951	0.238385	0.811583	0.870946
FTL_0781	170.3969	0.629681	0.203764	3.090241	0.002	0.005981
FTL_0782	108.9786	0.534237	0.247958	2.154547	0.031197	0.065116
FTL_0783	876.031	0.202265	0.106467	1.899787	0.057461	0.109372
FTL_0784	3485.541	-0.10044	0.067311	-1.49216	0.135657	0.221684
FTL_0785	1153.053	-0.08039	0.10647	-0.75507	0.450208	0.573823
FTL_0786	2806.318	0.429501	0.089336	4.807699	1.53E-06	7.36E-06
FTL_0787	1297.011	0.457295	0.093867	4.871749	1.11E-06	5.45E-06
FTL_0788	1570.175	0.376373	0.086044	4.374177	1.22E-05	5.35E-05
FTL_0789	3493.265	-0.04697	0.073777	-0.6366	0.524385	0.642116
FTL_0790	1590.16	-0.30566	0.084824	-3.60342	0.000314	0.001115
FTL_0791	175.7749	-0.15905	0.200887	-0.79176	0.4285	0.55246
FTL_0792	1122.878	0.108402	0.099992	1.084115	0.278314	0.395623
FTL_0793	330.0984	-0.05328	0.156493	-0.34049	0.733488	0.814087
FTL_0794	68.11203	0.344385	0.281541	1.223213	0.221249	0.328685
FTL_0795	5551.094	-0.2829	0.063987	-4.42112	9.82E-06	4.36E-05
FTL_0796	2897.991	0.165842	0.075477	2.197247	0.028003	0.060006
FTL_0797	914.0368	0.115058	0.110801	1.038426	0.299072	0.418909
FTL_0798	716.0881	0.289381	0.115413	2.507356	0.012164	0.029106
FTL_0799	1220.848	0.344622	0.107255	3.213104	0.001313	0.004111
FTL_0800	4830.004	0.140853	0.062581	2.250715	0.024404	0.053259
FTL_0801	5629.672	-0.08561	0.063621	-1.34569	0.178403	0.279058
FTL_0802	5822.11	-0.1581	0.060008	-2.63467	0.008422	0.02095
FTL_0803	3946.435	-0.33043	0.188275	-1.75505	0.079251	0.143121
FTL_0804	179.2165	-0.02792	0.206675	-0.13509	0.892543	0.927009
FTL_0805	20673.94	0.287835	0.055978	5.141893	2.72E-07	1.43E-06
FTL_0806	2985.392	-0.79298	0.077334	-10.254	1.14E-24	2.13E-23
FTL_0807	1461.425	-0.5334	0.096872	-5.50627	3.67E-08	2.10E-07
FTL_0808	1535.191	-0.23538	0.087163	-2.70045	0.006925	0.017663
FTL_0809	2017.33	-0.3105	0.084212	-3.68717	0.000227	0.000824
FTL_0810	69.20605	-0.06639	0.275525	-0.24096	0.809583	0.869728

FTL_0811	42.06818	-0.18504	0.309529	-0.5978	0.549971	0.665127
FTL_0812	34.71536	-0.42445	0.318948	-1.33078	0.183261	0.28599
FTL_0813	150.3868	-0.27876	0.213067	-1.30831	0.190769	0.294696
FTL_0814	76.5165	-1.0041	0.272939	-3.67886	0.000234	0.000847
FTL_0815	49.90529	-1.20526	0.304017	-3.96446	7.36E-05	0.000288
FTL_0816	39.98417	-1.31537	0.315988	-4.16271	3.14E-05	0.000132
FTL_0817	74.67768	0.189564	0.270288	0.701341	0.48309	0.605368
FTL_0818	37.90066	0.258337	0.320063	0.807143	0.419584	0.545161
FTL_0819	47.91236	0.235177	0.302579	0.777243	0.437015	0.561637
FTL_0820	146.1838	0.085191	0.215735	0.394887	0.692926	0.781145
FTL_0821	909.2802	-0.33095	0.105848	-3.12661	0.001768	0.005345
FTL_0822	1276.363	0.094862	0.108563	0.873796	0.382229	0.507787
FTL_0823	1341.186	0.050922	0.102657	0.496036	0.619869	0.719779
FTL_0824	1173.025	-0.16078	0.103636	-1.55144	0.120797	0.201162
FTL_0825	977.5018	-0.32654	0.113205	-2.8845	0.00392	0.010914
FTL_0826	314.0039	-0.16701	0.159348	-1.04808	0.294601	0.414089
FTL_0827	3323.06	-0.07042	0.070664	-0.9966	0.318958	0.441465
FTL_0828	7745.946	0.031885	0.058853	0.541768	0.587978	0.696017
FTL_0829	1773.837	0.210711	0.09779	2.154725	0.031183	0.065116
FTL_0830	2207.529	-0.03739	0.077331	-0.48353	0.628721	0.727535
FTL_0831	4896.794	-0.97492	0.065812	-14.8139	1.19E-49	6.30E-48
FTL_0832	3594.276	-1.34916	0.072783	-18.5367	1.05E-76	9.13E-75
FTL_0833	1473.455	-0.65594	0.089234	-7.35078	1.97E-13	1.83E-12
FTL_0834	2907.514	-0.36636	0.081369	-4.50247	6.72E-06	3.02E-05
FTL_0835	168.9079	-0.02838	0.202911	-0.13987	0.888765	0.924169
FTL_0836	359.4885	-0.06828	0.152311	-0.44828	0.65395	0.74769
FTL_0837	2636.203	-0.40063	0.074798	-5.35622	8.5E-08	4.74E-07
FTL_0838	1813.641	-0.52378	0.081822	-6.40141	1.54E-10	1.16E-09
FTL_0839	1402.264	-0.37266	0.08992	-4.14434	3.41E-05	0.000142
FTL_0840	444.2525	-0.0763	0.154502	-0.49386	0.621405	0.721095
FTL_0841	537.8521	0.049494	0.134997	0.36663	0.713895	0.799849
FTL_0842	132.8573	-0.12263	0.223668	-0.54826	0.583516	0.694004
FTL_0843	2848.792	0.086754	0.074919	1.15797	0.246876	0.360626
FTL_0844	551.5458	0.346808	0.129018	2.688061	0.007187	0.018285
FTL_0845	113.2746	-0.0043	0.235524	-0.01826	0.985429	0.988873
FTL_0846	560.4699	-0.27524	0.153083	-1.79796	0.072183	0.132864
FTL_0847	1855.282	-0.27993	0.083568	-3.34968	0.000809	0.002644
FTL_0848	8463.92	-0.09486	0.055826	-1.69917	0.089287	0.156876
FTL_0849	2304.026	-0.16376	0.075493	-2.16915	0.030072	0.063226
FTL_0850	3562.002	-0.1532	0.077538	-1.9758	0.048177	0.094382
FTL_0851	5927.901	-0.36221	0.06238	-5.80645	6.38E-09	4.02E-08
FTL_0852	3388.285	0.17007	0.071867	2.366459	0.017959	0.041114
FTL_0853	832.4477	0.451127	0.112915	3.995291	6.46E-05	0.000255

FTL_0854	544.0163	0.4365	0.132728	3.288675	0.001007	0.003217
FTL_0855	1360.105	0.153336	0.09472	1.618842	0.105481	0.18044
FTL_0856	1505.071	-0.13115	0.086344	-1.51893	0.128779	0.212169
FTL_0857	291.1834	-0.59446	0.177578	-3.34762	0.000815	0.00266
FTL_0858	2148	-0.0215	0.082102	-0.26184	0.793447	0.85882
FTL_0859	632.0408	-0.18164	0.126826	-1.43217	0.152096	0.243402
FTL_0860	287.4932	0.425367	0.169877	2.503969	0.012281	0.029317
FTL_0861	130.2247	0.115356	0.225326	0.511953	0.608684	0.712139
FTL_0862	816.2885	0.445608	0.112396	3.96464	7.35E-05	0.000288
FTL_0863	4256.609	0.364195	0.070355	5.176502	2.26E-07	1.21E-06
FTL_0864	1933.696	-0.02527	0.095287	-0.26521	0.790851	0.856933
FTL_0865	4105	-0.12198	0.068976	-1.76841	0.076992	0.139418
FTL_0866	554.4495	0.127361	0.139387	0.913718	0.360865	0.484528
FTL_0867	4001.106	0.456971	0.074884	6.102386	1E-09	7.07E-09
FTL_0868	138.5572	0.115099	0.219179	0.525134	0.59949	0.704663
FTL_0869	20.66291	0.646526	0.333249	1.940069	0.052371	0.101413
FTL_0870	8.530068	0.180978	0.305901	0.591622	0.554104	0.668115
FTL_0871	48.46239	0.144988	0.300564	0.482386	0.629532	0.728055
FTL_0872	54.71711	-0.08965	0.296882	-0.30199	0.762662	0.836763
FTL_0873	91.37805	0.100316	0.25389	0.395114	0.692758	0.781145
FTL_0874	72.50361	-0.22256	0.278813	-0.79824	0.42473	0.549008
FTL_0875	4755.823	0.59982	0.062917	9.533522	1.52E-21	2.33E-20
FTL_0876	2871.399	0.226131	0.070623	3.201972	0.001365	0.004247
FTL_0877	7502.845	-0.40252	0.063	-6.38914	1.67E-10	1.26E-09
FTL_0878	1047.404	-0.4155	0.099825	-4.16235	3.15E-05	0.000132
FTL_0879	885.5753	-1.03196	0.108747	-9.48957	2.32E-21	3.48E-20
FTL_0880	245.9253	-0.47185	0.190554	-2.47622	0.013278	0.031289
FTL_0881	123.2884	-0.77939	0.239132	-3.25925	0.001117	0.003536
FTL_0882	1581.021	-0.08244	0.087025	-0.94728	0.343494	0.466501
FTL_0883	1130.683	-0.13103	0.10023	-1.30733	0.1911	0.294696
FTL_0884	1409.93	0.201802	0.099258	2.033101	0.042042	0.084001
FTL_0885	6972.236	0.329919	0.063748	5.175327	2.28E-07	1.21E-06
FTL_0886	10145.79	0.389457	0.056556	6.886204	5.73E-12	4.84E-11
FTL_0887	942.211	0.31818	0.108257	2.939132	0.003291	0.009318
FTL_0888	495.0679	0.031125	0.13513	0.23033	0.817835	0.874853
FTL_0889	123.2975	-0.25389	0.23672	-1.07251	0.283489	0.400995
FTL_0890	407.516	0.137835	0.146266	0.942356	0.346011	0.468396
FTL_0891	11133.65	-0.53569	0.061036	-8.77657	1.69E-18	2.13E-17
FTL_0892	3906.668	-0.81604	0.071302	-11.4449	2.49E-30	5.70E-29
FTL_0893	7974.106	-0.99009	0.061053	-16.2167	3.84E-59	2.66E-57
FTL_0894	17338.07	-0.80114	0.05264	-15.2193	2.63E-52	1.47E-50
FTL_0895	3867.499	-0.58619	0.071317	-8.21953	2.04E-16	2.29E-15
FTL_0896	3000.463	-0.464	0.073344	-6.32626	2.51E-10	1.84E-09

FTL_0897	969.8741	-0.23871	0.104754	-2.2788	0.022679	0.050149
FTL_0898	5345.551	0.102165	0.068796	1.485038	0.137534	0.22384
FTL_0899	12795.81	-0.05871	0.059264	-0.99061	0.321877	0.443672
FTL_0900	1423.246	-0.17742	0.094279	-1.88185	0.059856	0.113287
FTL_0901	355.2285	-0.33966	0.155567	-2.18334	0.029011	0.06151
FTL_0902	211.105	-0.65031	0.191097	-3.40302	0.000666	0.002218
FTL_0903	7027.405	-0.01839	0.069857	-0.26324	0.792365	0.858111
FTL_0904	4335.227	-0.10621	0.070098	-1.5151	0.129748	0.21324
FTL_0905	2999.877	0.084129	0.082397	1.021016	0.307247	0.428865
FTL_0906	3850.912	-0.16161	0.071754	-2.25229	0.024304	0.053099
FTL_0907	885.5315	-0.39115	0.107353	-3.64362	0.000269	0.000965
FTL_0908	2659.337	0.145638	0.072781	2.001061	0.045386	0.089835
FTL_0909	132.4265	0.487434	0.224778	2.168511	0.03012	0.063261
FTL_0910	55.81315	0.789805	0.29223	2.702685	0.006878	0.017567
FTL_0911	222.7605	0.528708	0.186226	2.839064	0.004525	0.01231
FTL_0912	736.6436	0.876654	0.114215	7.675438	1.65E-14	1.66E-13
FTL_0913	6861.56	-0.17157	0.060506	-2.83554	0.004575	0.012426
FTL_0914	1759.708	-0.04268	0.085867	-0.49704	0.61916	0.719779
FTL_0915	1440.946	0.275302	0.10143	2.714198	0.006644	0.017076
FTL_0916	7237.085	0.117772	0.065697	1.79266	0.073027	0.134295
FTL_0917	7231.791	-0.03764	0.057348	-0.65632	0.511617	0.632441
FTL_0918	6301.304	0.021683	0.067296	0.322198	0.747303	0.825771
FTL_0919	1029.084	-0.201	0.11248	-1.78695	0.073945	0.135365
FTL_0920	976.5646	-0.39245	0.106973	-3.66872	0.000244	0.00088
FTL_0921	992.4691	-0.35649	0.103397	-3.44777	0.000565	0.001909
FTL_0922	2623.274	-0.33185	0.082871	-4.00446	6.22E-05	0.000246
FTL_0923	3678.777	-0.24002	0.069332	-3.46185	0.000536	0.001825
FTL_0924	137.8471	-1.45078	0.225377	-6.43711	1.22E-10	9.27E-10
FTL_0925	1442.041	-0.16681	0.095856	-1.74024	0.081818	0.146833
FTL_0926	2704.945	-0.44735	0.076233	-5.86828	4.40E-09	2.83E-08
FTL_0927	1887.742	0.087169	0.081449	1.070225	0.284518	0.402167
FTL_0928	3536.587	0.296335	0.074947	3.953945	7.69E-05	0.0003
FTL_0929	3773.567	0.217329	0.074374	2.922123	0.003477	0.009814
FTL_0930	1984.163	0.061703	0.078764	0.783394	0.433396	0.558056
FTL_0931	2196.845	-0.08592	0.076474	-1.12357	0.261194	0.376614
FTL_0932	907.4378	0.005339	0.104262	0.051203	0.959164	0.970755
FTL_0933	1465.996	-0.085	0.088811	-0.95705	0.338544	0.460713
FTL_0934	166.8983	0.002976	0.203997	0.014587	0.988361	0.991321
FTL_0935	423.9565	0.019338	0.143603	0.13466	0.892881	0.927009
FTL_0936	162.3816	0.139922	0.212547	0.658309	0.51034	0.631251
FTL_0937	737.7433	-0.74909	0.118685	-6.31158	2.76E-10	2E-09
FTL_0938	1818.249	-0.61152	0.089341	-6.84473	7.66E-12	6.44E-11
FTL_0939	1079.386	-0.77796	0.105062	-7.40483	1.31E-13	1.25E-12

FTL_0940	129.554	-0.73734	0.230682	-3.19636	0.001392	0.004324
FTL_0941	978.1477	-0.6786	0.105893	-6.4084	1.47E-10	1.12E-09
FTL_0942	1639.362	-0.45203	0.094385	-4.78921	1.67E-06	8.03E-06
FTL_0943	528.171	-0.23262	0.131491	-1.76909	0.076879	0.139418
FTL_0944	208.1108	-0.22079	0.193094	-1.14344	0.252855	0.367225
FTL_0945	249.1013	-0.22265	0.174768	-1.27398	0.202671	0.306985
FTL_0946	189.4636	0.211234	0.218411	0.967139	0.333474	0.455047
FTL_0947	128.6264	0.014246	0.225209	0.063257	0.949562	0.963462
FTL_0948	630.0206	0.07698	0.124032	0.620649	0.534831	0.652312
FTL_0949	9971.471	-0.25273	0.063525	-3.97842	6.94E-05	0.000273
FTL_0950	3469.494	-0.27441	0.090104	-3.04553	0.002323	0.006856
FTL_0951	2976.217	-0.31052	0.076516	-4.05824	4.94E-05	0.0002
FTL_0952	91.36846	-0.32179	0.27441	-1.17267	0.240927	0.353993
FTL_0953	1127.095	-1.65431	0.099104	-16.6926	1.48E-62	1.06E-60
FTL_0954	333.6306	-0.19441	0.155153	-1.25305	0.210189	0.314814
FTL_0955	2717.655	-0.00393	0.079729	-0.04932	0.960662	0.971621
FTL_0956	522.4478	-0.01878	0.129825	-0.14465	0.884987	0.92167
FTL_0957	576.4061	0.088343	0.126748	0.696996	0.485805	0.607256
FTL_0958	808.9128	-0.09076	0.108524	-0.83633	0.402967	0.528697
FTL_0959	1414.397	0.4977	0.093071	5.347549	8.92E-08	4.96E-07
FTL_0960	6031.897	0.340882	0.065973	5.167028	2.38E-07	1.26E-06
FTL_0961	193.3423	-0.02672	0.195211	-0.13688	0.891126	0.926145
FTL_0962	142.5666	-0.51397	0.221467	-2.32074	0.020301	0.04544
FTL_0963	801.4085	-0.60005	0.111559	-5.37878	7.5E-08	4.20E-07
FTL_0964	4547.205	-1.63318	0.069011	-23.6654	8.20E-124	1.27E-121
FTL_0965	1566.653	-1.17096	0.088229	-13.2719	3.37E-40	1.13E-38
FTL_0966	796.544	-0.31641	0.114811	-2.75591	0.005853	0.015338
FTL_0967	795.4294	-0.40814	0.119435	-3.41724	0.000633	0.002119
FTL_0968	4821.625	-0.03338	0.064143	-0.52044	0.602755	0.70724
FTL_0969	853.7936	0.244627	0.110616	2.211496	0.027001	0.058295
FTL_0970	173.8808	0.107591	0.203946	0.527549	0.597813	0.703103
FTL_0971	362.2744	0.091155	0.155222	0.587255	0.557032	0.67044
FTL_0972	243.3133	0.254017	0.182797	1.389613	0.164646	0.260141
FTL_0973	253.8184	0.094786	0.184003	0.515132	0.606461	0.710365
FTL_0974	902.7211	-0.25824	0.104275	-2.47656	0.013265	0.031289
FTL_0975	1306.775	0.004647	0.097454	0.047687	0.961965	0.971621
FTL_0976	796.0423	0.322499	0.118069	2.731452	0.006306	0.01632
FTL_0977	45.64834	0.902159	0.306848	2.940081	0.003281	0.009302
FTL_0978	46.0608	0.304801	0.30678	0.993549	0.320443	0.442361
FTL_0979	79.08763	-0.19024	0.268973	-0.70729	0.479384	0.601475
FTL_0980	235.8739	0.447738	0.187311	2.390341	0.016833	0.038756
FTL_0981	520.8682	0.090083	0.129498	0.695631	0.48666	0.607946

FTL_0982	558.9671	0.104498	0.134831	0.775031	0.438322	0.562901
FTL_0983	1333.931	-0.09972	0.096755	-1.03066	0.302702	0.423403
FTL_0984	9248.356	-0.2497	0.065774	-3.79638	0.000147	0.000549
FTL_0985	2565.761	-0.20606	0.075347	-2.73486	0.006241	0.016186
FTL_0986	10902.39	-0.09548	0.057182	-1.66982	0.094954	0.165245
FTL_0987	8774.694	-0.34346	0.056588	-6.06954	1.28E-09	8.60E-09
FTL_0988	497.7322	0.00951	0.13777	0.069025	0.94497	0.959772
FTL_0989	1516.232	-0.55477	0.097614	-5.68328	1.32E-08	8.02E-08
FTL_0990	780.4723	-0.46211	0.118678	-3.89377	9.87E-05	0.000376
FTL_0991	25.47221	0.413407	0.331129	1.248475	0.211857	0.316369
FTL_0992	1013.342	0.203536	0.101177	2.011687	0.044253	0.087807
FTL_0993	474.5269	0.097675	0.136854	0.713718	0.475401	0.597869
FTL_0994	3757.407	-0.04172	0.06636	-0.62868	0.529559	0.64706
FTL_0995	2118.209	-0.18814	0.077639	-2.42332	0.015379	0.035737
FTL_0996	831.7733	-0.12504	0.134056	-0.93275	0.35095	0.472477
FTL_0997	201.9987	0.543982	0.191114	2.84638	0.004422	0.012093
FTL_0998	193.4452	0.57284	0.193982	2.953057	0.003146	0.008958
FTL_0999	501.7381	-0.1365	0.145432	-0.9386	0.347937	0.469995
FTL_1000	549.6269	0.182734	0.13072	1.397896	0.162144	0.25784
FTL_1001	955.6378	0.100031	0.117771	0.849367	0.395677	0.5222
FTL_1002	148.897	0.100663	0.217433	0.46296	0.643393	0.739563
FTL_1003	1017.194	0.077692	0.103661	0.749477	0.45357	0.576645
FTL_1004	1170.857	0.257192	0.096409	2.667712	0.007637	0.019357
FTL_1005	1711.68	0.143155	0.08305	1.723709	0.08476	0.150591
FTL_1006	854.6656	-0.26747	0.113016	-2.36666	0.017949	0.041114
FTL_1007	575.8232	-0.3889	0.125174	-3.10689	0.001891	0.00568
FTL_1008	157.5268	-0.52753	0.216225	-2.43974	0.014698	0.034272
FTL_1009	153.9669	0.104205	0.211349	0.493047	0.62198	0.721095
FTL_1010	72.4023	-0.35532	0.281898	-1.26047	0.2075	0.312511
FTL_1011	23.22216	-0.1652	0.332213	-0.49726	0.619003	0.719779
FTL_1012	452.1516	-0.13035	0.138025	-0.94437	0.344978	0.467886
FTL_1013	1098.539	-0.25293	0.104829	-2.41276	0.015832	0.036705
FTL_1014	1141.092	-0.32245	0.097241	-3.31598	0.000913	0.002946
FTL_1015	5497.686	0.466764	0.08104	5.75968	8.43E-09	5.21E-08
FTL_1016	6956.654	0.480057	0.061737	7.775814	7.50E-15	7.69E-14
FTL_1017	3463.985	0.400202	0.07279	5.498046	3.84E-08	2.19E-07
FTL_1018	2421.417	0.043906	0.075846	0.578886	0.562666	0.675603
FTL_1019	30.18924	-0.19715	0.324762	-0.60707	0.543803	0.660626
FTL_1020	977.8309	0.206481	0.102676	2.010999	0.044326	0.087864
FTL_1021	1144.53	-0.12978	0.100423	-1.29237	0.196228	0.299451
FTL_1022	2637.746	-0.03776	0.080803	-0.46727	0.640307	0.737546
FTL_1023	258.5391	0.226967	0.180434	1.257897	0.208429	0.313111
FTL_1024	15933.8	0.276285	0.067112	4.116792	3.84E-05	0.000158

FTL_1025	5874.387	0.240089	0.06284	3.820667	0.000133	0.000502
FTL_1026	9557.046	0.223271	0.06428	3.473426	0.000514	0.001757
FTL_1027	6628.143	0.329087	0.060656	5.425436	5.78E-08	3.29E-07
FTL_1028	3852.482	-0.12443	0.069797	-1.78276	0.074626	0.136486
FTL_1029	3002.198	-0.18425	0.07208	-2.5562	0.010582	0.025845
FTL_1030	3020.674	-0.44837	0.070856	-6.32788	2.49E-10	1.83E-09
FTL_1031	637.409	-0.47785	0.119823	-3.98795	6.66E-05	0.000263
FTL_1032	294.876	-0.669	0.185245	-3.61142	0.000305	0.001083
FTL_1033	147.0961	0.265191	0.214467	1.236512	0.216268	0.321999
FTL_1034	1506.15	-0.10052	0.087507	-1.14867	0.250691	0.364344
FTL_1035	528.6735	-0.03528	0.131889	-0.26748	0.789096	0.855569
FTL_1036	218.2139	0.352946	0.193216	1.826689	0.067747	0.125735
FTL_1037	1113.368	-0.10939	0.101065	-1.08233	0.279104	0.396466
FTL_1038	811.7356	-0.17954	0.108545	-1.65405	0.098117	0.169575
FTL_1039	145.5194	-0.06793	0.22212	-0.30581	0.759748	0.835047
FTL_1040	782.0294	0.023382	0.114642	0.203955	0.838389	0.889475
FTL_1041	2381.924	-0.15506	0.076244	-2.03372	0.04198	0.08396
FTL_1042	4574.691	-0.09988	0.071927	-1.38861	0.164952	0.260246
FTL_1043	2663.531	-0.1706	0.075944	-2.24637	0.02468	0.053805
FTL_1044	1029.477	-0.12511	0.099306	-1.25986	0.207719	0.312511
FTL_1045	1727.754	-0.21158	0.083516	-2.5334	0.011296	0.027356
FTL_1046	7236.271	0.044331	0.058096	0.763055	0.445431	0.569177
FTL_1047	746.4044	0.757037	0.113918	6.645436	3.02E-11	2.43E-10
FTL_1048	2851.053	0.165652	0.074444	2.225202	0.026068	0.056522
FTL_1049	5263.349	-0.05308	0.064439	-0.82367	0.410126	0.535642
FTL_1050	10433.66	-0.07816	0.056212	-1.39048	0.164382	0.259959
FTL_1051	1882.224	-0.22903	0.090432	-2.53268	0.011319	0.027379
FTL_1052	245.4837	0.048536	0.180127	0.269457	0.787578	0.855057
FTL_1053	407.9142	1.6516	0.154023	10.7231	7.93E-27	1.68E-25
FTL_1054	294.6108	0.992099	0.168801	5.877344	4.17E-09	2.7E-08
FTL_1055	346.1812	0.209245	0.160086	1.307079	0.191186	0.294696
FTL_1056	79.0248	-0.15218	0.265246	-0.57373	0.56615	0.678569
FTL_1057	279.0138	-0.65675	0.178332	-3.68274	0.000231	0.000836
FTL_1058	1855.11	0.130062	0.086428	1.504866	0.132359	0.217353
FTL_1059	1545.043	0.254465	0.095715	2.658583	0.007847	0.019765
FTL_1060	10988.36	0.331481	0.059006	5.617778	1.93E-08	1.14E-07
FTL_1061	3649.408	0.571392	0.074689	7.650284	2.01E-14	1.99E-13
FTL_1062	1202.239	0.160082	0.100944	1.58585	0.112773	0.190163
FTL_1063	1064.715	0.027983	0.098984	0.282697	0.777409	0.847768
FTL_1064	2395.963	-0.04197	0.075546	-0.5555	0.578552	0.690963
FTL_1065	1495.23	-0.03715	0.087028	-0.4269	0.669455	0.760182
FTL_1066	770.3761	-0.01822	0.11697	-0.15574	0.876234	0.91806
FTL_1067	2748.709	-0.18941	0.071476	-2.65002	0.008049	0.02017

FTL_1068	545.6289	-0.3665	0.128411	-2.85411	0.004316	0.011851
FTL_1069	373.4273	-0.40878	0.166236	-2.45904	0.013931	0.03275
FTL_1070	10.38182	0.067403	0.316033	0.213277	0.831111	0.882945
FTL_1071	5169.128	-0.22106	0.073113	-3.02346	0.002499	0.007322
FTL_1072	7853.333	-0.1167	0.060585	-1.92625	0.054073	0.104107
FTL_1073	1532.534	-0.33594	0.093366	-3.59808	0.000321	0.001134
FTL_1074	2337.645	-0.12745	0.078087	-1.63216	0.102645	0.176189
FTL_1075	4299.795	-0.08861	0.064458	-1.37472	0.169217	0.26614
FTL_1076	902.6287	-0.15319	0.104263	-1.46924	0.141768	0.230172
FTL_1077	2490.75	-0.00162	0.075115	-0.02151	0.98284	0.987261
FTL_1078	484.0042	-0.37827	0.138201	-2.73712	0.006198	0.016095
FTL_1079	57.00912	-0.29939	0.288274	-1.03855	0.299014	0.418909
FTL_1080	122.5378	-0.25537	0.228668	-1.11676	0.264097	0.379177
FTL_1081	28.94406	-0.29011	0.327701	-0.88529	0.376	0.501167
FTL_1082	16.03418	-0.25664	0.332418	-0.77205	0.440084	0.564139
FTL_1083	54.72426	-0.84374	0.295312	-2.85711	0.004275	0.011788
FTL_1084	42.95275	0.087313	0.309387	0.282214	0.77778	0.847768
FTL_1085	53.01357	0.214953	0.294986	0.728689	0.466192	0.589337
FTL_1086	382.445	-0.4753	0.153378	-3.09891	0.001942	0.005818
FTL_1087	235.8588	0.017138	0.19134	0.089566	0.928632	0.949781
FTL_1088	1718.831	-0.15375	0.085117	-1.80638	0.070859	0.131148
FTL_1089	1732.12	-0.31242	0.087406	-3.57441	0.000351	0.001221
FTL_1090	779.7088	0.223184	0.112191	1.989324	0.046665	0.091746
FTL_1091	78.34006	-0.46368	0.267448	-1.73373	0.082967	0.148366
FTL_1092	434.0663	0.028497	0.143115	0.19912	0.842169	0.892335
FTL_1093	2874.883	-0.07361	0.084303	-0.87313	0.382594	0.507936
FTL_1094	2112.364	-0.03087	0.086138	-0.35833	0.720094	0.805517
FTL_1095	324.7712	-0.44279	0.157012	-2.82013	0.0048	0.012969
FTL_1096	8177.588	-0.32653	0.061589	-5.30173	1.15E-07	6.21E-07
FTL_1097	2205.493	-0.4009	0.097312	-4.11978	3.79E-05	0.000156
FTL_1098	359.1388	0.037516	0.15066	0.249013	0.803351	0.86767
FTL_1099	1567.999	-0.14945	0.091179	-1.6391	0.101192	0.173842
FTL_1100	2356.01	-0.1574	0.078801	-1.99748	0.045773	0.090288
FTL_1101	945.8208	0.075991	0.115062	0.660431	0.508977	0.629953
FTL_1102	726.7898	0.209051	0.116708	1.791234	0.073256	0.134384
FTL_1103	235.0341	0.403215	0.195317	2.064413	0.038979	0.078979
FTL_1104	263.0191	0.449656	0.178402	2.520463	0.01172	0.028246
FTL_1105	2311.676	0.05689	0.088495	0.642856	0.520317	0.639656
FTL_1106	8262.054	0.250692	0.058946	4.252882	2.11E-05	9.07E-05
FTL_1107	2272.488	0.229072	0.07727	2.964555	0.003031	0.008667
FTL_1108	3327.233	0.103824	0.069699	1.489598	0.13633	0.222422
FTL_1109	3522.087	-0.20475	0.067154	-3.04893	0.002297	0.006798
FTL_1110	1316.738	-0.34855	0.090897	-3.83455	0.000126	0.000476

FTL_1111	258.3515	-0.56063	0.178592	-3.13919	0.001694	0.00516
FTL_1112	190.9149	0.106068	0.198228	0.535083	0.592592	0.699419
FTL_1113	187.0393	-0.08577	0.201124	-0.42643	0.669792	0.760182
FTL_1114	232.0399	0.204893	0.186666	1.097644	0.27236	0.388258
FTL_1115	1895.303	0.150533	0.080728	1.864688	0.062225	0.117109
FTL_1116	1693.741	0.05851	0.090211	0.64859	0.516604	0.63743
FTL_1117	1983.388	-0.11026	0.079253	-1.39122	0.164158	0.259809
FTL_1118	3276.209	-0.14512	0.069246	-2.09565	0.036113	0.074372
FTL_1119	1802.237	0.033587	0.097244	0.345388	0.729802	0.811789
FTL_1120	585.7686	-0.26964	0.13556	-1.98906	0.046695	0.091746
FTL_1121	319.2904	-0.37487	0.165766	-2.26143	0.023732	0.052137
FTL_1122	133.0763	-0.34784	0.233097	-1.49227	0.135628	0.221684
FTL_1123	95.56657	0.121019	0.252611	0.479071	0.631888	0.72994
FTL_1124	65.95556	0.202938	0.279666	0.725647	0.468055	0.590407
FTL_1125	196.1666	0.048013	0.197049	0.243661	0.807493	0.869372
FTL_1126	69.36535	0.759394	0.279739	2.714653	0.006635	0.017075
FTL_1127	823.191	0.295596	0.113176	2.611825	0.009006	0.022184
FTL_1128	174.1318	0.108636	0.201919	0.538018	0.590565	0.697436
FTL_1129	1699.058	-0.06739	0.094464	-0.71337	0.475618	0.597869
FTL_1130	1039.055	-0.12705	0.113288	-1.12146	0.262092	0.377638
FTL_1131	902.5834	-0.23498	0.105995	-2.21687	0.026632	0.05756
FTL_1132	3396.819	-0.21208	0.070019	-3.02889	0.002455	0.007202
FTL_1133	921.9024	-0.18261	0.110683	-1.64983	0.098977	0.170767
FTL_1134	4873.184	-0.31378	0.062043	-5.05742	4.25E-07	2.18E-06
FTL_1135	1447.58	0.289971	0.092417	3.137644	0.001703	0.005179
FTL_1136	6317.813	0.180609	0.065933	2.739258	0.006158	0.016053
FTL_1137	55418.25	0.08331	0.056845	1.465572	0.142765	0.231045
FTL_1138	21905.23	-0.15625	0.055768	-2.8018	0.005082	0.013638
FTL_1139	16491.29	0.220969	0.058211	3.796005	0.000147	0.000549
FTL_1140	21171.07	0.290325	0.057111	5.083535	3.70E-07	1.92E-06
FTL_1141	9227.424	-0.06268	0.061536	-1.01859	0.308399	0.429877
FTL_1142	22101.43	0.006993	0.053724	0.130161	0.896439	0.928348
FTL_1143	10952.26	0.079271	0.063775	1.242973	0.213878	0.319149
FTL_1144	5808.876	-0.07407	0.062338	-1.18824	0.234737	0.34591
FTL_1145	9777.555	-0.3248	0.057565	-5.64224	1.68E-08	9.95E-08
FTL_1146	5485.351	-0.52177	0.081689	-6.38725	1.69E-10	1.27E-09
FTL_1147	6597.474	-0.5565	0.060996	-9.12346	7.28E-20	1.02E-18
FTL_1148	8403.488	-0.50804	0.067446	-7.53254	4.98E-14	4.83E-13
FTL_1149	4195.826	-0.63392	0.064547	-9.82106	9.14E-23	1.51E-21
FTL_1150	310.0237	-0.69562	0.167685	-4.1484	3.35E-05	0.00014
FTL_1151	149.8522	-0.08557	0.213142	-0.40146	0.688082	0.777865
FTL_1152	19.65079	0.047893	0.333326	0.143681	0.885752	0.921989
FTL_1153	955.6471	0.203138	0.104519	1.943558	0.051949	0.100789

FTL_1154	98.53162	0.609179	0.248018	2.456186	0.014042	0.032973
FTL_1155	130.7403	0.492862	0.225724	2.183471	0.029001	0.06151
FTL_1156	21.04544	-0.08154	0.333075	-0.2448	0.806613	0.869326
FTL_1157	1428.076	-3.34829	0.104003	-32.1942	2.13E-227	7.13E-225
FTL_1158	8955.224	-2.33584	0.060534	-38.5875	0	0
FTL_1159	6273.312	-1.75204	0.066669	-26.2798	3.27E-152	6.57E-150
FTL_1160	4240.683	-1.66231	0.065918	-25.2178	2.55E-140	4.28E-138
FTL_1161	808.1356	-1.55155	0.115256	-13.4617	2.63E-41	9.11E-40
FTL_1162	3726.811	-1.64911	0.082736	-19.9322	2.14E-88	2.26E-86
FTL_1163	399.9976	-1.50344	0.163421	-9.19983	3.59E-20	5.04E-19
FTL_1164	1079.904	-1.73816	0.111917	-15.5308	2.15E-54	1.23E-52
FTL_1165	906.5453	-1.67294	0.11418	-14.6518	1.31E-48	6.27E-47
FTL_1166	1244.548	-1.87157	0.099557	-18.799	7.69E-79	7.36E-77
FTL_1167	1049.44	-2.4451	0.11728	-20.8485	1.57E-96	1.86E-94
FTL_1168	475.5017	-1.45576	0.163815	-8.88662	6.30E-19	8.12E-18
FTL_1169	138.7758	-1.12546	0.233178	-4.82663	1.39E-06	6.76E-06
FTL_1170	215.0953	-1.58121	0.20273	-7.79956	6.21E-15	6.40E-14
FTL_1171	1666.158	-2.01225	0.090431	-22.2518	1.08E-109	1.45E-107
FTL_1172	1997.492	-2.62209	0.08584	-30.5463	6.33E-205	1.59E-202
FTL_1173	2494.127	-0.0753	0.075368	-0.99909	0.317752	0.440773
FTL_1174	2230.954	-0.88408	0.084165	-10.5041	8.27E-26	1.70E-24
FTL_1175	212.6099	-0.23964	0.190725	-1.25648	0.208943	0.313648
FTL_1176	212.4223	-0.03583	0.190672	-0.18793	0.850929	0.89972
FTL_1177	3629.321	-0.23679	0.072535	-3.26451	0.001097	0.003476
FTL_1178	407.339	0.095361	0.154737	0.616279	0.537711	0.654914
FTL_1179	4952.385	0.214572	0.064055	3.34981	0.000809	0.002644
FTL_1180	3352.691	0.120799	0.070765	1.707044	0.087814	0.15483
FTL_1181	2989.101	0.03335	0.074691	0.446513	0.655226	0.74816
FTL_1182	1434.693	-0.20099	0.087856	-2.2877	0.022155	0.049151
FTL_1183	4139.956	0.191717	0.074406	2.576624	0.009977	0.024456
FTL_1184	4645.937	0.095935	0.065735	1.459425	0.144448	0.233539
FTL_1185	9152.069	0.18783	0.058565	3.207229	0.00134	0.004183
FTL_1186	15882.17	0.170741	0.0598	2.855205	0.004301	0.011842
FTL_1187	17254.84	0.216222	0.057722	3.745955	0.00018	0.000664
FTL_1188	2092.147	-0.05983	0.077662	-0.77044	0.441041	0.565005
FTL_1189	1711.295	0.141914	0.088832	1.597555	0.110142	0.186508
FTL_1190	2841.496	-0.88451	0.073404	-12.0499	1.94E-33	5.20E-32
FTL_1191	71996.69	-0.61466	0.050042	-12.2829	1.12E-34	3.17E-33
FTL_1192	20176.55	-0.95094	0.051033	-18.6336	1.71E-77	1.57E-75

FTL_1193	1913.29	-0.84921	0.091657	-9.26511	1.95E-20	2.80E-19
FTL_1194	92.1046	1.144561	0.260256	4.397818	1.09E-05	4.83E-05
FTL_1195	997.5428	0.259304	0.104472	2.48204	0.013063	0.030891
FTL_1196	199.5931	1.096877	0.194283	5.645758	1.64E-08	9.81E-08
FTL_1197	4254.909	0.091808	0.0642	1.430026	0.152709	0.244189
FTL_1198	8168.381	0.319894	0.058003	5.515121	3.49E-08	2.01E-07
FTL_1199	1348.463	0.085446	0.109304	0.781731	0.434373	0.558822
FTL_1200	332.8805	0.72535	0.160494	4.519472	6.20E-06	2.80E-05
FTL_1201	465.8783	0.909942	0.137352	6.624889	3.48E-11	2.78E-10
FTL_1202	650.7585	0.480263	0.12521	3.835676	0.000125	0.000475
FTL_1203	1473.529	0.013751	0.088815	0.154833	0.876953	0.91806
FTL_1204	860.3775	-0.25748	0.107678	-2.39118	0.016794	0.038712
FTL_1205	69.8687	0.010794	0.275182	0.039224	0.968712	0.97508
FTL_1206	220.2832	0.085772	0.185501	0.462379	0.643809	0.739563
FTL_1207	2341.283	0.43186	0.085115	5.073863	3.90E-07	2.01E-06
FTL_1208	1664.38	0.218293	0.085546	2.551752	0.010718	0.026114
FTL_1209	1001.008	0.195495	0.10143	1.927391	0.053931	0.103932
FTL_1210	319.71	-0.21584	0.180963	-1.19275	0.232969	0.343557
FTL_1211	1406.768	-0.27365	0.098272	-2.78464	0.005359	0.014215
FTL_1212	12897.6	-0.08765	0.055148	-1.58944	0.111961	0.189264
FTL_1213	1089.508	-1.38562	0.1018	-13.6113	3.43E-42	1.28E-40
FTL_1214	14.96055	-0.41357	0.330955	-1.24962	0.211439	0.315979
FTL_1215	1158.639	-0.17335	0.102423	-1.69247	0.090556	0.158691
FTL_1216	3110.591	-0.19772	0.074168	-2.66584	0.00768	0.019417
FTL_1217	2203.888	-1.5945	0.246841	-6.45962	1.05E-10	8.02E-10
FTL_1218	1890.412	-1.47332	0.26334	-5.59474	2.21E-08	1.29E-07
FTL_1219	5994.399	-3.64219	0.067111	-54.271	0	0
FTL_1220	326.2216	-0.28942	0.16691	-1.734	0.082918	0.148366
FTL_1221	192.5633	0.70795	0.197892	3.577463	0.000347	0.001209
FTL_1222	384.7187	-0.15863	0.146717	-1.08122	0.2796	0.396609
FTL_1223	1215.887	-0.85104	0.094328	-9.02219	1.84E-19	2.50E-18
FTL_1224	2445.143	-0.93711	0.079219	-11.8295	2.75E-32	6.91E-31
FTL_1225	2240.005	-1.2986	0.081747	-15.8856	7.97E-57	5.34E-55
FTL_1226	339.6908	-0.55836	0.155515	-3.59038	0.00033	0.001164
FTL_1227	1050.78	-0.71508	0.099169	-7.21068	5.57E-13	5.00E-12
FTL_1228	2193.567	0.00338	0.081561	0.041439	0.966946	0.974851
FTL_1229	1568.255	0.136567	0.08691	1.571357	0.1161	0.194792
FTL_1230	6467.538	0.307977	0.059795	5.150569	2.60E-07	1.37E-06
FTL_1231	1047.669	0.490682	0.117672	4.169929	3.05E-05	0.000128
FTL_1232	3948.89	0.089259	0.069086	1.292002	0.196356	0.299451
FTL_1233	2914.904	-0.24596	0.072407	-3.39685	0.000682	0.002257
FTL_1234	239.6791	-0.52814	0.192898	-2.73792	0.006183	0.016095
FTL_1235	2221.511	0.258676	0.077924	3.319615	0.000901	0.002922

FTL_1236	2155.051	-0.10523	0.077524	-1.35744	0.17464	0.273598
FTL_1237	1699.865	0.088842	0.087923	1.010449	0.31228	0.434684
FTL_1238	148.9893	-0.20204	0.213388	-0.94683	0.343725	0.466501
FTL_1239	12330.83	0.004479	0.060388	0.074165	0.940879	0.958038
FTL_1240	13060.91	-7.58E-05	0.060603	-0.00125	0.999003	0.999003
FTL_1241	1517.55	0.089253	0.086474	1.032137	0.302008	0.422727
FTL_1242	398.9667	-0.59559	0.148846	-4.00141	6.30E-05	0.000249
FTL_1243	1088.048	-0.9119	0.108382	-8.41371	3.97E-17	4.62E-16
FTL_1244	993.4411	0.17339	0.103837	1.669834	0.094952	0.165245
FTL_1245	733.5377	-0.12733	0.113639	-1.1205	0.262499	0.377954
FTL_1246	553.115	-0.08944	0.12765	-0.70066	0.483514	0.605522
FTL_1247	1060.788	-0.29804	0.098725	-3.01892	0.002537	0.007411
FTL_1248	2811.202	-0.14811	0.071573	-2.06934	0.038514	0.078432
FTL_1249	575.0369	-0.34647	0.125825	-2.75355	0.005895	0.015429
FTL_1250	144.0831	0.173412	0.216176	0.802176	0.422451	0.547824
FTL_1251	2693.122	-0.88828	0.076248	-11.6499	2.30E-31	5.63E-30
FTL_1252	2524.045	0.108736	0.074117	1.46709	0.142352	0.230747
FTL_1253	903.9743	0.271187	0.107926	2.512703	0.011981	0.028737
FTL_1254	185.2903	0.026385	0.199596	0.132192	0.894833	0.927836
FTL_1255	314.6104	0.112673	0.162126	0.69497	0.487074	0.608086
FTL_1256	701.7223	-0.20717	0.125224	-1.65442	0.098041	0.169575
FTL_1257	475.4709	-0.36505	0.137569	-2.6536	0.007964	0.020009
FTL_1258	2312.913	0.103943	0.080299	1.294448	0.195511	0.298615
FTL_1259	533.4653	0.109474	0.136717	0.800733	0.423286	0.547957
FTL_1260	266.3478	0.383701	0.174292	2.20148	0.027702	0.059488
FTL_1261	555.9989	-0.2914	0.128857	-2.26141	0.023734	0.052137
FTL_1262	2351.273	0.048532	0.075709	0.641031	0.521503	0.640722
FTL_1263	959.159	0.183418	0.101787	1.801981	0.071548	0.131962
FTL_1264	601	0.025435	0.144403	0.176136	0.860187	0.906172
FTL_1265	7104.273	0.35589	0.05998	5.933493	3E-09	1.95E-08
FTL_1266	9188.836	-0.75859	0.057444	-13.2056	8.15E-40	2.64E-38
FTL_1267	3080.662	-1.07653	0.16914	-6.36471	1.96E-10	1.45E-09
FTL_1268	161.7998	-0.63317	0.21877	-2.89422	0.003801	0.010656
FTL_1269	235.4395	-0.09577	0.1839	-0.52076	0.602537	0.70724
FTL_1270	478.6345	0.728773	0.13746	5.301708	1.15E-07	6.21E-07
FTL_1271	2985.212	0.445212	0.070658	6.300952	2.96E-10	2.15E-09
FTL_1272	8920.89	0.0337	0.059126	0.569959	0.568706	0.681227
FTL_1273	8037.379	0.030472	0.059935	0.508421	0.611158	0.713788
FTL_1274	3096.169	0.036328	0.1786	0.203403	0.83882	0.889475
FTL_1275	2855.833	-0.34155	0.070794	-4.8245	1.40E-06	6.81E-06
FTL_1276	759.0422	-0.14647	0.111888	-1.30908	0.190507	0.294696
FTL_1277	710.5759	0.450078	0.118473	3.798998	0.000145	0.000545
FTL_1278	490.2573	0.181206	0.136495	1.327566	0.184322	0.287199

FTL_1279	458.3848	-0.07946	0.137395	-0.5783	0.56306	0.675672
FTL_1280	82.84257	0.065694	0.263527	0.249289	0.803137	0.86767
FTL_1281	205.8612	0.047652	0.192439	0.247622	0.804427	0.868366
FTL_1282	1574.094	0.07457	0.085092	0.876351	0.380839	0.50661
FTL_1283	4421.865	-0.15522	0.064798	-2.3955	0.016598	0.038303
FTL_1284	3153.831	-0.1341	0.070634	-1.89854	0.057626	0.109581
FTL_1285	4009.041	0.108459	0.073895	1.467744	0.142174	0.230645
FTL_1286	1116.887	0.144465	0.100383	1.439135	0.150112	0.240802
FTL_1287	5110.766	0.15101	0.069545	2.17142	0.029899	0.06293
FTL_1288	270.3836	-0.53851	0.19036	-2.8289	0.004671	0.01267
FTL_1289	85.6972	0.859715	0.269806	3.186415	0.00144	0.004448
FTL_1290	833.2386	0.206011	0.110971	1.85644	0.063391	0.118858
FTL_1291	61.62432	0.369815	0.283211	1.30579	0.191624	0.295145
FTL_1292	56.4644	0.643474	0.293985	2.1888	0.028611	0.06105
FTL_1293	1704.885	0.128827	0.086267	1.493364	0.135342	0.221529
FTL_1294	1685.726	-0.08499	0.088657	-0.95859	0.337764	0.459963
FTL_1295	798.2271	0.052151	0.114373	0.455972	0.64841	0.743047
FTL_1296	654.7839	0.083376	0.120282	0.693173	0.488201	0.609115
FTL_1297	120.9705	0.511567	0.243933	2.097163	0.035979	0.074172
FTL_1298	969.9326	0.203772	0.101855	2.000612	0.045434	0.089835
FTL_1299	211.6446	0.041309	0.189598	0.21788	0.827523	0.879599
FTL_1300	155.1622	-0.10966	0.212359	-0.5164	0.605574	0.70974
FTL_1301	140.2358	0.104237	0.21942	0.475058	0.634746	0.732526
FTL_1302	2237.54	-0.06229	0.081349	-0.76568	0.443867	0.567539
FTL_1303	6748.309	0.001658	0.067235	0.024654	0.980331	0.985725
FTL_1304	2895.12	-0.12708	0.076862	-1.65328	0.098274	0.169699
FTL_1305	986.3275	-0.01642	0.100803	-0.16293	0.870577	0.913908
FTL_1306	3747.617	-0.26885	0.066712	-4.03006	5.58E-05	0.000223
FTL_1307	563.1431	0.236376	0.125542	1.88285	0.059721	0.113137
FTL_1308	7528.529	0.412996	0.058373	7.075125	1.49E-12	1.30E-11
FTL_1309	14664.65	0.348343	0.05903	5.901163	3.61E-09	2.35E-08
FTL_1310	4771.128	0.12572	0.065771	1.911476	0.055943	0.107296
FTL_1311	6886.851	-0.03506	0.065045	-0.53901	0.589882	0.697038
FTL_1312	445.1631	-0.87858	0.142657	-6.1587	7.33E-10	5.07E-09
FTL_1313	477.3511	-0.89063	0.146008	-6.09986	1.06E-09	7.16E-09
FTL_1314	169.7131	0.159142	0.212267	0.749725	0.45342	0.576645
FTL_1315	83.61928	-1.47764	0.267406	-5.52585	3.28E-08	1.90E-07
FTL_1316	966.2915	0.240321	0.102648	2.341226	0.019221	0.043555
FTL_1317	4747.781	-0.76964	0.080497	-9.56108	1.17E-21	1.80E-20
FTL_1318	59.92964	0.117322	0.285469	0.41098	0.681087	0.771259
FTL_1319	144.4412	0.052865	0.21827	0.242202	0.808623	0.869627
FTL_1320	381.2032	0.345851	0.16033	2.15712	0.030996	0.064949
FTL_1321	76.20273	0.830659	0.27552	3.014876	0.002571	0.0075

FTL_1322	65.92549	0.3651	0.283246	1.288989	0.197402	0.300362
FTL_1323	176.275	0.350366	0.210645	1.663297	0.096253	0.167216
FTL_1324	30.47302	0.321492	0.325961	0.986289	0.323991	0.445738
FTL_1325	269.802	0.721073	0.17475	4.126308	3.69E-05	0.000152
FTL_1326	262.0747	0.116508	0.181128	0.643236	0.520071	0.639656
FTL_1327	1073.748	-0.18966	0.101756	-1.86391	0.062335	0.117205
FTL_1328	28172.16	-0.51009	0.051106	-9.98113	1.84E-23	3.25E-22
FTL_1329	736.6773	-0.07231	0.122136	-0.59208	0.553797	0.668115
FTL_1330	2208.913	0.405772	0.081354	4.987731	6.11E-07	3.06E-06
FTL_1331	2622.034	0.578561	0.077393	7.475576	7.69E-14	7.39E-13
FTL_1332	1676.444	0.617575	0.083906	7.360335	1.83E-13	1.72E-12
FTL_1333	3837.963	0.602231	0.066604	9.041928	1.54E-19	2.10E-18
FTL_1334	3785.756	0.204715	0.068435	2.991386	0.002777	0.00802
FTL_1335	1081.333	-0.27642	0.103481	-2.67119	0.007558	0.019182
FTL_1336	1982.735	-0.38576	0.081636	-4.7254	2.30E-06	1.09E-05
FTL_1337	1287.126	-0.34252	0.106213	-3.22488	0.00126	0.003964
FTL_1338	1234.742	-0.14153	0.094151	-1.50327	0.132769	0.217849
FTL_1339	1362.064	-0.38477	0.091502	-4.205	2.61E-05	0.000111
FTL_1340	1388.329	-0.36378	0.092353	-3.93904	8.18E-05	0.000316
FTL_1341	386.5993	-0.57842	0.146614	-3.9452	7.97E-05	0.000308
FTL_1342	766.1359	-0.07073	0.11115	-0.63637	0.524537	0.642116
FTL_1343	374.5768	0.023029	0.151549	0.151955	0.879222	0.918379
FTL_1344	302.5874	-0.25462	0.164889	-1.54421	0.122536	0.203721
FTL_1345	1253.899	-0.33222	0.097592	-3.40419	0.000664	0.002212
FTL_1346	265.5961	-0.02664	0.177895	-0.14974	0.880968	0.919391
FTL_1347	59.07902	-0.02672	0.287083	-0.09309	0.925835	0.948002
FTL_1348	164.8478	0.0245	0.205632	0.119146	0.90516	0.932533
FTL_1349	847.2572	0.269768	0.108087	2.495847	0.012566	0.029961
FTL_1350	5833.936	0.062896	0.063331	0.993135	0.320644	0.442361
FTL_1351	279.274	-0.50926	0.167682	-3.03704	0.002389	0.007041
FTL_1352	423.12	-0.10216	0.144421	-0.70738	0.479328	0.601475
FTL_1353	43.54278	-0.03894	0.308303	-0.12631	0.899483	0.930389
FTL_1354	1571.654	-0.41516	0.088552	-4.68832	2.75E-06	1.29E-05
FTL_1355	1200.208	0.259895	0.098403	2.641131	0.008263	0.020657
FTL_1356	1466.636	-0.05773	0.096505	-0.59817	0.54973	0.665127
FTL_1357	1110.91	0.092605	0.103351	0.896019	0.370243	0.495464
FTL_1358	3609.103	0.281451	0.06922	4.066048	4.78E-05	0.000193
FTL_1359	295.8787	0.119203	0.166705	0.715057	0.474574	0.597303
FTL_1360	418.478	-0.45765	0.14705	-3.11223	0.001857	0.005587
FTL_1361	2201.771	0.047566	0.100268	0.474393	0.63522	0.732526
FTL_1362	2079.599	-0.01902	0.078285	-0.24301	0.807997	0.869418
FTL_1363	1261.214	-0.05506	0.101778	-0.54098	0.588519	0.696246
FTL_1364	5019.862	-0.1872	0.067003	-2.79396	0.005207	0.013917

FTL_1365	2029.652		0.090662	0.082309	1.101478	0.270689	0.386648
FTL_1366	1404.042		-0.34101	0.106368	-3.20594	0.001346	0.004195
FTL_1367	1025.453		-0.40387	0.100036	-4.03726	5.41E-05	0.000217
FTL_1368	655.148		-0.55684	0.119019	-4.67856	2.89E-06	1.35E-05
FTL_1369	894.323		0.18142	0.105335	1.722308	0.085014	0.150819
FTL_1370	1211.432		0.047499	0.093207	0.509613	0.610323	0.713226
FTL_1371	1143.348		-0.29567	0.09788	-3.02074	0.002522	0.007378
FTL_1372	4218.504		-0.90979	0.064454	-14.1153	3.06E-45	1.34E-43
FTL_1373	57.41901		-0.23758	0.289441	-0.82084	0.41174	0.536704
FTL_1374	1635.916		0.076552	0.088571	0.864307	0.387419	0.513324
FTL_1375	193.226		0.057892	0.195867	0.295569	0.767559	0.839387
FTL_1376	552.8165		0.05075	0.127368	0.398449	0.690299	0.779495
FTL_1377	194.7377		0.266621	0.194027	1.374146	0.169397	0.266213
FTL_1378	62.0424		0.268284	0.284721	0.942271	0.346054	0.468396
FTL_1379	286.4849		0.279031	0.168099	1.65992	0.096931	0.168022
FTL_1380	60.14714		0.322346	0.286721	1.124249	0.260908	0.376472
FTL_1381	124.7693		-0.37435	0.227347	-1.64658	0.099644	0.171623
FTL_1382	420.4733		-0.32636	0.15206	-2.14627	0.031852	0.066275
FTL_1383	775.9855		-0.28316	0.111992	-2.5284	0.011458	0.027648
FTL_1384	396.8247		-0.19055	0.147602	-1.29094	0.196724	0.299784
FTL_1385	255.5776		0.469274	0.175952	2.667055	0.007652	0.019371
FTL_1386	232.2989		0.019154	0.184297	0.103928	0.917227	0.941672
FTL_1387	378.689		0.169163	0.151413	1.11723	0.263896	0.379177
FTL_1388	2575.96		-0.18279	0.073772	-2.4778	0.01322	0.031224
FTL_1389	4035.293		0.053012	0.069771	0.759806	0.447371	0.571293
FTL_1390	7154.75		0.067033	0.060691	1.104501	0.269376	0.385371
FTL_1391	3399.783		0.103344	0.073011	1.415468	0.156931	0.250542
FTL_1392	20057.4		-0.35905	0.05299	-6.7758	1.24E-11	1.02E-10
FTL_1393	20584.06		-0.00797	0.064135	-0.12429	0.901086	0.931199
FTL_1394	4193.818		-0.55485	0.064582	-8.59142	8.59E-18	1.05E-16
FTL_1395	2771.949		-0.6822	0.072344	-9.43001	4.10E-21	6.06E-20
FTL_1396	4395.591		-0.34404	0.068164	-5.04725	4.48E-07	2.29E-06
FTL_1397	5039.208		-0.36761	0.065434	-5.61803	1.93E-08	1.14E-07
FTL_1398	1807.139		-0.18696	0.087	-2.14901	0.031634	0.06589
FTL_1399	1313.139		-0.19897	0.090773	-2.19193	0.028384	0.060695
FTL_1400	663.6861		-0.04071	0.11828	-0.34415	0.730731	0.811923
FTL_1401	84.22856		0.112387	0.264456	0.424974	0.670856	0.76096
FTL_1402	889.8541		0.059527	0.107974	0.551305	0.581425	0.69286
FTL_1403	1066.871		-0.06151	0.120589	-0.51009	0.609985	0.713226
FTL_1404	3669.682		-0.37467	0.086898	-4.31156	1.62E-05	7.01E-05
FTL_1405	4670.217		-0.2351	0.069096	-3.40249	0.000668	0.002218
FTL_1406	24312.73		0.1503	0.062629	2.399854	0.016402	0.037893
FTL_1407	23491.3		0.059146	0.052451	1.127649	0.259468	0.375386

FTL_1408	974.7896	-0.15017	0.107321	-1.39923	0.161745	0.257408
FTL_1409	1585.795	-0.26753	0.085761	-3.11944	0.001812	0.005468
FTL_1410	1201.44	-0.21886	0.103014	-2.12459	0.033621	0.069597
FTL_1411	1149.563	-0.72544	0.101593	-7.1407	9.29E-13	8.22E-12
FTL_1412	2734.723	-0.6613	0.077177	-8.56862	1.05E-17	1.27E-16
FTL_1413	2129.543	-4.8891	0.111549	-43.8293	0	0
FTL_1414	5459.592	-0.03277	0.061876	-0.52965	0.596358	0.703018
FTL_1415	2235.475	0.016334	0.077335	0.211211	0.832722	0.88419
FTL_1416	3424.979	0.196203	0.068208	2.876526	0.004021	0.011121
FTL_1417	2769.004	0.685581	0.075558	9.073518	1.15E-19	1.59E-18
FTL_1418	2090.072	0.040795	0.083054	0.491183	0.623297	0.722091
FTL_1419	6046.838	0.134901	0.061413	2.196619	0.028048	0.060038
FTL_1420	1363.346	-0.53177	0.093028	-5.71625	1.09E-08	6.67E-08
FTL_1421	534.0781	-0.24466	0.13317	-1.83722	0.066178	0.123393
FTL_1422	521.6053	-0.12534	0.150868	-0.83082	0.406075	0.531733
FTL_1423	606.9348	-0.1834	0.146506	-1.25185	0.210624	0.315231
FTL_1424	759.4192	-0.20564	0.113971	-1.80434	0.071177	0.131495
FTL_1425	760.0416	-0.12984	0.141121	-0.92004	0.357551	0.481042
FTL_1426	1186.167	0.051104	0.223908	0.228239	0.81946	0.875416
FTL_1427	2368.261	0.050013	0.077715	0.643537	0.519875	0.639656
FTL_1428	1002.151	-0.09701	0.103583	-0.93657	0.348979	0.47077
FTL_1429	1018.899	-0.12892	0.102134	-1.26231	0.206839	0.311959
FTL_1430	2171.795	-0.2036	0.083174	-2.44793	0.014368	0.03362
FTL_1431	1128.03	0.059968	0.102335	0.585993	0.55788	0.670658
FTL_1432	2205.086	0.028326	0.079175	0.357762	0.720522	0.805517
FTL_1433	1869.388	0.17159	0.088348	1.942215	0.052111	0.101006
FTL_1434	737.7893	0.121587	0.121291	1.002442	0.31613	0.439434
FTL_1435	29.3419	0.56197	0.325792	1.724934	0.084539	0.150375
FTL_1436	14.60789	0.423457	0.326911	1.295326	0.195208	0.298615
FTL_1437	38.03527	0.310912	0.317388	0.979594	0.327287	0.449042
FTL_1438	15.50733	0.105906	0.331071	0.31989	0.749052	0.82725
FTL_1439	38.93086	0.790612	0.314706	2.512222	0.011997	0.028742
FTL_1440	50.28873	0.218133	0.303192	0.719455	0.471861	0.594633
FTL_1441	371.3168	-0.03651	0.160192	-0.22793	0.8197	0.875416
FTL_1442	6732.94	-0.11987	0.065104	-1.84119	0.065594	0.122532
FTL_1443	2086.711	0.212958	0.084566	2.518247	0.011794	0.028357
FTL_1444	961.9923	-0.06135	0.103571	-0.59236	0.553609	0.668115
FTL_1445	182.202	0.124109	0.201499	0.615929	0.537942	0.654914
FTL_1446	826.9801	0.255351	0.109592	2.330021	0.019805	0.044528
FTL_1447	164.5816	-0.02641	0.241761	-0.10923	0.91302	0.938706
FTL_1448	878.9302	-0.17118	0.111628	-1.5335	0.125153	0.207043
FTL_1449	696.9814	0.018357	0.125382	0.146406	0.883601	0.921181
FTL_1450	2931.664	-0.12457	0.071462	-1.74319	0.0813	0.146035

FTL_1451	1233.468	-0.17771	0.093516	-1.9003	0.057394	0.109347
FTL_1452	4629.201	-0.03901	0.073807	-0.52858	0.597098	0.703018
FTL_1453	7067.827	0.250514	0.063923	3.918979	8.89E-05	0.000342
FTL_1454	213.63	-0.096	0.189121	-0.50763	0.611711	0.714018
FTL_1455	295.6293	0.160578	0.166004	0.967313	0.333388	0.455047
FTL_1456	174.7168	-0.17137	0.202088	-0.84799	0.396443	0.522867
FTL_1457	132.4531	-0.25665	0.241205	-1.06403	0.287314	0.405833
FTL_1458	12699.34	-0.01459	0.054572	-0.26739	0.789166	0.855569
FTL_1459	789.9356	-0.06174	0.111727	-0.55263	0.580518	0.692488
FTL_1460	1293.812	-0.10755	0.098821	-1.08836	0.276435	0.393231
FTL_1461	10433.35	-0.01052	0.058312	-0.18049	0.856768	0.903392
FTL_1462	1124.381	0.167257	0.096752	1.728711	0.083861	0.149698
FTL_1463	141.1728	0.0961	0.218304	0.440212	0.659784	0.75147
FTL_1464	4009.612	-0.21242	0.067536	-3.14525	0.001659	0.005069
FTL_1465	110.7904	-0.42227	0.237995	-1.77429	0.076015	0.138146
FTL_1466	242.3972	-0.05434	0.176847	-0.30726	0.758649	0.834639
FTL_1467	140.9208	0.133223	0.218354	0.610127	0.541778	0.659185
FTL_1468	4009.378	-0.2126	0.067557	-3.14693	0.00165	0.005055
FTL_1469	110.7904	-0.42227	0.237995	-1.77429	0.076015	0.138146
FTL_1470	242.3972	-0.05434	0.176847	-0.30726	0.758649	0.834639
FTL_1471	141.9186	0.152141	0.223049	0.682099	0.495177	0.61667
FTL_1472	341.1294	0.116191	0.156045	0.7446	0.456513	0.58002
FTL_1473	7213.387	-0.13575	0.057525	-2.35981	0.018284	0.041716
FTL_1474	3766.301	-0.12654	0.082459	-1.53455	0.124893	0.207
FTL_1475	2308.126	-0.00329	0.079854	-0.04126	0.967091	0.974851
FTL_1476	4021.671	-0.34884	0.066518	-5.24423	1.57E-07	8.43E-07
FTL_1477	712.3131	0.080769	0.125228	0.644973	0.518945	0.639442
FTL_1478	11316.48	0.148041	0.054347	2.724026	0.006449	0.016662
FTL_1479	8098.734	-0.1571	0.057689	-2.72322	0.006465	0.016681
FTL_1480	1788.723	0.245313	0.082688	2.966741	0.00301	0.00863
FTL_1481	1315.212	-0.12065	0.093194	-1.29457	0.195469	0.298615
FTL_1482	1959.909	-0.33253	0.081785	-4.06597	4.78E-05	0.000193
FTL_1483	1672.133	-0.33806	0.083974	-4.02574	5.68E-05	0.000227
FTL_1484	607.4516	0.068257	0.124139	0.549848	0.582424	0.693526
FTL_1485	1062.615	0.124441	0.102888	1.209477	0.22648	0.335711
FTL_1486	1447.121	-0.03614	0.089784	-0.40254	0.68729	0.777407
FTL_1487	1935.138	-0.27845	0.084255	-3.30485	0.00095	0.003056
FTL_1488	1033.099	-0.1424	0.107674	-1.3225	0.186001	0.289366
FTL_1489	8555.153	-0.16799	0.056908	-2.95194	0.003158	0.008978
FTL_1490	7497.316	-0.05289	0.064179	-0.82407	0.409902	0.535642
FTL_1491	5878.924	-0.03629	0.062656	-0.57922	0.562444	0.675603
FTL_1492	1633.203	-0.32192	0.100766	-3.19469	0.0014	0.004342
FTL_1493	1189.208	0.292701	0.097117	3.013893	0.002579	0.007513

FTL_1494	1847.618	0.167549	0.087736	1.909694	0.056173	0.107633
FTL_1495	1514.929	0.115898	0.088626	1.307722	0.190968	0.294696
FTL_1496	1208.462	0.104654	0.102744	1.018587	0.308399	0.429877
FTL_1497	864.171	0.075918	0.107204	0.708161	0.478845	0.601475
FTL_1498	875.6437	0.335604	0.1092	3.073309	0.002117	0.006304
FTL_1499	1683.875	0.121647	0.092969	1.308465	0.190715	0.294696
FTL_1500	126.5406	0.028069	0.227239	0.123521	0.901694	0.931226
FTL_1501	21.76479	0.131967	0.332636	0.396733	0.691565	0.780486
FTL_1502	339.4745	-0.01386	0.156735	-0.08844	0.929531	0.949781
FTL_1503	1828.717	-0.9799	0.083252	-11.7702	5.56E-32	1.38E-30
FTL_1504	30318.79	0.014798	0.050239	0.294553	0.768336	0.83978
FTL_1505	839.8511	0.236785	0.113511	2.086011	0.036978	0.075765
FTL_1506	187.5718	-0.46317	0.197517	-2.34495	0.019029	0.043219
FTL_1507	202.3826	-0.75475	0.1911	-3.94952	7.83E-05	0.000304
FTL_1508	80.98139	-0.55646	0.269043	-2.06829	0.038613	0.078474
FTL_1509	480.3105	-1.44177	0.142625	-10.1088	5.05E-24	9.14E-23
FTL_1510	3127.009	-0.57038	0.077857	-7.32601	2.37E-13	2.17E-12
FTL_1511	5844.904	-0.36919	0.061839	-5.97014	2.37E-09	1.57E-08
FTL_1512	470.7296	-0.23409	0.134807	-1.73648	0.082479	0.147888
FTL_1513	115.9156	-0.19028	0.238175	-0.79889	0.424355	0.548876
FTL_1514	39.08062	-0.64496	0.318787	-2.02318	0.043055	0.085768
FTL_1515	47.79752	0.324142	0.306807	1.0565	0.29074	0.409808
FTL_1516	127.4586	0.045942	0.226163	0.203137	0.839028	0.889475
FTL_1517	310.9449	0.252711	0.161411	1.565633	0.117435	0.196867
FTL_1518	979.2217	0.045488	0.105075	0.432907	0.665082	0.756117
FTL_1519	403.5114	-0.12608	0.152094	-0.82898	0.407114	0.532747
FTL_1520	2205.735	0.081814	0.082122	0.996251	0.319128	0.441465
FTL_1521	10532.1	-0.59108	0.057158	-10.3412	4.59E-25	8.89E-24
FTL_1522	3330.859	-0.94594	0.071365	-13.255	4.22E-40	1.39E-38
FTL_1523	3512.61	-0.75214	0.07896	-9.52551	1.64E-21	2.50E-20
FTL_1524	1408.751	-0.58288	0.09843	-5.92173	3.19E-09	2.08E-08
FTL_1525	1740.889	-0.21315	0.08234	-2.58863	0.009636	0.023677
FTL_1526	1212.405	0.023263	0.098589	0.235957	0.813466	0.871901
FTL_1527	13765.02	0.068202	0.058365	1.168557	0.242582	0.355128
FTL_1528	2215.097	0.119143	0.08282	1.438584	0.150268	0.240861
FTL_1529	353.3544	0.205831	0.153584	1.34019	0.180184	0.281624
FTL_1530	1749.196	0.45482	0.085667	5.309139	1.10E-07	6E-07
FTL_1531	3810.944	0.054404	0.065472	0.830945	0.406005	0.531733
FTL_1532	1329.271	-0.16362	0.103907	-1.57469	0.115329	0.193822
FTL_1533	940.4523	-0.17652	0.104909	-1.6826	0.092453	0.161733
FTL_1534	2585.217	-0.23881	0.07291	-3.27545	0.001055	0.00335
FTL_1535	5104.716	-0.23507	0.070886	-3.31623	0.000912	0.002946
FTL_1536	1802.353	-0.07503	0.082434	-0.91017	0.362734	0.486712

FTL_1537	24131.57	0.09802	0.052562	1.864839	0.062204	0.117109
FTL_1538	4559.79	0.225197	0.063962	3.520805	0.00043	0.001486
FTL_1539	3295.22	-0.34046	0.071111	-4.78774	1.69E-06	8.07E-06
FTL_1540	1015.586	-0.4898	0.101562	-4.82271	1.42E-06	6.86E-06
FTL_1541	2164.688	-0.2017	0.076862	-2.6242	0.008685	0.021473
FTL_1542	8920.438	-0.00393	0.058749	-0.06684	0.946713	0.961057
FTL_1543	1903.904	-0.16094	0.082739	-1.94517	0.051755	0.100509
FTL_1544	1249.508	0.052081	0.092227	0.564699	0.572279	0.68469
FTL_1545	1746.887	-0.72104	0.085812	-8.40254	4.37E-17	5.05E-16
FTL_1546	1736.432	-0.63513	0.083659	-7.59186	3.15E-14	3.08E-13
FTL_1547	16838.53	-0.27877	0.052254	-5.33489	9.56E-08	5.26E-07
FTL_1548	1723.585	-0.15221	0.083362	-1.8259	0.067865	0.125838
FTL_1549	1817.636	-0.18035	0.081181	-2.22154	0.026314	0.056934
FTL_1550	1904.164	-0.39962	0.080055	-4.99177	5.98E-07	3.01E-06
FTL_1551	139.0915	0.004479	0.220012	0.020357	0.983759	0.98769
FTL_1552	4952.539	-0.01953	0.065959	-0.29607	0.767178	0.839387
FTL_1553	13715.53	-0.042	0.057708	-0.72776	0.466763	0.589688
FTL_1554	17159.78	0.095794	0.064005	1.496661	0.134482	0.2203
FTL_1555	948.5892	-0.00452	0.1138	-0.03973	0.968305	0.97508
FTL_1556	967.4976	-0.16271	0.111923	-1.45379	0.146004	0.235528
FTL_1557	358.0979	0.286337	0.155317	1.84356	0.065247	0.121997
FTL_1558	237.9707	0.353179	0.184226	1.9171	0.055225	0.106223
FTL_1559	242.898	0.185813	0.191309	0.971275	0.331412	0.453155
FTL_1560	62.99997	0.217102	0.289527	0.749852	0.453344	0.576645
FTL_1561	31.24891	0.46883	0.324384	1.445292	0.148376	0.238207
FTL_1562	216.3357	0.677611	0.189324	3.57911	0.000345	0.001205
FTL_1563	90.30482	0.320578	0.267942	1.196448	0.231522	0.341924
FTL_1564	57.08954	-0.03476	0.288639	-0.12043	0.904143	0.932533
FTL_1565	168.6346	-0.07921	0.214393	-0.36945	0.711794	0.798385
FTL_1566	389.5436	-0.11754	0.146817	-0.80059	0.423372	0.547957
FTL_1567	336.238	-0.06065	0.155559	-0.38991	0.696601	0.784848
FTL_1568	139.2276	-0.02944	0.221099	-0.13317	0.894058	0.927753
FTL_1569	972.563	-0.12697	0.112934	-1.12425	0.260908	0.376472
FTL_1570	1250.928	0.009332	0.092635	0.100739	0.919758	0.943221
FTL_1571	4347.13	0.121915	0.078566	1.551746	0.120723	0.201162
FTL_1572	130.651	0.21801	0.224789	0.969844	0.332124	0.453821
FTL_1573	196.4402	-0.00934	0.213326	-0.04379	0.965074	0.973794
FTL_1574	160.7966	0.175353	0.221371	0.792122	0.42829	0.55246
FTL_1575	950.1981	0.161103	0.110687	1.455475	0.145538	0.234965
FTL_1576	1252.793	-0.47766	0.094473	-5.05606	4.28E-07	2.19E-06
FTL_1577	758.831	-0.32021	0.113812	-2.81348	0.004901	0.013205
FTL_1578	783.6306	-0.2303	0.113304	-2.03259	0.042094	0.084021
FTL_1579	6573.686	0.012864	0.069858	0.184145	0.8539	0.901438

FTL_1580	2220.877	-0.47215	0.076991	-6.13261	8.64E-10	5.91E-09
FTL_1581	1599.991	0.544762	0.091548	5.950572	2.67E-09	1.76E-08
FTL_1582	2470.412	0.116827	0.079084	1.477249	0.139609	0.226851
FTL_1583	3674.112	0.198679	0.067727	2.933541	0.003351	0.009474
FTL_1584	1630.808	-0.02722	0.089867	-0.30289	0.761976	0.836467
FTL_1585	2642.568	0.070165	0.0749	0.936782	0.348871	0.47077
FTL_1586	876.5143	0.068402	0.106116	0.644597	0.519188	0.639442
FTL_1587	1021.413	-0.19647	0.099493	-1.97467	0.048306	0.09445
FTL_1588	371.4558	-0.04291	0.152543	-0.28128	0.778499	0.847768
FTL_1589	147.9954	0.022239	0.215432	0.103232	0.917779	0.941672
FTL_1590	3115.841	-0.10243	0.070806	-1.44659	0.148013	0.23796
FTL_1591	16167.03	0.476126	0.05692	8.36477	6.02E-17	6.92E-16
FTL_1592	5605.234	0.34161	0.060252	5.669663	1.43E-08	8.64E-08
FTL_1593	2266.528	0.0777	0.077944	0.996871	0.318827	0.441465
FTL_1594	1471.764	-0.4596	0.089761	-5.12024	3.05E-07	1.59E-06
FTL_1595	1038.404	-0.48167	0.099247	-4.85326	1.21E-06	5.95E-06
FTL_1596	1605.063	-0.05283	0.087198	-0.60584	0.544623	0.661046
FTL_1597	6957.07	-0.0372	0.059708	-0.62308	0.533233	0.650758
FTL_1598	8316.279	-0.11834	0.064353	-1.83892	0.065927	0.123039
FTL_1599	2158.457	-0.01158	0.079942	-0.1448	0.884873	0.92167
FTL_1600	407.9766	-0.03358	0.145131	-0.23139	0.817012	0.874438
FTL_1601	1281.259	-0.70843	0.093214	-7.60007	2.96E-14	2.90E-13
FTL_1602	5471.763	-0.66268	0.062456	-10.6104	2.67E-26	5.58E-25
FTL_1603	1137.776	-0.76864	0.103818	-7.40373	1.32E-13	1.25E-12
FTL_1604	2575.816	-0.61389	0.074833	-8.20343	2.34E-16	2.61E-15
FTL_1605	3932.084	-0.79169	0.066434	-11.9169	9.67E-33	2.49E-31
FTL_1606	3657.187	-0.23267	0.071691	-3.24542	0.001173	0.003706
FTL_1607	473.8795	0.078795	0.143472	0.549202	0.582867	0.693643
FTL_1608	1096.11	-0.29324	0.100697	-2.91216	0.003589	0.010076
FTL_1609	3391.65	-0.09157	0.0714	-1.28247	0.199678	0.303136
FTL_1610	893.1178	0.20685	0.10816	1.912441	0.05582	0.107161
FTL_1611	3161.128	-0.19801	0.070745	-2.79897	0.005127	0.013721
FTL_1612	824.2348	-0.36136	0.109463	-3.30118	0.000963	0.003091
FTL_1613	1818.908	-0.2209	0.083731	-2.63827	0.008333	0.020807
FTL_1614	1961.461	-0.1372	0.079693	-1.72165	0.085133	0.150898
FTL_1615	2247.809	-0.18313	0.076832	-2.3835	0.017149	0.039439
FTL_1616	8378.402	-0.06735	0.057578	-1.16979	0.242087	0.355004
FTL_1617	4988.223	-0.1306	0.063395	-2.06016	0.039384	0.079479
FTL_1618	430.1066	-0.20654	0.146458	-1.41023	0.158472	0.2528
FTL_1619	191.9481	-0.1208	0.204913	-0.58951	0.555519	0.66942
FTL_1620	462.0585	-0.23607	0.150007	-1.57375	0.115546	0.194025
FTL_1621	3464.08	0.243366	0.069542	3.499543	0.000466	0.001604
FTL_1622	2181.932	0.27449	0.078237	3.508455	0.000451	0.001554

FTL_1623	969.6496	-0.17097	0.106631	-1.60338	0.10885	0.18488
FTL_1624	1846.126	0.308007	0.085873	3.586753	0.000335	0.001177
FTL_1625	156.9735	-0.3124	0.223051	-1.4006	0.161334	0.257163
FTL_1626	43.87483	-0.1375	0.312446	-0.44009	0.659873	0.75147
FTL_1627	982.8311	0.212861	0.102889	2.068841	0.038561	0.078449
FTL_1628	169.7167	0.536773	0.205007	2.618315	0.008837	0.02182
FTL_1629	408.3093	0.447747	0.148705	3.010985	0.002604	0.007575
FTL_1630	891.467	0.24414	0.114181	2.13818	0.032502	0.067419
FTL_1631	188.1705	0.337842	0.199399	1.694301	0.090208	0.158218
FTL_1632	54.57271	0.373051	0.291993	1.277602	0.20139	0.305504
FTL_1633	346.5983	0.372528	0.15775	2.361513	0.018201	0.041572
FTL_1634	801.5644	0.100361	0.111895	0.896916	0.369764	0.495464
FTL_1635	288.267	0.141076	0.172818	0.816325	0.414314	0.539011
FTL_1636	111.7346	0.578222	0.248739	2.324612	0.020093	0.045124
FTL_1637	5601.224	0.040844	0.061286	0.666441	0.505129	0.627122
FTL_1638	1175.477	-0.12365	0.094374	-1.31024	0.190114	0.294696
FTL_1639	966.2485	-0.17539	0.102967	-1.70334	0.088504	0.155909
FTL_1640	1095.045	-0.80443	0.101399	-7.93327	2.13E-15	2.28E-14
FTL_1641	417.316	-0.37365	0.169044	-2.21038	0.027079	0.0584
FTL_1642	945.6615	-0.17763	0.113976	-1.55851	0.119113	0.199018
FTL_1643	1257.74	0.147198	0.092747	1.587094	0.112491	0.189847
FTL_1644	4351.953	-0.0787	0.067289	-1.16964	0.242145	0.355004
FTL_1645	1258.756	0.032451	0.102917	0.315316	0.752521	0.829259
FTL_1646	1188.012	0.37354	0.095841	3.89749	9.72E-05	0.000371
FTL_1647	149.1431	0.585123	0.220831	2.649639	0.008058	0.02017
FTL_1648	80.60215	0.340682	0.26267	1.296995	0.194633	0.29818
FTL_1649	47.78281	0.289098	0.306729	0.94252	0.345927	0.468396
FTL_1650	33.44	0.178176	0.321381	0.554406	0.579301	0.691446
FTL_1651	60.36721	0.242019	0.287815	0.840884	0.400413	0.526377
FTL_1652	22.12497	0.399636	0.332986	1.200157	0.230078	0.340393
FTL_1653	29.84243	-0.10441	0.328073	-0.31824	0.7503	0.828126
FTL_1654	195.7018	0.67381	0.199495	3.377585	0.000731	0.002413
FTL_1655	139.4289	0.109921	0.219683	0.50036	0.616822	0.718731
FTL_1656	2854.164	-0.21404	0.074413	-2.87639	0.004022	0.011121
FTL_1657	2170.549	-0.00557	0.078771	-0.07073	0.943616	0.959772
FTL_1658	4735.111	-0.09807	0.064691	-1.51599	0.129521	0.213042
FTL_1659	4107.711	0.180131	0.070071	2.570704	0.010149	0.024817
FTL_1660	546.9144	-0.19069	0.130021	-1.46663	0.142477	0.230764
FTL_1661	1468.865	-0.58188	0.089438	-6.506	7.72E-11	6.01E-10
FTL_1662	1982.299	-0.22046	0.079069	-2.78827	0.005299	0.014145
FTL_1663	1816.895	-0.18674	0.082406	-2.26615	0.023442	0.051735
FTL_1664	4056.86	-0.02505	0.071141	-0.35208	0.724779	0.808439
FTL_1665	2190.966	-0.13374	0.080502	-1.66138	0.096638	0.167739

FTL_1666	4634.627	-0.20102	0.065427	-3.07241	0.002123	0.006314
FTL_1667	1678.344	-0.15098	0.084291	-1.79111	0.073276	0.134384
FTL_1668	7821.21	-0.37717	0.06054	-6.23006	4.66E-10	3.30E-09
FTL_1669	639.63	-0.39138	0.122741	-3.18871	0.001429	0.004426
FTL_1670	1199.528	0.029048	0.097672	0.297402	0.766159	0.839387
FTL_1671	4510.142	-0.28322	0.069603	-4.06901	4.72E-05	0.000192
FTL_1672	6329.147	-0.32185	0.06517	-4.93866	7.87E-07	3.91E-06
FTL_1673	4960.189	0.138093	0.064026	2.15681	0.031021	0.064949
FTL_1674	170.7153	0.459804	0.203851	2.255584	0.024097	0.052761
FTL_1675	37.07942	0.438228	0.315441	1.389253	0.164756	0.260141
FTL_1676	55.58205	0.4806	0.29519	1.628106	0.103502	0.177357
FTL_1677	113.4748	0.413803	0.246959	1.675592	0.093818	0.163835
FTL_1678	7489.933	-1.01925	0.058382	-17.4584	2.97E-68	2.49E-66
FTL_1679	78.07229	-0.08811	0.26696	-0.33007	0.74135	0.820547
FTL_1680	154.9188	0.048209	0.216977	0.222183	0.824171	0.876963
FTL_1681	960.1905	0.251436	0.102694	2.448405	0.014349	0.033615
FTL_1682	42.08914	0.397656	0.309978	1.282851	0.199544	0.303136
FTL_1683	3484.79	-0.02285	0.07061	-0.32361	0.746233	0.825043
FTL_1684	2526.359	0.217021	0.082519	2.629939	0.00854	0.021192
FTL_1685	1985.714	-0.43991	0.086766	-5.07012	3.98E-07	2.04E-06
FTL_1686	763.4807	-0.19375	0.115768	-1.67362	0.094204	0.164367
FTL_1687	1051.19	0.252138	0.100251	2.515061	0.011901	0.02858
FTL_1688	248.58	0.524569	0.198998	2.636053	0.008388	0.020891
FTL_1689	1159.052	0.147209	0.095227	1.545876	0.122135	0.203221
FTL_1690	724.5099	0.596232	0.116421	5.121362	3.03E-07	1.59E-06
FTL_1691	275.7175	0.469698	0.172738	2.719142	0.006545	0.016866
FTL_1692	552.2822	0.253366	0.133905	1.892126	0.058474	0.11088
FTL_1693	389.8564	0.444272	0.146473	3.033122	0.00242	0.007123
FTL_1694	470.4768	0.352418	0.138078	2.55231	0.010701	0.026103
FTL_1695	467.7668	0.049304	0.138303	0.356495	0.72147	0.806023
FTL_1696	523.6606	-0.34668	0.142725	-2.42902	0.01514	0.035221
FTL_1697	34.45882	0.418982	0.320121	1.308827	0.190593	0.294696
FTL_1698	31.44033	0.193091	0.322621	0.598506	0.549502	0.665127
FTL_1699	2506.986	0.130099	0.073918	1.760051	0.078399	0.141711
FTL_1700	2328.88	-0.38921	0.077981	-4.99107	6.00E-07	3.02E-06
FTL_1701	6563.693	-0.49529	0.06433	-7.69912	1.37E-14	1.39E-13
FTL_1702	2742.493	-0.42557	0.072566	-5.86464	4.50E-09	2.88E-08
FTL_1703	6914.805	-0.50673	0.060168	-8.42194	3.70E-17	4.35E-16
FTL_1704	664.867	-0.17537	0.117901	-1.48743	0.136902	0.222992
FTL_1705	4046.454	0.127601	0.067415	1.892772	0.058388	0.110822
FTL_1706	928.2752	0.096115	0.115197	0.834354	0.404082	0.529814
FTL_1707	466.5861	-0.31463	0.144402	-2.17885	0.029343	0.062083
FTL_1708	3738.568	0.183502	0.065886	2.785134	0.005351	0.014215

FTL_1709	2705.555	-0.17916	0.074129	-2.41679	0.015658	0.036342
FTL_1710	4592.938	0.059609	0.069722	0.854943	0.392583	0.518797
FTL_1711	253.9882	0.689749	0.182158	3.786538	0.000153	0.000569
FTL_1712	188.8175	1.67296	0.207308	8.069934	7.03E-16	7.64E-15
FTL_1713	2174.93	-0.14325	0.082053	-1.74581	0.080843	0.145605
FTL_1714	134428.8	-0.06147	0.054227	-1.13363	0.256949	0.372901
FTL_1715	16095.39	0.271986	0.05889	4.618546	3.86E-06	1.78E-05
FTL_1716	516.9792	1.370673	0.136081	10.07249	7.31E-24	1.31E-22
FTL_1717	1554.302	-0.13715	0.089407	-1.53402	0.125025	0.207001
FTL_1718	1093.442	0.160626	0.099009	1.622335	0.104732	0.17931
FTL_1719	311.1151	0.057944	0.165219	0.35071	0.725806	0.809135
FTL_1720	1392.751	-0.29026	0.092282	-3.14541	0.001659	0.005069
FTL_1721	2876.799	-0.43188	0.072104	-5.98976	2.10E-09	1.4E-08
FTL_1722	2676.885	-0.17232	0.074717	-2.30625	0.021097	0.047011
FTL_1723	1980.652	-0.14274	0.081035	-1.76152	0.078151	0.141389
FTL_1724	4338.13	-0.23117	0.071756	-3.22164	0.001275	0.004003
FTL_1725	2174.465	-0.09048	0.081435	-1.11105	0.266545	0.382036
FTL_1726	3655.643	-0.21642	0.072897	-2.96885	0.002989	0.008583
FTL_1727	702.159	-0.63184	0.11825	-5.34328	9.13E-08	5.05E-07
FTL_1728	1644.805	0.016018	0.085775	0.186749	0.851857	0.900228
FTL_1729	1930.688	-0.31294	0.080718	-3.87699	0.000106	0.000402
FTL_1730	313.0446	0.427566	0.165614	2.581704	0.009831	0.024128
FTL_1731	591.1905	0.416524	0.12537	3.322369	0.000893	0.002898
FTL_1732	1269.85	-0.00524	0.095697	-0.05479	0.956307	0.968839
FTL_1733	4005.219	0.133527	0.066749	2.000431	0.045454	0.089835
FTL_1734	3327.237	0.22844	0.077201	2.95903	0.003086	0.008799
FTL_1735	7914.92	-0.04785	0.059459	-0.80474	0.420972	0.546611
FTL_1736	17730.98	0.149066	0.065322	2.282024	0.022488	0.049835
FTL_1737	3960.935	0.013931	0.071034	0.196117	0.844519	0.894354
FTL_1738	5164.647	-0.03354	0.07098	-0.47255	0.636537	0.733624
FTL_1739	2501.807	0.431259	0.07966	5.413747	6.17E-08	3.48E-07
FTL_1740	24458.15	-0.49857	0.051398	-9.70021	3.01E-22	4.80E-21
FTL_1741	4484.23	-0.00748	0.069945	-0.10689	0.914875	0.940132
FTL_1742	599.4988	-0.0826	0.1298	-0.63634	0.524554	0.642116
FTL_1743	81983.71	-0.17267	0.048225	-3.58058	0.000343	0.001201
FTL_1744	75494.77	0.138891	0.052358	2.652729	0.007984	0.020036
FTL_1745	11948.01	0.04175	0.062271	0.670451	0.50257	0.624716
FTL_1746	22072.73	0.15785	0.06339	2.490152	0.012769	0.03023
FTL_1747	21242.86	0.15618	0.054128	2.885369	0.00391	0.010905
FTL_1748	20135.02	0.181974	0.06318	2.880224	0.003974	0.011033
FTL_1749	10706.76	-0.07522	0.058483	-1.28617	0.198383	0.301626
FTL_1750	4934.008	-0.23539	0.072811	-3.23293	0.001225	0.003866
FTL_1751	184270.7	0.326703	0.061424	5.318824	1.04E-07	5.70E-07

FTL_1752	421.0477	-0.08705	0.147816	-0.58889	0.555933	0.669518
FTL_1753	988.2185	-0.44847	0.1009	-4.44471	8.8E-06	3.91E-05
FTL_1754	756.7299	-0.47956	0.138356	-3.46609	0.000528	0.001799
FTL_1755	1933.308	-0.40828	0.087611	-4.66007	3.16E-06	1.46E-05
FTL_1756	5290.574	-0.40393	0.066009	-6.11926	9.40E-10	6.38E-09
FTL_1757	961.2664	0.217995	0.104165	2.09279	0.036368	0.074744
FTL_1758	165.5893	0.098075	0.206614	0.474677	0.635017	0.732526
FTL_1759	189.7335	-0.11044	0.203079	-0.54381	0.586574	0.695994
FTL_1760	50.36235	0.399029	0.303942	1.312847	0.189235	0.293716
FTL_1761	9.901623	0.284547	0.311399	0.913772	0.360837	0.484528
FTL_1762	1817.188	0.040042	0.085922	0.466023	0.641199	0.737727
FTL_1763	740.2474	0.018703	0.114918	0.162753	0.870713	0.913908
FTL_1764	349.1895	0.159106	0.152931	1.040383	0.298162	0.41851
FTL_1765	199.1933	-0.31196	0.19315	-1.6151	0.10629	0.18136
FTL_1766	83.39648	-0.42022	0.261225	-1.60866	0.10769	0.183127
FTL_1767	61.477	0.328643	0.285385	1.151578	0.249494	0.363394
FTL_1768	25.62987	0.32995	0.329781	1.000512	0.317063	0.440426
FTL_1769	50.80992	0.82584	0.301671	2.737555	0.00619	0.016095
FTL_1770	177.8201	0.600129	0.205633	2.918451	0.003518	0.009917
FTL_1771	539.1511	0.129923	0.134464	0.966233	0.333927	0.455356
FTL_1772	32063.04	0.342274	0.054645	6.263639	3.76E-10	2.70E-09
FTL_1773	1305.366	-0.15961	0.091956	-1.73576	0.082607	0.147985
FTL_1774	121.4474	-0.22964	0.235944	-0.9733	0.330404	0.452085
FTL_1775	1396.514	-0.1513	0.091162	-1.65973	0.096968	0.168022
FTL_1776	865.7764	0.254492	0.121067	2.10207	0.035547	0.073357
FTL_1777	268.8356	0.145717	0.169333	0.860532	0.389496	0.515396
FTL_1778	595.3664	0.24205	0.12367	1.957232	0.05032	0.098007
FTL_1779	2968.973	-0.13512	0.072205	-1.87129	0.061305	0.11592
FTL_1780	3957.305	-0.08259	0.065652	-1.25802	0.208383	0.313111
FTL_1781	7591.769	-0.13406	0.059158	-2.26606	0.023448	0.051735
FTL_1782	4091.336	-0.37542	0.0661	-5.67961	1.35E-08	8.17E-08
FTL_1783	20629.77	-0.31893	0.05139	-6.20611	5.43E-10	3.79E-09
FTL_1784	29394.26	-0.18516	0.049736	-3.72288	0.000197	0.000721
FTL_1785	13653.71	-0.00663	0.052678	-0.12587	0.899839	0.930389
FTL_1786	14758.16	0.039654	0.05383	0.736657	0.461331	0.583927
FTL_1787	2492.475	0.006604	0.0744	0.08876	0.929273	0.949781
FTL_1788	8500.236	0.064817	0.056032	1.156794	0.247357	0.361065
FTL_1789	33996.15	0.335515	0.059763	5.614115	1.98E-08	1.16E-07
FTL_1790	7074.119	-0.57642	0.07704	-7.48206	7.32E-14	7.07E-13
FTL_1791	6912.726	-0.03954	0.059706	-0.66226	0.507805	0.629277
FTL_1792	2145.326	0.021792	0.090215	0.241553	0.809127	0.869703
FTL_1793	5639.905	0.028158	0.060982	0.461742	0.644266	0.739563
FTL_1794	4178.8	-0.17928	0.070403	-2.54656	0.010879	0.026409

FTL_1795	16694.23	-0.15646	0.057963	-2.69936	0.006947	0.017698
FTL_1796	8847.751	-0.0598	0.065842	-0.9083	0.363718	0.487708
FTL_1797	22645.79	-0.09599	0.055637	-1.72524	0.084485	0.150375
FTL_1798	5145.563	-0.2934	0.062185	-4.7182	2.38E-06	1.13E-05
FTL_1799	4537.803	-0.1526	0.075215	-2.02889	0.042469	0.084686
FTL_1800	9834.879	-0.12635	0.056309	-2.2438	0.024845	0.054105
FTL_1801	7142.326	0.047822	0.068063	0.702621	0.482292	0.604746
FTL_1802	932.3573	0.032643	0.10679	0.305676	0.759851	0.835047
FTL_1803	2888.6	-0.27444	0.077859	-3.52485	0.000424	0.001466
FTL_1804	1342.988	-0.55926	0.092143	-6.06946	1.28E-09	8.60E-09
FTL_1805	1388.213	-0.56279	0.096156	-5.85288	4.83E-09	3.08E-08
FTL_1806	1549.015	-0.56112	0.089798	-6.24869	4.14E-10	3E-09
FTL_1807	3538.759	-0.44319	0.072143	-6.14323	8.09E-10	5.57E-09
FTL_1808	1047.73	-0.38935	0.099235	-3.92353	8.73E-05	0.000336
FTL_1809	15924.89	-0.08952	0.065992	-1.35646	0.174951	0.273872
FTL_1810	8930.191	-0.04212	0.060334	-0.69812	0.485099	0.606751
FTL_1811	4450.494	-0.18082	0.066748	-2.70894	0.00675	0.017305
FTL_1812	1344.817	-0.44916	0.09031	-4.97353	6.57E-07	3.29E-06
FTL_1813	44.59508	0.535907	0.306787	1.746836	0.080666	0.145416
FTL_1814	367.6156	0.515928	0.153088	3.370149	0.000751	0.002471
FTL_1815	923.5768	0.195048	0.106325	1.834449	0.066587	0.123926
FTL_1816	708.9067	-0.19559	0.115424	-1.69456	0.090159	0.158218
FTL_1817	9983.396	-0.06885	0.054634	-1.26021	0.207595	0.312511
FTL_1818	6114.631	-0.26864	0.059068	-4.548	5.42E-06	2.46E-05
FTL_1819	7903.898	-0.33609	0.060654	-5.54112	3.01E-08	1.75E-07
FTL_1820	833.7597	-0.21124	0.111076	-1.9018	0.057197	0.109076
FTL_1821	2243.627	0.027095	0.092571	0.292696	0.769755	0.840873
FTL_1822	1728.283	0.028978	0.084936	0.341178	0.732969	0.81396
FTL_1823	4353.372	0.06513	0.06971	0.934303	0.350148	0.471714
FTL_1824	13974.58	-0.00422	0.054964	-0.07681	0.938774	0.956864
FTL_1825	9447.414	0.12511	0.067849	1.843964	0.065188	0.121997
FTL_1826	2247.423	0.074724	0.077634	0.962517	0.33579	0.457585
FTL_1827	6851.647	0.195873	0.060671	3.228457	0.001245	0.003921
FTL_1828	4729.582	0.289595	0.065671	4.409808	1.03E-05	4.58E-05
FTL_1829	3738.904	0.21303	0.078704	2.706723	0.006795	0.017399
FTL_1830	3115.398	0.510696	0.075835	6.734311	1.65E-11	1.33E-10
FTL_1831	1005.008	-0.32367	0.101764	-3.18063	0.00147	0.00453
FTL_1832	10413.22	-0.09541	0.056095	-1.70096	0.08895	0.156421
FTL_1833	1789.976	-0.01502	0.091983	-0.16326	0.870314	0.913908
FTL_1834	1941.891	-0.28568	0.081877	-3.48915	0.000485	0.001662
FTL_1835	931.2304	-0.28439	0.102899	-2.76373	0.005715	0.014995
FTL_1836	545.7976	0.292675	0.129623	2.257895	0.023952	0.052502
FTL_1837	96.63968	0.04486	0.249129	0.180068	0.857099	0.903392

FTL_1838	80.29082	0.116755	0.263775	0.442632	0.658032	0.750649
FTL_1839	591.1936	-0.15273	0.13116	-1.16442	0.244252	0.357312
FTL_1840	2382.472	-0.26674	0.074725	-3.56954	0.000358	0.001241
FTL_1841	7133.465	-0.01615	0.058004	-0.27835	0.780746	0.849188
FTL_1842	8131.394	0.152103	0.057189	2.659628	0.007823	0.019729
FTL_1843	1307.976	0.22403	0.091925	2.437091	0.014806	0.034484
FTL_1844	154.1308	0.51845	0.237445	2.183455	0.029002	0.06151
FTL_1845	201.0065	0.930286	0.199015	4.674459	2.95E-06	1.37E-05
FTL_1846	67.94799	0.619614	0.27777	2.230673	0.025703	0.055791
FTL_1847	277.8198	-0.14336	0.167847	-0.85408	0.393059	0.519086
FTL_1848	1021.112	-0.06959	0.099482	-0.6995	0.484238	0.606051
FTL_1849	2612.086	-0.0961	0.073886	-1.30071	0.193359	0.297134
FTL_1850	3227.898	-0.30952	0.072786	-4.25254	2.11E-05	9.07E-05
FTL_1851	172.9191	0.246339	0.205865	1.196602	0.231462	0.341924
FTL_1852	572.4305	-0.05628	0.126147	-0.44617	0.655478	0.74816
FTL_1853	200.9223	0.087786	0.191327	0.458827	0.646358	0.741118
FTL_1854	649.7937	-0.04534	0.129991	-0.34879	0.727247	0.809394
FTL_1855	189.6679	-0.12175	0.203189	-0.59921	0.549032	0.665127
FTL_1856	35.33834	-0.02217	0.31899	-0.06952	0.944579	0.959772
FTL_1857	55.99914	0.45955	0.294208	1.561987	0.118291	0.197972
FTL_1858	218.4474	0.069882	0.187837	0.372034	0.709867	0.796669
FTL_1859	241.6688	-0.31367	0.179901	-1.7436	0.08123	0.146035
FTL_1860	4600.114	-0.33844	0.062768	-5.39182	6.97E-08	3.93E-07
FTL_1861	3763.045	-0.11294	0.072773	-1.55201	0.12066	0.201162
FTL_1862	166.7562	-0.08683	0.209195	-0.41506	0.678101	0.768743
FTL_1863	614.7276	-0.18701	0.144935	-1.29028	0.196952	0.299905
FTL_1864	995.5461	-0.14639	0.101202	-1.44653	0.148028	0.23796
FTL_1865	8047.187	0.179998	0.063203	2.847937	0.0044	0.01205
FTL_1866	3147.512	-0.03875	0.085919	-0.45095	0.652023	0.745923
FTL_1867	5840.065	-0.64203	0.067029	-9.57846	9.85E-22	1.55E-20
FTL_1868	3181.921	0.005676	0.072723	0.078056	0.937784	0.956864
FTL_1869	2736.88	-0.03545	0.077148	-0.4595	0.645872	0.740984
FTL_1870	379.1467	-0.04329	0.148362	-0.29179	0.770446	0.841172
FTL_1871	318.0749	-0.03566	0.162292	-0.21972	0.826087	0.878537
FTL_1872	355.2099	-0.01246	0.159964	-0.07791	0.937902	0.956864
FTL_1873	3391.306	-0.04629	0.068627	-0.67449	0.499998	0.621904
FTL_1874	2226.869	-0.20811	0.088528	-2.35081	0.018733	0.042593
FTL_1875	544.9562	-0.22731	0.128522	-1.76865	0.076952	0.139418
FTL_1876	434.4304	-0.50849	0.13988	-3.63517	0.000278	0.000995
FTL_1877	137.0102	-0.07641	0.221676	-0.34468	0.730335	0.811923
FTL_1878	797.7626	-0.24984	0.109625	-2.27909	0.022662	0.050149
FTL_1879	3470.077	-0.97166	0.068669	-14.1499	1.87E-45	8.36E-44
FTL_1880	150.4807	-0.18584	0.213725	-0.86951	0.38457	0.510222

FTL_1881	1053.207	-0.13702	0.100122	-1.36856	0.171137	0.268739
FTL_1882	2416.266	-0.26484	0.080609	-3.28547	0.001018	0.003248
FTL_1883	3462.144	-0.31779	0.067714	-4.69314	2.69E-06	1.27E-05
FTL_1884	32.27698	0.627231	0.324159	1.934949	0.052997	0.102525
FTL_1885	221.8273	0.343101	0.183715	1.86757	0.061822	0.116678
FTL_1886	836.6194	0.292271	0.111359	2.624595	0.008675	0.021473
FTL_1887	12.91378	0.477735	0.322443	1.481609	0.138444	0.22514
FTL_1888	47.60423	0.269275	0.301112	0.894268	0.371178	0.496056
FTL_1889	132.0243	0.484308	0.225337	2.149256	0.031614	0.06589
FTL_1890	8.435603	0.290635	0.308889	0.940906	0.346753	0.469027
FTL_1891	978.636	0.216456	0.103312	2.09518	0.036155	0.074382
FTL_1892	4747.781	-0.76964	0.080497	-9.56108	1.17E-21	1.80E-20
FTL_1893	59.61799	0.128839	0.285714	0.450939	0.652033	0.745923
FTL_1894	237.5178	0.26262	0.179988	1.459097	0.144538	0.233539
FTL_1895	131.6227	0.198043	0.222971	0.888204	0.374431	0.499683
FTL_1896	1078.588	0.37209	0.097714	3.807946	0.00014	0.000526
FTL_1897	692.9932	0.193516	0.121029	1.598914	0.10984	0.18631
FTL_1898	1356.708	-0.22868	0.102852	-2.2234	0.026189	0.056724
FTL_1899	9336.887	0.233172	0.058976	3.953653	7.70E-05	0.0003
FTL_1900	4651.84	0.017918	0.063773	0.280964	0.778738	0.847768
FTL_1901	754.7069	-0.32732	0.119184	-2.74635	0.006026	0.015751
FTL_1902	1787.957	-0.51558	0.082484	-6.25065	4.09E-10	2.92E-09
FTL_1903	6433.747	0.29871	0.060763	4.91601	8.83E-07	4.37E-06
FTL_1904	3048.825	0.23036	0.072624	3.171963	0.001514	0.004646
FTL_1905	3184.823	-0.15572	0.069474	-2.24148	0.024995	0.054373
FTL_1906	7859.261	-0.14598	0.067702	-2.1562	0.031068	0.064982
FTL_1907	13165.61	-0.05993	0.058216	-1.02939	0.303299	0.423943
FTL_1908	4239.764	-0.36986	0.068206	-5.42264	5.87E-08	3.33E-07
FTL_1909	1444.303	-0.17972	0.0917	-1.95991	0.050006	0.09749
FTL_1910	5796.534	-0.12835	0.063007	-2.037	0.041651	0.083384
FTL_1911	976.2644	0.258552	0.107217	2.411483	0.015888	0.036791
FTL_1912	60894.36	-0.06317	0.056038	-1.12735	0.259596	0.375386
FTL_1913	933.4713	-0.17985	0.106689	-1.68574	0.091846	0.16081
FTL_1914	20039.22	0.192438	0.052569	3.66068	0.000252	0.000906
FTL_1915	1111.121	0.081294	0.098911	0.821885	0.411142	0.536621
FTL_1916	812.6883	0.321925	0.116105	2.772695	0.005559	0.014684
FTL_1917	1486.708	0.518233	0.089304	5.803025	6.51E-09	4.09E-08
FTL_1918	459.6142	0.438501	0.139577	3.141643	0.00168	0.005124
FTL_1919	73.55331	0.72088	0.274395	2.627162	0.00861	0.021339
FTL_1920	43.28724	0.367185	0.319448	1.149435	0.250377	0.364151
FTL_1921	339.5811	0.009084	0.154936	0.05863	0.953247	0.966226
FTL_1922	556.1181	-0.28689	0.145848	-1.96703	0.04918	0.095997
FTL_1923	3127.072	-0.47904	0.073172	-6.5467	5.88E-11	4.60E-10

FTL_1924	600.0087	-0.1457	0.123268	-1.18198	0.237213	0.349047
FTL_1925	850.3712	0.270805	0.108105	2.505022	0.012244	0.029264
FTL_1926	157.7999	0.125381	0.209031	0.599821	0.548626	0.665101
FTL_1927	60.69495	-0.03402	0.284621	-0.11953	0.904856	0.932533
FTL_1928	166.8286	-0.1412	0.21597	-0.65379	0.513246	0.634065
FTL_1929	3288.098	-0.69124	0.078902	-8.76076	1.94E-18	2.44E-17
FTL_1930	7034.34	-0.35478	0.059276	-5.98512	2.16E-09	1.43E-08
FTL_1931	3130.896	-0.38651	0.071845	-5.37969	7.46E-08	4.19E-07
FTL_1932	480.4613	-0.20444	0.137078	-1.49141	0.135854	0.221825
FTL_1933	819.6663	-0.22655	0.10957	-2.06767	0.038671	0.078514
FTL_1934	1222.66	-0.08757	0.097862	-0.89486	0.370861	0.495962
FTL_1935	4583.085	0.101875	0.06528	1.560582	0.118622	0.198362
FTL_1936	9078.184	-0.05114	0.062479	-0.81856	0.41304	0.537701
FTL_1937	125.9167	-0.00817	0.234675	-0.03479	0.972244	0.978083
FTL_1938	1658.972	-0.04754	0.086918	-0.5469	0.584446	0.694289
FTL_1939	948.2634	0.095548	0.107621	0.887819	0.374638	0.499683
FTL_1940	1582.652	0.242464	0.09815	2.470336	0.013499	0.031771
FTL_1941	1163.611	0.107076	0.102067	1.049084	0.294139	0.41373
FTL_1942	556.3355	0.004975	0.127092	0.039146	0.968774	0.97508
FTL_1943	1141.789	-0.15304	0.096981	-1.57802	0.11456	0.192878
FTL_1944	935.2677	0.215925	0.104503	2.066212	0.038809	0.078714
FTL_1945	257.7126	-0.03038	0.181956	-0.16695	0.867407	0.91228
FTL_1946	871.6259	0.018078	0.119285	0.151549	0.879542	0.918379
FTL_1947	6883.987	0.121573	0.059654	2.037949	0.041555	0.083276
FTL_1948	370.7246	0.06494	0.150857	0.430475	0.66685	0.757699
FTL_1949	15.83892	0.245289	0.329849	0.743642	0.457093	0.58039
FTL_1950	351.5854	-0.05465	0.153491	-0.35604	0.721812	0.806023
FTL_1951	102.2748	-0.04197	0.24496	-0.17132	0.863974	0.909685
FTL_1952	193.2966	-0.41789	0.195081	-2.14215	0.032181	0.066892
FTL_1953	71.29241	-0.04609	0.276873	-0.16645	0.867801	0.91228
FTL_1954	143.4118	0.217909	0.222832	0.977907	0.32812	0.449878
FTL_1955	153.6746	0.412343	0.21522	1.915912	0.055376	0.106411
FTL_1956	2121.87	-0.48436	0.083529	-5.7987	6.68E-09	4.17E-08
FTL_1957	6868.416	0.242349	0.065528	3.698393	0.000217	0.00079
FTL_1958	5871.629	0.375726	0.066367	5.661325	1.5E-08	9.01E-08
FTL_1959	89.31863	0.532078	0.25957	2.049846	0.040379	0.081325
FTL_1960	820.2794	0.217365	0.109621	1.982883	0.047381	0.093003
FTL_1961	454.8956	-0.22865	0.13891	-1.64601	0.099761	0.171678
FTL_1962	364.4782	-0.28903	0.149814	-1.92926	0.053698	0.103583
FTL_1963	255.7882	-0.35257	0.174742	-2.01767	0.043626	0.086734
FTL_1964	1926.288	-0.14089	0.08943	-1.57543	0.115158	0.193696
FTL_1965	1603.355	-0.18066	0.091173	-1.98156	0.047529	0.093203
FTL_1966	5188.572	-0.04305	0.078115	-0.55117	0.58152	0.69286

FTL_1967	766.4778	-0.15543	0.111679	-1.39175	0.163999	0.259762
FTL_1968	681.5694	-0.27239	0.136211	-1.99978	0.045524	0.089885
FTL_R0001	652794.8	0.121844	0.244906	0.497513	0.618827	0.719779
FTL_R0002	273.9096	0.177845	0.169453	1.049528	0.293935	0.41373
FTL_R0004	119.5155	-0.01099	0.233366	-0.0471	0.962436	0.971621
FTL_R0005	208.4834	-0.04366	0.192957	-0.22629	0.820974	0.875416
FTL_R0008	77.06307	-0.22654	0.26772	-0.84619	0.397449	0.523165
FTL_R0009	393.4905	0.230797	0.146263	1.577957	0.114575	0.192878
FTL_R0010	53664.03	0.594356	0.053841	11.03919	2.47E-28	5.58E-27
FTL_R0011	784.7801	0.193816	0.113279	1.710957	0.087089	0.153822
FTL_R0012	1217.357	0.092609	0.09775	0.947411	0.34343	0.466501
FTL_R0013	42.99293	0.075907	0.307484	0.246865	0.805012	0.868532
FTL_R0014	259.6168	0.743526	0.174855	4.252237	2.12E-05	9.07E-05
FTL_R0015	612.9555	0.691229	0.129373	5.342898	9.15E-08	5.05E-07
FTL_R0017	119.5155	-0.01099	0.233366	-0.0471	0.962436	0.971621
FTL_R0018	208.4834	-0.04366	0.192957	-0.22629	0.820974	0.875416
FTL_R0021	508.89	0.660367	0.144746	4.562264	5.06E-06	2.31E-05
FTL_R0022	2479.267	-0.00048	0.083209	-0.00578	0.995391	0.99689
FTL_R0023	695.2833	-0.14535	0.132857	-1.09405	0.273933	0.389948
FTL_R0024	155.7879	-0.02157	0.220709	-0.09774	0.922142	0.945184
FTL_R0025	386.1751	0.072591	0.175424	0.413802	0.679019	0.769351
FTL_R0026	585.945	0.011591	0.14976	0.077395	0.93831	0.956864
FTL_R0027	88.91777	-0.21008	0.266959	-0.78693	0.43132	0.55574
FTL_R0029	119.5155	-0.01099	0.233366	-0.0471	0.962436	0.971621
FTL_R0030	208.4834	-0.04366	0.192957	-0.22629	0.820974	0.875416
FTL_R0033	31.51915	-0.02452	0.322276	-0.0761	0.939341	0.956957
FTL_R0034	6.506623	-0.10994	0.293472	-0.37463	0.707937	0.79539
FTL_R0035	471.9523	-0.15255	0.149278	-1.02189	0.306832	0.428584
FTL_R0036	1203.597	-0.06611	0.099935	-0.66156	0.50825	0.629441
FTL_R0037	1713.707	0.010324	0.083878	0.123087	0.902038	0.931226
FTL_R0038	1280.328	0.014579	0.095743	0.152273	0.878972	0.918379
FTL_R0039	670.3318	-0.00895	0.124015	-0.07215	0.942482	0.959184
FTL_R0040	114.095	-0.41385	0.239351	-1.72905	0.0838	0.149698
FTL_R0041	76.40869	-0.18259	0.270048	-0.67614	0.49895	0.620984
FTL_R0042	46.94297	0.137108	0.303661	0.451517	0.651617	0.745923
FTL_R0043	255.9728	1.341582	0.180262	7.442417	9.89E-14	9.42E-13
FTL_R0044	113.2439	0.076718	0.243118	0.315559	0.752337	0.829259
FTL_R0045	1269.438	0.289265	0.099164	2.917026	0.003534	0.009948
FTL_R0046	2846.671	0.208038	0.074712	2.784518	0.005361	0.014215
FTL_R0047	1148.047	0.101179	0.096202	1.051737	0.29292	0.412593
FTL_R0048	1215.407	0.060839	0.098289	0.618985	0.535926	0.653251
FTL_R0049	253.0713	-0.03436	0.17742	-0.19367	0.846433	0.895908
FTL_R0050	287.9103	0.019024	0.169105	0.112498	0.910429	0.937001

FTL_R0051	1186.242		-0.34358	0.115849	-2.96573	0.00302	0.008646
FTL_R0052	1494.833		-0.31435	0.108801	-2.88922	0.003862	0.010811
